# Stereoselective Synthesis of Carbon-Sulfur-Bridged Glycomimetics by Photoinitiated Thiol-Ene Coupling Reactions

**DOI:** 10.3390/ijms21020573

**Published:** 2020-01-16

**Authors:** Magdolna Csávás, Dániel Eszenyi, Erika Mező, László Lázár, Nóra Debreczeni, Marietta Tóth, László Somsák, Anikó Borbás

**Affiliations:** 1 Department of Pharmaceutical Chemistry University of Debrecen, Egyetem tér 1, H-4032 Debrecen, Hungary; csavas.magdolna@science.unideb.hu (M.C.); eszenyid@gmail.com (D.E.); mezo.erika@science.unideb.hu (E.M.); debreczeni.nora@science.unideb.hu (N.D.); 2 Department of Organic Chemistry, University of Debrecen, Egyetem tér 1, H-4032 Debrecen, Hungary; lazar.laszlo@science.unideb.hu (L.L.); toth.marietta@science.unideb.hu (M.T.); somsak.laszlo@science.unideb.hu (L.S.); 3 Doctoral School of Chemistry, University of Debrecen, Egyetem tér 1, H-4032 Debrecen, Hungary

**Keywords:** carbohydrate, disaccharide, glycomimetic, thioglycoside, *C*-glycoside, photochemical addition, thiyl radical, diastereoselective synthesis

## Abstract

Oligosaccharides and glycoconjugates are abundant in all living organisms, taking part in a multitude of biological processes. The application of natural *O*-glycosides in biological studies and drug development is limited by their sensitivity to enzymatic hydrolysis. This issue made it necessary to design hydrolytically stable carbohydrate mimetics, where sulfur, carbon, or longer interglycosidic connections comprising two or three atoms replace the glycosidic oxygen. However, the formation of the interglycosidic linkages between the sugar residues in high diastereoslectivity poses a major challenge. Here, we report on stereoselective synthesis of carbon-sulfur-bridged disaccharide mimetics by the free radical addition of carbohydrate thiols onto the exo-cyclic double bond of unsaturated sugars. A systematic study on UV-light initiated radical mediated hydrothiolation reactions of enoses bearing an exocyclic double bond at C1, C2, C3, C4, C5, and C6 positions of the pyranosyl ring with various sugar thiols was performed. The effect of temperature and structural variations of the alkenes and thiols on the efficacy and stereoselectivity of the reactions was systematically studied and optimized. The reactions proceeded with high efficacy and, in most cases, with complete diastereoselectivity producing a broad array of disaccharide mimetics coupling through an equatorially oriented methylensulfide bridge.

## 1. Introduction

Carbohydrates, in the form of oligosaccharides, polysaccharides, and glycoconjugates, are ubiquitous in nature and play crucial roles in a wide range of intercellular recognition events, including adhesion, signaling, trafficking, immune response, metastasis, inflammation, as well as bacterial and viral infections [1,2,3]. Due to these important biological functions, the development of sugar-based drugs could be of great pharmaceutical interest [4,5]. However, the sensitivity of the native glycosidic bond to chemical and enzymatic degradation hinders the therapeutic application of carbohydrates [6].

**Scheme 1:**
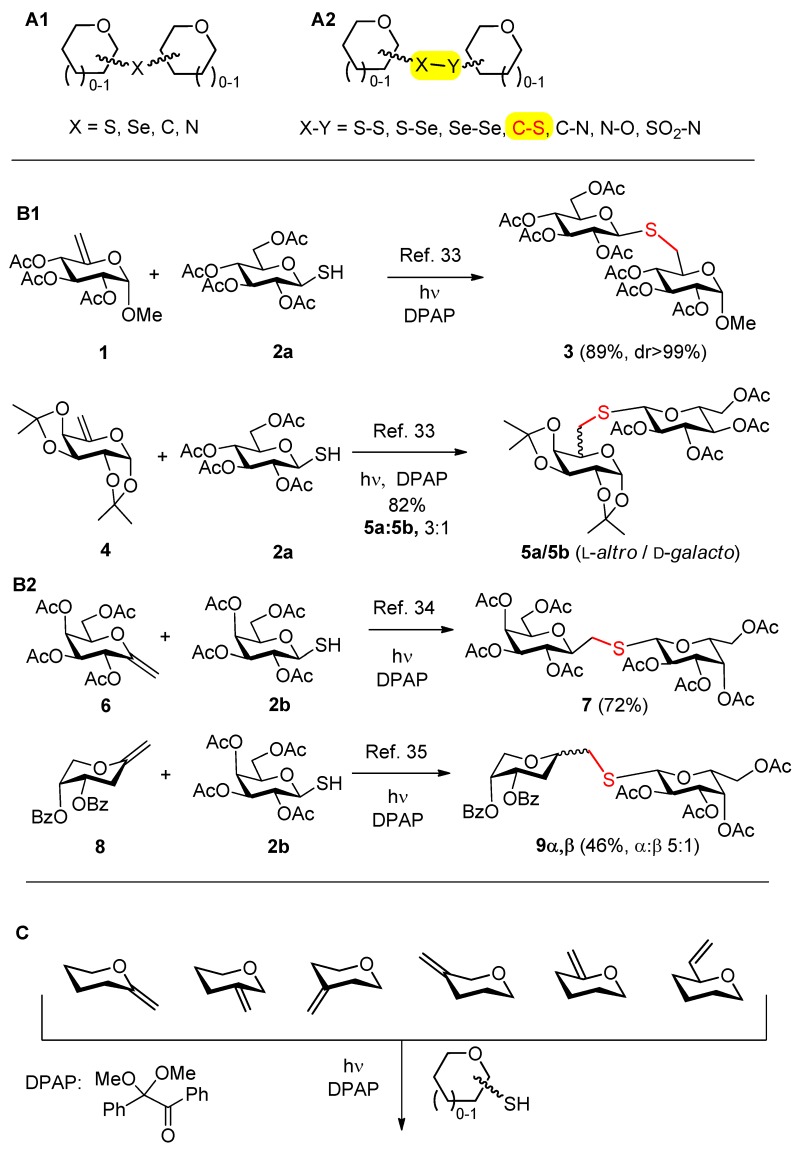
(**A**) Disaccharide mimetics with two-bond and three-bond linkages. Yellow background indicates the three-bond connection, and the *C*-*S* bond discussed in this study is highlighted in red. (**B**) Literature results on the synthesis of *C*-*S*-bridged disaccharide mimetics. The newly formed C-S bonds are highlighted in red. (**C**) This work: systematic study on UV-light induced hydrothiolation reactions of enopyranoses bearing an exocyclic double bond at C1, C2, C3, C4, C5, and C6 positions.

A broad variety of carbohydrate mimetics with various unnatural glycosidic linkages have been prepared to address this issue [6,7,8,9,10,11]. The replacement of glycosidic oxygen by carbon, sulfur, selenium, or nitrogen is the most obvious way to produce derivatives with increased stability, while maintaining the biological properties of the natural compounds (Scheme 1(A1)). In recent years, numerous extended linkage modes that consist of two (or even more) bridging atoms in the place of a native *O*-glycosidic bond have been designed (Scheme 1(A2)) [7,8]. The extended, three-bond interglycosidic connections, including S-S [12,13,14,15], Se-S, Se-Se [16], C-S [17,18], C-N [19], N-O [20], and SO_2_-N [21] bonds, provide specific conformational properties, and, in some cases, advantageous binding capabilities to carbohydrate mimetics. 

The carbon-sulfur-bridged oligosaccharides may be of particular interest, because they may combine the beneficial properties of the two most common carbohydrate mimetics, thioglycosides [8,9], and *C*-glycosides [10,11,22]. 

The radical-mediated addition of thiols to non-activated double bonds is widely used in organic syntheses, material sciences, and glycochemistry as a robust ligation tool [23,24,25,26]. Recent results have demonstrated that applying unsaturated sugars as the alkene partners in the thiol-ene coupling reactions offers a rapid route to a broad range of thio-linked glycomimetics, including 1,2-cis α-*S*-glycosides [27,28,29,30,31], 2-thio-β-mannosides [32] as well as *C*-*S*-bonded carbohydrate mimetics, wherein the CH_2_-S linkage is attached to the C1 or the C5 position of the pyranosyl ring [33,34,35] (Scheme 1B). Dondoni and co-workers reported that, while photoinduced hydrothiolation of 5-exomethylene pyranoside **1** with 1-thioaldose **2a** furnished the corresponding *S*-linked disaccharide **3** with complete regio- and stereoselectivity, addition reaction to galactopyranosyl 5-exomethylene **4** occured with only moderate stereoselectivity, yielding a 3:1 mixture of **5a** and **5b** (Scheme 1(B1)) [33]. Our group and Somsák’s group investigated the synthesis of glycosylmethyl-sulfide type disaccharide derivatives by thiol-ene reactions of several hexo- and pentopyranosyl *exo*-glycals. (Scheme 1(B2)) [34,35]. Addition to the *exo*-galactal derivative **6** provided the pseudodisaccharide **7** with exclusive β-selectivity [34]. However, upon hydrothiolation of the pentopyranosyl 2-deoxy-*exo*-glycal, a decreased stereoselectivity was observed, which afforded an anomeric mixture of **9** in favour of the α-configured product [35]. These results demonstrated that the enose structure greatly influences the stereoselectivity and the efficacy of the additions. We decided to perform a systematic study on UV-light initiated radical mediated hydrothiolation reactions of enoses bearing an exocyclic double bond at C1, C2, C3, C4, and C5 positions of the hexopyranosyl ring, as thiol-ene reactions of C2, C3, and C4 exomethylene derivatives of pyranosides have not been reported. Moreover, the hydrothiolation of a galactose-derived hept-6-enopyranose to produce *C*-*S*-bridged analogue of the biorelevant *N*-acetyl-neuraminic-acid-α(2,6)-d-galactose motif is also reported (Scheme 1C).

## 2. Results

**Figure 1:**
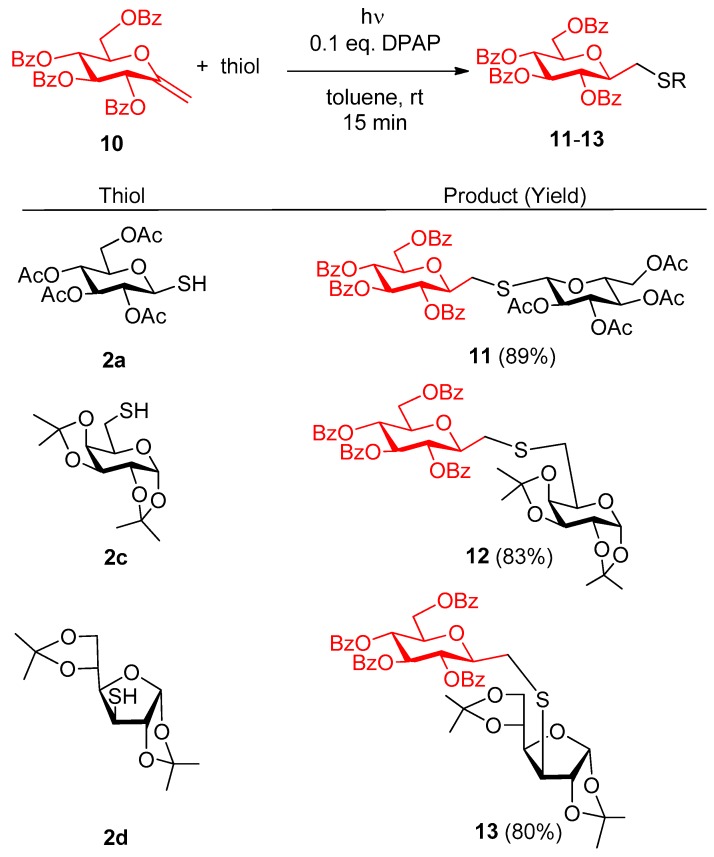
Stereoselective synthesis of glycosylmethyl sulfide type glycomimetics by thiol-ene reactions of *exo*-glucal **10**.

The perbenzoylated *exo*-glucal **10** [36,37,38] was reacted with thiols **2a**, **2c**, and **2d** while applying the optimized conditions established in our recent work for hydrothiolation reactions of unsaturated sugars [27,28,29]. Thus, the reactions were carried out in toluene at room temperature with a 1.5:1 thiol:ene ratio by irradiation at λ_max_ = 365 nm in the presence of 2,2-dimethoxy-2-phenylacetophenone (DPAP, 0.1 equiv) as the cleavable photoinitiator [24]. The addition of 1-thiosugar **2a**, 6-thio-galactopyranose **2c**, as well as 3-thio-glucofuranose **2d**, went to completion within 15 min. to provide the β-*C*-*S*-bonded disaccharide mimetics **11**–**13** with exclusive regio- and stereoselectivity (Figure 1).

**Scheme 2:**
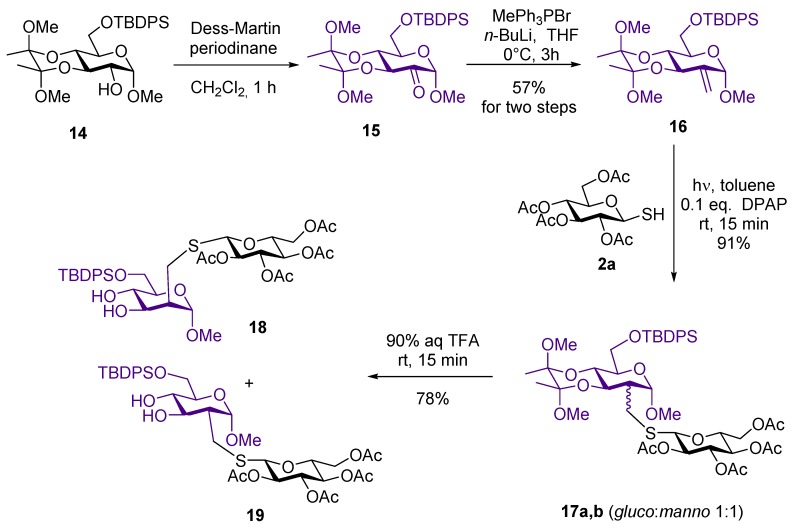
Hydrothiolation reaction of the glucose-derived enoside **16** bearing a C2-exomethylene group.

**Scheme 3:**
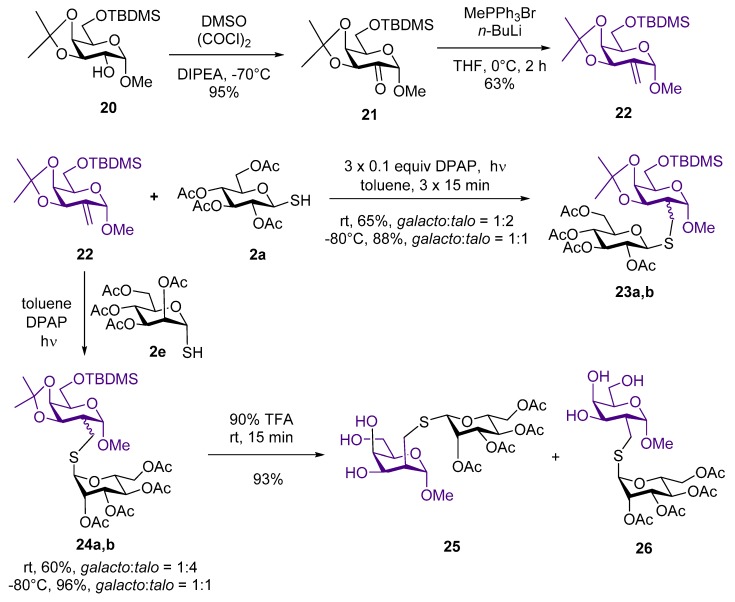
Addition of thiols onto the C2-exocyclic double bond of the galactose derived **22**.

**Scheme 4:**
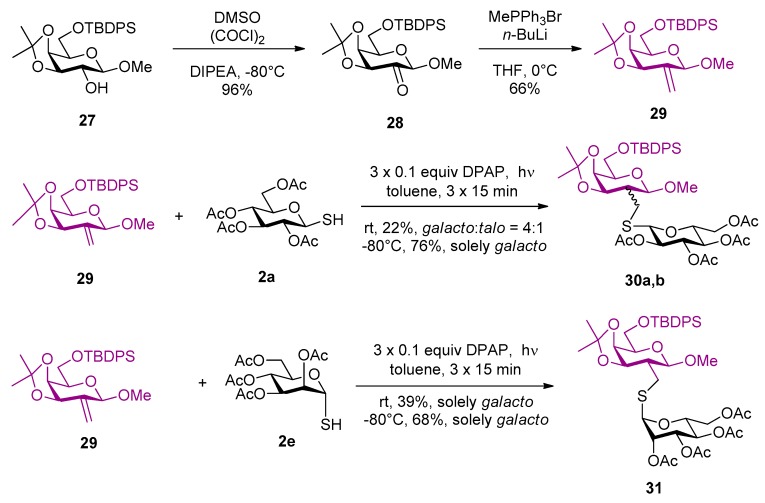
Hydrothiolation reactions of the β-configured C2-exomethylene enoside **29**.

Next, thiol-ene reactions of enopyranosides bearing an exomethylene moiety at the C2 position were studied (Scheme 2, Scheme 3 and Scheme 4). The properly protected methyl α-d-glucopyranoside derivative **14** [39] was converted into the C2-exomethylene derivative **16** in two steps, including oxidation with Dess–Martin periodinane [40] and olefination of the resulting 2-ulose **15** by a Wittig reaction (Scheme 2). The addition of 1-thioglucose tetra-*O*-acetate **2a** onto 2-exomethylene **16** at room temperature readily occurred to result in an inseparable 1:1 mixture of the d-*gluco* and d-*manno* configured thiodisaccharides (**17a**,**b**) with 91% yield. After the acidic removal of the butane-2,3-diacetal (BDA) group, the partially protected diastereoisomeric glycomimetics **18** and **19** were successfully separated and characterized.

The methyl α-D-galactopyranoside derivative **22** with a C2 exocyclic double bond was also synthesized, starting from compound **20,** in order to study the effect of the enose configuration on the stereochemical outcome of the hydrothiolation reaction [41] (Scheme 3). Swern-oxidation [42] of **20**, followed by Wittig-olefination of the resulting 2-ulose **21** furnished **22** with good overall yield. Addition of β-1-thiosugar **2a** onto the galactose-derived enoside **22** at rt occurred with lower efficacy than in the *gluco*-case and led again to the formation of an inseparable diastereoisomeric mixture of the corresponding axially and equatorially coupled *C*-*S*-bonded disaccharides **23a** and **23b**. In this case a moderate selectivity was observed in favour of the d-*talo*-configured product. Recently, we have found that cooling was advantageous to the thiol-ene reactions of enosides to increase the yields [29], and, in the case of enofuranosides and pentopyranosyl endoglycals, to raise the stereoselectivity significantly [18,31]. Indeed, conducting the reaction between **22** and **2a** at −80 °C the yield of **23a**,**b** reached 88%. However, surprisingly, a complete lack of stereoselectivity was observed at this temperature. 

Reacting **22** with α-1-thiomannose derivative **2e** at rt occurred with a higher d-*talo* selectivity, which resulted in a 4:1 mixture of the *C*-*S*-bonded glycomimetics **24a** and **24b**. The cooling was again beneficial to the efficacy and detrimental to the diastereoselectivity of the reaction affording the d-*talo* and d-*galacto* configured products in a 1:1 ratio in 96% yield. The partial deprotection of **24a,b** with TFA gave a mixture of **25** and **26**, from which the d-*talo* configured **25** could be isolated in the pure form.

For studying the impact of the anomeric configuration on the stereochemical outcome of the thiol-ene reaction, compound **29**, the β-analogue of **22**, was prepared from methyl β-d-galactoside **27** [43] via oxidation and Wittig-olefination (Scheme 4). Surprisingly, the addition of **2a** and **2e** onto **29** at rt proceeded with low efficacy resulting in the *C*-*S* bridged products **30** and **31** in low yields. On the other hand, an opposite and increased stereoselectivity was observed with both thiols when compared to the α-configured enoside **22**. Using **2a** as the thiol, a 4:1 mixture was formed at rt in favour of the *galacto*-configured product with 22% yield, while complete *galacto* selectivity was observed with thiol **2e,** albeit the yield was only moderate at rt. Running the reaction at −80 °C significantly increased the yields and exclusive formation of the d-*galacto* configured products was observed with both thiols.

**Scheme 5:**
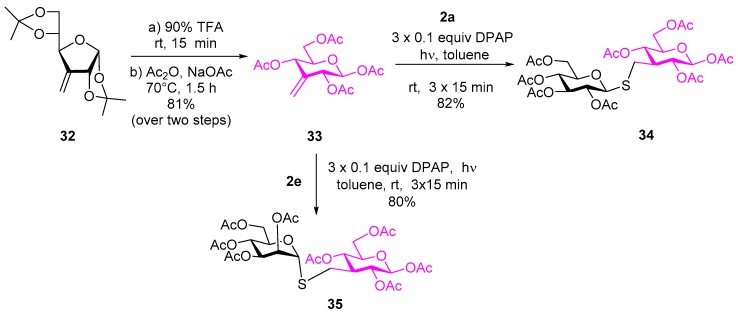
Thiol-ene reactions of enoside with a C3-exomethylene moiety.

Our investigation was continued with the hydrothiolation of the pyranosyl C3-exomethylene **33** (Scheme 5). The deprotection of glucofuranosyl 3-exomethylene **32** [44] while using TFA, followed by Ac_2_O-NaOAc mediated acetylation furnished the pyranose derivative **33** [45] in β-anomeric form. The hydrothiolation of **33** with thiols **2a** and **2e** occurred with full stereoselectivity and high yields producing the thiodisaccharides **34** and **35** with an equtorial carbon-sulfur linkage at position C3.

**Scheme 6:**
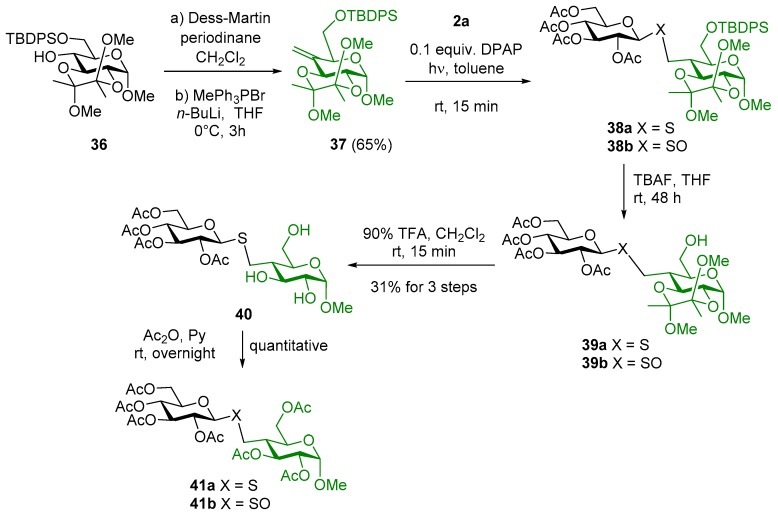
Addition of 1-thioglucose **2a** onto the C4-positioned exocyclic double bond of enopyranoside **37**.

**Scheme 7:**
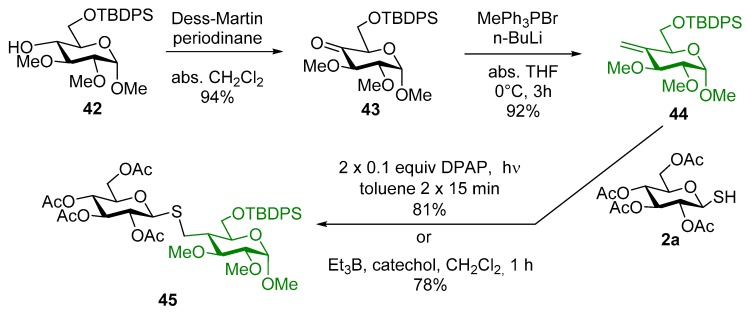
The addition of 1-thioglucose **2a** onto the C4-exomethylene derivative **44** using different initiation methods.

Next, we turned our attention to hydrothiolation of pyranoses containing a C4 exocyclic double bond (Scheme 6 and Scheme 7). The oxidation of the methyl α-d-glucopyranoside derivative **36** [39] at position 4, followed by Wittig-olefination resulted in the unsaturated sugar **37** bearing 6-*O*-silyl and 2,3-*O*-butane diacetal protecting groups (Scheme 6). The hydrothiolation of **37** with 1-thioglucose peracetate **2a** went to completion within 15 min. to result in an inseparable mixture of two compounds. On the basis of NMR and MS data, the components of the obtained mixture were tentatively identified as the expected disaccharide mimetic **38a** and its sulfoxide derivative **38b**, although at this stage of the study the configuration of the C4 stereocenter of the methyl glucopyranoside residue was uncertain. The attempted separation of compounds **39a** and **39b** obtained by TBAF-mediated desilylation failed. Fortunately, after deacetalation while using TFA, the major product was isolated in pure form and, after acetylation, undoubtedly identified as the the equatorially 4-*C-S*-bonded disaccharide mimetic. Interestingely, the acetylation of compound **40** led again to an inseparable mixture of the corresponding fully protected thiodisaccharide **41a** and its sulfoxide derivative **41b**.

We were curious as to whether the susceptibility to oxidation of **38** was due to the C4 position of the *C*-*S* bond or the substitution pattern of the enoside reactant. Therefore, C4-exomethylene **44**, a 2,3-di-*O*-methylated analogue of **37**, was prepared from **42** [46] via the oxidation-Wittig-olefination reaction sequence (Scheme 7).

UV-light initiated addition of thiol **2a** onto **44** occured with complete diastereoselectivity to provide the corresponding equatorially coupled disaccharide mimetic **45** with 81% yield. The addition between **2a** and **44** was also elicited by using triethylborane in combination with catechol, which was recently reported by Renaud and co-workers as an efficient reagent system for radical hydrothiolation of allylic double bonds [47]. The reaction took place with similar efficacy to the one that was observed in the case of photoinitiation and the formation of sulfoxide was not observed in either case.

**Scheme 8:**
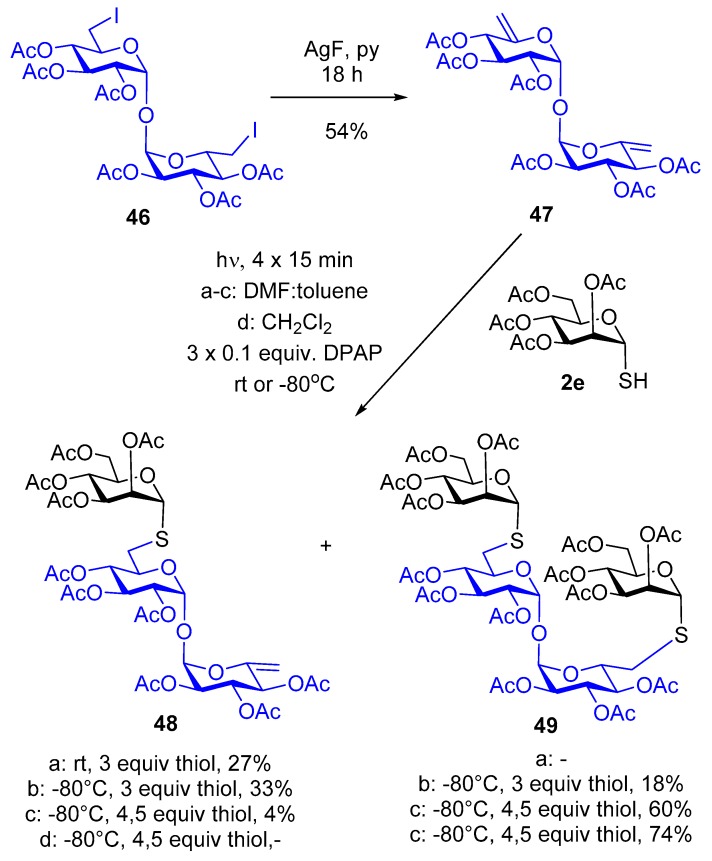
Synthesis of higher oligosaccharides by photoinitiated hydrothiolation of disaccharide dienoside **47** with different thiol-ene ratios and different temperatures.

To push the scope of the reaction further, we extended our study to disaccharide **47** bearing exocyclic double bonds at positions C5 and C5′ (Scheme 8). The 6,6′-diiodo trehalose derivative **46** [48] was treated with AgF [49] in pyridine to afford the 5,5′-dienoside **47** with 54% yield. The hydrothiolation of **47** with 1-thiomannose derivative **2e** at rt while using the usual thiol:alkene ratio (1.5 equiv thiol/double bond) resulted in the thiotrisaccharide **48** as the major product (27%) and the expected dithiotetrasaccharide **49** could not be isolated in pure form. The reaction was carried out in a mixture of DMF and toluene due to the low solubility of **47** in toluene. Conducting the reaction at −80 °C increased the yield and provided 33% of **48** and 18% of **49**. Raising the thiol excess to 4.5 equivalents (2.25 equiv. thiol/double bond) and running the addition reaction at −80 °C afforded **49** (60%) as the major product, along with 4% of **48**. Changing the solvent to CH_2_Cl_2_, a more clean and efficient reaction was observed, providing **49** with 74% yield. As the trisaccharide enoside **48** can be subjected to further hydrothiolation reaction with different thiosugars, the hydrothiolation of a dienoside offers the possibility for homo- and heterodisubstitution, depending on the thiol excess applied. 

**Scheme 9:**
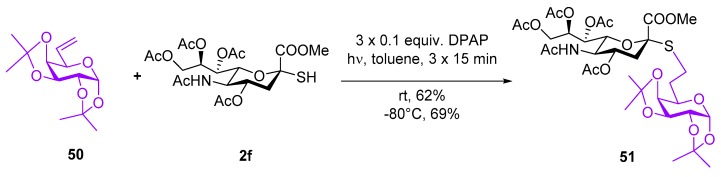
Rapid, thio-click route to the biorelevant sialyl galactoside mimetic **51**.

Finally, the thiol-ene reaction was utilized for the synthesis of a *C*-*S*-bridged analogue of the biorelevant *N*-acetyl-neuraminic-acid-α(2,6)-d-galactose disaccharide sequence [50,51,52]. The galacto-configured 6,7-enopyranose **50** [53,54,55] was reacted with 2-thio-neuraminic acid derivative **2f** to achieve this goal (Scheme 9). The reaction afforded the expected sialyl galactoside mimetic **51** with 62% yield at rt and a slightly increased 69% yield at −80 °C. 

## 3. Discussion

We prepared nine enopyranosyl derivatives with an exocyclic double bond at C1, C2, C3, C4, C5, and C6 positions and studied their UV-initiated hydrothiolation reactions while using various sugar thiols. The reactions, except for the C2-exomethylene cases, took place with complete regio- and stereoselectiviy providing the corresponding equatorial *C-S*-bonded di- and oligosaccharide mimetics with high yields. 

**Scheme 10:**
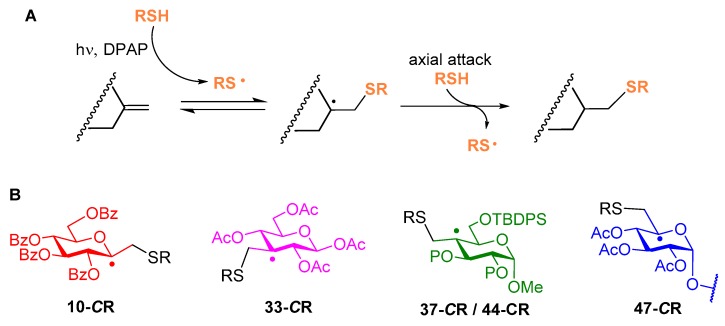
(**A**) Reversible thiyl addition (propagation) step and irreversible hydrogen abstraction (chain transfer) step upon addition of thiols onto exocyclic double bonds. (**B**) Carbon-centered radical intermediates with an equatorial methylenesulfide moiety formed by addition of a thiyl radical to C1-, C3-, C4-, or C5-positioned exomethylene group.

The thiol-ene reaction proceeds through a reversible thiyl addition (propagation) step, followed by an irreversible hydrogen abstraction (chain transfer) step by the carbon centered radical intermediate formed (Scheme 10A) [56,57,58]. While the addition of thiyl radicals to unsymmetrically substituted linear alkenes generally exhibits poor stereoselectivity, in the case of substituted cyclic olefins with an endocyclic double bond, the addition is known to preferentially occur in a trans-diaxial manner as the result of a kinetically favored axial attack of the thiyl radical onto the cyclic alkene in its half-chair conformation together with a stereoselective hydrogen abstraction from the thiol into an axial position [27,28,29,30,31,59,60]. We assume that, in the case of *exo*-glycal **10** and C3-, C4-, and C-5 exomethylenes **33**, **37**, **44**, and **47**, respectively, the thiol-ene reactions exclusively occur through the stable ^4^*C*_1_ chair conformer of the corresponding carbon-centered radical (Scheme 10B). Axial H-abstraction by these radicals from a thiol leads to the formation of the equatorial *C-S* interglycosidic linkages. Other possible carbon-centered radical intermediates of higher energy rather decompose in an intramolecular reaction, instead of forming the final product intermolecularly, due to the rapidly reversible nature of the thiyl addition step [58].

**Scheme 11:**
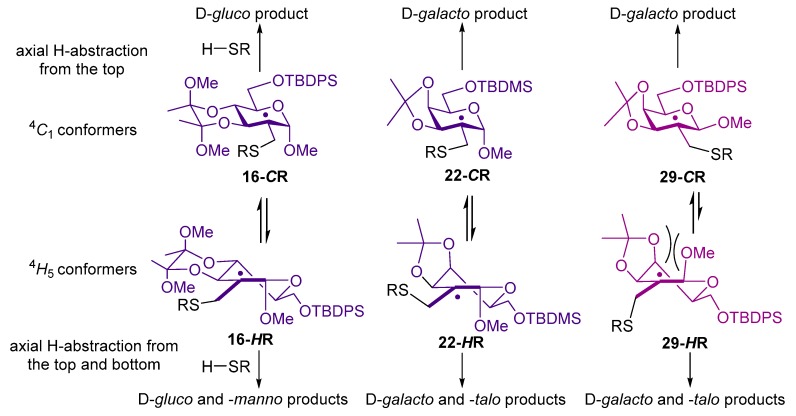
Assumed conformations of the C2-centered radicals and the configuration of the possible products formed from these radicals upon axial H-abstraction.

We have found that the hydrothiolation of the C2-exomethylene derivatives **16**, **22**, and **29** led to diastereoisomeric mixtures of disaccharide mimetics linked through axial and equatorial methylenesulfide bonds. We assume that, in these cases, the reaction can proceed through both the ^4^*C*_1_ chair and ^4^*H*_5_ half-chair conformers of the C2-centered radical bearing an equatorial or a quasi equatorial C2 substituent (Scheme 11). The equatorially *C-S*-linked products can be formed either through the ^4^*C*_1_ or the ^4^*H*_5_ conformation of the C2 radicals via axial H-abstraction from the upper face. At the same time, axial H-abstraction by the ^4^*H*_5_ conformer from the bottom face might lead to the formation of the epimeric disaccharides with an axial interglycosidic connection at position C2. The ratio of products showed great variation, depending on the configuration of enosides and thiols, as well as the temperature. The different stereoselectivity that was observed with the different thiosugars can be explained by the different fitting of the C2 radicals to thiols of different configurations. This phenomenon, which is known as double stereodifferentiation, is well-documented in the field of chemical glycosylation [61,62]. In the case of the β-configured **29**, the addition reactions occurred with remarkable or complete d-*galacto*-selectivity, which was probably due to the 1,3-diaxial repulsion between the aglycon and the C4-substituent in the ^4^*H*_5_ conformation, which increases the energy, thereby decreasing the lifetime of this conformer.

According to our previous results [27,28,29,30,31], cooling was beneficial to the efficacy of the additions, which can be explained by the rapid reversibility of the thiyl addition step. At higher temperatures, the dissociation of the carbon-centered radical is entropically favored, which shifts the equilibrium toward reactants before the carbon-centered radical can be trapped through hydrogen abstraction fom a thiol, thus reducing the overall conversion. Conducting the reaction at −80 °C increases the life-time of the intermediate radical, allowing it to react with a thiol in the irreversible hydrogen abstraction step and, thus, increasing the overall conversion.

Conducting the reactions at low temperature also modified the stereoselecivity of the reactions by shifting the product ratio toward the stereoisomer, the formation of which required lower transition state energy.

We demonstrated that the thiol-ene coupling reaction can successfully be extended to disaccharide dienoside **47**, which opens the way for a rapid synthesis of higher thio-oligosaccharides under mild conditions. The practical utility of the presented method was also demonstrated by the efficient synthesis of **51**, a novel thio-linked analogue of the sialyl-α(2,6)-galactoside structure that is of high biological importance. 

## 4. Materials and Methods

### 4.1. General Methods

The carbohydrate thiols **2a** [63], **2b** [64], **2c** [65], **2d** [66], **2e** [67], and **2f** [68] were prepared according to the literature procedures; 2,2-dimethoxy-2-phenylacetophenone (DPAP) was purchased from Sigma–Aldrich Chemical Co. (St. Louis, MO, USA) Optical rotations were measured at room temperature with a Perkin–Elmer 241 automatic polarimeter. Thin-layer chromatography (TLC) was performed on Kieselgel 60 F_254_ (Merck) with detection by immersing into 5% ethanolic sulfuric acid solution, followed by heating. Column chromatography was performed on Silica gel 60 (Merck 0.063–0.200 mm). Organic solutions were dried over MgSO_4_ and concentrated in vacuum. The ^1^H NMR (360 and 400 MHz) and ^13^C NMR (90.54 and 100.28 MHz) spectra were recorded with Bruker DRX-360 and DRX-400 spectrometers at 25 °C. Chemical shifts are referenced to Me_4_Si or DSS (0.00 ppm for 1H) and to the solvent signals (CDCl_3_: 77.00 ppm for ^13^C). ESI-QTOF MS measurements were carried out on a maXis II UHR ESI-QTOF MS instrument (Bruker), in positive ionization mode. The following parameters were applied for the electrospray ion source: capillary voltage: 3.6 kV; end plate offset: 500 V; nebulizer pressure: 0.5 bar; dry gas temperature: 200 °C and dry gas flow rate: 4.0 L/min. The MS method was tuned according to the examined mass range, which was 200–1000 *m*/*z*. Constant background correction was applied for each spectrum, and the background was recorded before each sample by injecting the blank sample matrix (solvent). Na-formate calibrant was injected after each sample, which enabled internal calibration during data evaluation. Mass spectra were recorded by otofControl version 4.1 (build: 3.5, Bruker) and processed by Compass DataAnalysis version 4.4 (build: 200.55.2969). MALDI-TOF MS analyses of the compounds were carried out in the positive reflectron mode using a BIFLEX III mass spectrometer (Bruker, Germany) equipped with delayedion extraction. The matrix solution was a saturated 2,4,6-trihydroxy-acetophenone (THAP) solution in MeCN. Elemental analyses (C, H, S) were performed while using an Elementar Vario MicroCube instrument.

### 4.2. Synthesis

#### 4.2.1. General Method for Photoinduced Addition of Thiols to Exoglycals or Sugar Exomethylene Derivatives

Sugar thiol (1.2–1.5 equiv.) and 2,2-dimethoxy-2-phenylacetophenone (DPAP, 0.10 equiv/alkene) were added to a solution of the starting unsaturated monosaccharide in dry toluene (it is indicated when some other solvent was used) (7–8 mL/1 mmol alkene). The solution was irradiated at room temperature (it is indicated when the reaction was performed at lower temperature) for 15 min. The progress of the reaction was monitored by TLC after this reaction period and irradiation and addition of DPAP were repeated if necessary, once or twice more. In these cases, no additional thiol was added to the reaction mixture. Subsequently, the solution was concentrated and the residue was purified by column chromatography or flash column chromatography.

#### 4.2.2. 2,6-Anhydro-3,4,5,7-tetra-*O*-benzoyl-1-deoxy-1-*S*-(2′,3′,4′,6′-tetra-*O*-acetyl-β-d-glucopyranosyl)-1-thio-d-*glycero*-d-*gulo*-heptitol (**11**)

*Exo*-glucal **10** (50 mg, 0.084 mmol) and thiol **2a** (44 mg, 0.12 mmol) were reacted according to the general method. The crude product was purified by silica gel chromatography in hexane/ethyl acetate 6/4 to give compound **11** (72 mg).

Yield: 89%, white foam. [α]_D_^20^ = −14.6 (*c* 0.2, CHCl_3_). R_f_ 0.45 (CH_2_Cl_2_/acetone 95/5 ). ^1^H NMR (360 MHz, CDCl_3_): δ = 8.10–7.79 (m, 8H, arom), 7.59-7.24 (m, 12H, arom), 5.89 (t, *J* = 9.6 Hz, 1H), 5.70 (t, *J* = 9.8 Hz, 1H), 5.54 (t, *J* = 9.7 Hz, 1H), 5.25 (t, *J* = 9.6 Hz, 1H), 5.09 (t, *J* = 9.7 Hz, 1H), 4.48 (t, *J* = 9.8 Hz, 1H), 4.73 (d, *J* = 10 Hz, 1H, H-1′), 4.72 (dd, *J* = 9.6, 2.1 Hz, 2H), 4.50 (dd, *J* = 12.2, 5.2 Hz, 1H), 4.24–4.02 (m, 4H), 3.70–3.65 (m, 1H), 3.13 (dd, *J* = 14.6, 9.0 Hz, 1H, SC*H*_2a_), 2.81 (dd, *J* = 14.6, 2.5 Hz, 1H, SC*H*_2b_), 2.11 (s, 3H, COC*H_3_*), 2.05 (s, 3H, COC*H_3_*), 2.01 (s, 3H, COC*H_3_*), 1.98 (s, 3H, COC*H_3_*). ^13^C NMR (91 MHz, CDCl_3_): δ = 170.6, 170.0, 169.3, 169.3, 166.0, 165.8, 165.1, 165.1 (8C, 8×*C*O), 133.4-128.2 (24 C, arom), 82.9, 78.5, 76.3, 75.7, 74.3, 73.8, 71.4, 70.2, 69.4, 68.1 (10C, skeleton carbons), 62.8, 61.7 (C-7, C-6’), 30.5 (S*C*H_2_), 20.7, 20.5, 20.5, 20.5 (4C, 4×CO*C*H_3_). MS (ESI-TOF) *m*/*z*: [M + Na]^+^ Calcd for C_49_H_48_O_18_NaS 979.2439; Found 979.2459; [M + Na]^+^.

#### 4.2.3. 2,6-Anhydro-3,4,5,7-tetra-*O*-benzoyl-1-deoxy-1-*S*-(1′,2′:3′,4′-di-*O*-isopropylidene-α-d-galactopyranose-6′-yl)-1-thio-d-*glycero*-d-*gulo*-heptitol (**12**)

*Exo*-glucal **10** (59 mg, 0.10 mmol) and thiol **2c** (42 mg, 0.15 mmol) were reacted according to the general method. The crude product was purified by silica gel chromatography in CH_2_Cl_2_/acetone 95/5 to give compound **12** (70 mg).

Yield: 83%, colorless syrup. [α]_D_^20^ = −11.4 (*c* 0.9, CHCl_3_). R_f_ 0.45, (CH_2_Cl_2_/acetone 9/1). ^1^H NMR (360 MHz, CDCl_3_): δ = 8.19–7.73 (m, 8H, arom.), 7.64–7.15 (m, 12H, arom.), 5.89 (t, *J* = 9.6 Hz, 1H), 5.63 (t, *J* = 9.6 Hz, 1H), 5.50 (t, *J* = 9.6 Hz, 1H), 5.45 (d, *J* = 5.1 Hz, 1H, H-1′), 4.64 (dd, *J* = 12.2, 2.8 Hz, 1H), 4.52 (dd, *J* = 7.9, 2.3 Hz, 1H), 4.44 (dd, *J* = 12.2, 5.2 Hz, 1H), 4.25 (dd, *J* = 5.1, 2.4 Hz, 1H), 4.21 (dd, *J* = 7.9, 1.8 Hz, 1H), 4.18–4.09 (m, 1H), 4.10–3.98 (m, 1H), 3.85 (t, *J* = 6.2 Hz, 1H), 3.00–2.73 (m, 4H, 2×SC*H_2_*); 1.50 (s, 3H ,C*H_3_*), 1.38 (s, 3H ,C*H_3_*), 1.27 (s, 3H ,C*H_3_*), 1.26 (s, 3H, C*H_3_*); ^13^C NMR (91 MHz, CDCl_3_): δ =166.3, 166.0, 165.5 and 165.4 (4C, 4×*C*OPh), 133.5, 133.3, 133.2, 130.0, 129.8, 129.0, 128.5 and 128.4 (24C, arom.), 109.3 and 108.6 (2C, 2×C_q_, *i*-propylidene), 96.7 (C-1), 79.8, 76.4, 74.4, 72.2, 71.9, 71.1, 70.6, 69.9 and 67.3 (9C, skeleton carbons), 63.5 (C-7), 33.3 (C-6’), 32.7 (S*C*H_2_), 26.2, 26.0, 25.0 and 24.5 (4C, 4×C*C*H_3_). MS (ESI-TOF) *m*/*z*: [M + Na]^+^ Calcd for C_47_H_48_O_14_NaS 891.2662; Found 891.2657 [M + Na]^+^.

#### 4.2.4. 2,6-Anhydro-3,4,5,7-tetra-*O*-benzoyl-1-deoxy-1-*S*-(1′,2′:5′,6′-di-*O*-isopropylidene-α-d-glucopyranose-3′-yl)-1-thio-d-*glycero*-d-*gulo*-heptitol (**13**)

*Exo*-glucal **10** (59 mg, 0.10 mmol) and thiol **2d** (42 mg, 1.5 mmol) were reacted, according to the general method. The crude product was purified by silica gel chromatography in hexane/acetone 8/2, and then in CH_2_Cl_2_/acetone 95/5 to give compound **13** (69 mg).

Yield: 80%, colorless syrup. [α]_D_^20^ = +2.1 (*c* 0.5, CHCl_3_). R_f_ 0.67 (CH_2_Cl_2_/acetone 9/1). ^1^H NMR (360 MHz, CDCl_3_): δ 8.05–7.79 (m, 8H, arom), 7.55–7.23 (m, 12H, arom), 5.92 (t, 1H, *J* = 9.6 Hz), 5.78 (d, 1H, *J* = 3.4 Hz,H-1), 5.69 (t, 1H, *J* = 9.7 Hz), 5.61 (t, 1H, *J* = 9.6 Hz), 4.72 (d, 1H, *J* = 3.5 Hz), 4.63 (dd, 1H, *J* = 3.1 Hz, *J* = 12.2 Hz), 4.51 (dd, 1H, *J* = 5.1 Hz, *J* = 12.1 Hz), 4.39–4.34 (m, 1H), 4.19–4.12 (m, 2H), 4.11–4.03 (m, 2H), 3.97 (dd, 1H, *J* = 5.1 Hz, *J* = 8.6 Hz), 3.55 (d, 1H, *J*= 3.6 Hz), 3.04–2.92 (m, 2H, SC*H*_2_), 1.41 (s, 3H, CH_3_), 1.40 (s, 3H, CH_3_), 1.35 (s, 3H, CH_3_), 1.22 (s, 3H, CH_3_); ^13^C NMR (90 MHz, CDCl_3_): δ = 166.0, 165.8, 165.3 and 165.1 (4C, 4×*C*OPh), 133.5–128.2 (24C, arom), 111.8 and 109.4 (2C, 2×C_q_, *i*-propylidene), 104.7 (C-1), 86.0, 80.1, 78.7, 76.1, 74.2, 73.9, 71.8 and 69.7 (8C, skeleton carbons), 67.6 (C-7), 63.3 (C-6′), 53.2 (C-3′), 33.8 (S*C*H_2_), 26.8, 26.5, 26.1 and 25.2 (4C, 4×C*C*H_3_); MS (ESI-TOF) *m*/*z*: [M + Na]^+^ Calcd for C_47_H_48_O_14_NaS 891.2662; Found 891.2657 [M + Na]^+^.

#### 4.2.5. Methyl-6-*O*-*tert*-butyldiphenylsilyl-2,3-(2′,3′-dimethoxybutane-2′3′-diyl)-2-deoxy-2-C-methylene-α-d-*arabino*-hexopyranoside (**16**)

Compound **14** (1.989 g, 3.638 mmol) was dissolved in abs. CH_2_Cl_2_ (20 mL). Dess-Martin periodinane (1.855 g, 4.366 mmol, 1.2 equiv.) was added and the reaction was stirred for one hour. When TLC showed complete disappearance of the starting material, the reaction mixture was diluted with CH_2_Cl_2_, aq NaOH solution (28 mL, 1.3 M) was added and the mixture was vigorously stirred for 10 min. In the next step the organic layer was separated and washed with water, dried over MgSO_4_ and concentrated in vacuo to yield **15** (1.965 g, 99%). This compound was used in the next step without further purification. Dry tetrahydrofurane (20 mL) was stirred under argon and methyltriphenylphosphonium bromide (2.062 g, 5.772 mmol, 1.6 equiv.) was added. The suspension was cooled to 0 °C and *n*-butyllithium in hexane (2.309 mL, 5.772 mmol, c = 2.5 M, 1.6 equiv.) was added dropwise. After stirring the mixture for 30 min., **15** (1.965 g, 3.067 mmol) dissolved in dry tetrahydrofurane (10 mL) was added dropwise. The reaction was monitored by TLC. After three hours, ethyl acetate (200 mL) was added, and the organic layer was washed three times with satd aq NH_4_Cl solution and water, dried over MgSO_4_, and evaporated in vacuo. The crude product was purified by column chromatography to give **16** (1.130 g)

Yield: 57% from **14**, colorless syrup. [α]_D_^20^ = +170.3 (*c* 0.1, CHCl_3_). R_f_ 0.72 (hexane/acetone 7/3). ^1^H NMR (400 MHz, CDCl_3_): δ = 7.74–7.70 (m, 4H, arom), 7.42–7.32 (m, 6H, arom), 5.27 (s, 1H, C*H_2a_*), 5.09 (s, 1H, C*H_2b_*), 5.00 (s, 1H, H-1), 4.60 (dt, *J* = 9.9, 2.2 Hz, 1H, H-3), 3.94 (ddd, *J* = 10.1, 6.8, 3.8 Hz, 1H, H-5), 3.90 (d, *J* = 3.3 Hz, 2H, H-6_a_ and H-6_b_), 3.68 (t, *J* = 9.8 Hz, 1H, H-4), 3.36 (s, 3H, OC*H_3_*), 3.27 (s, 3H, OC*H_3_*), 3.17 (s, 3H, OC*H_3_*), 1.36 (s, 3H, C*H_3_*, butanedione), 1.29 (s, 3H, C*H_3_* butanedione), 1.03 (s, 9H,3×CH_3_, *t*-Bu); ^13^C NMR (101 MHz, CDCl_3_): δ = 141.4 (C-2), 136.1, 135.7, 134.1, 133.6, 129.6, 127.7, 127.6 (arom), 109.0 (*C*H_2_), 102.2 (C-1), 100.1, 99.8 (2×C_q_, butanedione), 71.5, 69.2 and 68.3 (C-5, C-4 and C-3), 62.4 (C-6), 54.3 (O*C*H_3_), 48.2, 48.2 (2×O*C*H_3_, butanedione), 26.9 (3×*C*H_3_, *t*-Bu), 19.5 (*C*_q_, *t*-Bu), 18.0, 17.9 (2×*C*H_3_, butanedione). MS (ESI-TOF) *m*/*z*: [M + Na]^+^ Calcd for C_30_H_42_O_7_NaSi 565.2597; Found 565.2592 [M + Na]^+^.

#### 4.2.6. Methyl 6-*O*-*tert*-butyldiphenylsilyl-2-deoxy-2-C-(2′,3′,4′,6′-tetra-*O*-acetyl-1′-thiomethyl-β-d-glucopyranosyl)-2,3-(2″,3″-dimethoxybutane-2″3″-diyl)-α-d-glucopyranoside (**17a**) and Methyl 6-*O*-*tert*-butyldiphenylsilyl-2-deoxy-2-*C*-(2′,3′,4′,6′-tetra-*O*-acetyl-1′-thiomethyl-β-d-glucopyranosyl)-2,3-(2″,3″-dimethoxybutane-2″3″-diyl)-α-d-mannopyranoside (**17b**)

Compound **16** (535 mg, 986 mmol) and **2a** (431 mg, 1.183 mmol, 1.2 equiv.) were reacted, according to the general method*,* to give an inseparable 1:1 mixture of **17a** and **17b** (813 mg). The diastereoisomeric ratio was determined on the basis of ^1^H NMR spectrum. 

Yield: 91%. R_f_ 0.40 (hexane/acetone 7/3). MS (ESI-TOF) *m*/*z*: [M + Na]^+^ Calcd for C_44_H_62_O_16_NaSSi 929.3426; Found 929.3421 [M + Na]^+^.

#### 4.2.7. Methyl-6-*O*-*tert*-butyldiphenylsilyl-2-deoxy-2-C-(2′,3′,4′,6′-tetra-*O*-acetyl-1′-thiomethyl-β-d-glucopyranosyl)-α-d-mannopyranoside (**18**) and Methyl-6-*O*-*tert*-butyldiphenylsilyl-2-deoxy-2-*C*-(2′,3′,4′,6′-tetra-*O*-acetyl-1′-thiomethyl-β-d-glucopyranosyl)-α-d-glucopyranoside (**19**)

The mixture of **17a** and **17b** (295 mg, 0.325 mmol) was dissolved in CH_2_Cl_2_ (5 mL) and 2 mL 90 *v*/*v*% trifluoroacetic acid (1.8 mL trifluoroacetic acid + 0.2 mL water) was added dropwise. After 15 min. toluene (5 mL) was added and the mixture was evaporated in vacuo. The crude product was purified by flash chromatography to give **18** (45 mg), **19** (64 mg), and a mixture of **18** and **19** (94 mg).

**18**: Yield: 17%, colorless syrup. [α]_D_^20^ = +5.0 (*c* 0.06 CHCl_3_). R_f_ 0.50 (CH_2_Cl_2_/MeOH 95/5). ^1^H NMR (400 MHz, CDCl_3_): δ = 7.73–7.37 (m, 10H, arom), 5.21 (t, *J* = 9.3 Hz, 1H, H-3′), 5.14–5.08 (m, 1H, H-4′), 5.06 (t, *J* = 8.3 Hz, 1H, H-2’), 4.75 (s, 1H, H-1), 4.48 (d, *J* = 10.0 Hz, 1H, H-1’), 4.27 (dd, *J* = 12.4, 4.4 Hz, 1H, H-6’_a_), 4.15–4.10 (m, 1H, H-6′_b_), 4.04 (dd, *J* = 9.0, 5.4 Hz, 1H, H-3), 3.88 (s, 1H, H-6_a_), 3.87 (s, 1H, H-6_b_), 3.72–3.67 (m, 1H, H-5′), 3.62 (t, *J* = 9.3 Hz, 1H, H-4), 3.57–3.52 (m, 1H, H-5), 3.28 (s, 3H, OC*H*_3_), 3.23 (dd, *J* = 13.9, 2.1 Hz, 1H, SC*H*_2a_), 2.44 (dd, *J* = 13.6, 11.1 Hz, 1H, SC*H*_2b_), 2.32–2.25 (m, 1H, H-2), 2.07 (s, 3H, COC*H*_3_), 2.05 (s, 3H, COC*H*_3_), 2.02 (s, 3H, COC*H*_3_), 2.01 (s, 3H, COC*H*_3_), 1.07 (s, 9H, 3×C*H*_3_, *t*-Bu), ^13^C NMR (101 MHz, CDCl_3_): δ = 170.9, 170.3, 169.5 and 169.5 (4×*C*OCH_3_), 135.7, 135.7, 133.0, 132.9, 130.1, 130.1, 128.0 and 128.0 (10C, arom), 100.3 (C-1), 83.8 (C-1′), 76.0, 74.0, 70.9, 70.7, 70.5, 69.9 and 68.3 (7C, skeleton carbons), 65.1 (C-6), 62.0 (C-6’), 55.0 (O*C*H_3_), 46.2 (C-2), 27.0 (3C, 3×*C*H_3_, *t*-Bu), 25.9 (S*C*H_2_), 20.8, 20.8, 20.7 and 20.7 (4C, 4×CO*C*H_3_), 19.3 (C_q_, *t*-Bu); MS (ESI-TOF) *m*/*z*: [M + Na]^+^ Calcd for C_38_H_52_O_14_SSiNa 815.274; Found 815.276.

**19**: Yield: 25%, colorless syrup. [α]_D_^20^ = +28.6 (*c* = 0.07 CHCl_3_). R_f_ 0.56 (CH_2_Cl_2_/MeOH 95/5). ^1^H NMR (400 MHz, CDCl_3_): δ = 7.73–7.34 (m, 10H, arom), 5.21 (t, *J* = 9.3 Hz, 1H, H-3’), 5.09–4.98 (m, 2H, H-2′, H-4′), 4.80 (d, *J* = 3.3 Hz, 1H, H-1), 4.52 (d, *J* = 10.1 Hz, 1H, H-1′), 4.21 (dd, *J* = 12.4, 4.9 Hz, 1H, H-6′_a_), 4.15 (dd, *J* = 12.3, 2.3 Hz, 1H, H-6′_b_), 3.88 (s, 1H, H-6_a_), 3.87 (s, 1H, H-6_b_), 3.74–3.58 (m, 3H, H-3, H-5 and H-5′), 3.48 (t, *J* = 9.1 Hz, 1H, H-4), 3.28 (s, 3H, OC*H*_3_), 3.13 (s, 1H, OH), 3.07 (dd, *J* = 13.6, 4.0 Hz, 1H, SC*H*_2a_), 2.84 (s, 1H, OH), 2.74 (dd, *J* = 13.5, 10.0 Hz, 1H, SC*H*_2b_), 2.06 (s, 3H, COC*H*_3_), 2.06 (s, 3H, COC*H*_3_), 2.02 (s, 3H, COC*H*_3_), 2.00 (s, 3H, COC*H*_3_), 1.98–1.92 (m, 1H, H-2), 1.06 (s, 9H, 3×C*H*_3_, *t*-Bu), ^13^C NMR (101 MHz, CDCl_3_): δ = 170.8, 170.3, 169.6 and 169.5 (4×*C*OCH_3_), 135.7, 133.0, 132.9, 130.0, 130.0 and 127.9 (10C, arom), 99.4 (C-1), 85.0 (C-1’), 77.5, 77.2, 76.8, 76.0, 74.5, 73.9, 72.9, 70.2 and 68.4 (7C, skeleton carbons), 65.5 (C-6), 62.2 (C-6’), 54.9 (O*C*H_3_), 46.3 (C-2), 29.2 (S*C*H_2_), 26.9 (3C, 3×*C*H_3_, *t*-Bu), 20.9, 20.8, 20.7 and 20.7 (4C, 4×CO*C*H_3_), 19.3 (C_q_, *t*-Bu). Anal. Calcd for C_38_H_52_O_14_SSi: C, 55.96; H, 6.61; O, 28.25; S, 4.04; Si, 3.54. Found: C, 6.60, H, 6.60; S, 4.11.

Mixture of **18** and **19**: Yield: 36%, colorless syrup.

#### 4.2.8. Methyl-6-*O*-*tert*-butyldimethylsilyl-3,4-di-*O*-isopropylidene-α-d-*lyxo*-hexopyranoside-2-ulose (**21**)

A mixture of dimethyl sulfoxide (163 μL, 179 mg, 2.30 mmol, 4 equiv.) and abs. CH_2_Cl_2_ (2 mL) was cooled to −80 °C and oxalyl chloride (97 μL, 144 mg, 1.15 mmol, 2 equiv.) was added. After 15 min., compound **20** (200 mg, 0.57 mmol) dissolved in CH_2_Cl_2_ (1 mL) was added. After 30 min., *N*,*N*-diisopropylethylamine (1.000 mL, 0.742 g, 5.741 mmol, 10 equiv.) was added and the mixture was then allowed to warm to room temperature. After 2 h, when TLC showed complete conversion, the mixture was diluted with CH_2_Cl_2_, extracted with 1 M HCl and water. The organic layer was dried over MgSO_4_, filtered and concentrated in vacuo. The crude product was purified by column chromatography (hexane/ethyl acetate 7/3) to give **21** (189 mg).

Yield: 95%, colorless syrup. [α]_D_^20^ = +58.6 (*c* 0.1, CHCl_3_). R_f_ 0.52 (hexane/ethyl acetate 7/3). ^1^H NMR (400 MHz, CDCl_3_): δ = 4.61 (s, 1H, H-1), 4.57 (d, *J* = 5.5 Hz, 1H, H-3), 4.48 (dd, *J* = 5.6, 1.9 Hz, 1H, H-4), 4.26 (td, *J* = 6.6, 1.9 Hz, 1H, H-5), 3.83 (dd, *J* = 10.0, 6.8 Hz, 1H, H-6_a_), 3.77 (dd, *J* = 10.0, 6.5 Hz, 1H, H-6_b_), 3.40 (s, 3H, OC*H*_3_), 1.35, 1.29 (2s, 6H, 2×*i*-Pr C*H*_3_), 0.84 (s, 9H, 3×*t*-Bu C*H*_3_), 0.03 (s, 6H, 2×Si-C*H*_3_); ^13^C NMR (101 MHz, CDCl_3_): δ = 199.2 (*C*O), 110.6 (*i*-Pr *C*_q_), 100.5 (C-1), 77.1, 75.5, 68.2 (C-3, C-4, C-5), 61.9 (C-6), 55.3 (OC*H*_3_), 27.2, 26.1 (2C, 2×*i*-Pr *C*H_3_), 25.8 (3C, 3×*t*-Bu C*H*_3_), 18.2 (C-2), -5.4, -5.5 (2C, Si-*C*H_3_).

#### 4.2.9. Methyl 6-*O*-*tert*-butyldimethylsilyl-2-deoxy-3,4-*O*-isopropylidene-2-*C*-methylene-α-d-*lyxo*-hexopyranoside (**22**)

Methyltriphenylphosphonium bromide (332 mg, 0.929 mmol, 1.6 equiv.) was suspended in tetrahydrofurane (1.5 mL) under argon and cooled to 0 °C. *n*-Butyllithium (372 μL, 0.929 mmol, 1.6 equiv, 2.5 M solution in hexane) was added and the suspension was stirred. After 30 min. **21** (200 mg, 0.581 mmol) dissolved in tetrahydrofurane (1.5 mL) was added. When TLC showed the complete disappearance of **21**, the mixture was diluted with ethyl acetate and washed with satd aq NH_4_Cl solution. The organic layer was separated, dried over MgSO_4_, and concentrated in vacuo. The crude product was purified by column chromatography (hexane/ethyl acetate 9/1) to give **22** (125 mg)

Yield: 63%, colorless syrup. [α]_D_^20^ = +71.8 (*c* 0.4, CHCl_3_). R_f_ 0.48 (hexane/ethyl acetate 9/1). ^1^H NMR (400 MHz, CDCl_3_): δ = 5.52–5.48 (m, 2H, C*H*_2_), 5.23 (s, 1H, H-1), 4.83 (d, *J* = 6.9 Hz, 1H, H-3), 4.34 (dd, *J* = 6.9, 1.5 Hz, 1H, H-4), 3.92–3.80 (m, 3H, H-5, H-6_a_, H-6_b_), 3.52 (s, 3H, OC*H*_3_), 1.55, 1.42 (2s, 6H, 2 x *i*-Pr C*H*_3_), 0.97 (s, 9H, 3×*t*-Bu C*H*_3_), 0.15 (s, 6H, 2 x Si-C*H*_3_); ^13^C NMR (101 MHz, CDCl_3_): δ = 141.0 (C-2), 118.4 (*C*H_2_), 110.0 (*i*-Pr *C*_q_), 99.4 (C-1), 75.5, 74.8, 70.4 (C-3, C-4, C-5), 62.3 (C-6), 54.8 (OC*H*_3_), 26.9, 26.0 (2C, 2×*i*-Pr *C*H_3_), 25.7 (3C, 3×*t*-Bu C*H*_3_), 18.4 (C-2), -5.2, -5.4 (2C, Si-*C*H_3_); MS (ESI-TOF) *m*/*z*: [M + Na]^+^ Calcd for C_17_H_32_O_5_NaSi 367.1911; Found 367.1917.

#### 4.2.10. Methyl 6-*O*-*tert*-butyldimethylsilyl-2-deoxy-3,4-*O*-isopropylidene-2-*C*-(2′,3′,4′,6′-tetra-*O*-acetyl-1′-thiomethyl-β-d-glucopyranosyl)-α-d-galactopyranoside (**23a**) and Methyl 6-*O*-*tert*-butyldimethylsilyl-2-deoxy-3,4-*O*-isopropylidene-2-*C*-(2′,3′,4′,6′-tetra-*O*-acetyl-1′-thiomethyl-β-D-glucopyranosyl)-α-d-talopyranoside (**23b**)

Exomethylene **22** (100 mg, 0.290 mmol), thiol **2a** (127 mg, 0.348 mmol, 1.2 equiv.), and DPAP (7 mg, 0.29 mmol) were reacted with three irradiation cycles according to the general method. The crude product was purified by column chromatography (hexane/ethyl acetate 75/25) to give **23** (134 mg, 65%) as an inseparable diastereoisomeric mixture with a ratio of **23a *galacto***:**23b *talo*** ~ 1:2. The reaction was also carried out at −80 °C to give **23** (182 mg, 88%), with a ratio of **23a *galacto***:**23b *talo*** ~ 1:1. The diastereoisomeric ratios were determined on the basis of ^1^H NMR spectrum. 

Yield (mixture of **23a** and **23b**): 88%, white foam. R_f_ 0.39 (hexane/ethyl acetate 75/25). ^1^H NMR (400 MHz, CDCl_3_): δ = 5.21 (t, *J* = 9.3 Hz, 1.59H), 5.11–4.94 (m, 2.98H), 4.89 (d, *J* = 3.2 Hz, 0.58H), 4.67 (dd, *J* = 7.9, 2.5 Hz, 1.05H), 4.63 (d, *J* = 10.1 Hz, 0.97H), 4.48 (d, *J* = 10.1 Hz, 0.60H), 4.42 (d, *J* = 7.4 Hz, 1.02H), 4.27–4.15 (m, 3.71H), 4.13–4.06 (m, 1.02H), 4.00–3.82 (m, 1.58H), 3.84–3.61 (m, 4.32H), 3.37 (s, 3.00H, OC*H*_3_), 3.34 (s, 1.70H, OC*H*_3_), 3.06–2.82 (m, 2.70H), 2.68–2.59 (m, 0.63H, COC*H*_3_), 2.14–1.97 (m, 17.42H, COC*H*_3_), 1.48, 1.43, 1.32 (3s, 11.03H, *i*-Pr C*H*_3_), 0.90, 0.89 (2s, 13.81H, *t*-Bu C*H*_3_), 0.11–0.03 (m, 7.81H, Si-C*H*_3_); ^13^C NMR (101 MHz, CDCl_3_): δ = 170.8, 170.7, 170.2, 170.2, 169.5, 169.4, 169.3, 169.3 (*C*O), 109.2, 109.0 (2×*i*-Pr *C*_q_), 101.0, 98.7 (2×C-1), 85.7, 85.4 (2×C-1′), 76.0, 75.9, 74.9, 73.9, 73.6, 71.6, 71.5, 70.8, 70.5, 70.2, 68.4 67.9 (skeleton carbons), 62.7, 62.4, 62.2, 62.2 (4×C-6), 55.1, 54.9 (2×OC*H*_3_), 43.4, 41.1, 31.3, 31.0 (2×S-*C*H_2_), 28.6, 26.6, 26.2 (*i*-Pr *C*H_3_), 25.9, 25.9, 25.1 (*t*-Bu C*H*_3_), 20.8, 20.6 (CO*C*H_3_), 18.3, 17.1 (2×C-2), -5.3, -5.4 (2C, Si-*C*H_3_). 

#### 4.2.11. Methyl 6-*O*-*tert*-butyldimethylsilyl-2-deoxy-3,4-*O*-isopropylidene-2-*C*-(2′,3′,4′,6′-tetra-*O*-acetyl-1′-thiomethyl-α-d-mannopyranosyl)-α-d-galactopyranoside (**24a**) and Methyl 6-*O*-*tert*-butyldimethylsilyl-2-deoxy-3,4-*O*-isopropylidene-2-*C*-(2′,3′,4′,6′-tetra-*O*-acetyl-1′-thiomethyl-α-d-mannopyranosyl)-α-d-talopyranoside (**24b**)

Exomethylene **22** (100 mg, 0.290 mmol), thiol **2e** (127 mg, 0.348 mmol, 1.2 equiv.) and 2,2-dimethoxy-2-phenylacetophenone (7 mg, 0.029 mmol, 0.1 equiv.) were dissolved in toluene (2.3 mL) and then reacted according to the general method. The reaction mixture was concentrated in vacuo, the crude product was purified by flash chromatography (hexane/acetone 9/1 → 85/15) to give an inseparable mixture of **24a *galacto*** and **24b *talo*** (123 mg, 60%) as a colorless syrup. R_f_ 0.42 (hexane/acetone 75/25) in a 1:4 ratio of **24a *galacto*** and **24b *talo***. The reaction was also carried out at −80 °C to give **37** (197 mg, 96%) with a ratio of **24a *galacto***:**24b *talo*** ~ 1:1. The diastereoisomeric ratios were determined on the basis of ^1^H NMR spectrum (see Supplementary Materials).

Yield (Mixture of **24a** and **24b**): 96%, white foam. R_f_ 0.42 (hexane/acetone 75/25).

#### 4.2.12. Methyl 2-deoxy-2-*C*-(2′,3′,4′,6′-tetra-*O*-acetyl-1′-thiomethyl-α-d-mannopyranosyl)-α-d-galactopyranoside (**25**) and Methyl 2-deoxy-2-*C*-(2′,3′,4′,6′-tetra-*O*-acetyl-1′-thiomethyl-α-d-mannopyranosyl)-α-d-talopyranoside (**26**)

Compound **24** (161 mg, 0.227 mmol, 1:1 mixture of **24a *galacto*** and **24b *talo***) was dissolved in CH_2_Cl_2_ (2 mL) and 500 μL 70 *v*/*v*% trifluoroacetic acid (350 μL trifluoroacetic acid + 150 μL water) was added dropwise. After 15 min. toluene (5 mL) was added and the mixture was evaporated in vacuo. The crude product was purified by flash chromatography to give **25** (37 mg, 29%) and a mixture of **25** and **26** (80 mg, 64%).

**25** Yield: 29%, colorless syrup. [α]_D_^20^ = +91.8 (*c* 0.15, CHCl_3_), ^1^H NMR (400 MHz, CDCl_3_): δ = 5.35 (dd, *J* = 3.0, 1.4 Hz, 1H, H-2′), 5.32–5.27 (m, 2H, H-4′, H-1′), 5.24 (dd, *J* = 9.9, 3.1 Hz, 1H, H-3′), 4.91 (s, 1H, H-1), 4.34 (ddd, *J* = 9.2, 5.2, 2.3 Hz, 1H, H-5′), 4.28 (dd, *J* = 12.1, 5.3 Hz, 1H, H-6′_a_), 4.15 (dd, *J* = 12.0, 2.2 Hz, 1H, H-6′_b_), 4.06 (dd, *J* = 4.9, 3.8 Hz, 1H, **H-3**), 3.97–3.92 (m, 2H, H-4, H-6_a_), 3.88 (dd, *J* = 11.7, 4.3 Hz, 1H, H-6_b_), 3.77 (td, *J* = 4.6, 1.8 Hz, 1H, H-5), 3.46 (s, 1H, OH), 3.37 (s, 3H, OMe), 3.15 (dd, *J* = 13.7, 3.3 Hz, 1H, SCH_2a_), 2.93 (dd, *J* = 13.6, 11.1 Hz, 1H, SCH_2b_), 2.20–2.14 (m, 4H, COC*H_3_* and H-2), 2.11 (s, 3H, COC*H_3_*), 2.06 (s, 3H, COC*H_3_*), 2.00 (s, 3H, COC*H_3_*); ^13^C NMR (101 MHz, CDCl_3_): δ = 171.1, 170.2, 170.1 and 169.9 (4C, 4×*C*OCH_3_), 100.8 (C-1), 84.5 (C-1′), 71.2, 70.1, 69.5, 69.4, 69.2, 66.9 and 66.5 (7C, skeleton carbons), 63.6 (C-6), 62.6 (C-6’), 55.2 (O*C*H_3_), 44.3 (C-2), 30.3 (S*C*H_2_), 21.0, 20.8, 20.8 and 20.7 (4C, 4×CO*C*H_3_). MS (ESI-TOF) *m*/*z*: [M + Na]^+^ Calcd for C_22_H_34_O_14_NaS 577.1567; Found 557.1560; [M + Na]^+^.

The NMR spectra of the mixture of **25** and **26** can be found in the Supplementary Material.

#### 4.2.13. Methyl-6-*O*-*tert*-butyldiphenylsilyl-3,4-*O*-isopropylidene-β-d-*lyxo*-hexopyranoside-2-ulose (**28**)

Dimethyl sulfoxide (1.135 mL, 1.248 g, 15.978 mmol, 4 equiv.) was dissolved in abs. CH_2_Cl_2_ (19 mL), cooled to −80 °C and oxalyl chloride (676 μL, 1.014 g, 7.989 mmol, 2 equiv.) was added. After 15 min. **27** (1.888 g, 3.994 mmol) dissolved in CH_2_Cl_2_ (19 mL) was added. After 30 min, *N*,*N*-diisopropylethylamine (2.783 mL, 2.065 g, 15.978 mmol, 4 equiv.) was added and the mixture was then allowed to warm to room temperature. After 2 h, when TLC showed complete conversion, the mixture was diluted with CH_2_Cl_2_, extracted with 1 M HCl and water. The organic layer was dried over MgSO_4_, filtered, and concentrated in vacuo. The crude product was purified by column chromatography (hexane/acetone 75/25) to give **28** (1.798 g).

Yield: 96%, colorless syrup. [α]_D_^20^= −13.1 (*c* 0.2, CHCl_3_). R_f_ 0.27 (hexane/acetone 8/2). ^1^H NMR (400 MHz, CDCl_3_): δ = 7.74–7.68 (m, 4H, arom.), 7.46–7.36 (m, 6H, arom.), 4.75 (d, *J* = 0.5 Hz, 1H, H-1), 4.68 (dd, *J* = 5.6, 1.7 Hz, 1H, H-4), 4.50 (d, *J* = 5.6 Hz, 1H, H-3), 4.15 (td, *J* = 6.6, 1.6 Hz, 1H, H-5), 4.02–3.97 (m, 2H, H-6_a_, H-6_b_), 3.57 (s, 3H, OC*H_3_*), 1.42 (s, 3H, C*H_3_*, *i*-Pr), 1.38 (s, 3H, C*H_3_*, *i*-Pr), 1.08 (s, 9H, 3×C*H_3_*, *t*-Bu); ^13^C NMR (101 MHz, CDCl_3_): δ = 198.7 (C-2), 135.7, 135.6, 133.3, 133.2, 129.9, 127.9 and 127.8 (12C, arom.), 111.2 (C_q_, *i*-Pr), 100.4 (C-1), 77.8, 77.7 and 73.3 (C-3, C-4 and C-5), 62.5 (C-6), 56.9 (O*C*H_3_), 27.3 (*C*H_3_, *i*-Pr), 26.8(3C, 3×*C*H_3_, *t*-Bu), 26.1 (*C*H_3_, *i*-Pr), 19.3 (C_q_, *t*-Bu). Anal. Calcd for C_26_H_34_O_6_Si: C, 66.36; H, 7.28. Found: C, 66.71; H, 7.07.

#### 4.2.14. Methyl 6-*O*-*tert*-butyldiphenylsilyl-2-deoxy-3,4-*O*-isopropylidene-2-*C*-methylene-β-d-*lyxo*-hexopyranoside (**29**)

Methyltriphenylphosphonium bromide (2.119 g, 5.932 mmol, 1.6 equiv.) was suspended in tetrahydrofurane (20 mL) under argon and cooled to 0 °C. *n*-Butyllithium (2.373 mL, 5.932 mmol, 1.6 equiv, 2.5 M solution in hexane) was added and the suspension was stirred. After 30 min. **28** (1.745 g, 3.708 mmol) dissolved in tetrahydrofurane (17 mL) was added. When TLC showed complete disappearance of **28** the mixture was diluted with ethyl acetate and washed with satd aq NH_4_Cl solution. The organic layer was separated, dried over MgSO_4_, and then concentrated in vacuo. The crude product was purified by column chromatography to give **29** (1.153 g).

Yield: 66%, colorless syrup. [α]_D_^20^= +16.7 (c= 0.18, CHCl_3_). R_f_ 0.68 (hexane/acetone 8/2). ^1^H NMR (400 MHz, CDCl_3_): δ = 7.74–7.68 (m, 4H), 7.44–7.34 (m, 6H), 5.47–5.45 (m, 1H, C*H_2a_*), 5.44–5.42 (m, 1H, C*H_2b_*), 4.84 (s, 1H, H-1), 4.69 (dt, *J* = 6.3, 1.4 Hz, 1H, H-3), 4.30 (dd, *J* = 6.3, 1.9 Hz, 1H, H-4), 3.96 (dd, *J* = 10.0, 6.9 Hz, 1H, H-6_a_), 3.90 (dd, *J* = 10.1, 6.3 Hz, 1H, H-6_b_), 3.74–3.70 (m, 1H, H-5), 3.50 (s, 3H, OC*H_3_*), 1.46 (s, 3H, C*H_3_*, *i*-Pr), 1.36 (s, 3H, C*H_3_*, *i*-Pr), 1.06 (s, 9H, 3×C*H_3_*, *t*-Bu); ^13^C NMR (101 MHz, CDCl_3_): δ = 141.7 (C-2), 135.8, 135.7, 133.7, 133.6, 129.8, 127.8 and 127.7 (12C arom.), 115.8 (C-2), 110.3 (C_q_, *i*-Pr), 101.0 (C-1), 75.3, 73.7 and 73.3 (C-5, C-4, C-3), 63.0 (C-6), 56.2 (O*C*H_3_), 27.4 (*C*H_3_, *i*-Pr), 26.9 (3C, 3×*C*H_3_, *t*-Bu), 26.2 (*C*H_3_, *i*-Pr), 19.4 (C_q_, *t*-Bu); MS (MALDI-TOF) *m*/*z*: [M + Na]^+^ Calcd for C_27_H_36_O_5_NaSi 491.223; Found: 491.290.

#### 4.2.15. Methyl 6-*O*-*tert*-butyldiphenylsilyl-2-deoxy-3,4-*O*-isopropylidene-2-*C*-(2′,3′,4′,6′-tetra-*O*-acetyl-1′-thiomethyl-β-d-glucopyranosyl)-β-d-galactopyranoside (**30a**) and Methyl 6-*O*-*tert*-butyldiphenylsilyl-2-deoxy-3,4-*O*-isopropylidene-2-*C*-(2′,3′,4′,6′-tetra-*O*-acetyl-1′-thiomethyl-β-d-glucopyranosyl)-β-d-talopyranoside (**30b**)

Compound **29** (100 mg, 0.213 mmol), **2a** (93 mg, 0.256 mmol, 1.2 equiv.) and 2,2-dimethoxy-2-phenylacetophenone (5 mg, 0.021 mmol, 0.1 equiv.) were dissolved in toluene (1.9 mL) and reacted according to the general method with three irradiation cycles. The crude product was purified by column chromatography (hexane/acetone 8/2 → 75/25) to give **30** (39 mg, 22%) in 4:1 ratio of the **d*-galacto*** and **d*-talo*** isomers (the diastereoisomeric ratio was determined on the basis of ^1^H NMR spectrum). The reaction was also carried out at −80 °C to exclusively give the **d*-galacto*** isomer **30a** (135 mg). 

**30a *galacto*** Yield: 76%, colorless syrup. [α]_D_^20^= −37.7 (*c* 0.2, CHCl_3_). R_f_ 0.45 (hexane/acetone 7/3). ^1^H NMR (400 MHz, CDCl_3_): δ = 7.74–7.67 (m, 4H, arom.), 7.46–7.34 (m, 6H, arom.), 5.21 (t, *J* = 9.4 Hz, 1H, H-3’), 5.12–5.02 (m, 2H, H-4’, H-2’), 4.49 (d, *J* = 10.0 Hz, 1H, H-1’), 4.27–4.21 (m, 2H) and 4.18–4.12 (m, 3H) (H-6’_a_, H-6’_b_, H-4, H-3, H-1), 3.99 (dd, *J* = 9.8, 7.3 Hz, 1H, H-6_a_), 3.92 (dd, *J* = 9.8, 6.1 Hz, 1H, H-6b), 3.85–3.80 (m, 1H, H-5), 3.71 (ddd, *J* = 9.9, 5.0, 2.3 Hz, 1H, H-5’), 3.46 (s, 3H, OC*H_3_*), 3.02 (dd, *J* = 12.7, 5.1 Hz, 1H, SC*H_2a_*), 2.95 (dd, *J* = 12.7, 3.6 Hz, 1H, SC*H_2b_*), 2.07 (s, 3H, COC*H_3_*), 2.07 (s, 3H, COC*H_3_*), 2.03 (s, 3H, COC*H_3_*), 2.01 (s, 3H, COC*H_3_*), 1.98–1.91 (m, 1H, H-2), 1.47 (s, 3H, C*H_3_*, *i*-Pr), 1.33 (s, 3H, C*H_3_*, *i*-Pr), 1.05 (s, 9H, 3×C*H_3_*, *t*-Bu); ^13^C NMR (101 MHz, CDCl_3_): δ = 170.7 (*C*OCH_3_), 170.3 (*C*OCH_3_), 169.5 (*C*OCH_3_), 169.5 (*C*OCH_3_), 135.7, 135.7, 133.6, 133.5, 129.8, 127.8 and 127.7 (12C, arom.), 109.7 (C_q_, *i*-Pr), 102.4 (C-1), 84.2 (C-1’), 76.0, 75.2, 74.0, 73.4, 71.8, 70.3, 68.5, 63.0 (C-6), 62.4 (C-6’), 56.6 (O*C*H_3_), 44.8 (C-2), 28.5 (*C*H_3_, *i*-Pr), 28.4 (S*C*H_2_), 26.8 (3C, 3×*C*H_3_, *t*-Bu), 26.6 (*C*H_3_, *i*-Pr), 20.8 (CO*C*H_3_), 20.8 (CO*C*H_3_), 20.7 (CO*C*H_3_), 20.7 (CO*C*H_3_), 19.3 (C_q_, *t*-Bu); MS (MALDI-TOF) *m*/*z*: [M + Na]^+^ Calcd for C_41_H_56_NaO_14_SSi 855.306; Found: 855.209.

#### 4.2.16. Methyl 6-*O*-*tert*-butyldiphenylsilyl-2-deoxy-3,4-*O*-isopropylidene-2-*C*-(2′,3′,4′,6′-tetra-*O*-acetyl-1′-thiomethyl-α-d-mannopyranosyl)-β-d-galactopyranoside (**31**)

Compound **29** (100 mg, 0.213 mmol), **2e** (93 mg, 0.256 mmol, 1.2 equiv.) and 2,2-dimethoxy-2-phenylacetophenone (5 mg, 0.021 mmol, 0.1 equiv.) were dissolved in toluene (1.9 mL) and reacted according to the general method with three irradiation cycles. The crude product was purified by column chromatography (hexane/acetone 8/2) to give **31** (69 mg, 39%) as a colorless syrup. The reaction was also carried out at −80°C to give **31** (120 mg).

Compound **31**: Yield: 68%, [α]_D_^20^= +84.7 (*c* 0.2, CHCl_3_). R_f_ 0.41 (hexane/acetone 7/3). ^1^H NMR (400 MHz, Acetone-d6): δ = 7.81–7.75 (m, 4H, arom.), 7.51–7.41 (m, 6H, arom), 5.35–5.32 (m, 2H, H-1’, H-2’), 5.29 (t, *J* = 10.0 Hz, 1H, H-4’), 5.21 (dd, *J* = 10.1, 3.0 Hz, 1H, H-3’), 4.40 (ddd, *J* = 8.3, 5.6, 2.2 Hz, 1H, H-5’), 4.33 - 4.24 (m, 4H, H-6’_a_, H-4, H-3, H-1), 4.15 - 4.08 (m, 2H, H-6’_b_, H-5), 4.00 (dd, *J* = 9.8, 6.3 Hz, 1H, H-6_a_), 3.92 (dd, *J* = 9.8, 6.7 Hz, 1H, H-5), 3.44 (s, 3H, OC*H*_3_), 3.01 (dd, *J* = 12.9, 4.8 Hz, 1H, C*H*_2a_), 2.92 (dd, *J* = 12.9, 3.5 Hz, 1H, C*H*_2b_), 2.13 (s, 3H, COC*H*_3_), 2.05 (s, 6H, 2×COC*H*_3_), 1.96 (s, 3H, COC*H*_3_), 1.94–1.87 (m, 1H, H-2), 1.42 (s, 3H, C*H*_3_, *i*-Pr), 1.31 (s, 3H, C*H*_3_, *i*-Pr), 1.06 (s, 9H, C*H*_3_, *t*-Bu); ^13^C NMR (101 MHz, Acetone-d6): δ = 170.7, 170.4, 170.4 and 170.2 (4C, 4×*C*OCH_3_), 136.4, 134.2, 130.7, 128.7 and 128.6 (12C, arom.), 110.0 (C_q_, *i*-Pr), 103.0 (C-1), 83.6 (C-1’), 76.3, 74.2, 72.9, 71.5, 70.3, 70.1, 66.9, 64.2 and 63.0 (C-6’ and C-6), 56.4 (O*C*H_3_), 46.0 (C-2), 29.6 (S*C*H_2_) 28.7 (CH_3_, *i*-Pr), 27.1 (3C, 3×CH_3_, *t*-Bu), 26.7 (CH_3_, *i*-Pr), 20.7, 20.7, 20.6 and 20.5 (4C, 4×CO*C*H_3_), 19.7 (*t*-Bu, C_q_). MS (ESI-TOF) *m*/*z*: [M + Na]^+^ Calcd for C_41_H_56_O_14_SSiNa 855.3058; Found 855.3055; [M + Na]^+^.

#### 4.2.17. 1,2,4,6-Tetra-*O*-acetyl-3-deoxy-3-*C*-methylene-β-d-*ribo*-hexopyranose (**33**)

Compound **32** (1.613 g, 6.293 mmol) was dissolved in 5 mL 90 *v*/*v*% trifluoroacetic acid (4.5 mL trifluoroacetic acid + 0.5 mL water) and the mixture was stirred at room temperature. After 15 min., toluene (12 mL) was added and the mixture was concentrated in vacuo. Acetic anhydride (4.73 mL, 50.1 mmol, 8 equiv.) and sodium acetate (825 mg, 10.020 mmol, 1.6 equiv.) were added to the residue and the suspension was stirred at 70 °C. After 1.5 h the mixture was allowed to cool down, ethyl acetate was added, and the mixture was washed with water, satd aq NaHCO_3_, and water. The organic layer was dried over MgSO_4_, filtered, and evaporated in vacuo. The crude product was purified by column chromatography (hexane/ethyl acetate 7/3) to give **33** (1.755 g).

Yield: 81% over two steps, colorless syrup. [α]_D_^20^= +6.1 (*c* 0.2, CHCl_3_). R_f_ 0.47 (hexane/ethyl acetate 6/4). ^1^H NMR (400 MHz, CDCl_3_): δ = 5.62 (d, *J* = 7.5 Hz, 1H, H-1), 5.42–5.34 (m, 2H, H-2 and H-4), 5.14 (t, *J* = 1.7 Hz, 1H, C*H_2a_*), 5.11 (t, *J* = 1.6 Hz, 1H C*H_2b_*), 4.32 (dd, *J* = 12.3, 4.7 Hz, 1H, H-6_a_), 4.14 (dd, *J* = 12.2, 2.8 Hz, 1H, H-6_b_), 3.78 (ddd, *J* = 9.0, 4.7, 2.8 Hz, 1H, H-5), 2.16 (s, 3H, COC*H_3_*), 2.15 (s, 3H, COC*H_3_*), 2.13 (s, 3H, COC*H_3_*), 2.09 (s, 3H, COC*H_3_*); ^13^C NMR (101 MHz, CDCl_3_): δ = 169.1 and 169.0 (4×*C*OCH_3_), 137.5 (*C*H_2_), 109.8 (C-3), 93.4 (C-1), 75.8, 70.6 and 68.0 (3C, C-5, C-4, C-2), 62.2 (C-6), 21.0 (CO*C*H_3_), 20.8 (CO*C*H_3_), 20.7 (CO*C*H_3_), 20.7 (CO*C*H_3_). Anal. Calcd for C_15_H_20_O_9_: C, 52.32; H, 5.85. Found: C, 57.06; H, 6.01.

#### 4.2.18. 1,2,4,6-Tetra-*O*-acetyl-3-deoxy-3-*C*-(2′,3′,4′,6′-tetra-*O*-acetyl-1’-thiomethyl-β-d-glucopyranosyl)-β-d-glucopyranose (**34**)

Compound **33** (200 mg, 0.581 mmol) and thiol **2a** (254 mg, 697 mmol, 1.2 equiv.) were reacted according to the general method with three irradiation cycles. The crude product was purified by flash chromatograpy to give **34** (332 mg)

Yield: 82%, white foam. [α]_D_^20^= −30.7 (*c* 0.1, CHCl_3_). R_f_ 0.23 (hexane/acetone 7/3). ^1^H NMR (400 MHz, CDCl_3_): δ = 5.67 (d, *J* = 7.9 Hz, 1H, H-1), 5.21 (t, *J* = 9.4 Hz, 1H) and 5.11–4.91 (m, 4H) (H-4, H-4′, H-3′, H-2, H-2′), 4.40 (d, *J* = 10.0 Hz, 1H, H-1′), 4.27 (dd, *J* = 12.4, 4.8 Hz, 1H), 4.18 (dd, *J* = 12.4, 4.9 Hz, 1H) and 4.12–4.04 (m, 2H) (H-6_a_, H-6′_a_, H-6_b_, H-6′_b_), 3.77 (ddd, *J* = 9.5, 4.7, 2.3 Hz, 1H, H-5), 3.72 (ddd, *J* = 10.0, 4.7, 2.1 Hz, 1H, H-5′), 2.86–2.76 (m, 2H, C*H_2_*), 2.38 - 2.30 (m, 1H, H-3), 2.12 (s, 3H), 2.10 (s, 6H), 2.08 (s, 6H), 2.06 (s, 3H), 2.03 (s, 3H) and 2.00 (s, 3H) (8×COC*H_3_*); ^13^C NMR (101 MHz, CDCl_3_): δ = 170.7, 170.5, 170.0, 169.6, 169.5, 169.4, 169.3 and 169.2 (8C, 8×*C*OCH_3_), 93.3 (C-1), 82.5 (C-1′), 76.0 (C-5’), 75.4 (C-5), 73.7, 69.6, 69.2, 68.2 and 67.5 (C-4, C-4′, C-3′, C-2, C-2′), 62.0 (2C, C-6, C-6’), 43.8 (C-3), 26.3 (*C*H_2_), 20.9, 20.8, 20.7, 20.7 and 20.6 (8C, 8×CO*C*H_3_). Anal. Calcd for C_29_H_40_O_18_S: C, 49.14; H, 5.69; S, 4.51. Found: C, 48.93; H, 5.65; S, 4.62.

#### 4.2.19. 1,2,4,6-Tetra-*O*-acetyl-3-deoxy-3-*C*-(2′,3′,4′,6′-tetra-*O*-acetyl-1′-thiomethyl-α-d-mannopyranosyl)-β-d-glucopyranose (**35**)

Compound **33** (100 mg, 0.290 mmol) and thiol **2e** (127 mg, 0.349 mmol, 1.2 equiv.) dissolved in toluene (2.3 mL) were reacted according to the general method with three irradiation cycles. The reaction mixture was concentrated in vacuo and the crude product was purified with column chromatography (hexane/acetone 65/35) to give **35** (162 mg).

Yield: 80%, colorless syrup. [α]_D_^20^= +77.8 (*c* 0.3, CHCl_3_). R_f_ 0.33 (hexane/acetone 65/35). ^1^H NMR (400 MHz, Acetone-d6): δ = 5.73 (d, *J* = 8.0 Hz, 1H, H-1), 5.31 (d, *J* = 1.4 Hz, 1H, H-1′), 5.29 (dd, *J* = 3.3, 1.4 Hz, 1H, H-2′), 5.29–5.24 (m, 1H, H-4′), 5.19 (dd, *J* = 10.1, 3.4 Hz, 1H, H-3′), 5.03 (t, *J* = 9.3 Hz, 1H, H-4), 4.98 (dd, *J* = 10.1, 7.2 Hz, 1H, H-2), 4.29 (ddd, *J* = 9.5, 5.5, 2.3 Hz, 1H, H-5’), 4.25 - 4.16 (m, 2H, H-6′_a_, H-6’_b_), 4.11 (dd, *J* = 12.0, 2.3 Hz, 1H, H-6_a_), 4.03 (dd, *J* = 12.3, 2.5 Hz, 1H, H-6_b_), 3.97 (ddd, *J* = 9.6, 5.0, 2.5 Hz, 1H, H-5), 2.84 (dd, *J* = 13.9, 3.8 Hz, 1H, SC*H*_2a_), 2.75 (dd, *J* = 13.9, 5.5 Hz, 1H, SC*H*_2b_), 2.53 (tdd, *J* = 10.8, 5.4, 3.9 Hz, 1H, H-3), 2.12 (s, 3H, COC*H_3_*), 2.11 (s, 3H, COC*H_3_*), 2.07 (s, 3H, COC*H_3_*), 2.05 (s, 3H, COC*H_3_*), 2.05 (s, 3H, COC*H_3_*), 2.04 (s, 3H, COC*H_3_*), 2.00 (s, 3H, COC*H_3_*), 1.94 (s, 3H, COC*H_3_*); ^13^C NMR (101 MHz, Acetone-d6): δ = 170.8, 170.8, 170.4, 170.3, 170.2 and 169.6 (8C, 8×*C*OCH_3_), 93.9 (C-1), 82.6 (C-1′), 75.8, 71.2, 70.7, 70.3, 70.2, 69.3 and 66.8 (7C, skeleton carbons), 63.1 and 62.9 (2C, C-6 and C-6′), 45.0 (C-3), 29.2 (S*C*H_2_), 21.1, 20.8, 20.7, 20.7 and 20.6 (8C, 8×CO*C*H_3_); MALDI-TOF *m*/*z*: [M + Na]^+^ Calcd for C_29_H_40_O_18_SNa 731.183; Found 731.135. 

#### 4.2.20. Methyl-6-*O*-*tert*-butyldiphenylsilyl-2,3-(2′,3′-dimethoxybutane-2′3′-diyl)-4-deoxy-4-C-methylene-α-d-*xylo*-hexopyranoside (**37**)

Compound **36** (2.862 g, 5.235 mmol) was dissolved in abs. CH_2_Cl_2_ (29 mL). Dess-Martin periodinane (2.669 g, 6.282 mmol, 1.2 equiv.) was added and the reaction was stirred for one hour. When TLC showed complete disappearance of the starting material, the reaction mixture was diluted with CH_2_Cl_2_, aq NaOH solution (28 mL, 1.3 M) was added and the mixture was vigorously stirred for 10 min. In the next step, the organic layer was separated and washed with water, dried over MgSO_4_, and concentrated in vacuo. The crude product **4-ulose** (2.822 mg, 99%) was used in the next step without further purification. Dry tetrahydrofurane (30 mL) was stirred under argon and methyltriphenylphosphonium bromide (2.961 g, 8.289 mmol, 1.6 equiv.) was added. The suspension was cooled to 0 °C and *n*-butyllithium in hexane (3.316 mL, c = 2.5 M, 8.289 mmol) was added dropwise. After stirring the mixture for 30 min., **4-ulose** dissolved in dry tetrahydrofurane (15 mL) was added dropwise. The reaction was monitored by TLC. After thtre hours, ethyl acetate (200 mL) was added, and the organic layer was washed three times with satd aq NH_4_Cl solution and water, dried over MgSO_4_, and evaporated in vacuo. The crude product was purified by column chromatography to give **37** (1.831 g).

Yield: 65% (over two steps), colorless syrup. [α]_D_^20^ = +21.8 (*c* 0.1, CHCl_3_). R_f_ 0.73 (hexane/acetone 8/2). ^1^H NMR (400 MHz, CDCl_3_): δ = 7.72–7.67 (m, 4H, arom), 7.46-7.33 (m, 6H arom), 5.27 (s, 1H, methylene C*H*_2a_), 4.95 (s, 1H, methylene C*H*_2b_), 4.80 (d, *J* = 3.6 Hz, 1H, H-1), 4.52 (d, *J* = 10.3 Hz, 1H, H-3), 4.23 (t, *J* = 5.5 Hz, 1H, H-5), 4.03 (dd, *J* = 10.7, 4.9 Hz, 1H, H-6_a_), 3.88 (dd, *J* = 10.7, 6.4 Hz, 1H, H-6_b_), 3.69 (dd, *J* = 10.3, 3.6 Hz, 1H, H-2), 3.41 (s, 3H, OC*H_3_*), 3.26 (s, 3H, OC*H_3_*), 3.23 (s, 3H, OC*H_3_*), 1.36 (s, 3H, C*H_3_* butanedione), 1.34 (s, 3H C*H_3_* butanedione), 1.06 (d, *J* = 7.0 Hz, 9H, 3×C*H_3_*, *t*-Bu); ^13^C NMR (101 MHz, CDCl_3_): δ = 141.4(C-4), 135.8, 135.8, 134.9, 133.6, 133.6, 129.8, 127.9, 127.8 and 127.8 (arom), 106.2 (*C*H_2_), 100.0 and 99.9 (2xC_q_ butanedione), 98.1 (C-1), 71.9, 69.7, 66.4 and 63.8 (C-5, C-3, C-2), 55.0 (C-1-O*C*H_3_), 48.0 (2×O*C*H_3_ butanedione), 26.9 (3×*C*H_3_, *t*-Bu), 19.4 (C_q_, *t*-Bu), 18.0 and 17.9 (2×*C*H_3_ butanedione). Anal. Calcd for C_30_H_42_O_7_Si: C, 66.39; H, 7.80. Found: C, 67.88; H, 7.63.

#### 4.2.21. Methyl-6-*O*-*tert*-butyldiphenylsilyl-4-deoxy-4-*C*-(2′,3′,4′,6′-tetra-*O*-acetyl-1′-thiomethyl-β-d-glucopyranosyl)-2,3-(2″,3″-dimethoxybutane-2″3″-diyl)-α-d-glucopyranoside (**38a**) and Methyl-6-*O*-*tert*-butyldiphenylsilyl-4-deoxy-4-*C*-(2′,3′,4′,6′-tetra-*O*-acetyl-1′-sulfinylmethyl-β-d-glucopyranosyl)-2,3-(2″,3″-dimethoxybutane-2″3″-diyl)-α-d-glucopyranoside (**38b**)

4-Exomethylene **37** (1.380 g, 2.543 mmol), thiol **2a** (1.112 g, 3.051 mmol, 1.2 equiv.) and DPAP (65 mg, 0.254 mmol, 0.1 equiv.) were reacted according to the general method with one irradiation cycle to provide compounds **38a** and **38b** (2.056 g, **38a**:**38b** ~ 3:2 on the basis of ^1^H NMR spectrum, see supplementary file). The two products cannot be separated and used as a mixture in the next step. 

R_f_ 0.27 (hexane/acetone 8/2). ^1^H NMR (360 MHz, CDCl_3_) Characteristic peaks: δ 4.76 (d, *J* = 3.5 Hz, 1H, H-1major), 4.74 (d, *J* = 3.9 Hz, 0.6H, H-1minor), 4.49 (d, *J* = 10.1 Hz, 0.6H, H-1’minor), 4.26 (d, *J* = 9.8 Hz, 1H, H-1′major).

MS (ESI-TOF) *m*/*z*: [M + Na]^+^ **38a**: Calcd for C_44_H_62_O_16_SSiNa 929.3420; Found 929.3430; [M + Na]^+^ **38b**: Calcd for C_44_H_62_O_17_SSiNa 945.3369; Found 945.3375.

#### 4.2.22. Methyl-4-deoxy-4-*C*-(2′,3′,4′,6′-tetra-*O*-acetyl-1′-thiomethyl-β-d-glucopyranosyl)-2,3-(2″,3″-dimethoxybutane-2″3″-diyl)-α-d-glucopyranoside (**39a**) and Methyl-4-deoxy-4-*C*-(2′,3′,4′,6′-tetra-*O*-acetyl-1′-sulfinylmethyl-β-d-glucopyranosyl)-2,3-(2″,3″-dimethoxybutane-2″3″-diyl)-α-d-glucopyranoside (**39b**)

The mixture of **38a** and **38b** (2.056 g, 2.3 mmol) were dissolved in tetrahydrofurane (20 mL), 1M tetrabutyl-ammonium-fluoride in tetrahydrofurane (3.400 mL, 3.400 mmol, 1.5 equiv.) was added and the solution was stirred for 12 h. When TLC showed complete disappearance of the starting material the mixture was evaporated in vacuo, the crude product was purified by flash chromatography (hexane/acetone 75/25 → 65:35) to give **39a**,**b** as an inseparable mixture (877 mg). 

**39a**,**b**: R_f_ 0.12 (hexane/acetone 7/3). ^1^H NMR (360 MHz, CDCl_3_) Characteristic peaks: δ 4.78 (d, *J* = 3.5 Hz, 1H, H-1major), 4.72 (d, *J* = 3.9 Hz, 0.5H, H-1minor), 4.55 (d, *J* = 10.1 Hz, 0.5H, H-1’minor), 4.48 (d, *J* = 9.8 Hz, 1H, , H-1’major).

MS (ESI-TOF) *m*/*z*: [M + Na]^+^ **39a**,**b**: Calcd for C_28_H_44_O_16_SNa 691.225; Found 691.240; [M + Na]^+^ **39b**: Calcd for C_28_H_44_O_17_SNa 707.220; Found 707.224.

#### 4.2.23. Methyl-4-deoxy-4-*C*-(2′,3′,4′,6′-tetra-*O*-acetyl-1′-thiomethyl-β-d-glucopyranosyl)-α-d-glucopyranoside (**40**)

The mixture of **39a**,**b** (850 mg) was dissolved in CH_2_Cl_2_ and 3 mL 90 *v*/*v*% trifluoroacetic acid (2.7 mL trifluoroacetic acid + 0.3 mL water) was added dropwise. After 15 min. toluene (5 mL) was added and the mixture was evaporated in vacuo. The crude product was purified by flash chromatography to provide **40** (443 mg).

Yield: 31% for three steps, colorless syrup. R_f_ 0.13 (CH_2_Cl_2_/acetone 7/3). ^1^H NMR (360 MHz, CDCl_3_): δ = 5.23 (t, *J* = 9.3 Hz, 1H), 5.07 (t, *J* = 9.8 Hz, 1H) and 5.07 (t, *J* = 9.7 Hz, 1H) (H-2′, H-3′, H-4′), 4.81 (d, *J* = 3.8 Hz, 1H, H-1), 4.51 (d, *J* = 10.0 Hz, 1H, H-1’), 4.27–4.17 (m, 2H, H-6′_a_, H-6′_b_), 3.84–3.65 (m, 6H) and 3.51 (dd, *J* = 9.2, 3.7 Hz, 1H) (H-2, H-3, H-4, H-5, H-6_a_, H-6_b_, H-5′), 3.41 (s, 3H, OC*H*_3_), 3.02 (dd, *J* = 13.6, 3.1 Hz, 1H, SC*H*_2a_), 2.90 (dd, *J* = 13.6, 4.2 Hz, 1H, SC*H*_2b_), 2.12 (s, 3H, COC*H*_3_), 2.08 (s, 3H, COC*H*_3_), 2.04 (s, 3H, COC*H*_3_), 2.01 (s, 3H, COC*H*_3_), ^13^C NMR (91 MHz, CDCl_3_): δ = 99.8 (C-1), 83.7 (C-1′), 76.2, 73.8, 73.7, 70.8, 69.9, 69.8 and 68.5 (7C, skeleton carbons), 62.5 and 62.2 (C-6 and C-6′), 55.5 (C-4), 42.4 (S*C*H_2_), 26.9, 20.9 and 20.7 (4C, 4×CO*C*H_3_), MS (MALDI-TOF) *m*/*z*: [M + Na]^+^ Calcd for C_22_H_34_O_14_SNa 577.157; Found: 577.169.

#### 4.2.24. Methyl-2,3,6-tri-*O*-acetyl-4-deoxy-4-*C*-(2′,3′,4′,6′-tetra-*O*-acetyl-1′-thiomethyl-β-d-glucopyranosyl)-α-d-glucopyranoside (**41a**) and Methyl-2,3,6-tri-*O*-acetyl-4-deoxy-4-*C*-(2′,3′,4′,6′-tetra-*O*-acetyl-1′-sulfinylmethyl-β-d-glucopyranosyl)-α-d-glucopyranoside (**41b**)

Compound **40** (430 mg) was dissolved in CH_2_Cl_2_, acetic anhydride (0.141 mL, 1.487 mmol, 3.6 equiv.) and pyridine (0.100 mL, 1.237 mmol, 3.0 equiv.) was added. The mixture was stirred overnight. When TLC showed the complete disappearance of the starting material, the solvent was evaporated in vacuo, and the crude product was purified by column chromatography to give **41a** and **41b** (223 mg). **41a** and **41b** cannot be separated from each other.

Yield: 69%, colorless syrup. R_f_ 0.82 (CH_2_Cl_2_/acetone 7/3). 

^1^H NMR (400 MHz, CDCl_3_): δ 5.41 (dd, *J* = 10.9, 9.7 Hz, 1H, **H-3**), 5.28 (dd, *J* = 5.3, 1.7 Hz, 0.3H), 5.21 (t, *J* = 9.4 Hz, 1H), 5.08 (t, *J* = 9.7 Hz, 1H), 5.01 (td, *J* = 9.8, 3.6 Hz, 2H), 4.96–4.91 (m, 1H), 4.89 (d, *J* = 3.6 Hz, 1H), 4.82 (dd, *J* = 9.7, 3.6 Hz, 1H), 4.40 (d, *J* = 9.8 Hz, 2H), 4.36–4.15 (m, 8H), 4.12 (dt, *J* = 10.7, 3.5 Hz, 1H), 3.81 (ddd, *J* = 10.1, 4.3, 2.2 Hz, 1H), 3.70 (ddd, *J* = 10.1, 4.9, 2.3 Hz, 1H), 3.38 (s, 2H), 3.37 (s, 3H), 2.99 (dd, *J* = 13.1, 3.3 Hz, 1H), 2.84 (dd, *J* = 13.1, 7.7 Hz, 1H), 2.78 (d, *J* = 3.9 Hz, 2H), 2.56 (q, *J* = 4.1 Hz, 1H), 2.22 (ddt, *J* = 14.9, 11.8, 3.8 Hz, 1H), 2.11 (s, 3H), 2.10 (s, 1H), 2.10 (s, 6H), 2.09 (s, 4H), 2.08 (d, *J* = 0.8 Hz, 1H), 2.06 (s, 3H), 2.06 (s, 3H), 2.03 (s, 1H), 2.02 (s, 3H), 2.01 (s, 1H), 2.00 (s, 3H). ^13^C NMR (101 MHz, CDCl_3_) δ 170.78, 170.75, 170.69, 170.21, 170.15, 169.68, 169.49, 169.47, 169.45, 97.24, 97.02, 83.84, 82.52, 76.05, 73.80, 72.67, 69.83, 69.58, 69.12, 68.41, 68.28, 68.12, 67.82, 67.15, 64.13, 63.25, 62.04, 61.90, 55.35, 55.15, 41.26, 40.48, 25.54, 22.44, 21.04, 20.92, 20.77, 20.68. MS (MALDI-TOF) *m*/*z*: [M + Na]^+^ **41a**: Calcd for C_28_H_40_O_17_SNa 703.188; Found 703.199; **41b**: *m*/*z*: [M + Na^+^] Calcd for C_28_H_40_O_18_SNa 719.183; Found 719.195.

#### 4.2.25. Methyl-6-*O*-*tert*-butyldiphenylsilyl-2,3-di-*O*-methyl-α-d-glucopyranoside (**42**)

Methyl-2,3-di-*O*-methyl-α-d-glucopyranoside [69] was dissolved in abs. CH_2_Cl_2_ (5 mL) and pyridine (0.653 mL, 6.75 mmol 2 equiv.). *tert*-Butyl-diphenylsilyl chloride (1.038 mL, 4.050 mmol, 1.2 equiv.) was added and the mixture was stirred overnight. When the reaction was complete, CH_2_Cl_2_ was added and the mixture was extracted with 1M HCl, satd aq NaHCO_3_ solution and water. The organic phase was dried over MgSO_4_ and then concentrated; the crude product was purified by flash chromatography to give **42** (1.180 g).

Yield: 76%, colorless syrup. [α]_D_^20^ = +48.7 (*c* 0.2, CHCl_3_). R_f_ 0.77 (CH_2_Cl_2_/acetone 8/2). ^1^H NMR (400 MHz, CDCl_3_): δ = 7.73–7.35 (m, 10H, arom), 4.82 (d, *J* = 3.5 Hz, 1H, H-1), 3.93–3.83 (m, 2H, H-6_a_ and H-6_b_), 3.69–3.63 (m, 1H, H-5), 3.64 (s, 3H, OC*H*_3_), 3.56 (dt, *J* = 9.4, 1.6 Hz, 1H, H-4), 3.50 (s, 3H, OC*H*_3_), 3.50–3.45 (m, *J* = 8.5, 4.5 Hz, 1H, H-3), 3.39 (s, 3H, OC*H*_3_), (dd, *J* = 9.4, 3.6 Hz, 1H, H-2), 2.77 (d, *J* = 2.0 Hz, 1H, OH), 1.06 (s, 9H, 3×C*H*_3_, *t*-Bu), ^13^C NMR (101 MHz, CDCl_3_): δ = 135.7, 133.3, 129.9 and 127.8 (12C, arom), 97.4 (C-1), 83.0, 81.9, 71.7 and 70.8 (4C, skeleton carbons), 64.5 (C-6), 61.3 (OC*H*_3_), 58.6 (OC*H*_3_), 55.1 (OC*H*_3_), 26.9 (3C, 3×*C*H_3_, *t*-Bu), 19.3 (C_q_, *t*-Bu). Anal. Calcd for C_25_H_36_O_6_Si: C, 65.19; H, 7.88. Found: C, 67.02; H, 7.65.

#### 4.2.26. Methyl-6-*O*-*tert*-butyldiphenylsilyl-2,3-di-*O*-methyl-α-d-*xylo*-hexopyranoside-4-ulose (**43**)

Dess–Martin periodinane (1.239 g, 2.915 mmol, 1.2 equiv.) was added and the mixture was stirred for one hour to a solution of **42** [46] (1.119 g, 2.429 mmol) dissolved in abs. CH_2_Cl_2_ (10 mL). The mixture was diluted with CH_2_Cl_2_, aq NaOH solution (19 mL, 1.3M) was added and vigorously stirred for 10 min. The organic phase was separated and washed with water, over MgSO_4_ concentrated to give **43** (1.056 g). The crude **43** was used in the next step without further purification.

Yield: 94%, colorless syrup. R_f_ = 0.56 (hexane/ethyl acetate 6/4). ^1^H NMR (400 MHz, CDCl_3_): δ = 7.74–7.34 (m, 10H, arom), 5.03 (d, *J* = 3.4 Hz, 1H, H-1), 4.19 (dd, *J* = 6.4, 3.1 Hz, 1H), 4.11–4.06 (m, 2H, H-6_a_ and H-6_b_), 3.89 (dd, *J* = 11.3, 6.4 Hz, 1H), 3.58 (s, 3H, OC*H*_3_), 3.56 (s, 3H, OC*H*_3_), 3.54 (s, 3H, OC*H*_3_), 3.53–3.50 (m, *J* = 6.2, 2.1 Hz, 1H), 1.04 (s, 9H, 3×C*H*_3_, *t*-Bu), ^13^C NMR (101 MHz, CDCl_3_): δ = 135.8, 135.7, 133.5, 133.3, 129.8, 127.8, 127.8 (12C, arom), 97.6 (C-1), 84.6, 82.7 and 74.3 (C-2, C-3 and C-5), 62.1 (C-6), 60.3 (OC*H*_3_), 59.8 (OC*H*_3_), 56.0 (OC*H*_3_), 26.8 (3C, 3×*C*H_3_, *t*-Bu), 19.3 (C_q_, *t*-Bu). 

#### 4.2.27. Methyl-6-*O*-*tert*-butyldiphenylsilyl-4-deoxy-4-methylene-2,3-di-*O*-methyl-α-d-*xylo*-hexopyranoside (**44**)

Dry tetrahydrofurane (10 mL) was stirred under argon and methyltriphenylphosphonium bromide (1.255 g, 3.513 mmol) was added. The suspension was cooled to 0 °C and *n*-butyllithium in hexane (1.405 mL, c = 2.5 M, 3.513 mmol) was added dropwise. After stirring the mixture for 30 min., **43** (1.007 g, 2.196 mmol) dissolved in dry tetrahydrofurane (5 mL) was added dropwise. The reaction was monitored by TLC. After three hours, ethyl acetate (100 mL) was added, and the organic layer was washed three times with satd aq NH_4_Cl solution and water, over MgSO_4_ evaporated in vacuo. The crude product was purified by column chromatography to give **44** (920 mg).

Yield: 92%, colorless syrup. [α]_D_^20^ = +95.0 (*c* 0.2, CHCl_3_). R_f_ 0.46 (hexane/ethyl acetate 7/3). ^1^H NMR (400 MHz, CDCl_3_): δ = 7.73–7.35 (m, 10H, arom), 5.20 (s, 1H, =C*H*_2a_), 4.96 (s, 1H, =C*H*_2b_), 4.88 (d, *J* = 3.6 Hz, 1H, H-1), 4.17 (t, *J* = 5.7 Hz, 1H, H-5), 4.02–3.94 (m, 2H, H-6_a_ and H-3), 3.88 (dd, *J* = 10.6, 6.6 Hz, 1H, H-6_b_), 3.53 (s, 3H, OC*H*_3_), 3.53 (s, 3H, OC*H*_3_), 3.42 (s, 3H, OC*H*_3_), 3.18 (dd, *J* = 9.5, 3.6 Hz, 1H, H-2), 1.05 (s, 9H, 3×C*H*_3_, *t*-Bu); ^13^C NMR (101 MHz, CDCl_3_): δ = 142.5 (C-4), 135.8, 135.8, 133.6, 132.6, 129.9, 127.8 and 127.8 (12C, arom), 107.4 (=*C*H_2_), 97.9 (C-1), 83.8, 81.2 and 69.6 (C-2, C-3 and C-5), 63.9 (C-6), 59.5 (OC*H*_3_), 59.4 (OC*H*_3_), 55.2 (OC*H*_3_), 26.9 (3C, 3×*C*H_3_, *t*-Bu), 19.4 (C_q_, *t*-Bu). Anal. Calcd for C_26_H_36_O_5_Si: C, 68.39; H, 7.95. Found: C, 66.74; H, 8.18.

#### 4.2.28. Methyl-6-*O*-*tert*-butyldiphenylsilyl-4-deoxy-4-*C*-(2′,3′,4′,6′-tetra-*O*-acetyl-1′-thiomethyl-β-d-glucopyranosyl)-2,3-di-*O*-methyl-α-d-glucopyranoside (**45**)

Preparation by photoinduced free radical hydrothiolation: **44** (106 mg, 0.232 mmol), and **2a** (100 mg, 0.278 mmol, 1.2 equiv.) were reacted according to the general method with two irradiation cycle, to give compound **45** (153 mg, 81%). 

The preparation by Et_3_B-catechol initiated free radical hydrothiolation: **44** (100 mg, 0.262 mmol) and **2a** (95 mg, 0.182 mmol, 1.2 equiv.) were dissolved in CH_2_Cl_2_ (2 mL) and catechol (29 mg, 0.263 mmol, 1.2 equiv.) and Et_3_B in hexane (307 μL, c = 1 M, 0.307 mmol, 1.4 equiv.) were added. After 1 h, the solvents was evaporated and the crude product was purified by column chromatography to give **45** (116 mg, 78%).

Yield: 81%, [α]_D_^20^ = +1.30 (*c* = 0.23, CHCl_3_). R_f_ 0.33 (CH_2_Cl_2_/acetone 95/5). ^1^H NMR (400 MHz, CDCl_3_): δ = 7.74–7.35 (m, 10H, arom), 5.19 (t, *J* = 9.4 Hz, 1H, H-3′), 5.03 (t, *J* = 9.7 Hz, 1H, H-4’), 4.95 (t, *J* = 9.7 Hz, 1H, H-2′), 4.86 (d, *J* = 3.5 Hz, 1H, H-1), 4.38 (d, *J* = 10.1 Hz, 1H, H-1′), 4.17 (dd, *J* = 12.4, 4.9 Hz, 1H, H-6′_a_), 4.01 (dd, *J* = 12.4, 2.2 Hz, 1H, H-6′_b_), 3.89 (dd, *J* = 11.2, 2.2 Hz, 1H, H-6_b_), 3.74 (dd, *J* = 11.3, 4.7 Hz, 1H, H-6_a_), 3.68 (ddd, *J* = 10.5, 4.4, 2.2 Hz, 1H, H-5), 3.62–3.59 (m, 1H, H-5′), 3.58 (s, 3H, OC*H*_3_), 3.52 (s, 3H, OC*H*_3_), 3.46–3.39 (m, 1H, H-3), 3.39 (s, 3H, OC*H*_3_), 3.23 (dd, *J* = 9.2, 3.5 Hz, 1H, H-2), 2.82 (dd, *J* = 13.1, 4.6 Hz, 1H, SC*H*_2a_), 2.71 (dd, *J* = 13.1, 2.8 Hz, 1H, SC*H*_2b_), 2.02 (s, 3H, COC*H*_3_), 2.01 (s, 3H, COC*H*_3_), 2.00 (s, 3H, COC*H*_3_), 1.98–1.94 (m, 1H, H-4), 1.93 (s, 3H, COC*H*_3_), 1.06 (s, 9H, 3×C*H*_3_, *t*-Bu), ^13^C NMR (101 MHz, CDCl_3_): δ = 170.5, 170.2, 169.3 and 169.1 (4×*C*OCH_3_), 135.7, 135.7, 135.7, 133.5, 133.4, 129.8, 129.7, 127.7 and 127.7 (10C, arom), 97.6 (C-1), 83.6 (C-2), 83.4 (C-1′), 78.7 (C-3), 75.8 (C-5′), 73.9 (C-3′), 71.1 (C-5), 69.8 (C-2′), 68.3 (C-4′), 64.1 (C-6), 62.0 (C-6′), 61.1, 58.5 and 55.0 (3×O*C*H_3_), 43.3 (C-4), 26.8 (4C, 3×*C*H_3_, *t*-Bu and S*C*H_2_), 20.6, 20.6, 20.6 and 20.5 (4C, 4×CO*C*H_3_), 19.3 (C_q_, *t*-Bu). Anal. Calcd for C_40_H_56_O_14_SSi: C, 58.53; H, 6.88; S, 3.89. Found: C, 59.90; H, 7.05; S, 3.60.

#### 4.2.29. 2,3,4-Tri-*O*-acetyl-α-d-*xylo*-hex-5-enopyranosyl-(1→1)-2,3,4-tri-*O*-acetyl-α-d-*xylo*-hex-5-enopyranoside (**47**)

To a solution of **46** (1.63 g, 2 mmol) in pyridine (50 mL) AgF (3.16 g, 25 mmol) was added and the mixture was stirred in the dark overnight, and then the completion of the reaction was controlled by TLC (CH_2_Cl_2_:acetone 95:5). Et_2_O (320 mL) was added to the mixture and the organic layer was washed with 10% aq Na_2_S_2_O_3_ solution and water. The organic phase was dried over Na_2_SO_4_, evaporated under reduced pressure, and the residue was purified by column chromatography to give **47** (608 mg).

Yield: 54%, white needles. m.p.: 214.3 °C [α]_D_^20^ = + 11.2 (*c* 0.08, CHCl_3_). R_f_ 0.35 (hexane/acetone 7/3). ^1^H NMR (400 MHz, CDCl_3_): δ = 5.52 (d, *J* = 3.6 Hz, 1H, H-1), 5.50-5.35 (m, *2*H, H-3 and H-4), 5.02 (dd, *J* = 8.6, 3.6, Hz, 1H, H-2), 4.66 (t, *J* = 2.0 Hz, 1H, =C*H*_2a_), 4.55 (t, *J* = 1.8 Hz, 1H, =C*H*_2b_), 2.11 (s, 3H, C*H_3_*CO), 2.06 (s, 3H, C*H_3_*CO), 2.04 (s, 3H, C*H_3_*CO).^13^C NMR (101 MHz, CDCl_3_): δ = 170.1, 169.5, 169.3, (3C, 3 x CO) 149.7 (C-5), 97.7 (C-6), 90.3 (C-1), 69.7, 69.3, 69.2 (3C, C-2, C-3 and C-4), 20.7, 20.7, 20.6 (3C, 3 x *C*H_3_CO). MS (MALDI-TOF) *m*/*z*: [M + Na]^+^ Calcd for C_24_H_30_ NaO_15_ 581.1482; Found: 581.323.

#### 4.2.30. 2,3,4-Tri-*O*-acetyl-6-thio-6-*S*-(2,3,4,6-tetra-*O*-acetyl-α-d-mannopyranosyl)-α-d-glucopyranosyl-(1→1)-2,3,4-tri-*O*-acetyl-α-d-*xylo*-hex-5-enopyranoside (**48**) and 2,3,4-Tri-*O*-acetyl-6-thio-6-*S*-(2,3,4,6-tetra-*O*-acetyl-α-d-mannopyranosyl)-α-d-glucopyranosyl-(1→1)-2,3,4-tri-*O*-acetyl-6-thio-6-*S*-(2,3,4,6-tetra-*O*-acetyl-α-d-mannopyranosyl)-α-d-glucopyranoside (**49**)

Diene **47** (100 mg, 0.18 mmol), thiol **2e** (195 mg, 0.54 mmol, 3.0 equiv.) and DPAP (5 mg, 0.1 equiv.) were reacted according to the general method in DMF:toluene 3:1 (3 mL) with four irradiation cycles. The crude product was purified by column chromatography (hexane/acetone 6/4) to give compound **48** (44 mg, 27%) as a colorless foam. The reaction was also carried out in the same scale in DMF:toluene 3:1 at −80 °C to give compounds **48** (55 mg, 33%) and **49** (41 mg, 18%). The reaction was repeated while using 4.5 equiv. of thiol **2e** in DMF:toluene 3:1 at −80 °C to give compounds **48** (7 mg, 4 %) and **49** (135 mg, 60%). The reaction was repeated using 4.5 equiv. of thiol **2e** in dichloromethane to give compound **49** (164 mg, 74%).

**48**: Yield: 33%, colorless foam. [α]_D_^20^ = +196.6 (c = 0.18, CHCl_3_). R_f_ 0.40 (hexane/acetone 6/4). ^1^H NMR (400 MHz, CDCl_3_) δ (ppm) 5.51 (d, 1H, *J* = 3.6 Hz, 1H, H-1_enoside_), 5.47–5.43 (m, 2H), 5.41 (d, 1H, *J* = 3.6 Hz, 1H, H-1_Glcp_), 5.34 (dd, *J* = 3.2, 1.4 Hz, 1H), 5.33–5.25 (m, 5H), 5.16 (ddd, *J* = 9.2, 3.6, 0.8 Hz, 1H), 5.06–4.99 (m, 1H), 4.91 (dd, *J* = 10.2, 3.9 Hz, 1H), 4.67 (t, *J* = 1.9 Hz, 1H, =CH_2a_), 4.55 (t, *J* = 1.8 Hz, 1H, =CH_2b_), 4.33 (dd, *J* = 12.2, 5.0 Hz, 1H, H-6a_Manp_), 4.26 (ddd, *J* = 9.5, 4.9, 2.1 Hz, 1H, H-5_Manp_), 4.07 (dd, *J* = 12.2, 2.2 Hz, 1H, H-6b_Manp_), 3.95 (ddd, *J* = 10.0, 6.9, 3.0 Hz, 1H, H-5_Glcp_), 2.81 (dd, *J* = 14.0, 3.1 Hz, 1H, SCH_2a_), 2.65 (dd, *J* = 14.0, 7.0 Hz, 1H, SCH_2b_), 2.18 (s, 3H, CH_3_CO), 2.13 (s, 3H, CH_3_CO), 2.12 (s, 3H, CH_3_CO), 2.11 (s, 3H, CH_3_CO), 2.05 (s, 3H, CH_3_CO), 2.05–2.04 (m, 9H, 3×CH_3_CO), 2.03 (s, 3H, CH_3_CO), 2.00 (s, 3H, CH_3_CO). ^13^C NMR (101 MHz, CDCl_3_) δ 170.7, 170.247, 169.9, 169.9, 169.8, 169.7, 169.7, 169.4 (10C, 10×CO), 149.8 (C-5), 98.0 (C-6), 91.4, 90.9 (2C, 2×C-1_trehalose_), 82.6 (C-1_Manp_), 71.2, 70.9, 70.0, 69.6, 69.8, 69.7, 69.5, 69.4, 69.2 (2×), 66.2 (11C, skeleton), 62.3 (C-6_Manp_), 31.3 (SCH_2_), 21.0, 20.9 (2×), 20.8 (5×), 20.7 (2×) (10C, 10×CH_3_CO). MS (MALDI-TOF) *m*/*z*: [M + Na]^+^ Calcd for C_38_H_50_NaO_24_S 945.231; Found: 945.230.

**49**: Yield: 74%, colorless foam. [α]_D_^20^ = +49.3 (c = 0.15, CHCl_3_). R_f_ 0.24 (hexane/acetone 6/4). ^1^H NMR (400 MHz, CDCl_3_) δ (ppm) 5.46 (t, *J* = 9.8 Hz, 1H), 5.36–5.21 (m, 5H), 5.05 (dt, *J* = 9.6, 6.9 Hz, 2H), 4.39–4.23 (m, 2H), 4.14–3.96 (m, 2H), 2.82 (dd, *J* = 13.9, 3.0 Hz, 1H, SC*H*_2a_), 2.67 (dd, *J* = 14.0, 6.6 Hz, 1H, SC*H*_2b_), 2.17 (s, 3H, C*H_3_*CO), 2.11 (s, 6H, 2 x C*H_3_*CO), 2.06 (s, 6H, 2×C*H_3_*CO), 2.03 (s, 3H, C*H_3_*CO), 1.99 (s, 3H, C*H_3_*CO).^13^C NMR (101 MHz, CDCl_3_) δ 170.6, 170.0, 169.9, 169.8, 169.7, 169.7 (2×) (7×CO), 92.1 (1C, C-1_trehalose_), 82.4 (C-1_Manp_), 71.1, 70.8, 70.0, 69.9, 69.5, 69.4, 69.3, 66.2 (8C, skeleton), 62.4 (C-6_Manp_), 31.3 (S*C*H_2_), 20.9, 20.8, 20.7 (3×), 20.6 (7C, 7 x *C*H_3_CO). MS (MALDI-TOF) *m*/*z*: [M + Na]^+^ Calcd for C_52_H_70_NaO_33_S_2_ 1309.314; Found: 1309.319.

#### 4.2.31. Methyl (5-acetamido-4,7,8,9-tetra-*O*-acetyl-3,5-dideoxy-2-thio-d-*glycero*-α-d-*galacto*-non-2-ulopyranosyl)-onate-(2→7)-6’-deoxy-1′,2′:3′,4′-di-*O*-isopropylidene-7′-S-α-d-*galacto*-heptopyranose (**51**)

Exomethylene **50** (22 mg, 0.11 mmol) and thiol **2f** (66 mg, 0.13 mmol) were reacted according to the general method at −80 °C with three irradiation cycles to give compound **51** (65 mg).

Yield: 69%, colorless syrup. [α]_D_^20^ = −4.0 (c = 0.5, CHCl_3_). R_f_ 0.29 (CH_2_Cl_2_/aceton 8/2). ^1^H NMR (500 MHz, CDCl_3_): δ = 5.49 (d, *J* = 5.0 Hz, 1H, H-1′), 5.38 (ddd, *J* = 8.0, 5.1, 2.7 Hz, 1H, H-8), 5.31 (dd, *J* = 8.5, 2.2 Hz, 1H, H-7), 5.16 (d, *J* = 10.1 Hz, 1H, NH), 4.86 (ddd, *J* = 11.7, 10.5, 4.6 Hz, 1H, H-4), 4.59 (dd, *J* = 7.9, 2.3 Hz, 1H, H-3′), 4.32–4.27 (m, 2H, H-9_a_, H-2′), 4.17 (dd, *J* = 7.9, 1.8 Hz, 1H, H-4′), 4.11 (dd, *J* = 12.5, 5.2 Hz, 1H, H-9b), 4.04 (q, *J* = 10.4 Hz, 1H, H-5), 3.86 (ddd, *J* = 8.9, 4.0, 1.4 Hz, 1H, H-5’), 3.82 (dd, *J* = 10.8, 2.2 Hz, 1H, H-6), 3.80 (s, 3H, OC*H_3_*), 2.84–2.68 (m, 3H, H-7′_a_, H-7′_b_, H-3_a_), 2.14 (s, 6H, 2×COCH_3_), 2.03 (s, 3H, COCH_3_), 2.03 (s, 3H, COCH_3_), 2.01–1.96 (m, 1H, H-3_b_), 1.93–1.88 (m, 1H, H-6’_a_), 1.87 (s, 3H, COCH_3_), 1.81–1.74 (m, 1H, H-6’_b_), 1.59 (s, 3H, C*H_3_*, *i*-Pr), 1.44 (s, 3H, C*H_3_*, *i*-Pr), 1.34 (s, 3H, C*H_3_*, *i*-Pr), 1.32 (s, 3H, C*H_3_*, *i*-Pr); ^13^C NMR (91 MHz, CDCl_3_) δ (ppm) 171.1, 170.8, 170.3 and 170.0 (6C, 6×CO), 109.2 and 108.6 (2C, 2C_q_, *i*-Pr), 96.6 (C-1′), 83.4 (C-2), 74.3 (C-6), 72.7 (C-4′), 71.1 (C-3’), 70.7 (C-2’), 69.8 (C-4), 68.6 (C-8), 67.4 (C-7), 66.3 (C-5’), 62.3 (C-9), 53.1 (O*C*H_3_), 49.5 (C-5), 38.2 (C-3), 30.8 (C-6’), 26.2 (*C*H_3_, *i*-Pr), 26.1 (*C*H_3_, *i*-Pr), 25.4 (C-7’), 25.2 (*C*H_3_, *i*-Pr), 24.5 (*C*H_3_, *i*-Pr), 23.4, 21.2, 21.0 and 20.9 (5C, 5×CO*C*H_3_); MS (ESI-QTOF) m/z: [M+Na]^+^ Calcd for C_33_H_49_NNaO_17_S 786.2619; Found 786.2616.

## Supplementary Materials

Supplementary materials can be found at https://www.mdpi.com/1422-0067/21/2/573/s1.

## Abbreviations

DPAP	2,2-dimethoxy-2-phenylacetophenone
BDA	butane-2,3-diacetal
DIPEA	*N*,*N*-diisopropylethylamine
TBDMS	*tert*-butyldimethylsilyl
TBDPS	*tert*-butyldiphenylsilyl
TFA	trifluoroacetic acid
TBAF	tetrabutylammonium fluoride
