# Supplementary material for: Stereoselective Synthesis of Carbon-Sulfur-Bridged Glycomimetics by Photoinitiated Thiol-Ene Coupling Reactions
Source: Int J Mol Sci. 2020 Jan 16;21(2):573. doi: 10.3390/ijms21020573 (PMC7013897; doi:10.3390/ijms21020573)
Supplement: Supplementary file 1 [file ijms-21-00573-s001.pdf]

Supplementary data

Stereoselective Synthesis of Carbon-Sulfur-Bridged Glycomimetics by Photoinitiated Thiol-Ene Coupling Reactions

Magdolna Csávás [1], Dániel Eszenyi [1], Erika Mező [1], László Lázár [2], Nóra Debreczeni [1,3], Marietta Tóth [2], László Somsák [2] and Anikó Borbás [1,*]

[1] Department of Pharmaceutical Chemistry University of Debrecen, Egyetem tér 1, H-4032, Debrecen, Hungary; csavas.magdolna@science.unideb.hu (M.C.), eszenyid@gmail.com (D.E.), mezo.erika@science.unideb.hu (E.M.), debreczeni.nora@science.unideb.hu (N.D.)

[2] Department of Organic Chemistry, University of Debrecen, Egyetem tér 1, H-4032, Debrecen, Hungary; lazar.laszlo@science.unideb.hu (L.L.), toth.marietta@science.unideb.hu (M.T.), somsak.laszlo@science.unideb.hu (L.S.)

[3] Doctoral School of Chemistry, University of Debrecen, Egyetem tér 1, H-4032, Debrecen, Hungary

* Correspondence: borbas.aniko@pharm.unideb.hu; Tel.: +36-52-512900-22472 (A.B.)

NMR spectra of selected compounds

The ^{1}H NMR (360 and 400 MHz) and ^{13}C NMR (90.54 and 100.28 MHz) spectra were recorded with Bruker DRX-360 and DRX-400 spectrometers at 25 °C. Chemical shifts are referenced to Me_4Si or DSS (0.00 ppm for ^{1}H) and to the solvent signals ($CDCl_3$: 77.00 ppm for ^{13}C).

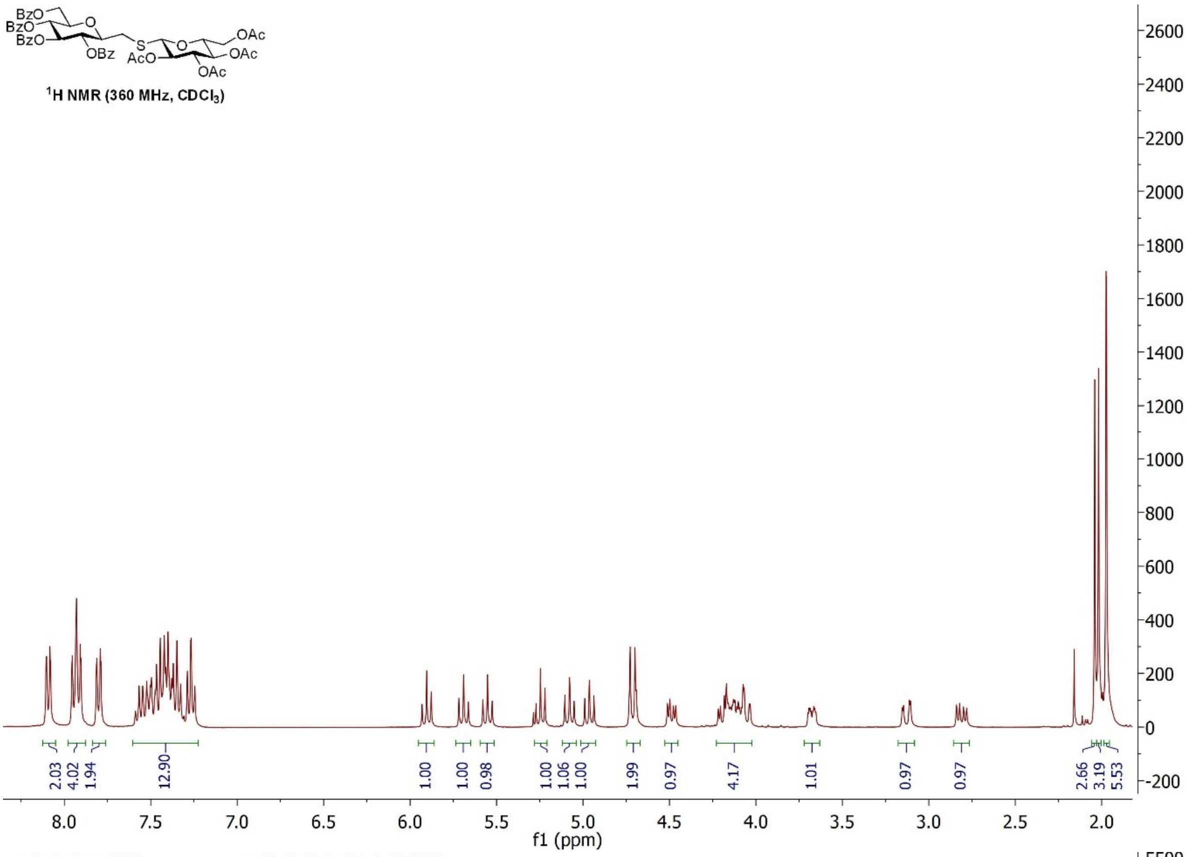
BzO
BzO
BzO
OBz
S
AcO
OAc
OAc
OAc
1H NMR (360 MHz, CDCl3)
2.03
4.02
1.94
12.90
1.00
1.00
0.98
1.00
1.06
1.00
1.99
0.97
4.17
1.01
0.97
0.97
2.66
3.19
5.53
f1 (ppm)

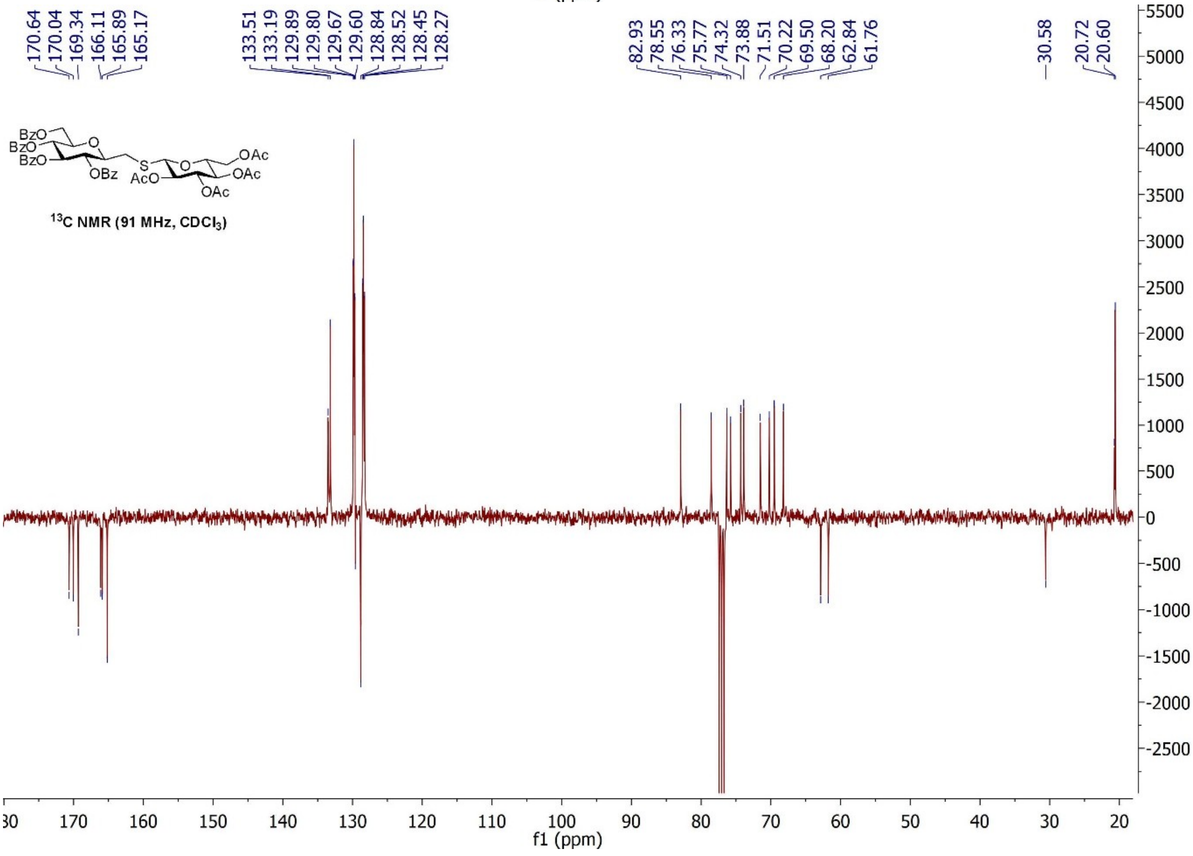
170.64
170.04
169.34
166.11
165.89
165.17
133.51
133.19
129.89
129.80
129.67
129.60
128.84
128.52
128.45
128.27
82.93
78.55
76.33
75.77
74.32
73.88
71.51
70.22
69.50
68.20
62.84
61.76
30.58
20.72
20.60
BzO
BzO
BzO
OBz
S
AcO
OAc
OAc
OAc
13C NMR (91 MHz, CDCl3)
f1 (ppm)

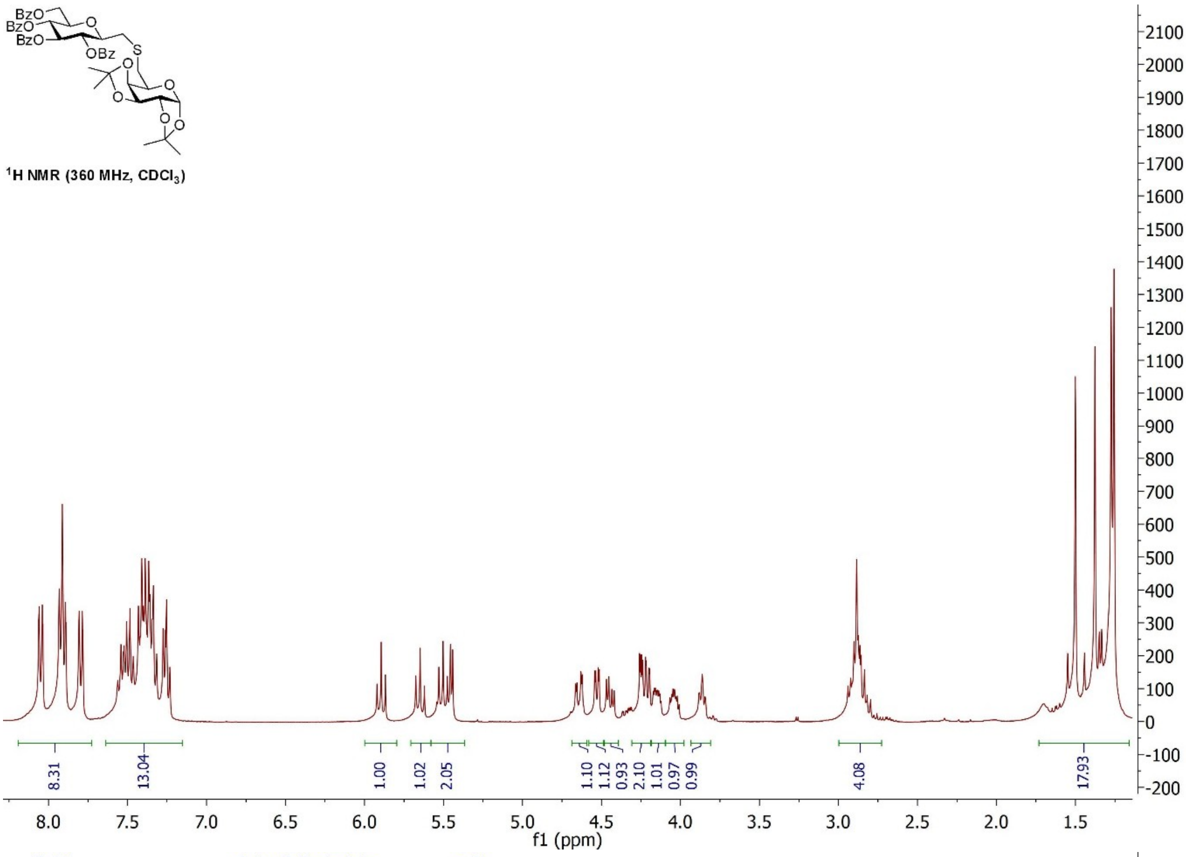
BzO
BzO
BzO
OBz
S
^{1}H NMR (360 MHz, $CDCl_3$)
8.31
13.04
1.00
1.02
2.05
1.10
1.12
0.93
2.10
1.01
0.97
0.99
4.08
17.93
f1 (ppm)

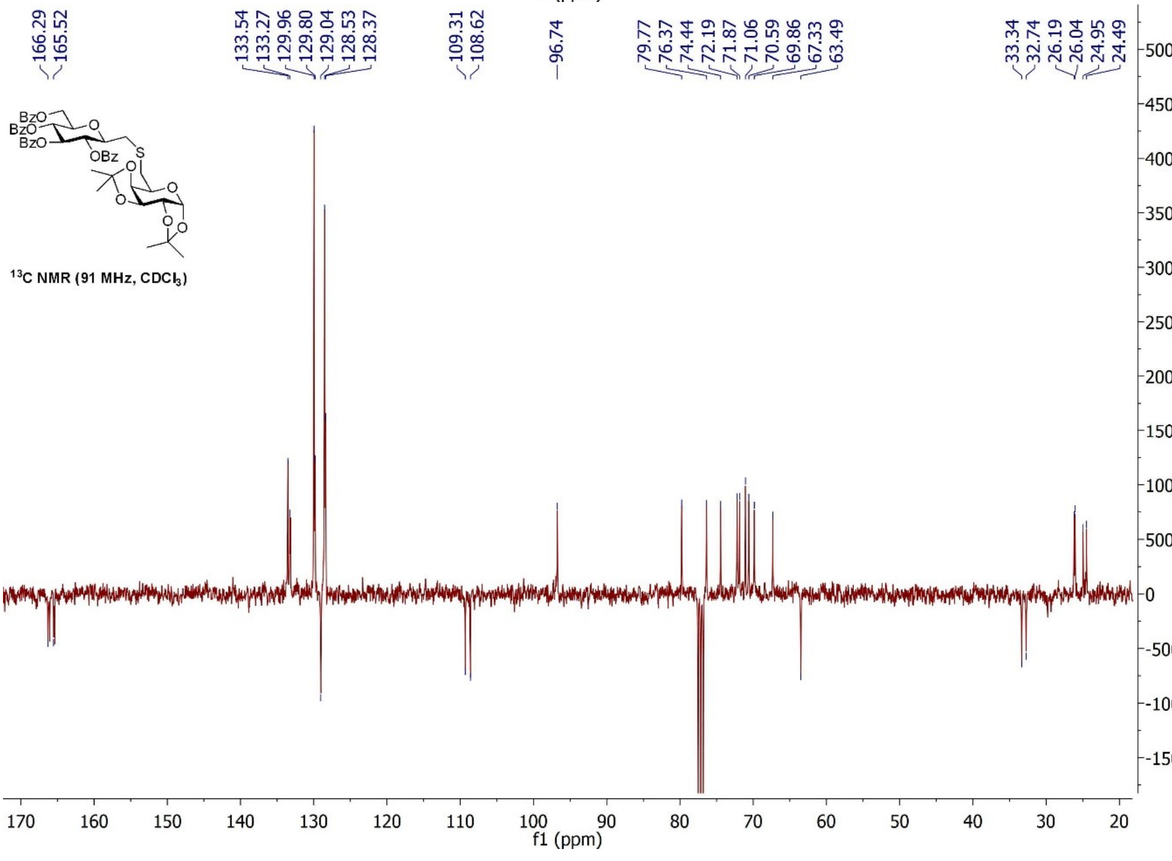
166.29
165.52
133.54
133.27
129.96
129.80
129.04
128.53
128.37
109.31
108.62
96.74
79.77
76.37
74.44
72.19
71.87
71.06
70.59
69.86
67.33
63.49
33.34
32.74
26.19
26.04
24.95
24.49
BzO
BzO
BzO
OBz
S
^{13}C NMR (91 MHz, $CDCl_3$)
f1 (ppm)

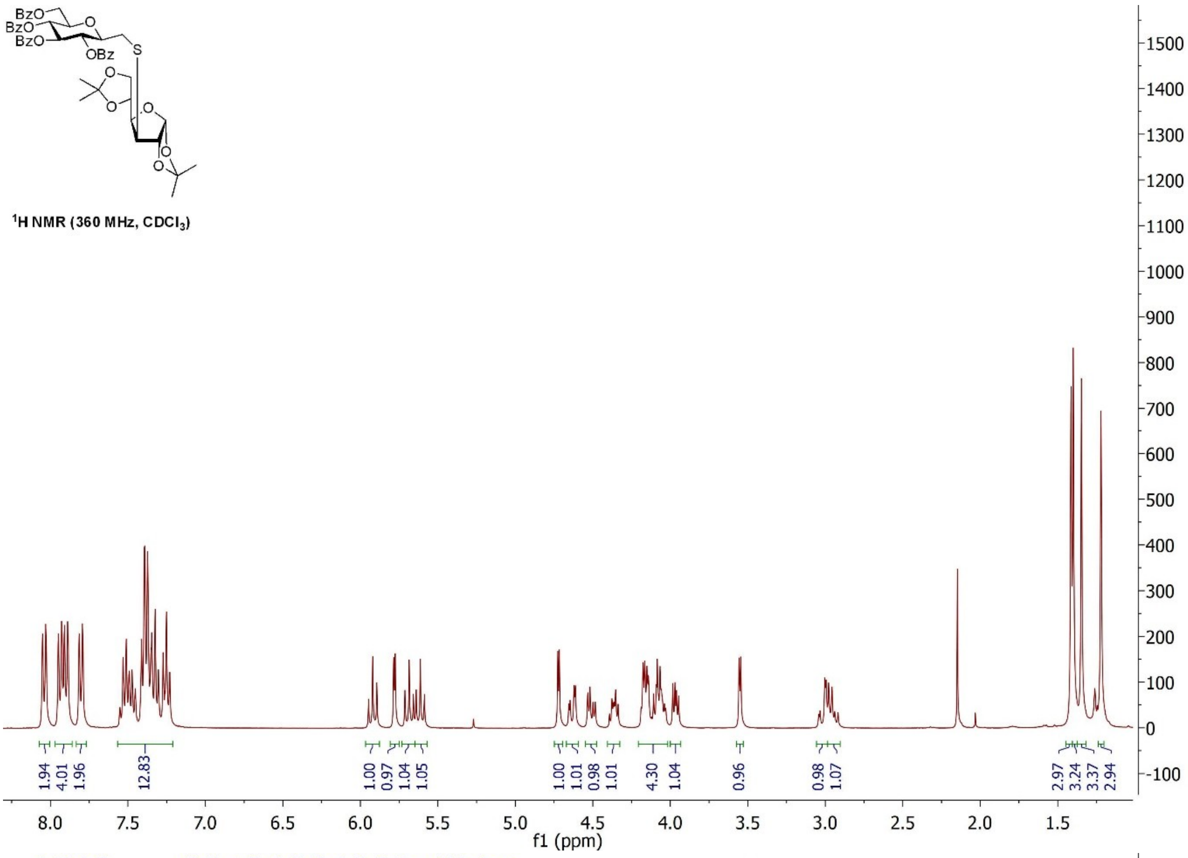

^{1}H NMR (360 MHz, CDCl$_3$)

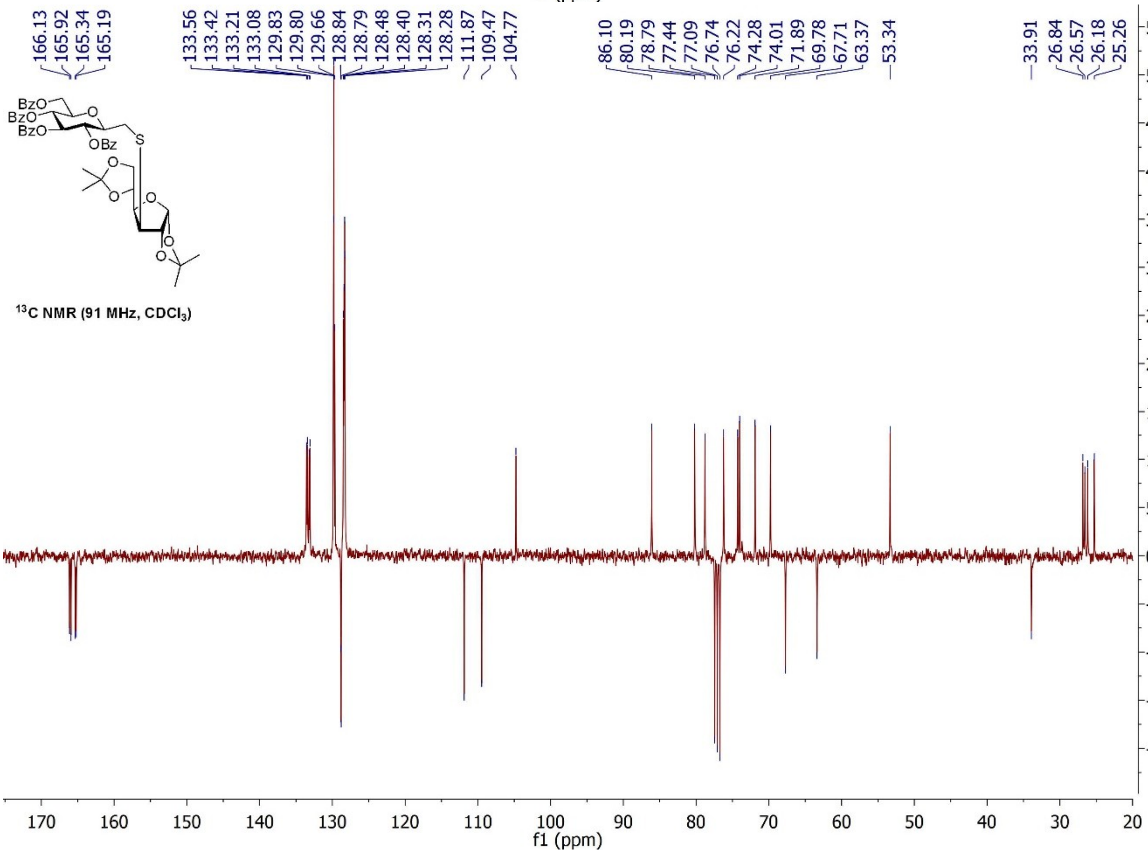

^{13}C NMR (91 MHz, CDCl$_3$)

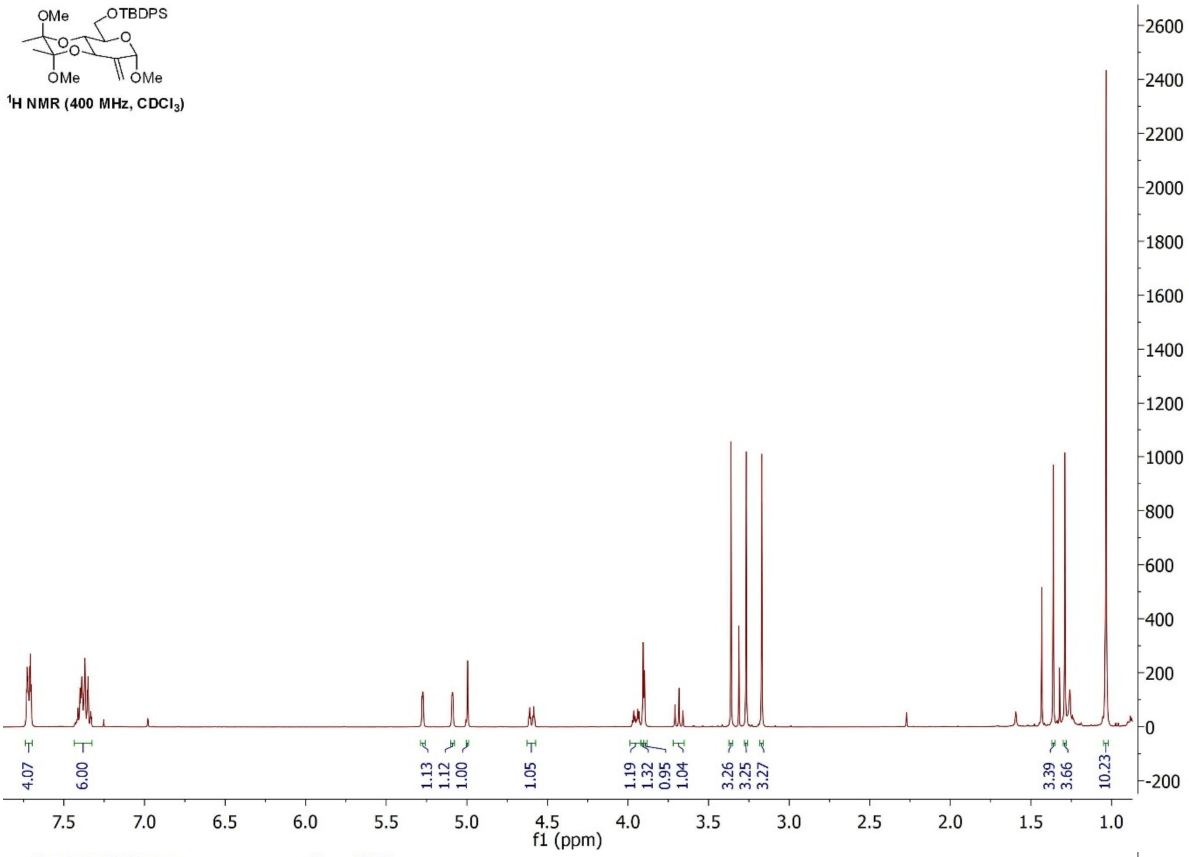
OMe
OTBDPS
O
O
OMe
OMe
^{1}H NMR (400 MHz, CDCl$_3$)
4.07
6.00
1.13
1.12
1.00
1.05
1.19
1.32
0.95
1.04
3.26
3.25
3.27
3.39
3.66
10.23
f1 (ppm)

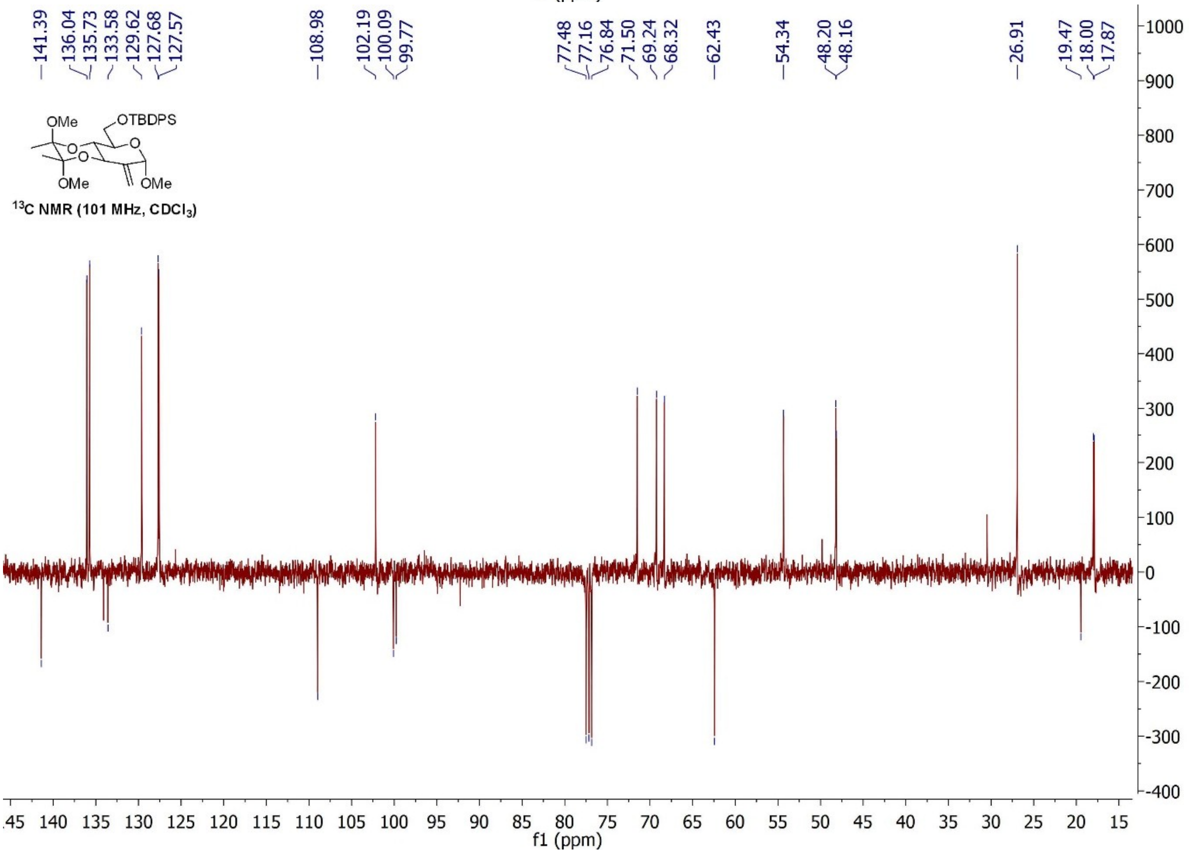
141.39
136.04
135.73
133.58
129.62
127.68
127.57
108.98
102.19
100.09
99.77
77.48
77.16
76.84
71.50
69.24
68.32
62.43
54.34
48.20
48.16
26.91
19.47
18.00
17.87
OMe
OTBDPS
O
O
OMe
OMe
^{13}C NMR (101 MHz, CDCl$_3$)
f1 (ppm)

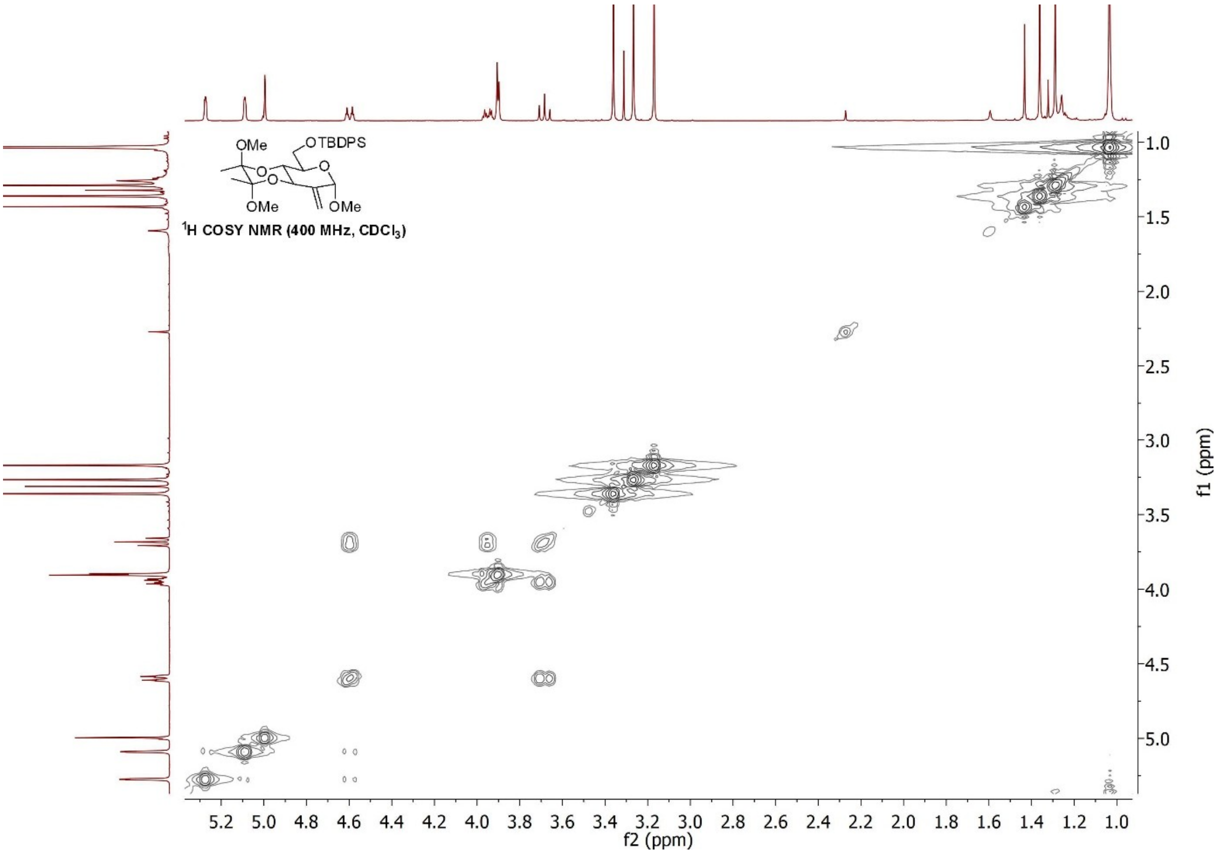
OMe
OTBDPS
OMe
OMe
¹H COSY NMR (400 MHz, CDCl₃)
f2 (ppm)
f1 (ppm)

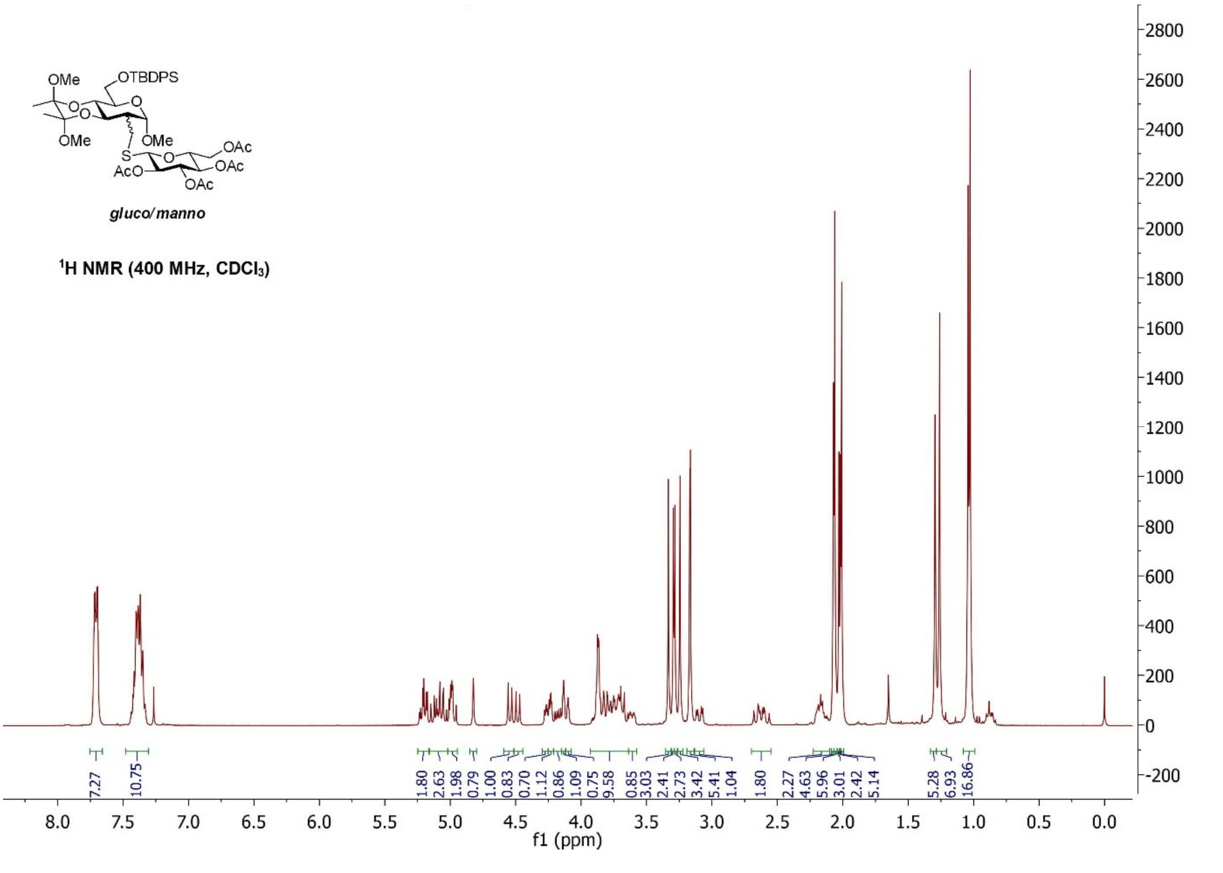
OMe
OTBDPS
OMe
OMe
OAc
OAc
AcO
OAc
gluco/manno
¹H NMR (400 MHz, CDCl₃)
f1 (ppm)

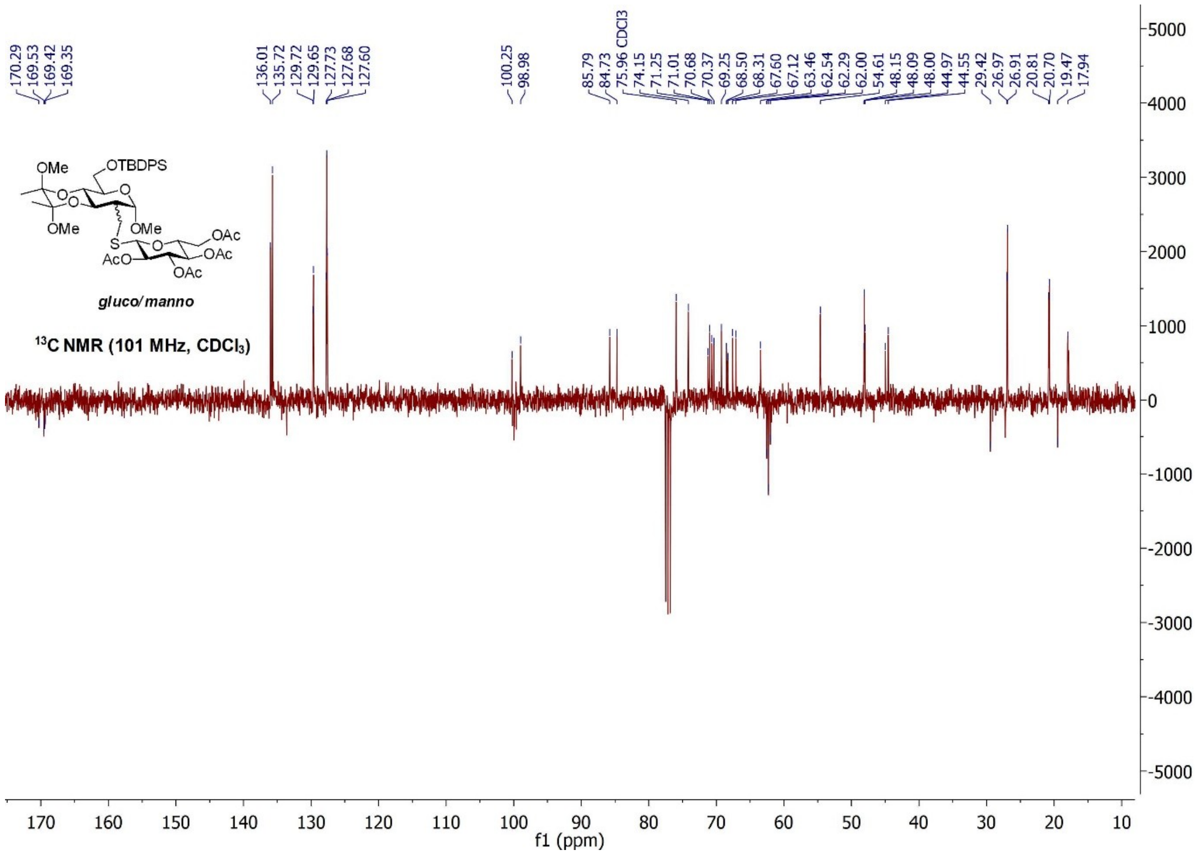

170.29
169.53
169.42
169.35
136.01
135.72
129.72
129.65
127.73
127.68
127.60
100.25
98.98
85.79
84.73
75.96 CDCl3
74.15
71.25
71.01
70.68
70.37
69.25
68.50
68.31
67.60
67.12
63.46
62.54
62.29
62.00
54.61
48.15
48.09
48.00
44.97
44.55
29.42
26.97
26.91
20.81
20.70
19.47
17.94
OMe
OTBDPS
OMe
OMe
OMe
S
OAc
OAc
AcO
OAc
gluco/manno
13C NMR (101 MHz, CDCl3)
f1 (ppm)

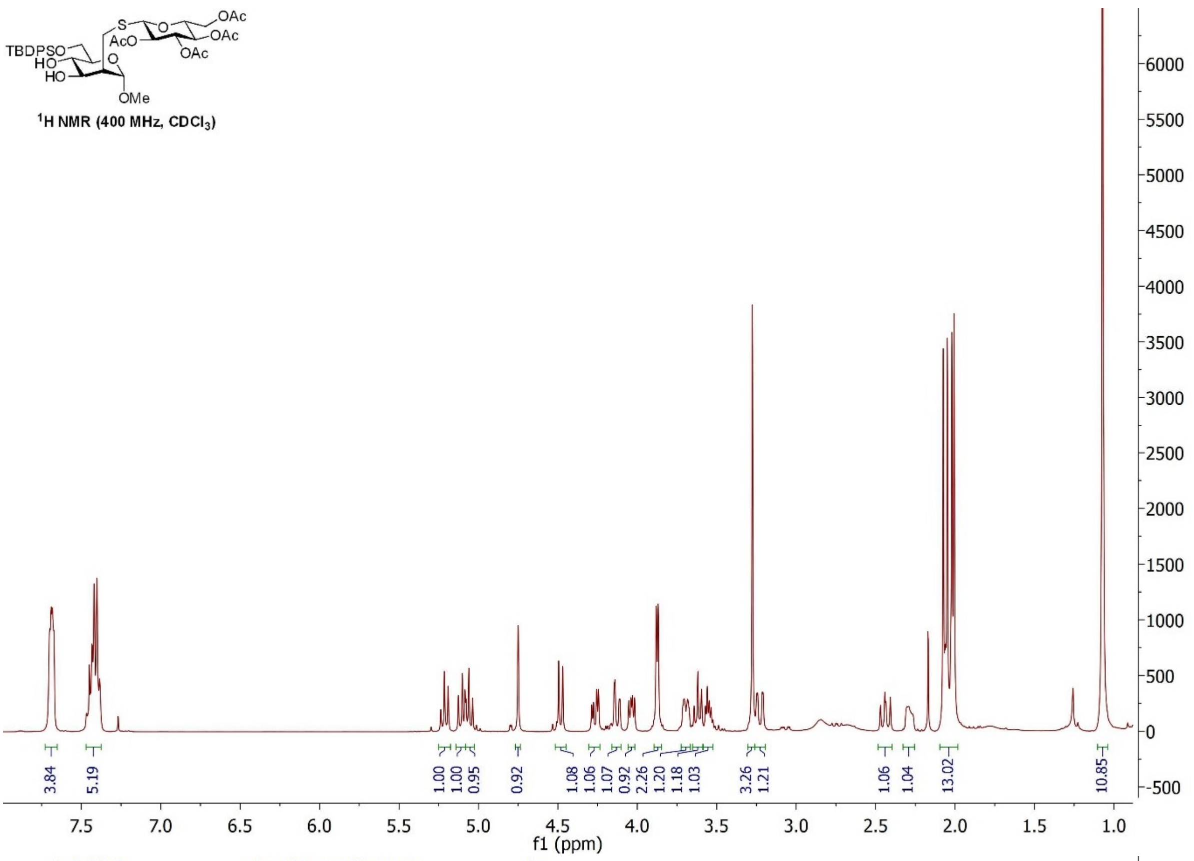
TBDPSO
HO
HO
S
AcO
O
OAc
OAc
OAc
OMe
^{1}H NMR (400 MHz, CDCl$_3$)
3.84
5.19
1.00
1.00
0.95
0.92
1.08
1.06
1.07
0.92
2.26
1.20
1.18
1.03
3.26
1.21
1.06
1.04
13.02
10.85
f1 (ppm)

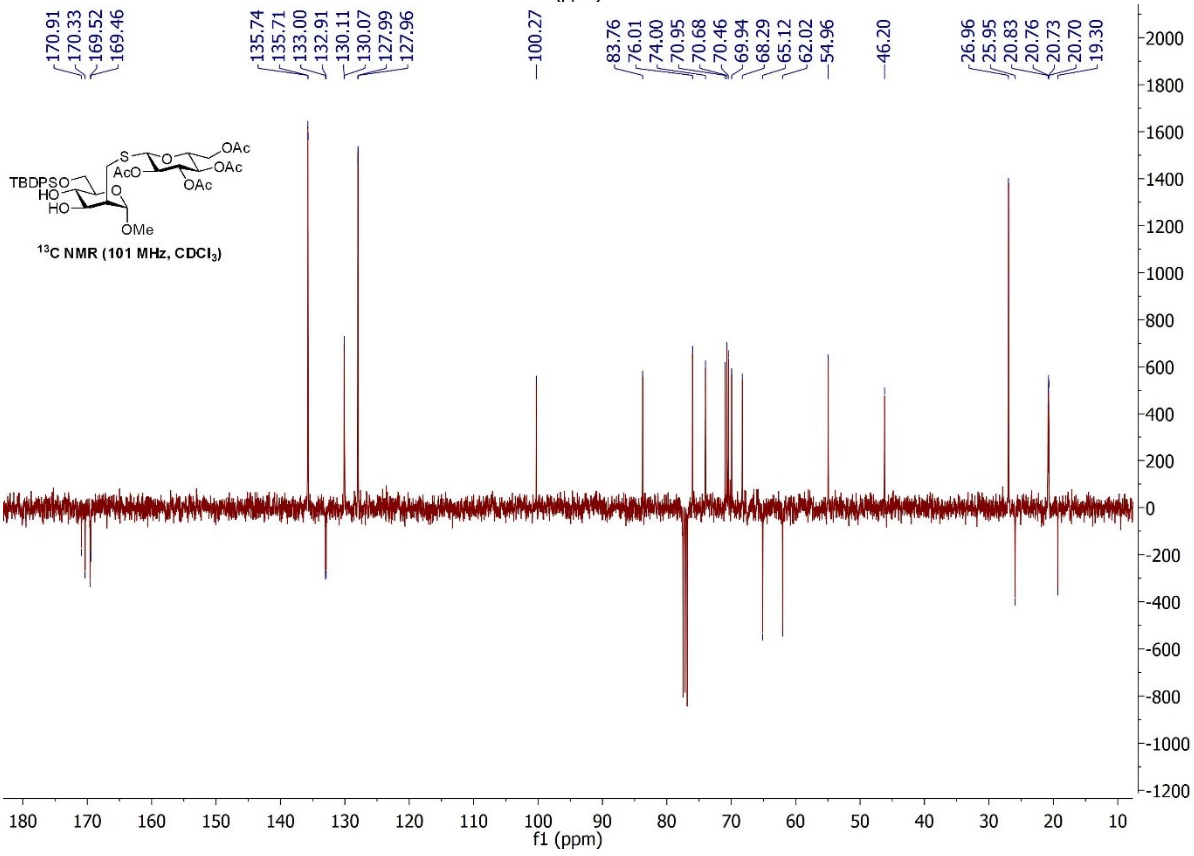
170.91
170.33
169.52
169.46
135.74
135.71
133.00
132.91
130.11
130.07
127.99
127.96
100.27
83.76
76.01
74.00
70.95
70.68
70.46
69.94
68.29
65.12
62.02
54.96
46.20
26.96
25.95
20.83
20.76
20.73
20.70
19.30
TBDPSO
HO
HO
S
AcO
O
OAc
OAc
OAc
OMe
^{13}C NMR (101 MHz, CDCl$_3$)
f1 (ppm)

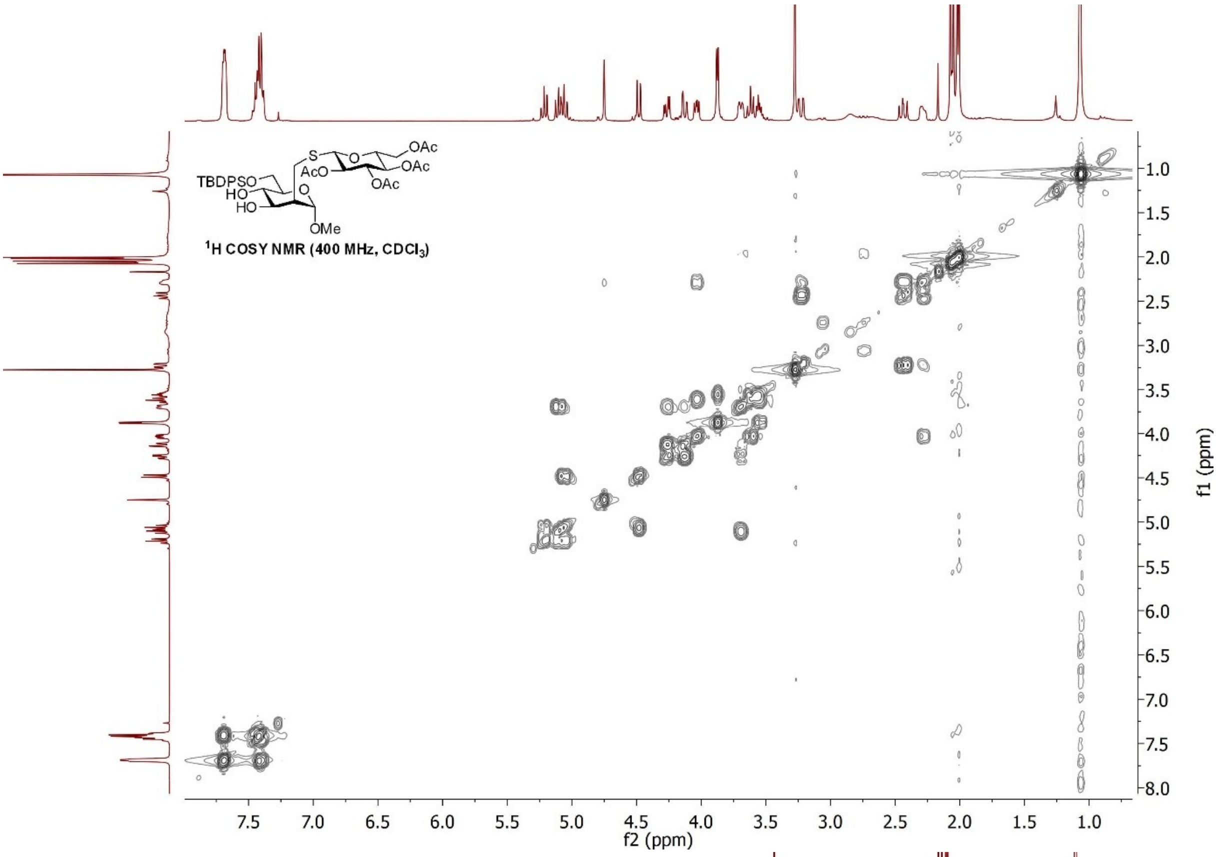

TBDPSO
HO
HO
S
AcO
O
OAc
OAc
OAc
OMe
^{1}H COSY NMR (400 MHz, $CDCl_3$)
f2 (ppm)
f1 (ppm)

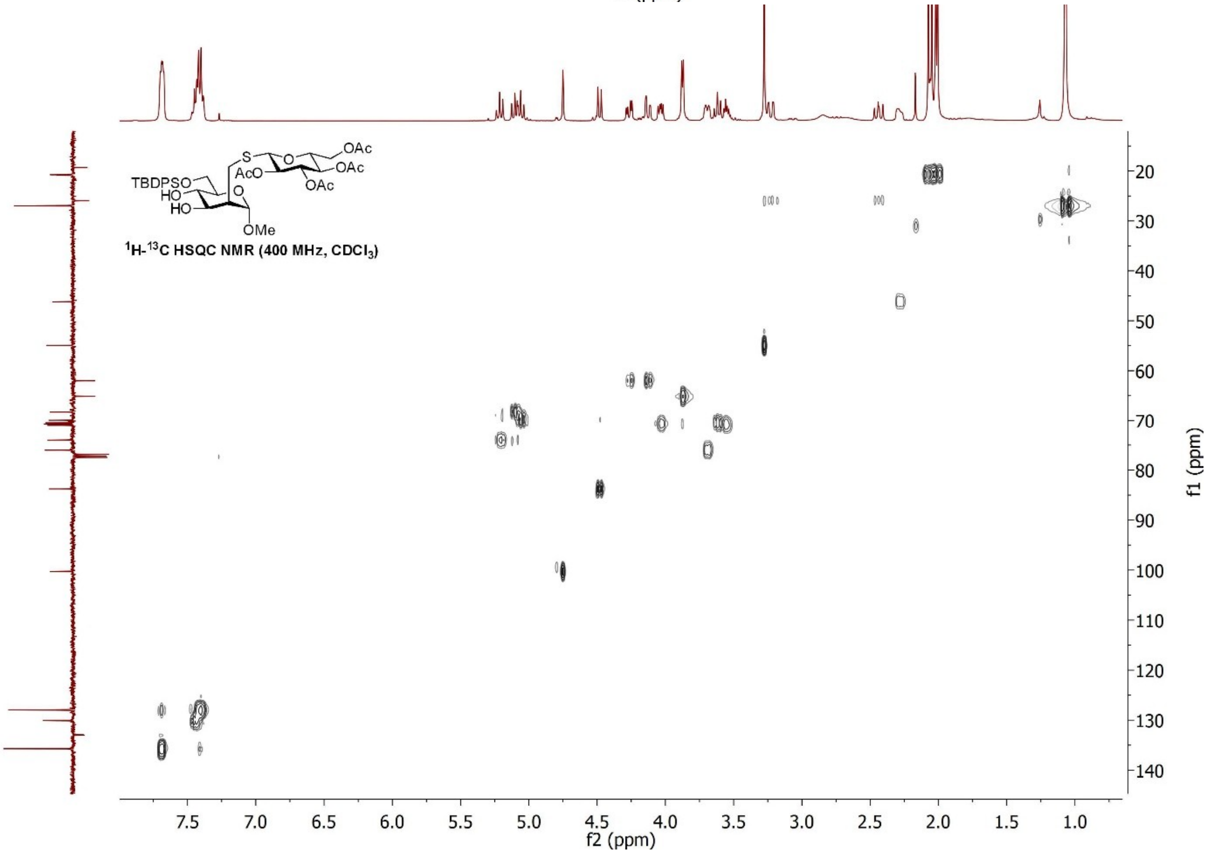

TBDPSO
HO
HO
S
AcO
O
OAc
OAc
OAc
OMe
^{1}H-^{13}C HSQC NMR (400 MHz, $CDCl_3$)
f2 (ppm)
f1 (ppm)

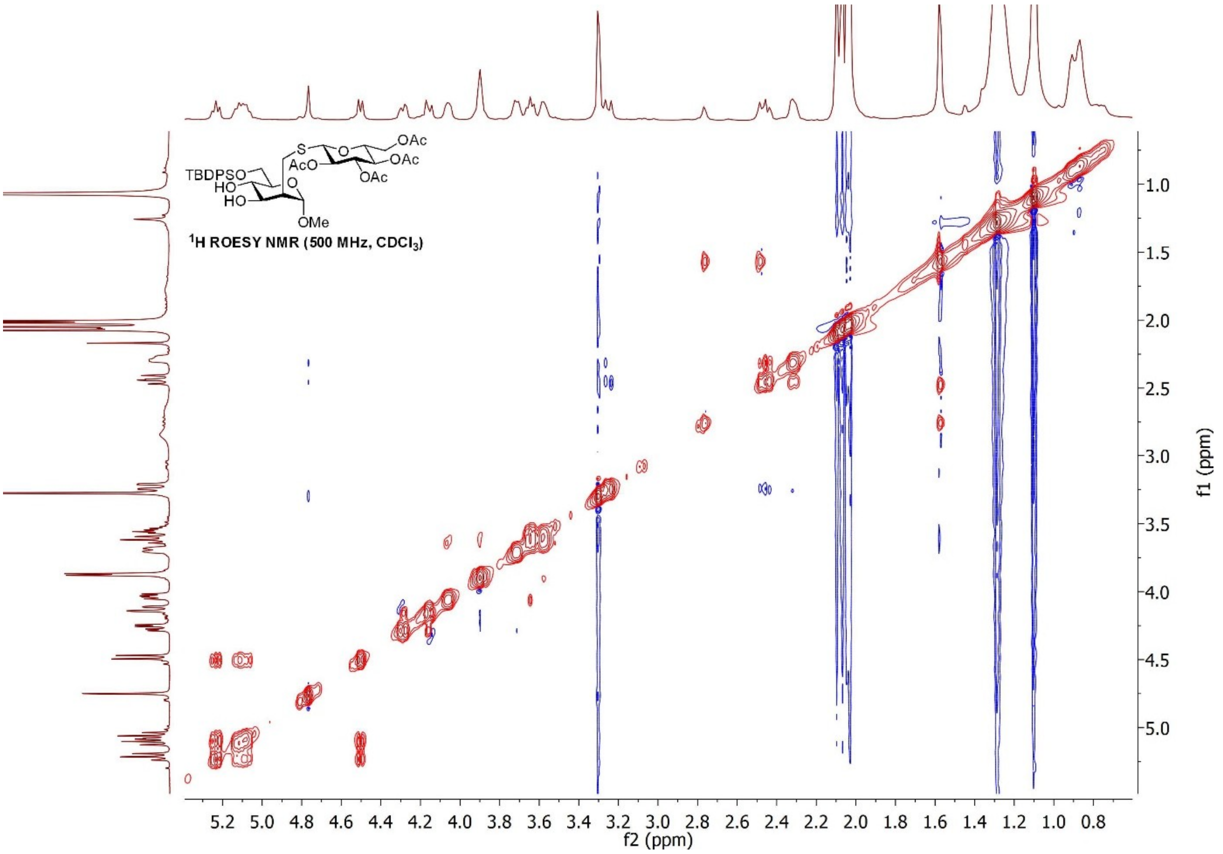
TBDPSO
HO
HO
S
AcO
OAc
OAc
OAc
O
OMe
^{1}H ROESY NMR (500 MHz, CDCl$_3$)
f2 (ppm)
f1 (ppm)

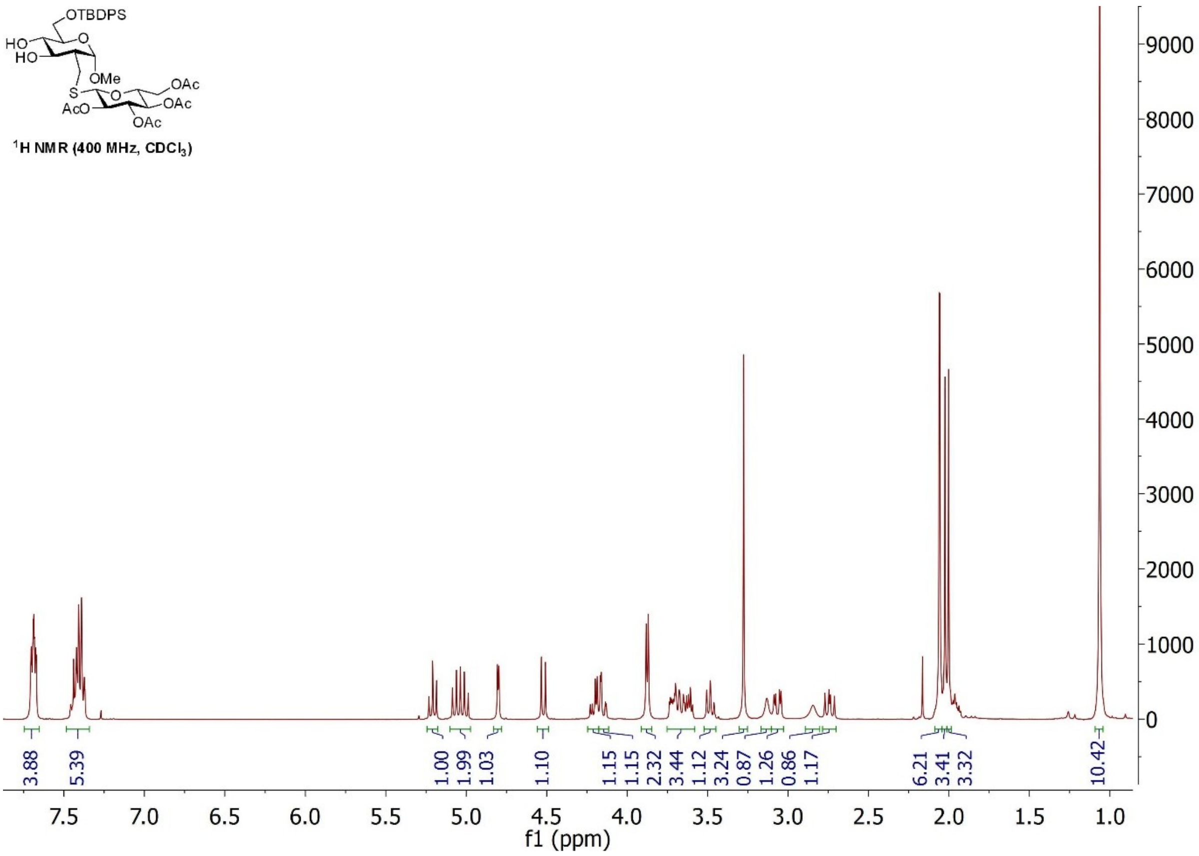
OTBDPS
HO
HO
OMe
S
AcO
OAc
OAc
OAc
^{1}H NMR (400 MHz, CDCl$_3$)
f1 (ppm)

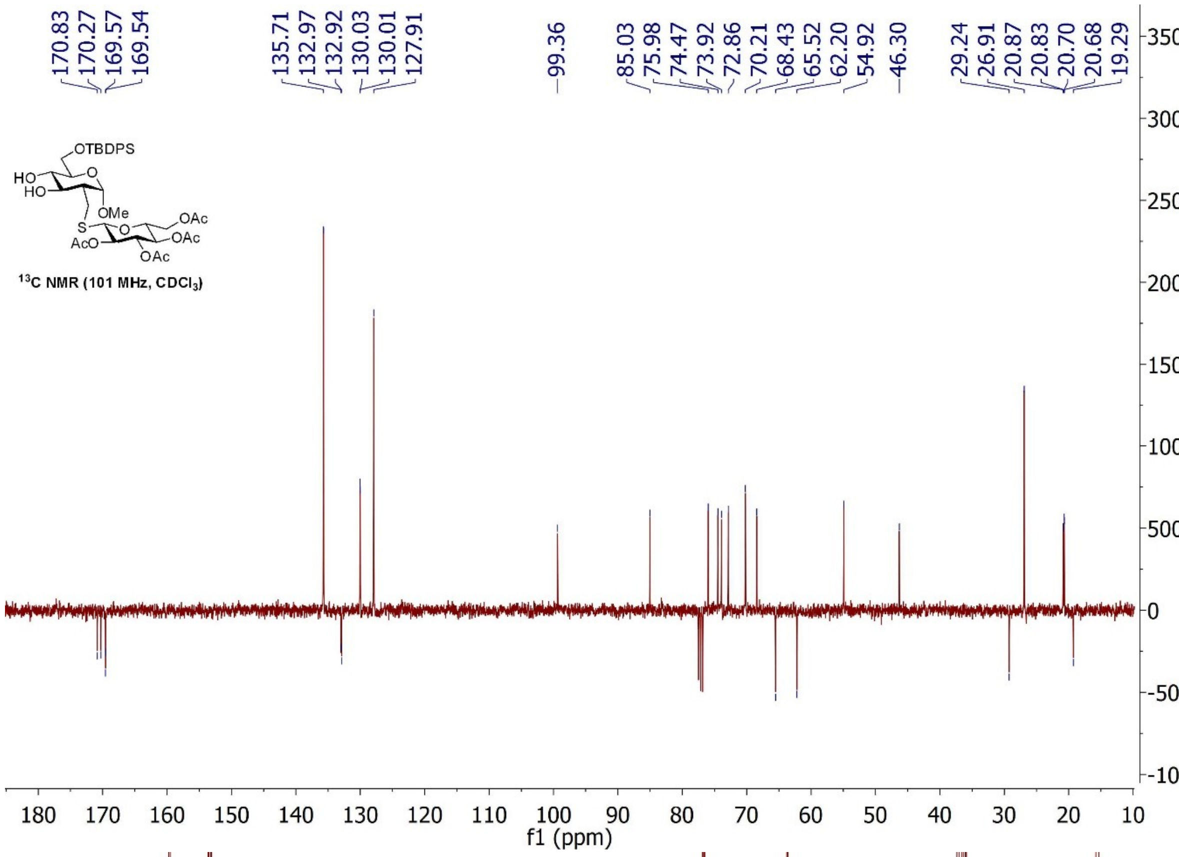

170.83
170.27
169.57
169.54
135.71
132.97
132.92
130.03
130.01
127.91
99.36
85.03
75.98
74.47
73.92
72.86
70.21
68.43
65.52
62.20
54.92
46.30
29.24
26.91
20.87
20.83
20.70
20.68
19.29
OTBDPS
HO
HO
O
S
OMe
O
OAc
OAc
AcO
OAc
13C NMR (101 MHz, CDCl3)
3500
3000
2500
2000
1500
1000
500
0
-500
-1000
180 170 160 150 140 130 120 110 100 90 80 70 60 50 40 30 20 10
f1 (ppm)

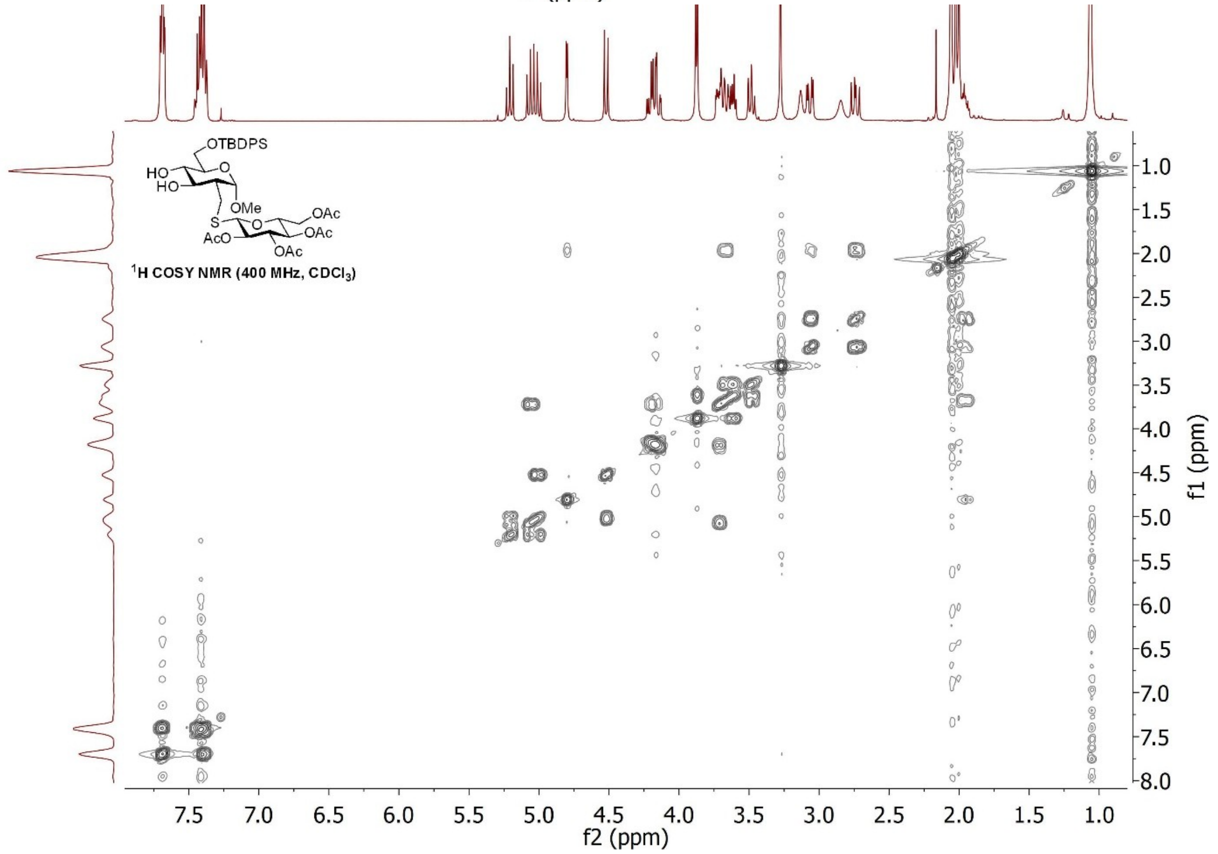

OTBDPS
HO
HO
O
S
OMe
O
OAc
OAc
AcO
OAc
1H COSY NMR (400 MHz, CDCl3)
1.0 1.5 2.0 2.5 3.0 3.5 4.0 4.5 5.0 5.5 6.0 6.5 7.0 7.5 8.0
f1 (ppm)
7.5 7.0 6.5 6.0 5.5 5.0 4.5 4.0 3.5 3.0 2.5 2.0 1.5 1.0
f2 (ppm)

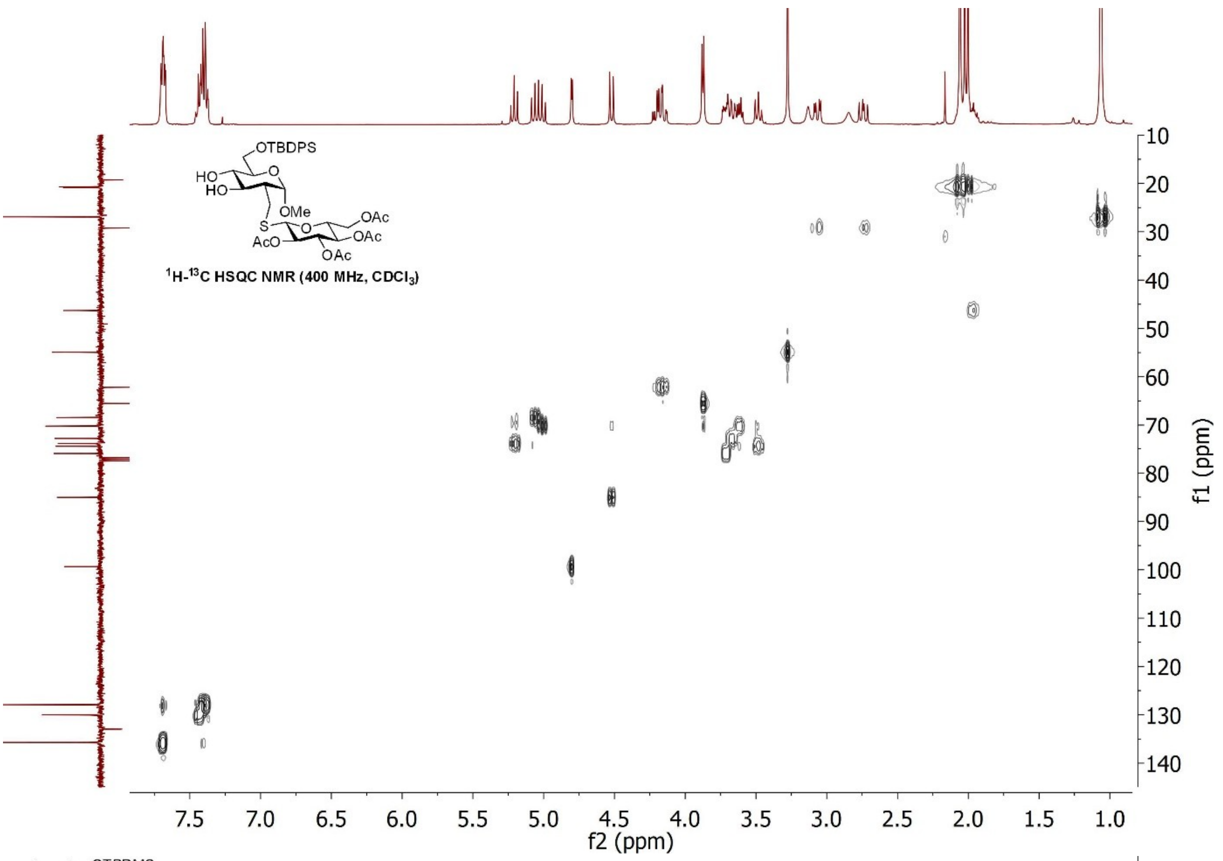
OTBDPS
HO
HO
OMe
S
OAc
AcO
OAc
OAc
¹H-¹³C HSQC NMR (400 MHz, CDCl₃)
f2 (ppm)
f1 (ppm)

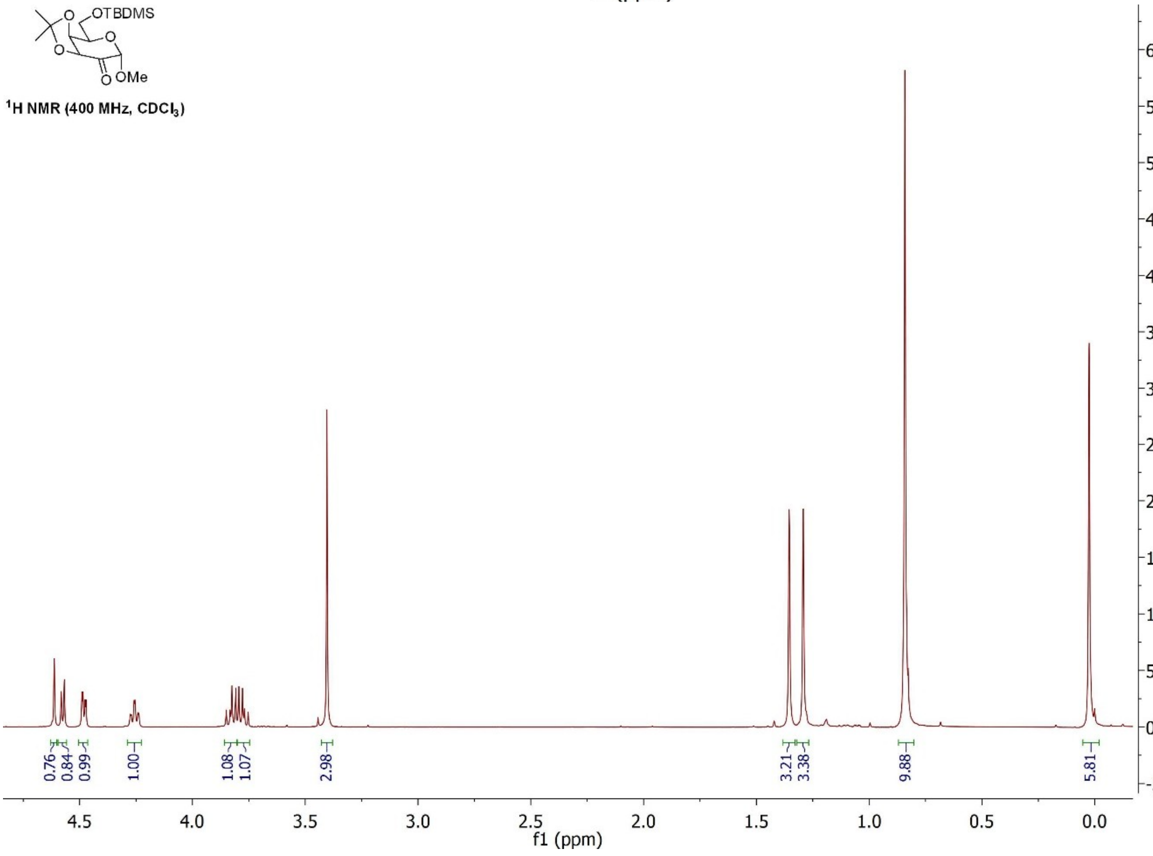
OTBDMS
OMe
¹H NMR (400 MHz, CDCl₃)
0.76
0.84
0.99
1.00
1.08
1.07
2.98
3.21
3.38
9.88
5.81
f1 (ppm)

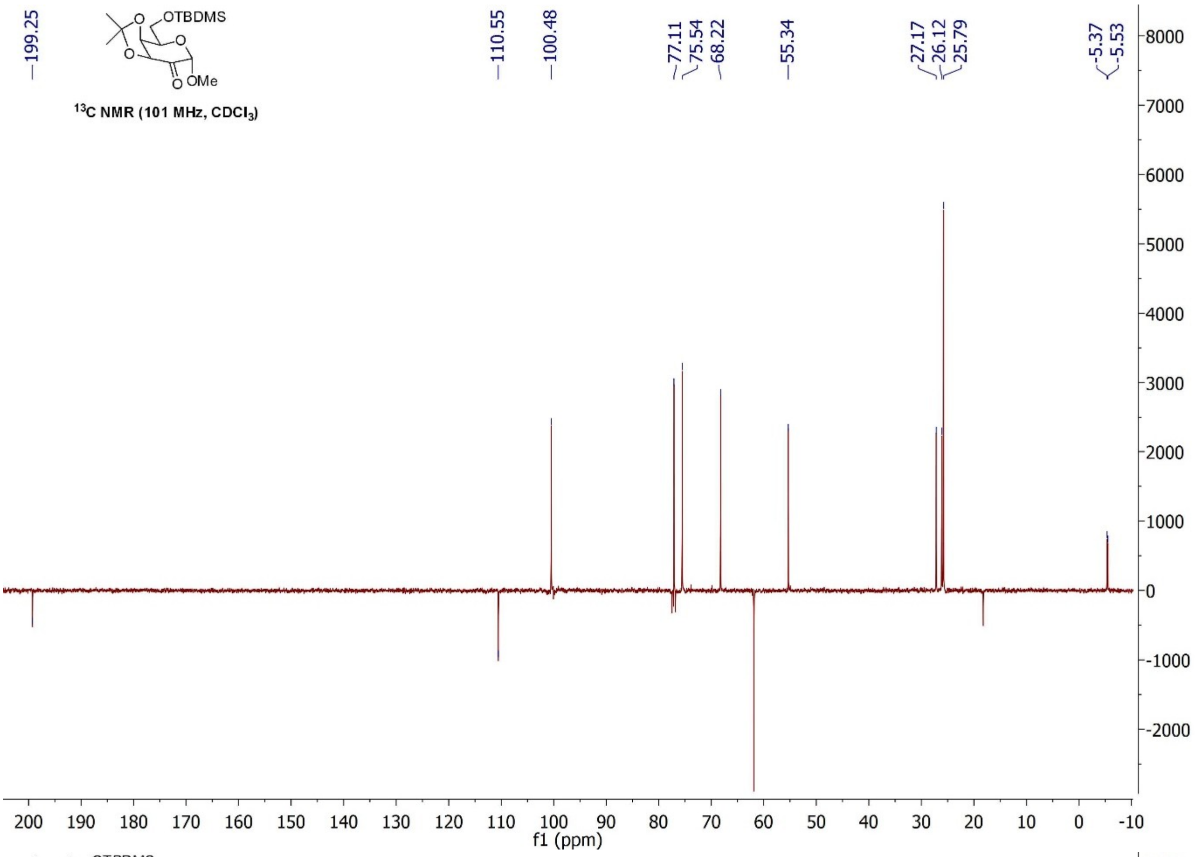

OTBDMS
OMe
^{13}C NMR (101 MHz, CDCl$_3$)
199.25
110.55
100.48
77.11
75.54
68.22
55.34
27.17
26.12
25.79
-5.37
-5.53
f1 (ppm)

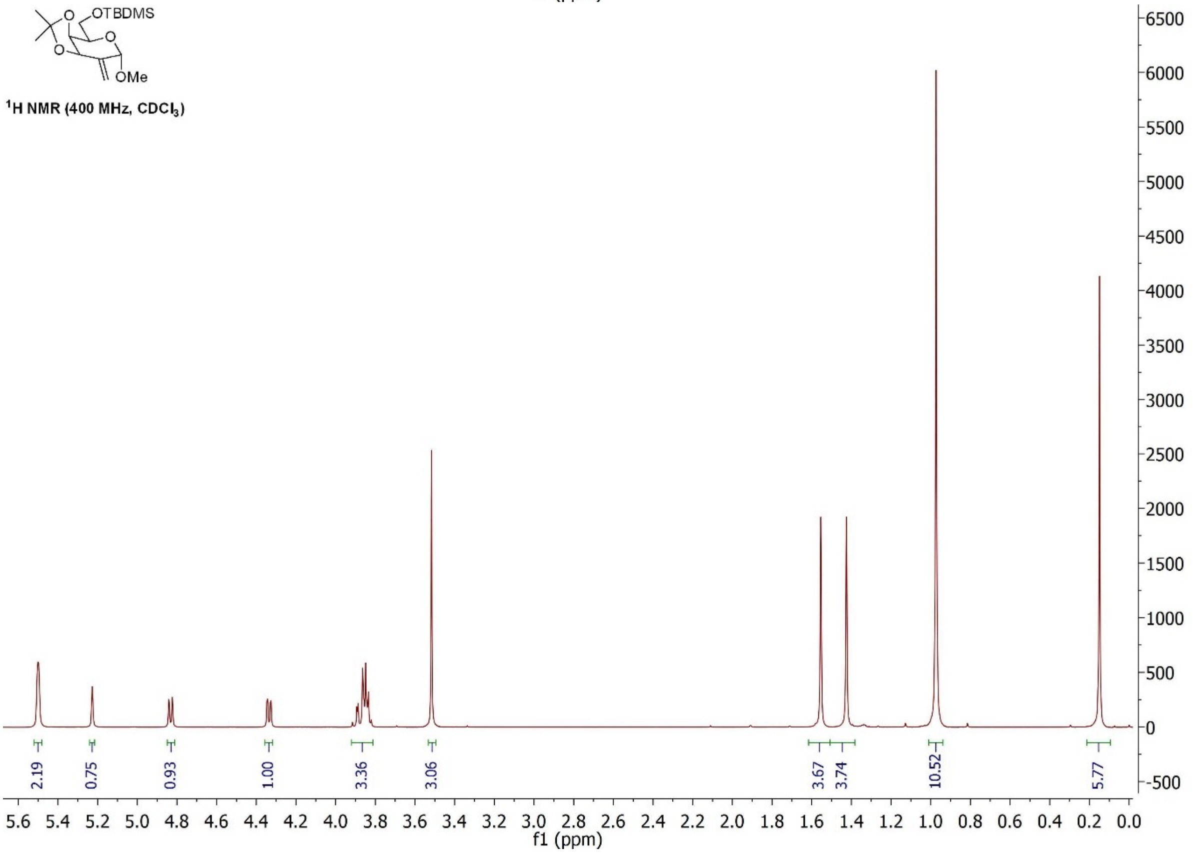

OTBDMS
OMe
^{1}H NMR (400 MHz, CDCl$_3$)
2.19
0.75
0.93
1.00
3.36
3.06
3.67
3.74
10.52
5.77
f1 (ppm)

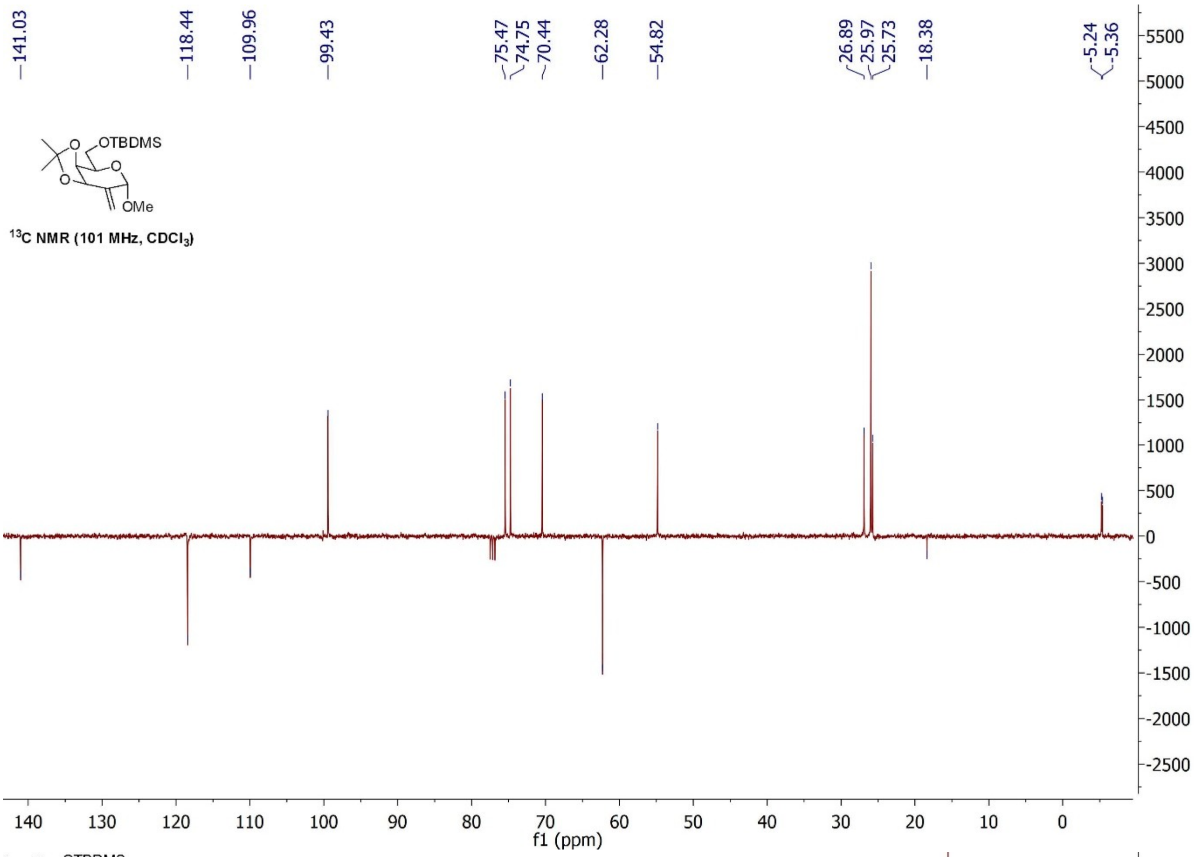
141.03
118.44
109.96
99.43
75.47
74.75
70.44
62.28
54.82
26.89
25.97
25.73
18.38
-5.24
-5.36
OTBDMS
OMe
^{13}C NMR (101 MHz, $CDCl_3$)
f1 (ppm)

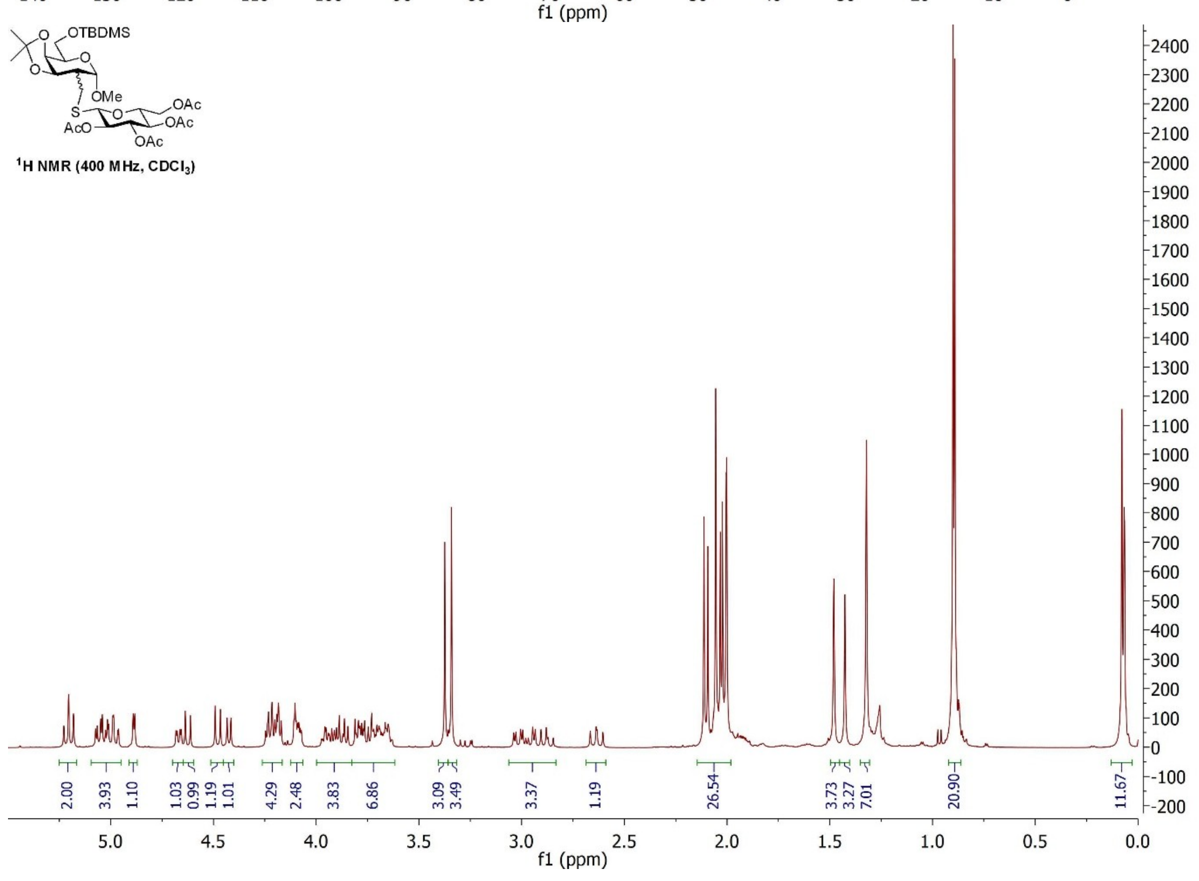
OTBDMS
OMe
OAc
AcO
^{1}H NMR (400 MHz, $CDCl_3$)
2.00
3.93
1.10
1.03
0.99
1.19
1.01
4.29
2.48
3.83
6.86
3.09
3.49
3.37
1.19
26.54
3.73
3.27
7.01
20.90
11.67
f1 (ppm)

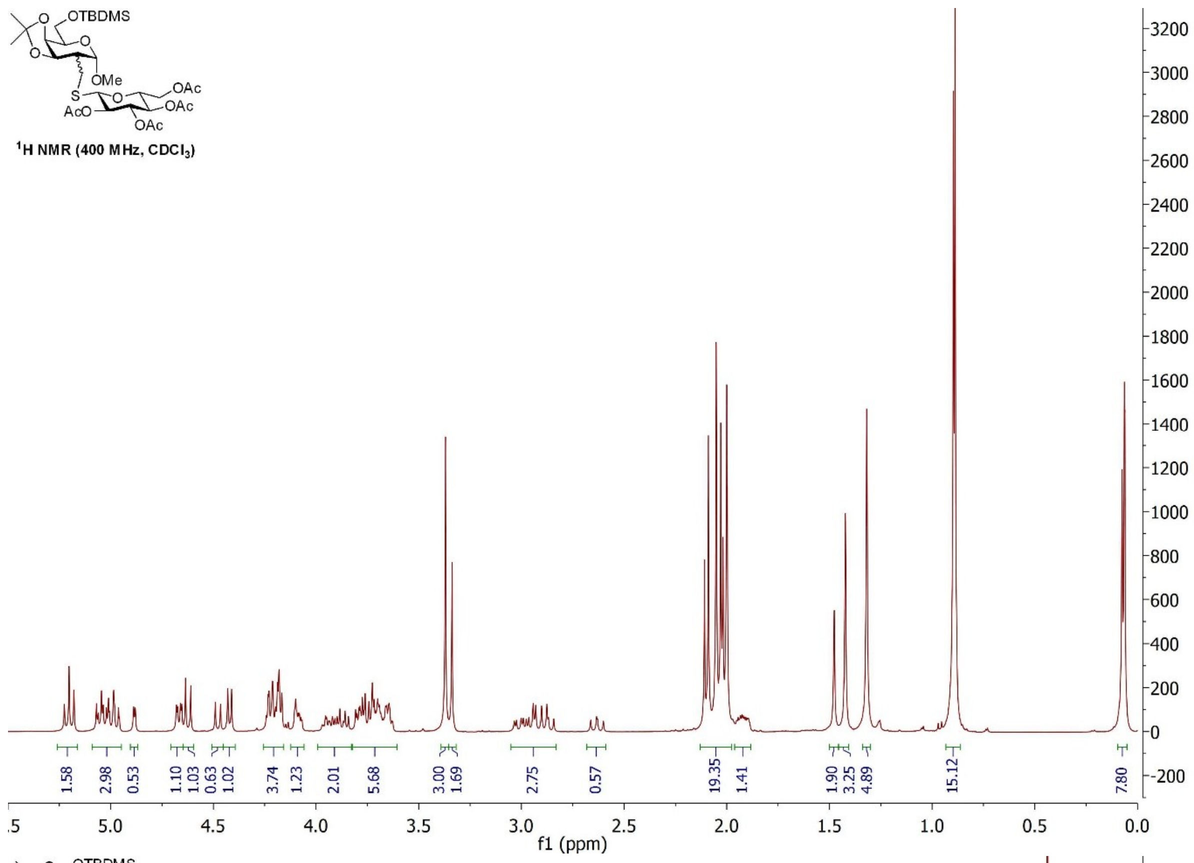
OTBDMS
OMe
S
OAc
AcO
^{1}H NMR (400 MHz, $CDCl_3$)
1.58
2.98
0.53
1.10
1.03
0.63
1.02
3.74
1.23
2.01
5.68
3.00
1.69
2.75
0.57
19.35
1.41
1.90
3.25
4.89
15.12
7.80
f1 (ppm)

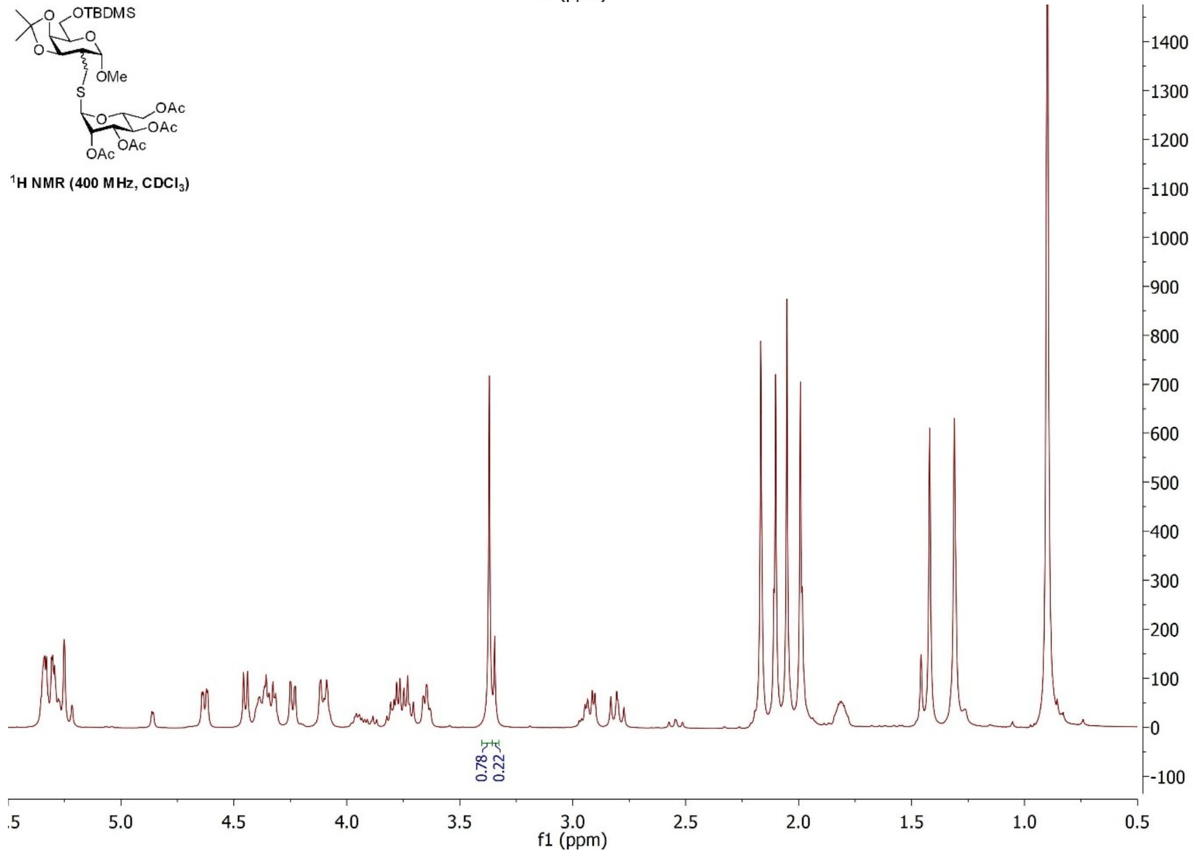
OTBDMS
OMe
S
OAc
^{1}H NMR (400 MHz, $CDCl_3$)
0.78
0.22
f1 (ppm)

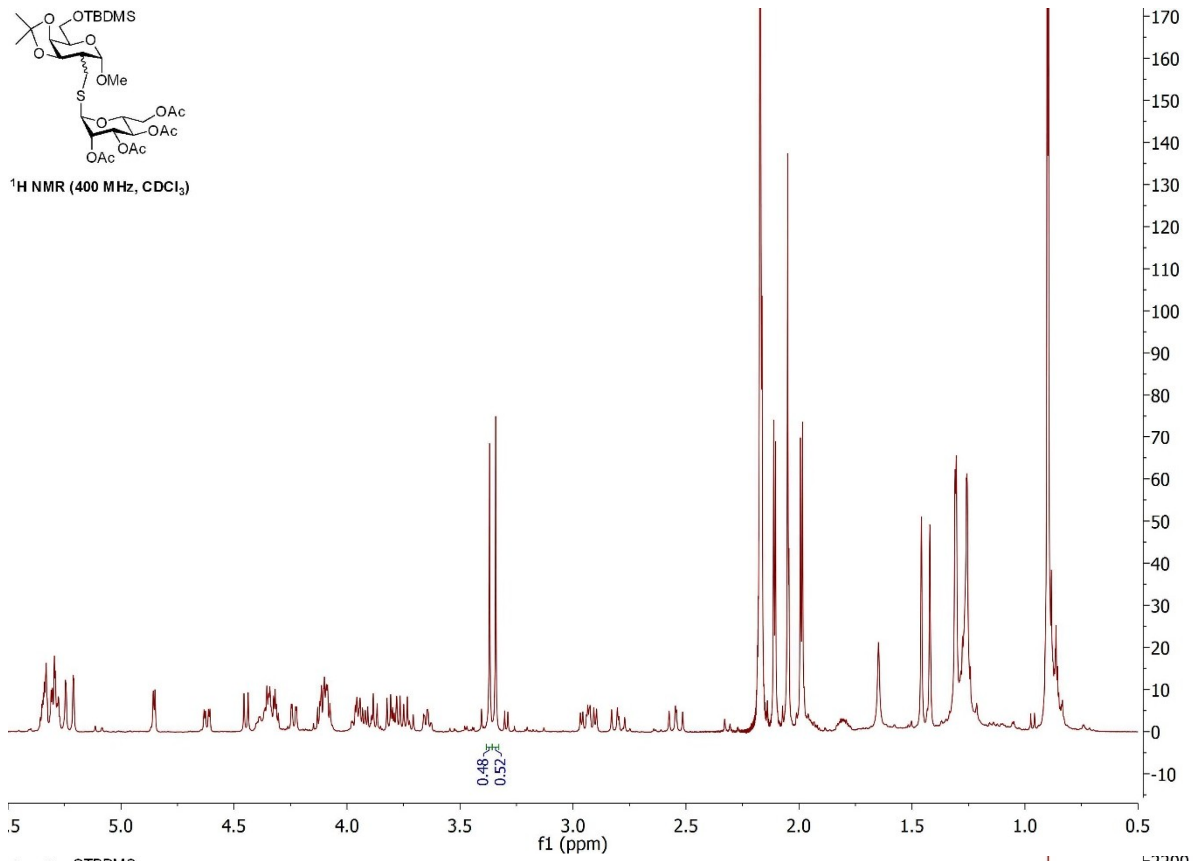
OTBDMS
OMe
S
OAc
OAc
OAc
OAc
¹H NMR (400 MHz, CDCl₃)
0.48
0.52
f1 (ppm)

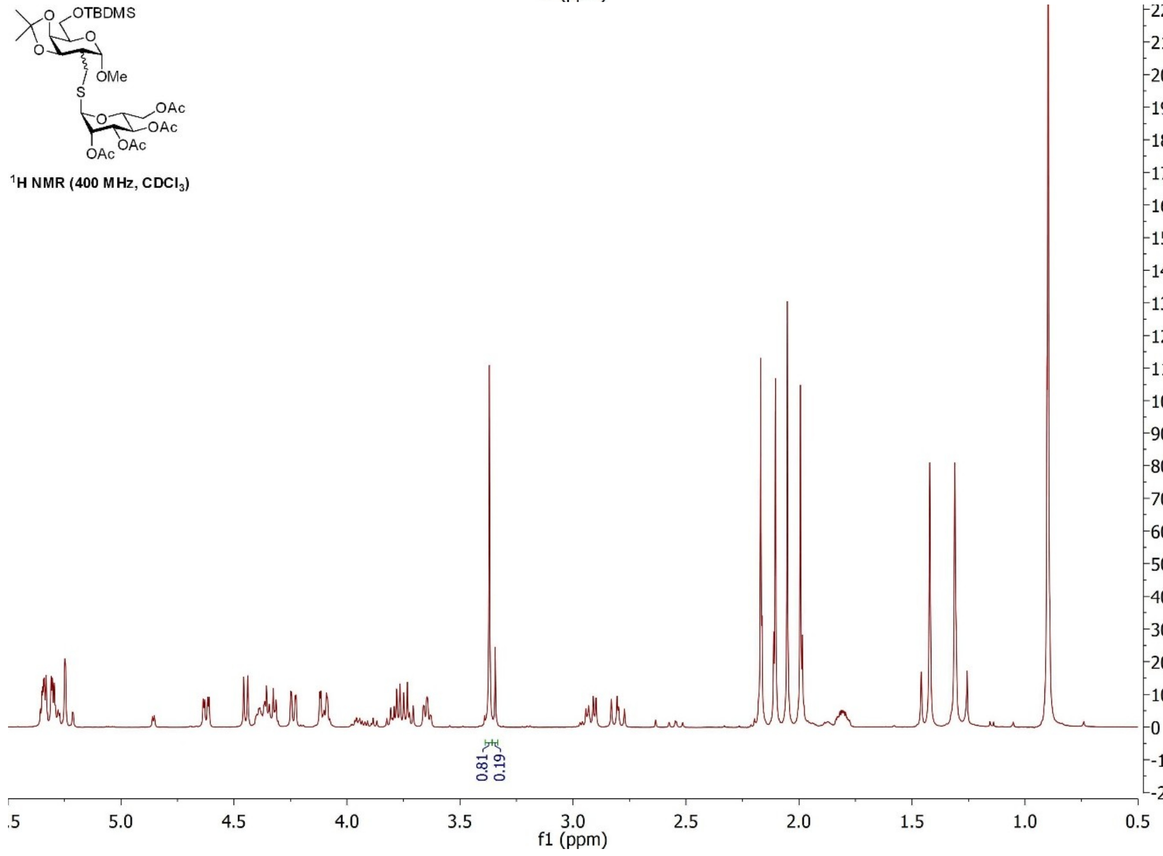
OTBDMS
OMe
S
OAc
OAc
OAc
OAc
¹H NMR (400 MHz, CDCl₃)
0.81
0.19
f1 (ppm)

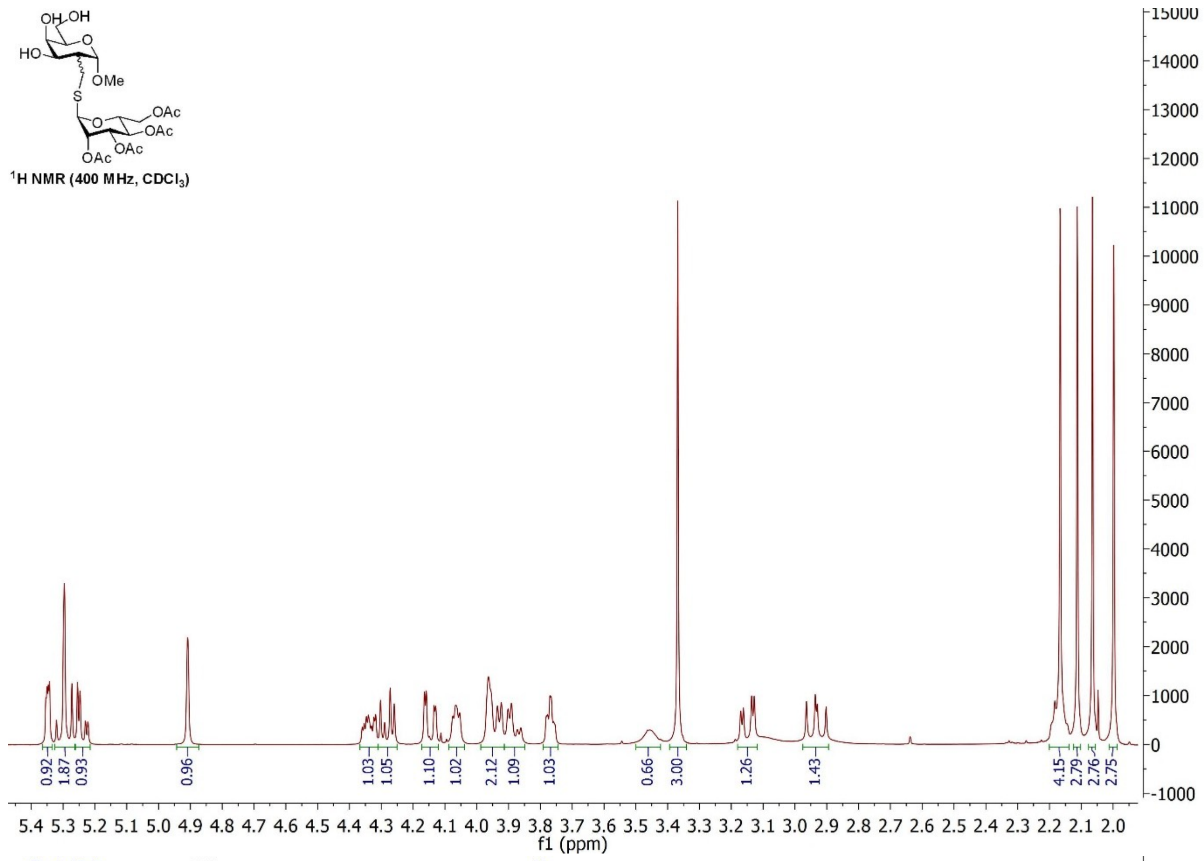

OAc
OMe
S
¹H NMR (400 MHz, CDCl3)
0.92
1.87
0.93
0.96
1.03
1.05
1.10
1.02
2.12
1.09
1.03
0.66
3.00
1.26
1.43
4.15
2.79
2.76
2.75
f1 (ppm)

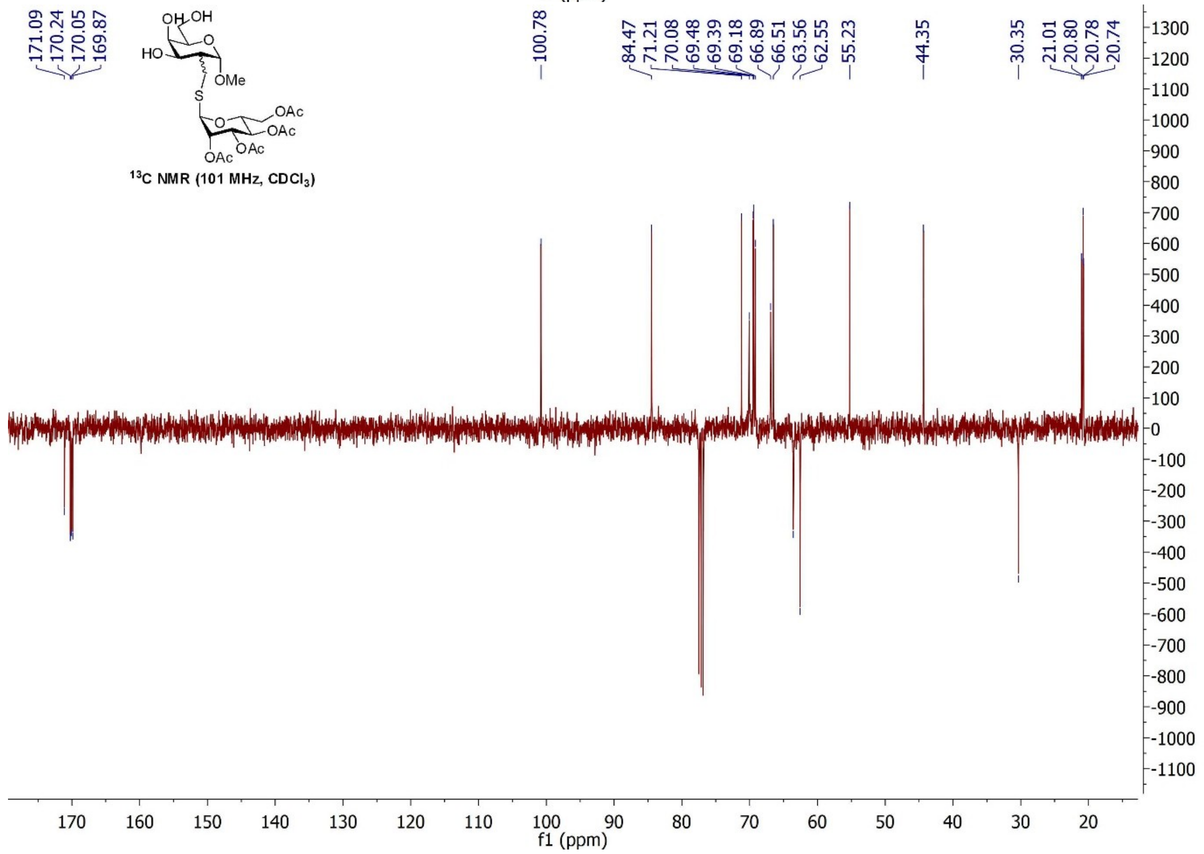

171.09
170.24
170.05
169.87
100.78
84.47
71.21
70.08
69.48
69.39
69.18
66.89
66.51
63.56
62.55
55.23
44.35
30.35
21.01
20.80
20.78
20.74
OAc
OMe
S
¹³C NMR (101 MHz, CDCl3)
f1 (ppm)

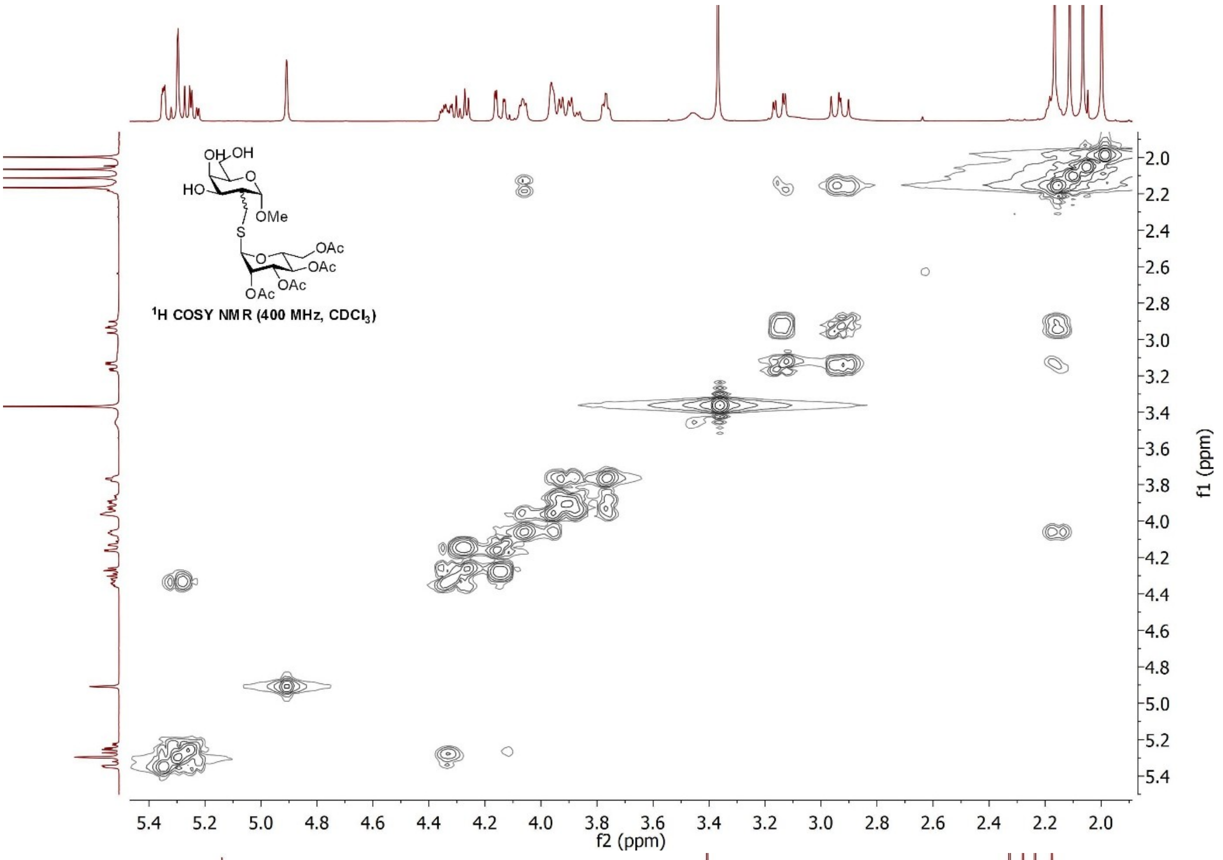
OH OH
HO
OMe
S
OAc
OAc
OAc
OAc
1H COSY NMR (400 MHz, CDCl3)
f2 (ppm)
f1 (ppm)

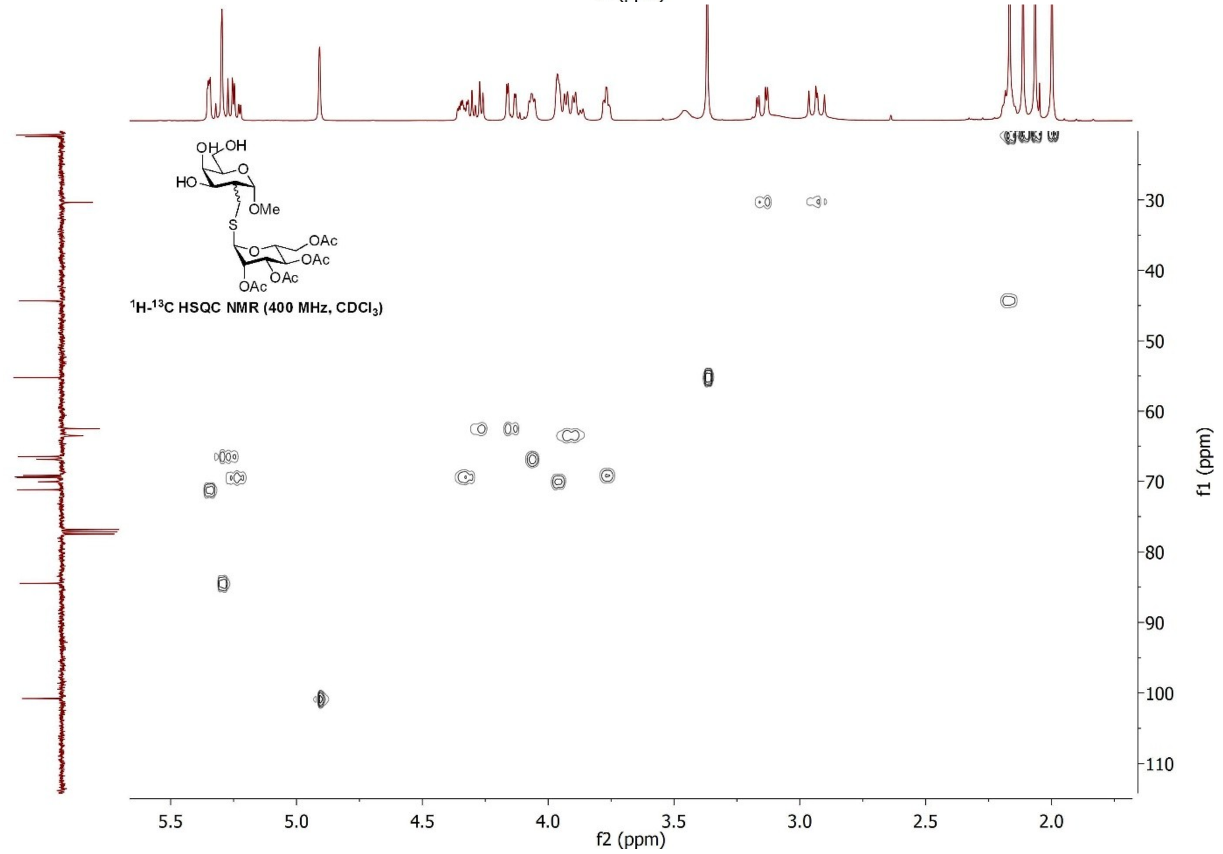
OH OH
HO
OMe
S
OAc
OAc
OAc
OAc
1H-13C HSQC NMR (400 MHz, CDCl3)
f2 (ppm)
f1 (ppm)

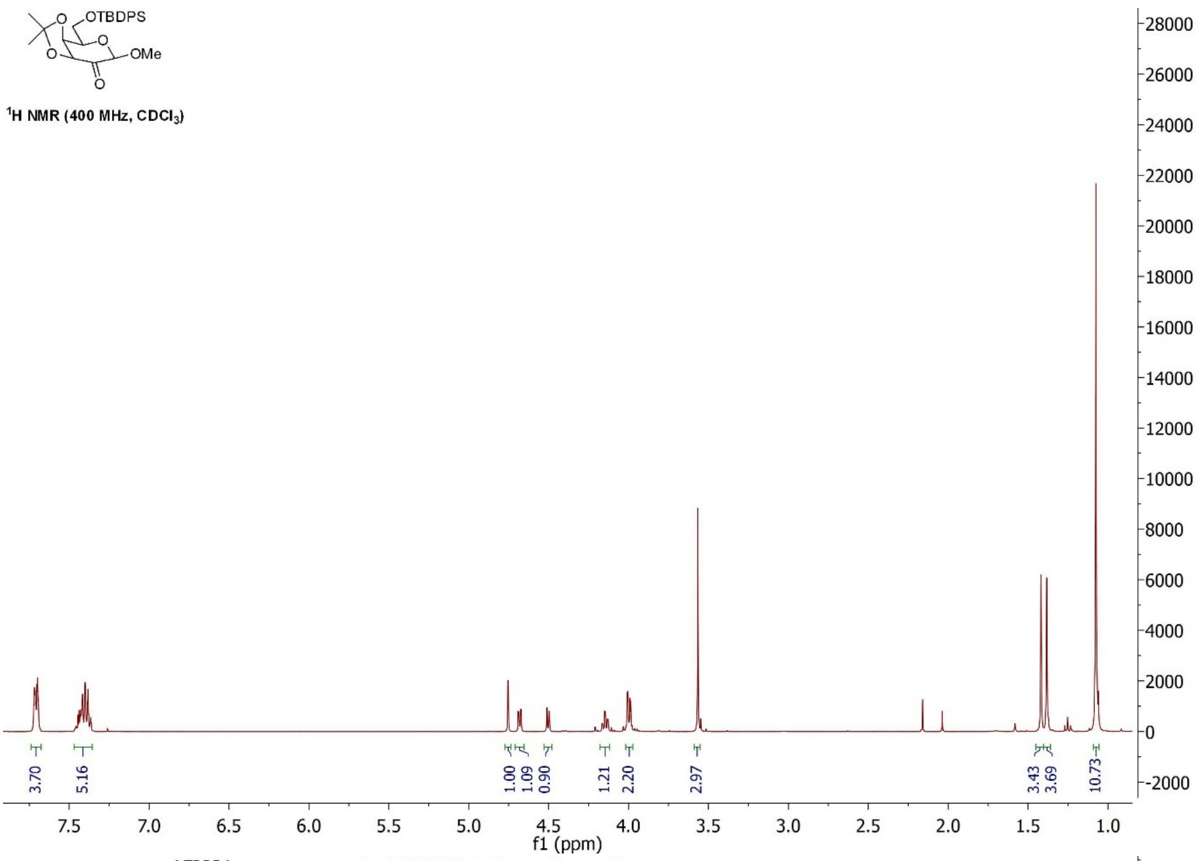

^{1}H NMR (400 MHz, $CDCl_3$)

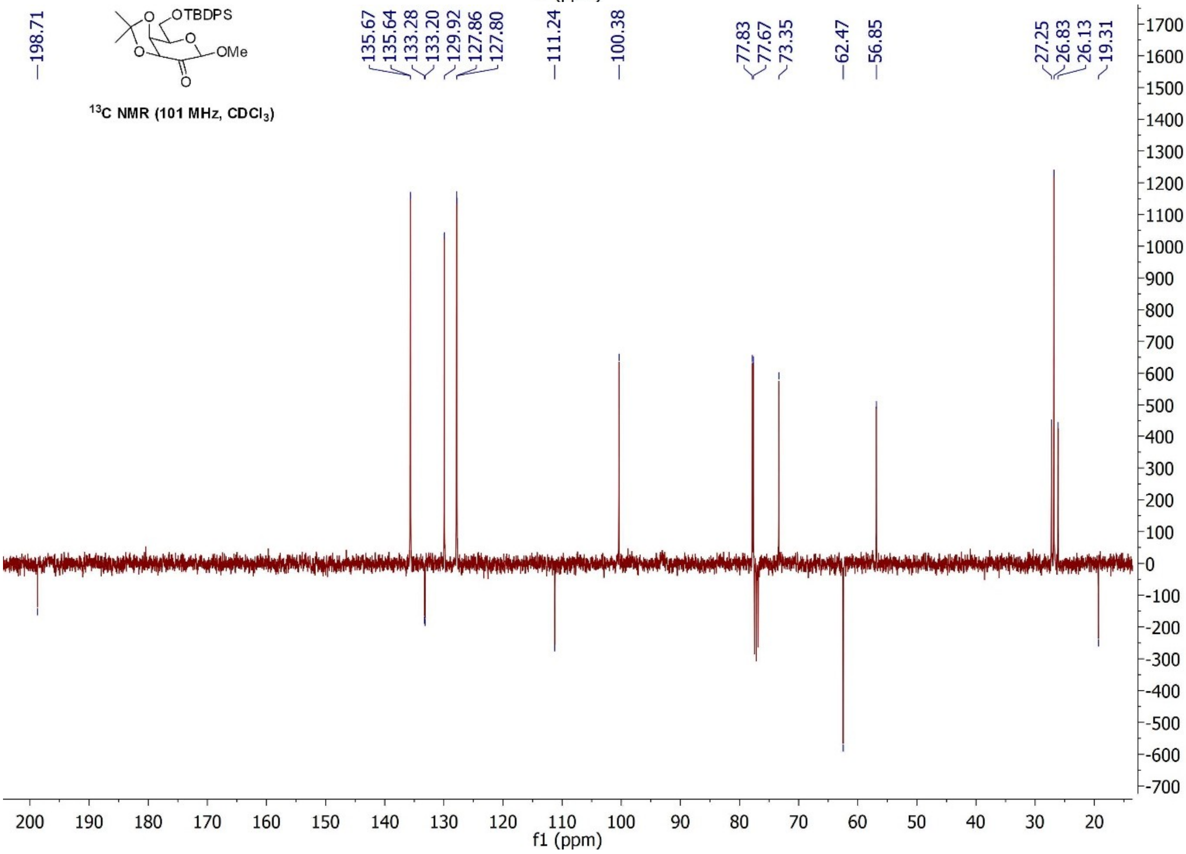

^{13}C NMR (101 MHz, $CDCl_3$)

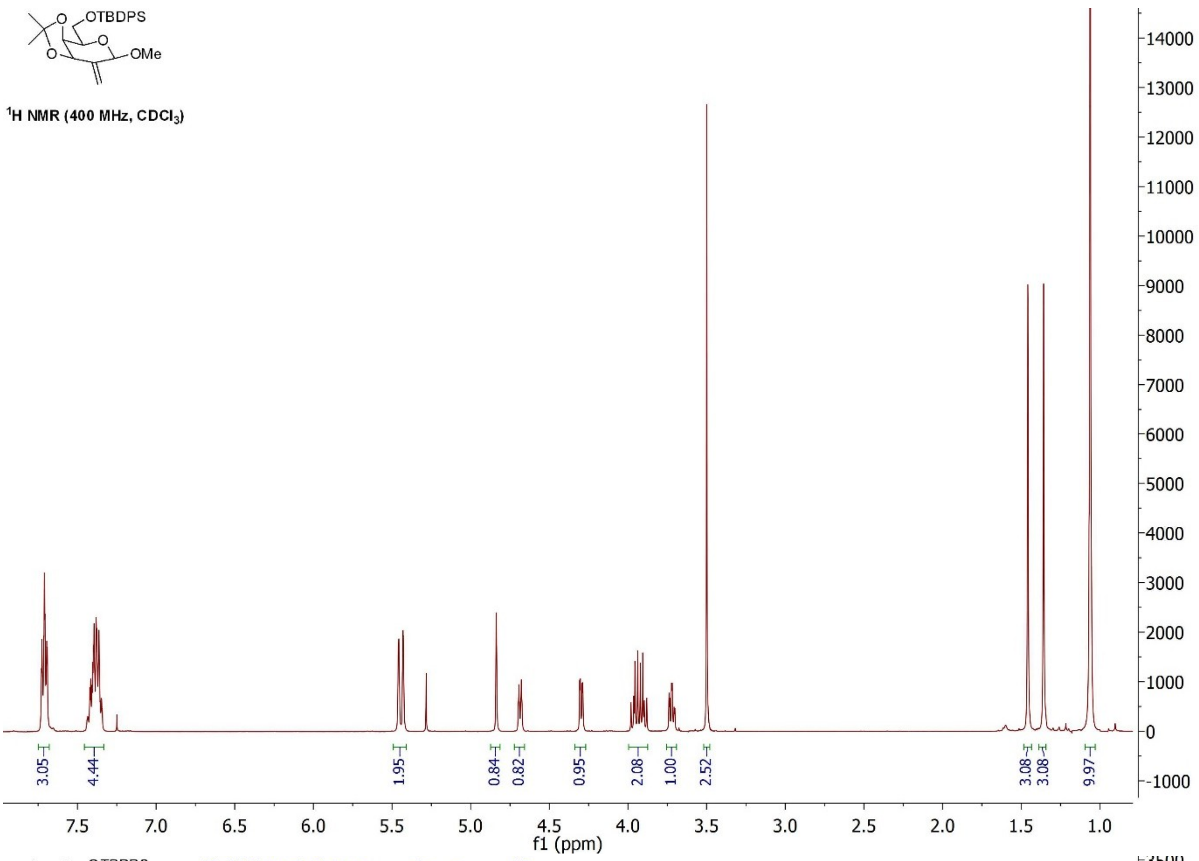

^{1}H NMR (400 MHz, $CDCl_3$)

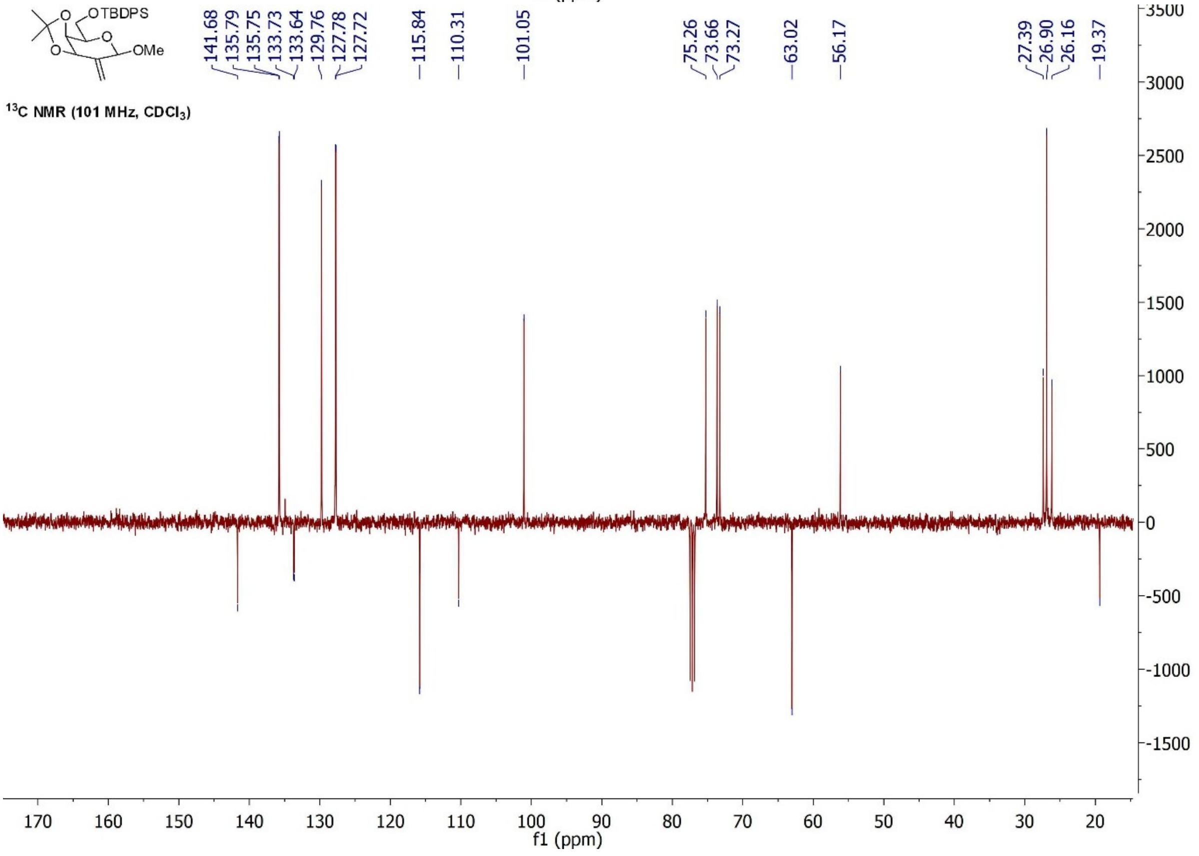

^{13}C NMR (101 MHz, $CDCl_3$)

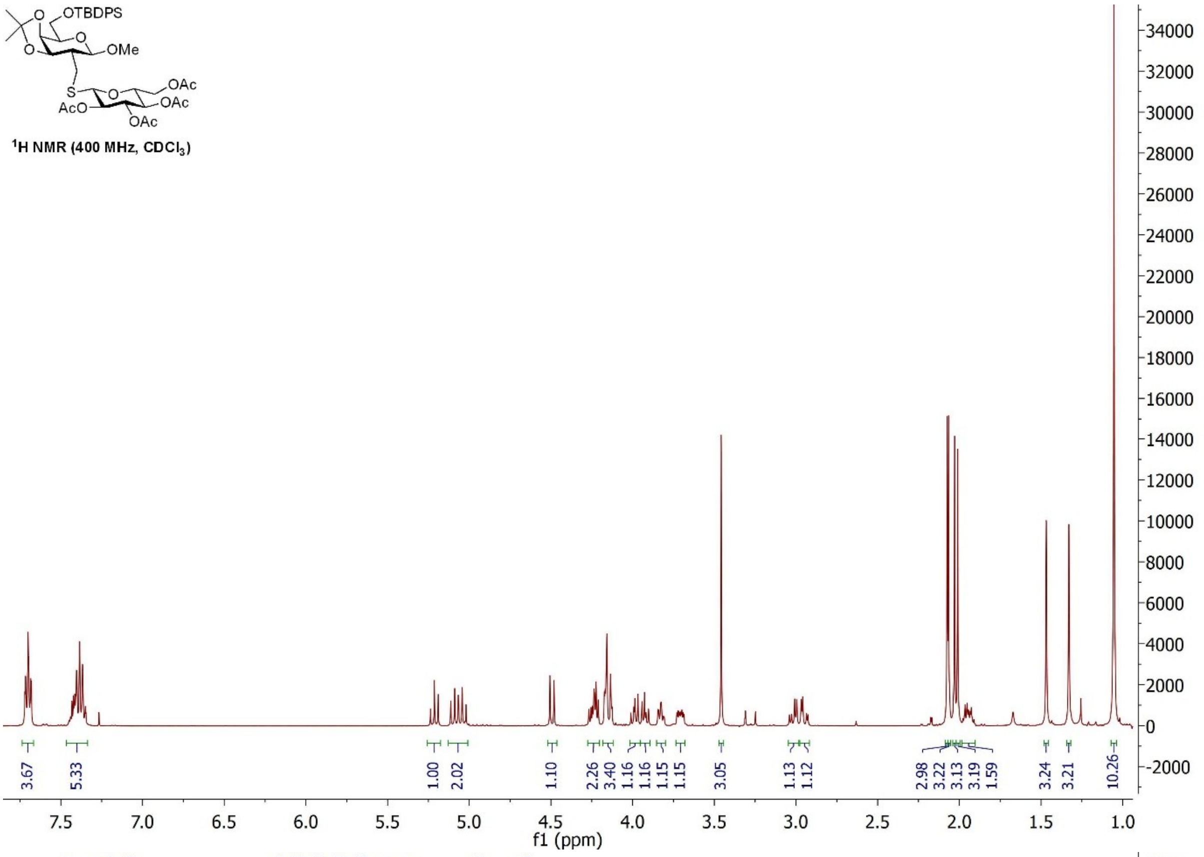
OTBDPS
OMe
OAc
OAc
AcO
OAc
S
O
^{1}H NMR (400 MHz, CDCl$_3$)
3.67 5.33 1.00 2.02 1.10 2.26 3.40 1.16 1.16 1.15 1.15 3.05 1.13 1.12 2.98 3.22 3.13 3.19 1.59 3.24 3.21 10.26
f1 (ppm)

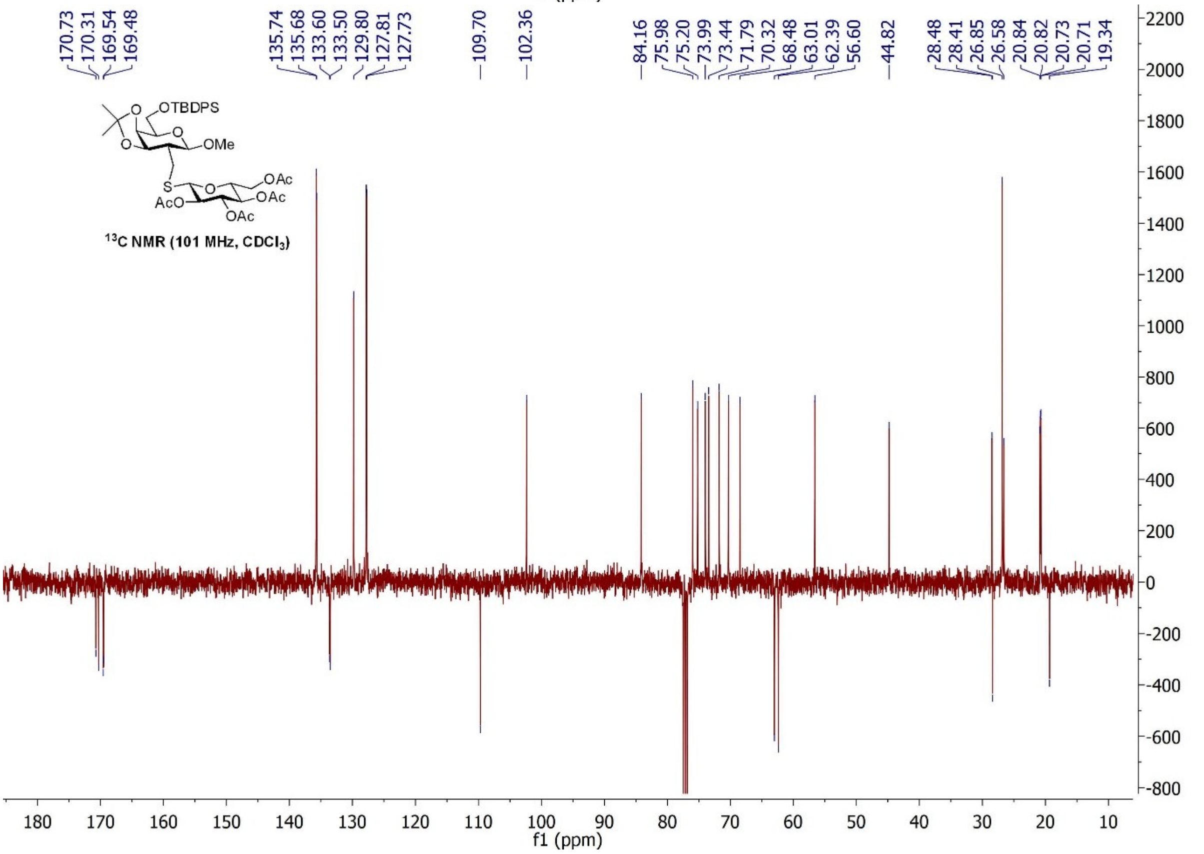
170.73 170.31 169.54 169.48 135.74 135.68 133.60 133.50 129.80 127.81 127.73 109.70 102.36 84.16 75.98 75.20 73.99 73.44 71.79 70.32 68.48 63.01 62.39 56.60 44.82 28.48 28.41 26.85 26.58 20.84 20.82 20.73 20.71 19.34
OTBDPS
OMe
OAc
OAc
AcO
OAc
S
O
^{13}C NMR (101 MHz, CDCl$_3$)
f1 (ppm)

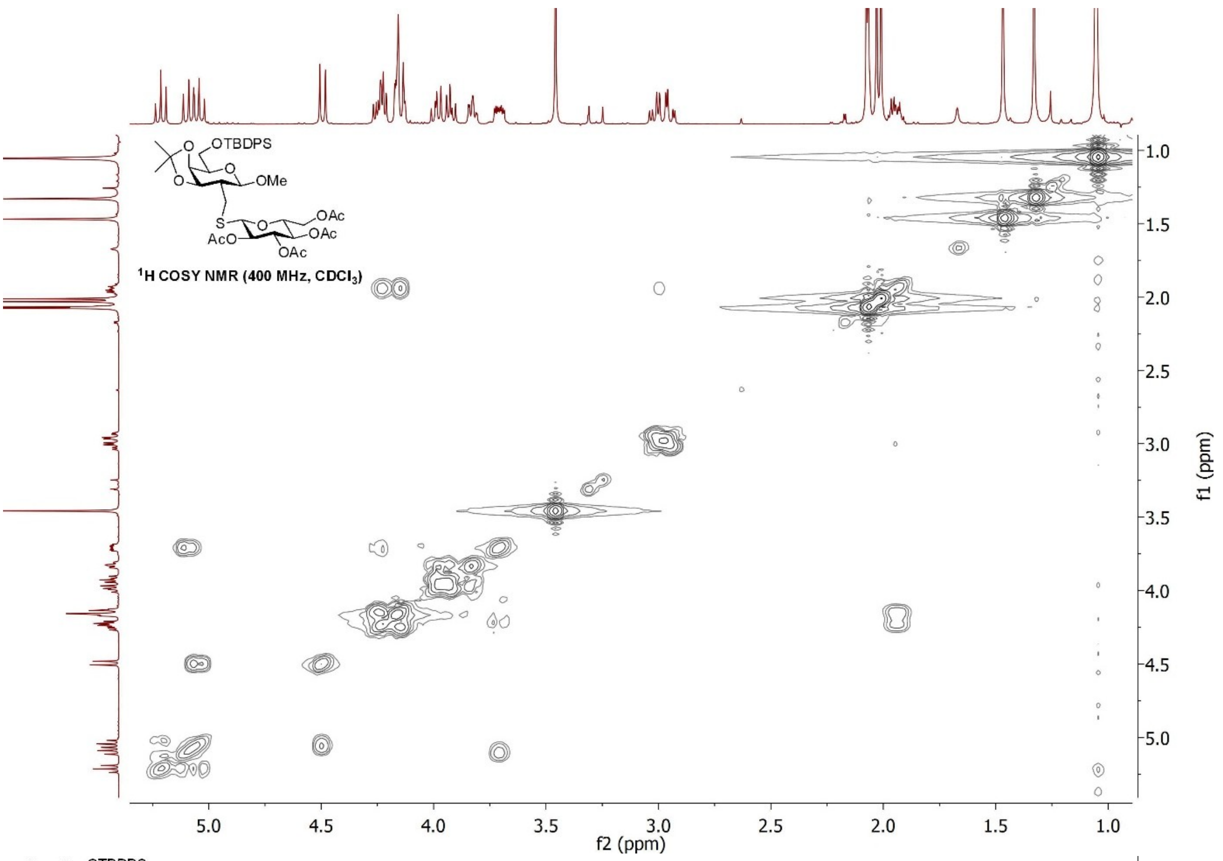
OTBDPS
OMe
S
OAc
AcO
OAc
OAc
1H COSY NMR (400 MHz, CDCl3)
f2 (ppm)
f1 (ppm)

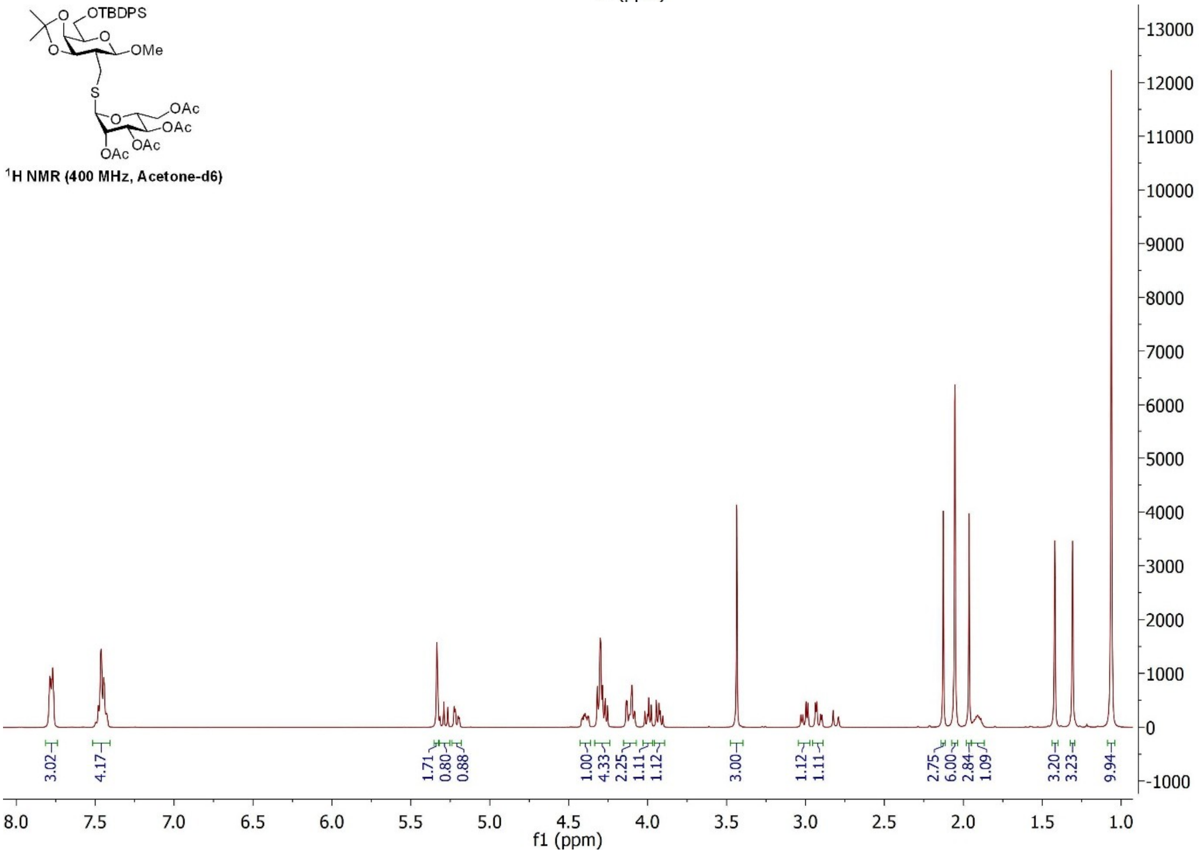
OTBDPS
OMe
S
OAc
OAc
OAc
OAc
1H NMR (400 MHz, Acetone-d6)
3.02
4.17
1.71
0.80
0.88
1.00
4.33
2.25
1.11
1.12
3.00
1.12
1.11
2.75
6.00
2.84
1.09
3.20
3.23
9.94
f1 (ppm)

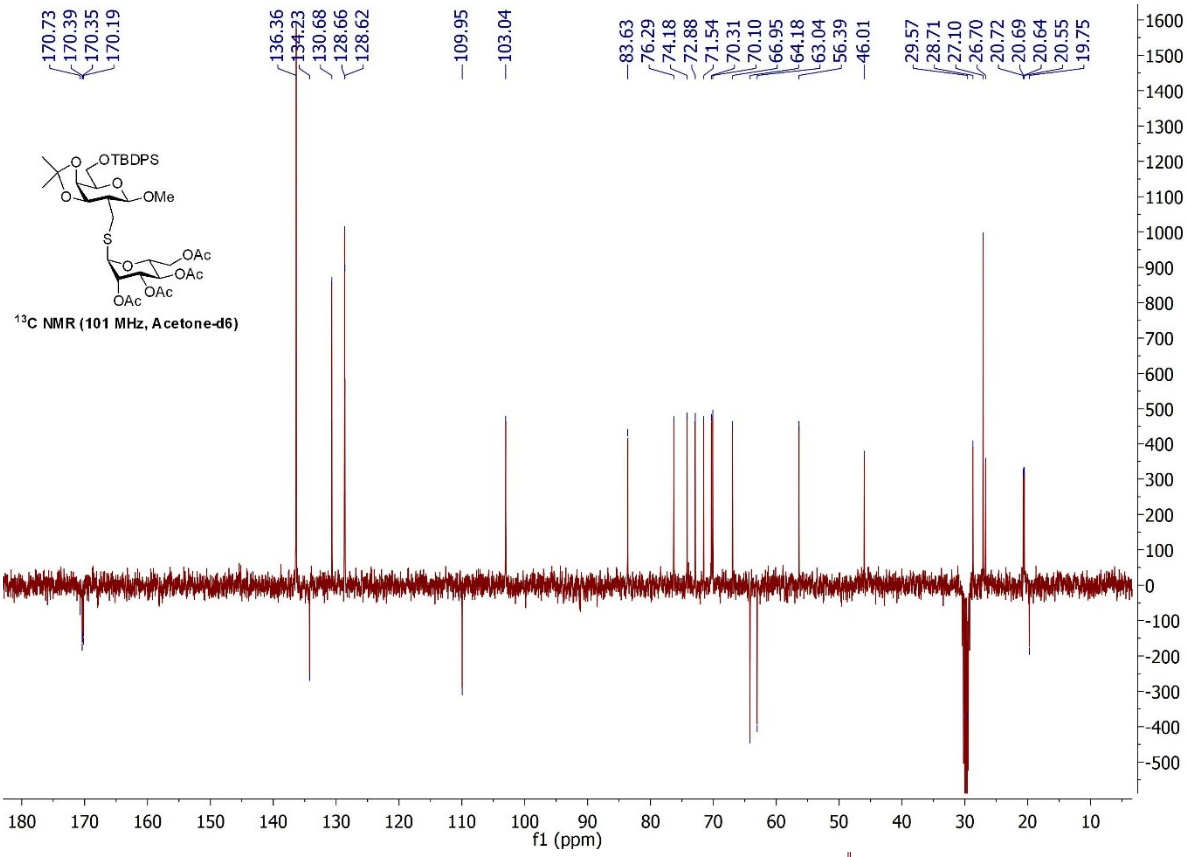
170.73
170.39
170.35
170.19
136.36
134.23
130.68
128.66
128.62
109.95
103.04
83.63
76.29
74.18
72.88
71.54
70.31
70.10
66.95
64.18
63.04
56.39
46.01
29.57
28.71
27.10
26.70
20.72
20.69
20.64
20.55
19.75
OTBDPS
OMe
S
OAc
OAc
OAc
OAc
^{13}C NMR (101 MHz, Acetone-d6)
f1 (ppm)

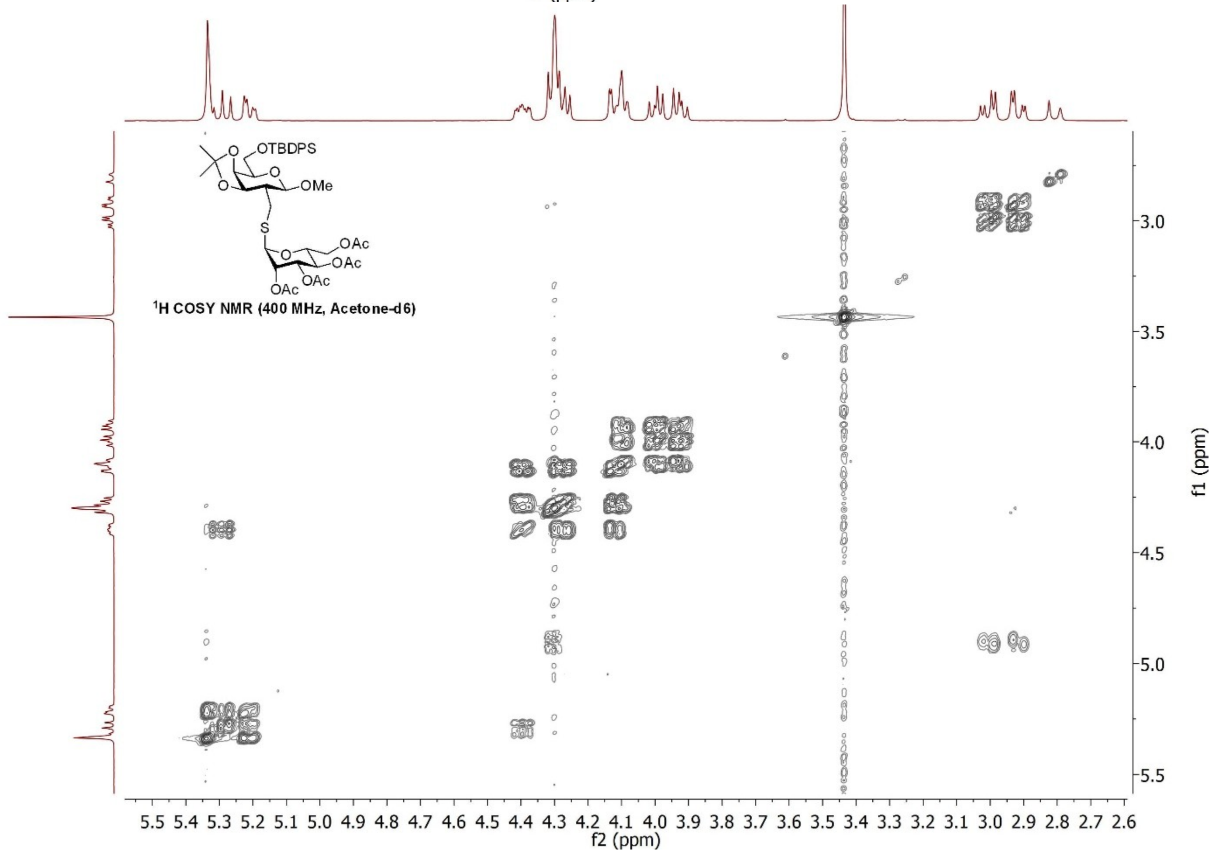
OTBDPS
OMe
S
OAc
OAc
OAc
OAc
^{1}H COSY NMR (400 MHz, Acetone-d6)
f2 (ppm)
f1 (ppm)

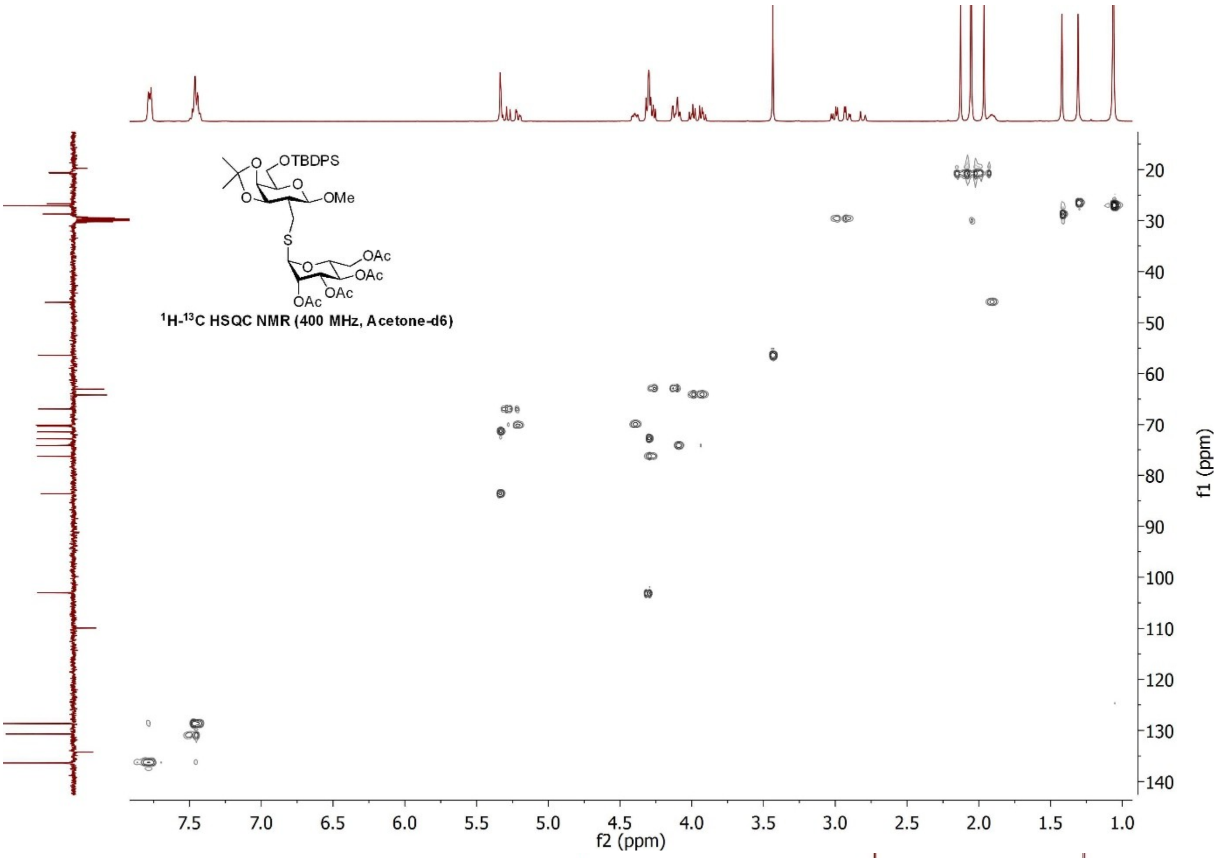

OTBDPS
OMe
S
OAc
OAc
OAc
OAc
¹H-¹³C HSQC NMR (400 MHz, Acetone-d6)
f2 (ppm)
f1 (ppm)

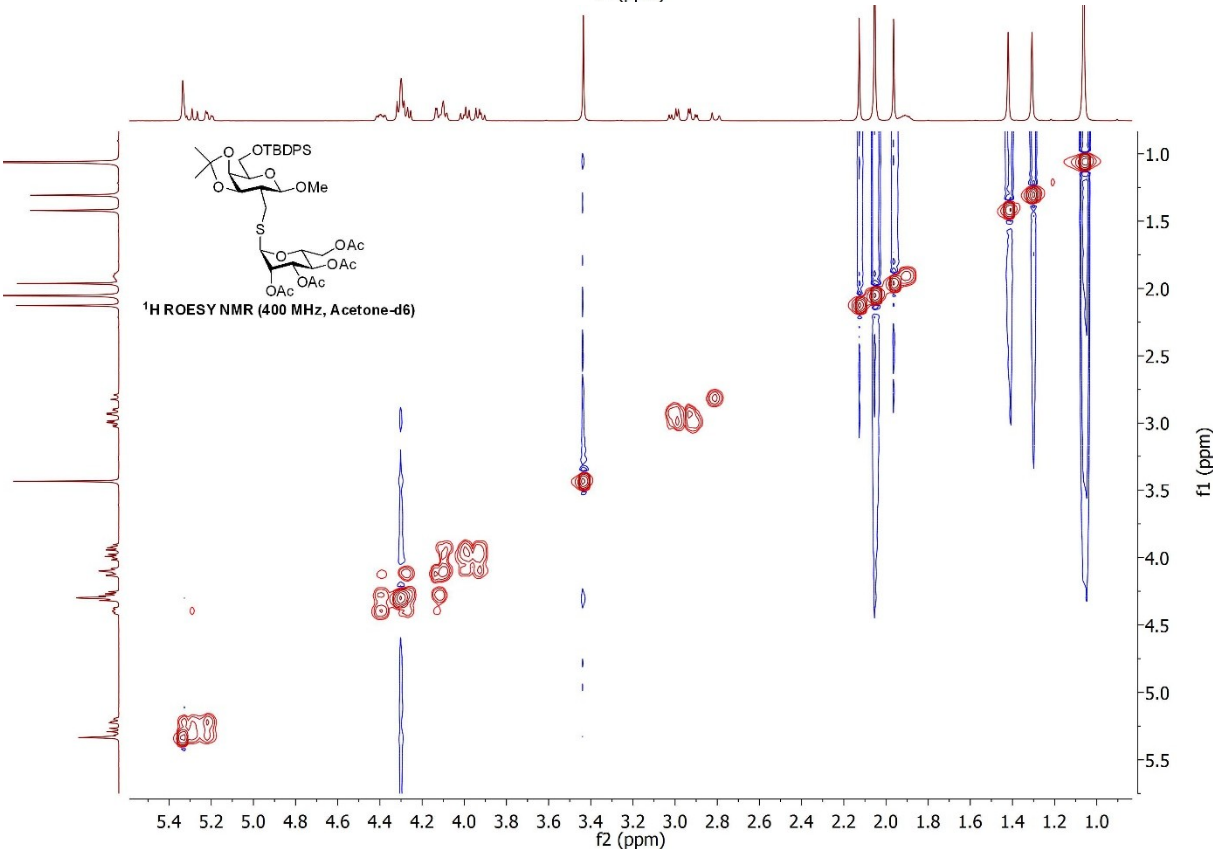

OTBDPS
OMe
S
OAc
OAc
OAc
OAc
¹H ROESY NMR (400 MHz, Acetone-d6)
f2 (ppm)
f1 (ppm)

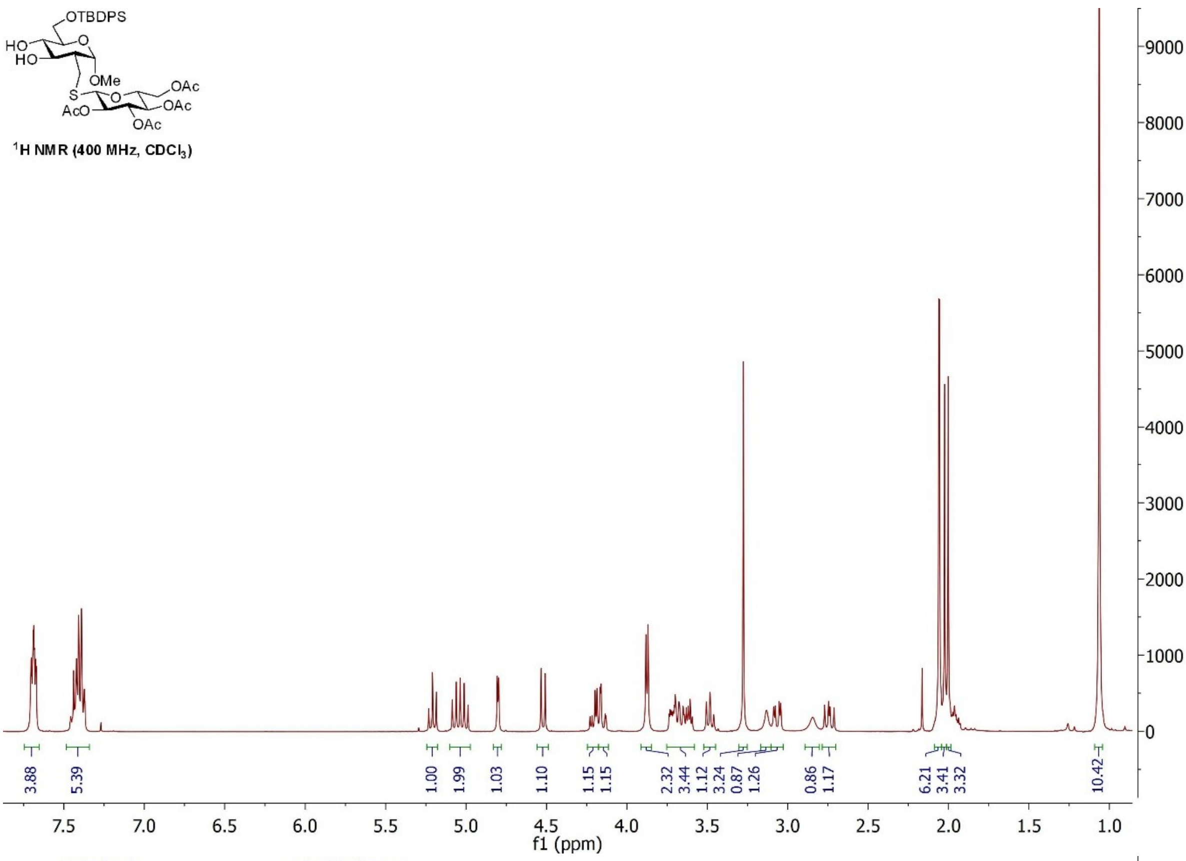
OTBDPS
HO
HO
O
OMe
S
O
OAc
OAc
AcO
OAc
¹H NMR (400 MHz, CDCl₃)
3.88
5.39
1.00
1.99
1.03
1.10
1.15
1.15
2.32
3.44
1.12
3.24
0.87
1.26
0.86
1.17
6.21
3.41
3.32
10.42
7.5 7.0 6.5 6.0 5.5 5.0 4.5 4.0 3.5 3.0 2.5 2.0 1.5 1.0
f1 (ppm)
9000 8000 7000 6000 5000 4000 3000 2000 1000 0

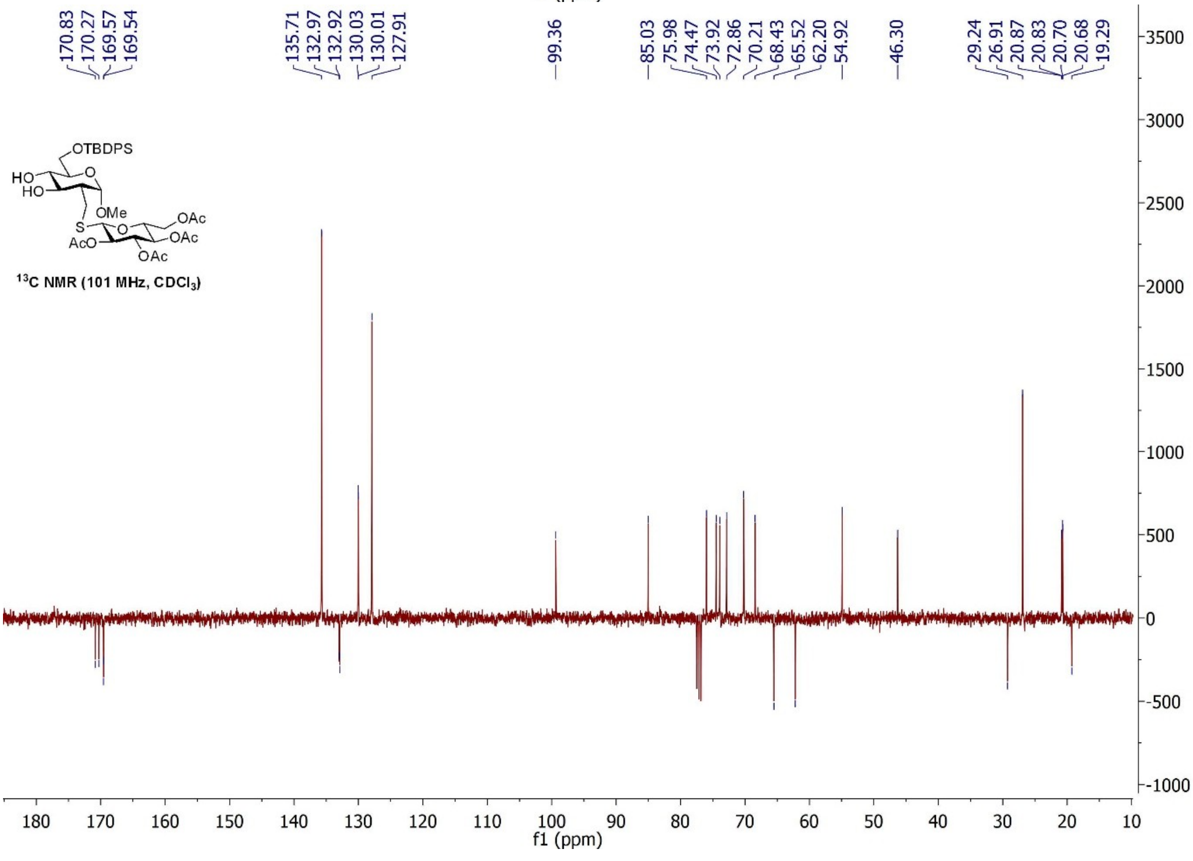
170.83
170.27
169.57
169.54
135.71
132.97
132.92
130.03
130.01
127.91
99.36
85.03
75.98
74.47
73.92
72.86
70.21
68.43
65.52
62.20
54.92
46.30
29.24
26.91
20.87
20.83
20.70
20.68
19.29
OTBDPS
HO
HO
O
OMe
S
O
OAc
OAc
AcO
OAc
¹³C NMR (101 MHz, CDCl₃)
180 170 160 150 140 130 120 110 100 90 80 70 60 50 40 30 20 10
f1 (ppm)
3500 3000 2500 2000 1500 1000 500 0 -500 -1000

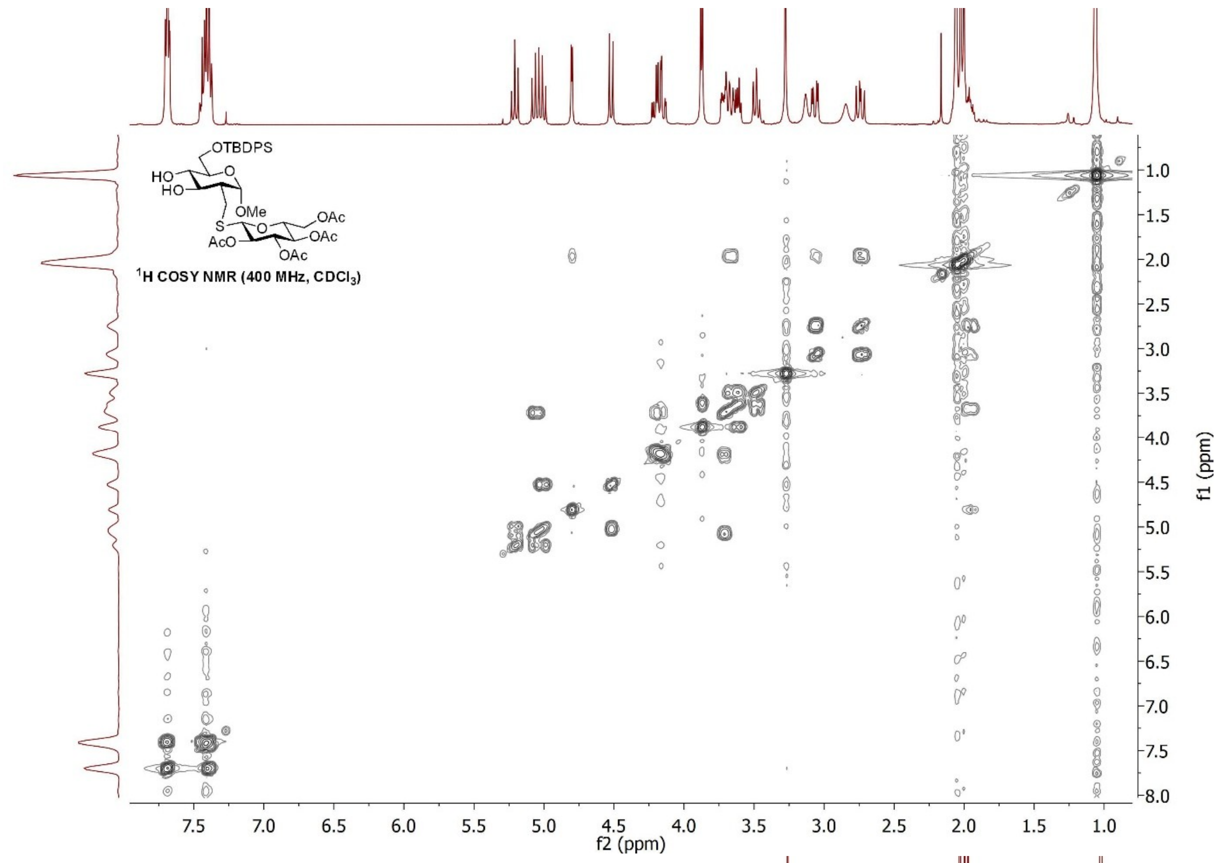
OTBDPS
HO
HO
OMe
S
AcO
OAc
OAc
OAc
^{1}H COSY NMR (400 MHz, CDCl$_3$)
f2 (ppm)
f1 (ppm)

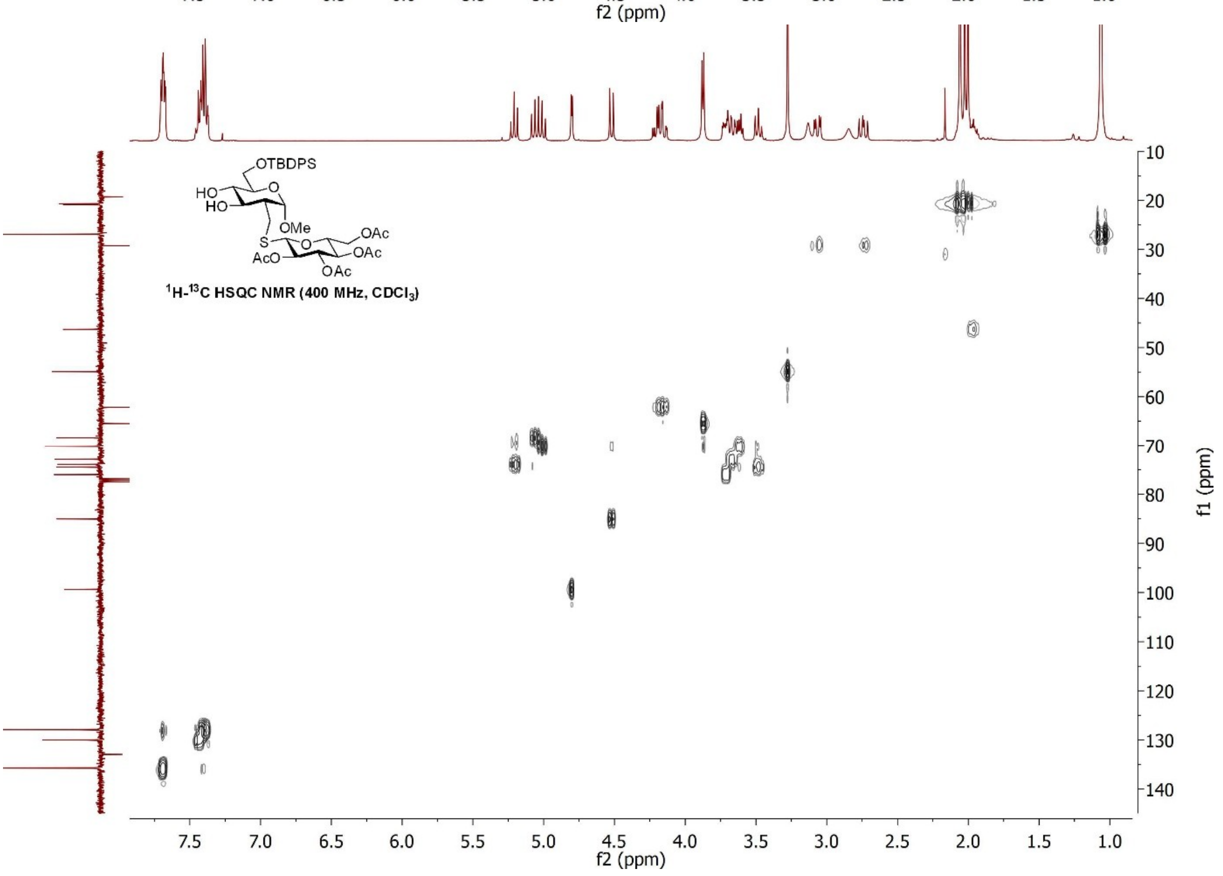
OTBDPS
HO
HO
OMe
S
AcO
OAc
OAc
OAc
^{1}H-^{13}C HSQC NMR (400 MHz, CDCl$_3$)
f2 (ppm)
f1 (ppm)

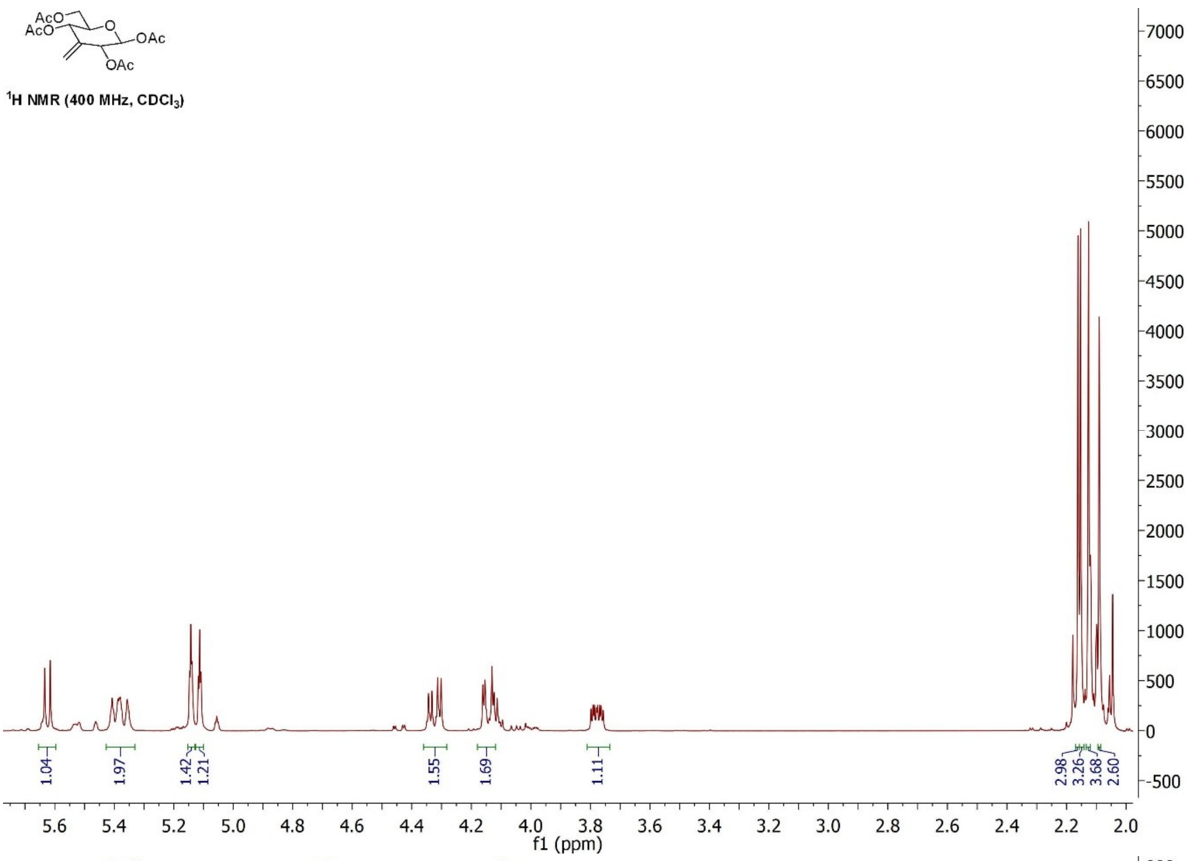
AcO
AcO
OAc
OAc
1H NMR (400 MHz, CDCl3)
1.04
1.97
1.42
1.21
1.55
1.69
1.11
2.98
3.26
3.68
2.60
f1 (ppm)

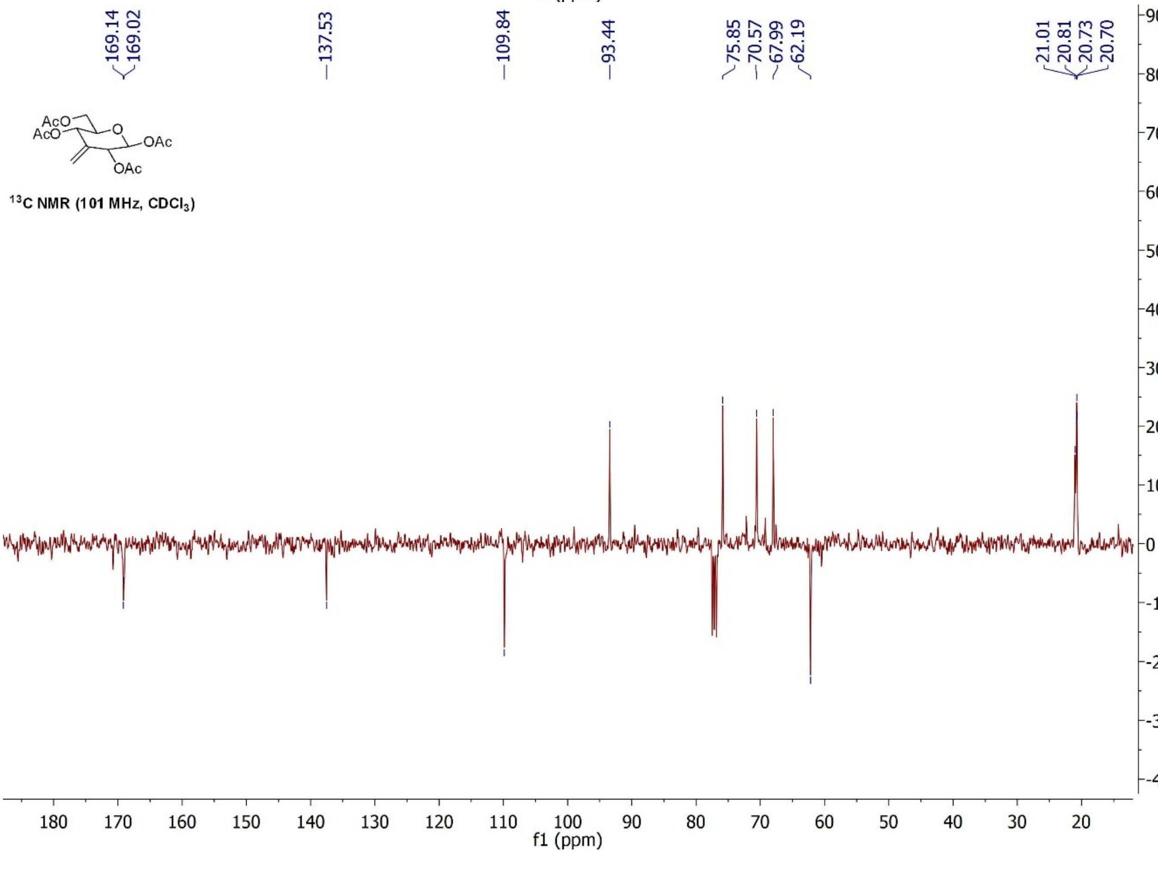
169.14
169.02
137.53
109.84
93.44
75.85
70.57
67.99
62.19
21.01
20.81
20.73
20.70
AcO
AcO
OAc
OAc
13C NMR (101 MHz, CDCl3)
f1 (ppm)

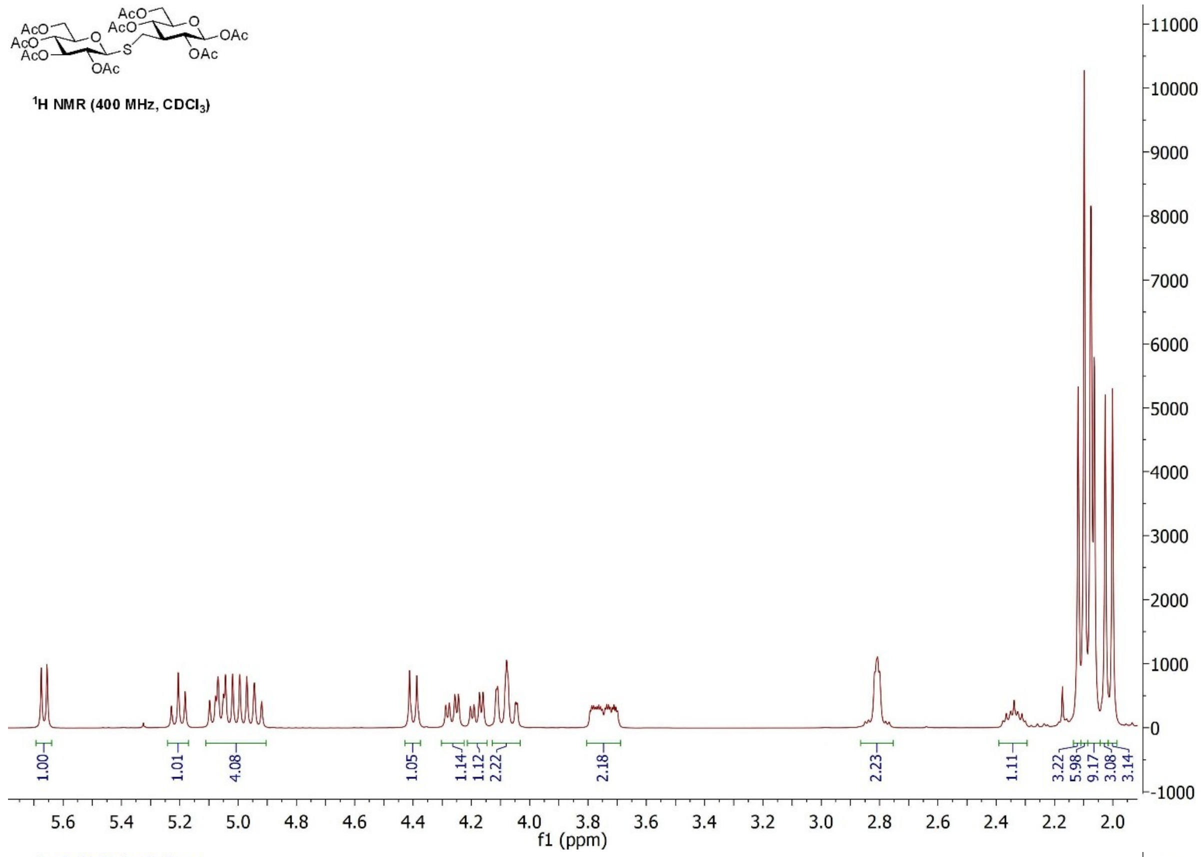

^{1}H NMR (400 MHz, CDCl$_3$)
1.00
1.01
4.08
1.05
1.14
1.12
2.22
2.18
2.23
1.11
3.22
5.98
9.17
3.08
3.14
f1 (ppm)

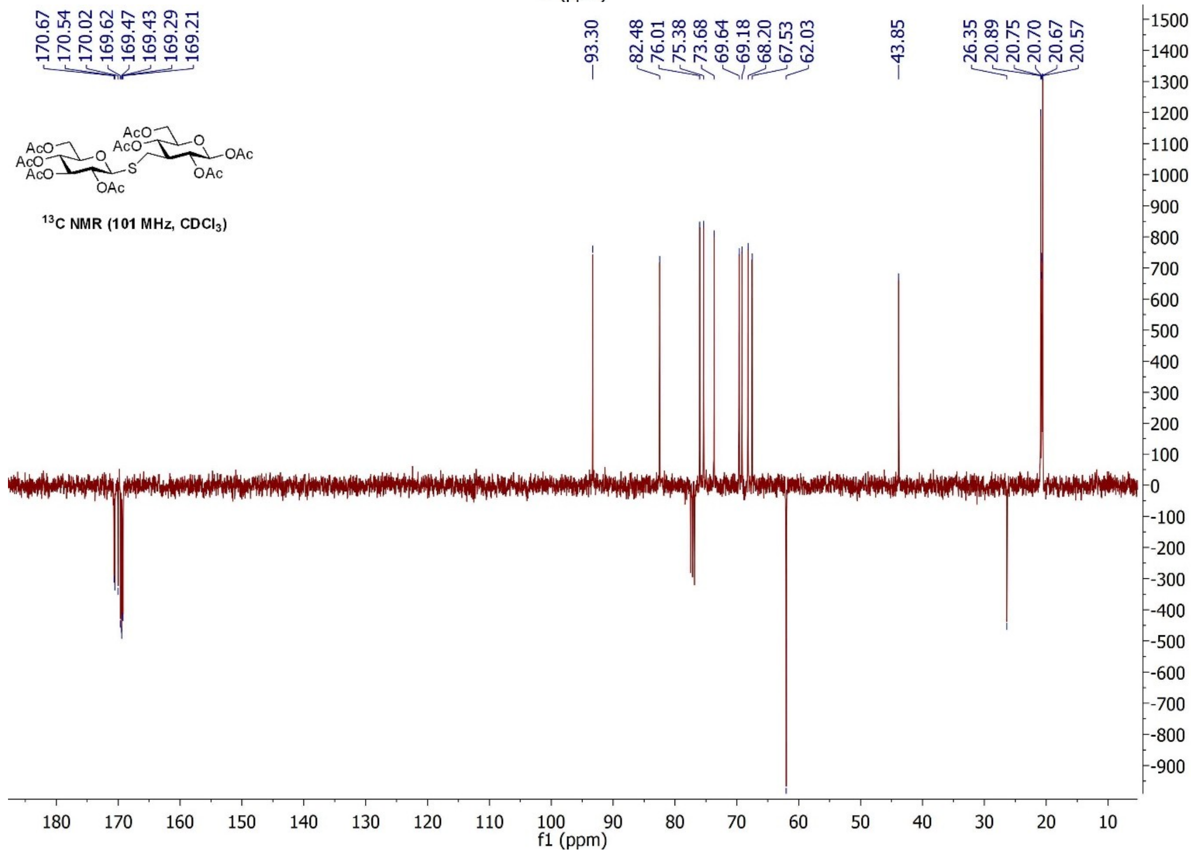

170.67
170.54
170.02
169.62
169.47
169.43
169.29
169.21
93.30
82.48
76.01
75.38
73.68
69.64
69.18
68.20
67.53
62.03
43.85
26.35
20.89
20.75
20.70
20.67
20.57
^{13}C NMR (101 MHz, CDCl$_3$)
f1 (ppm)

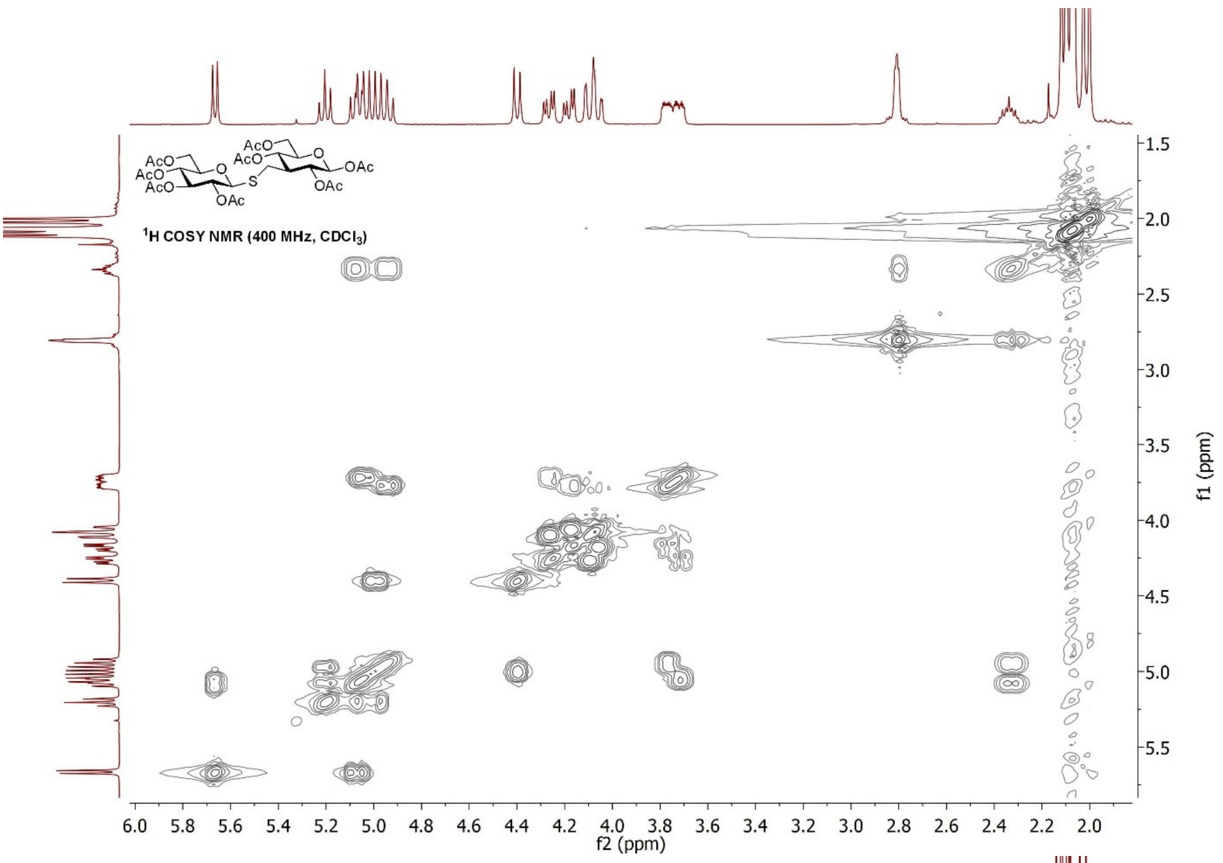

AcO
AcO
AcO
OAc
S
AcO
AcO
OAc
OAc
O
O
¹H COSY NMR (400 MHz, CDCl₃)
f2 (ppm)
f1 (ppm)

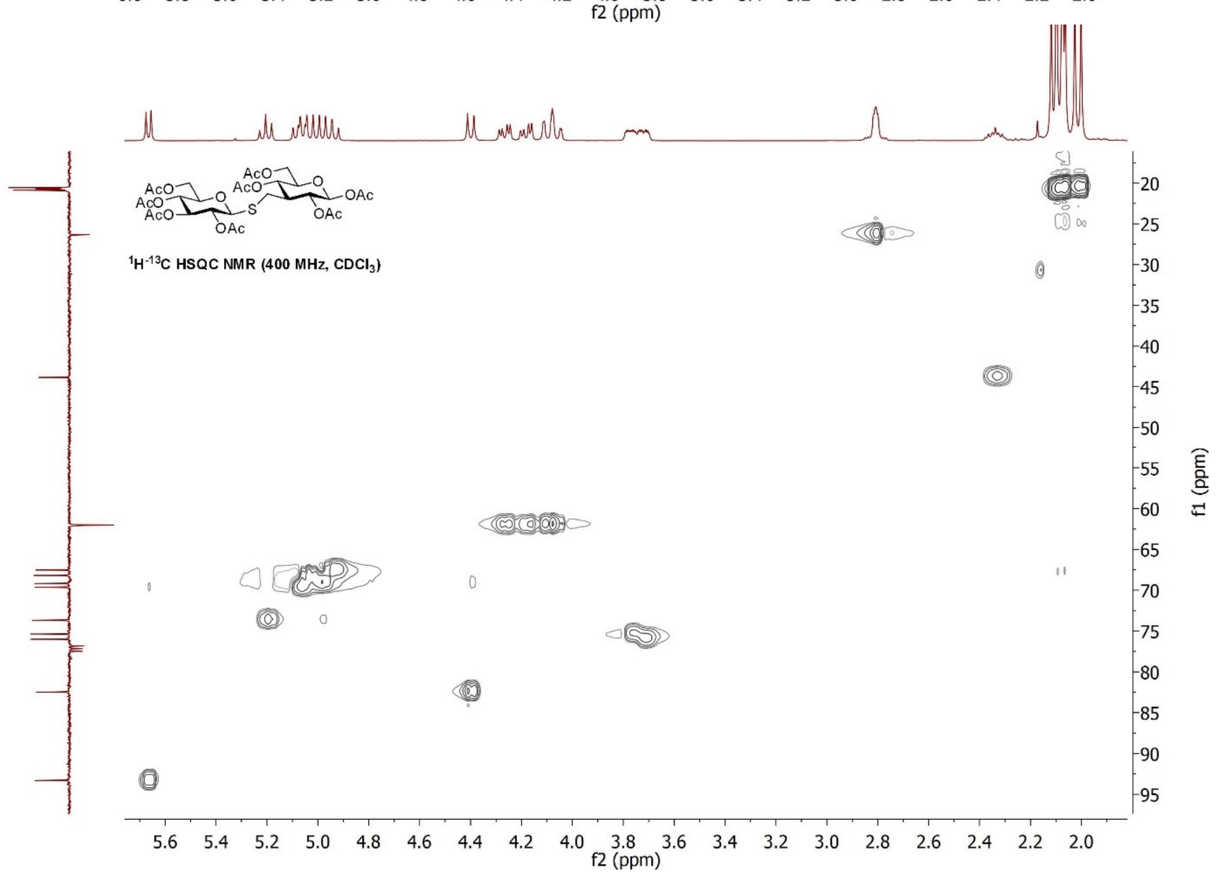

AcO
AcO
AcO
OAc
S
AcO
AcO
OAc
OAc
¹H-¹³C HSQC NMR (400 MHz, CDCl₃)
f2 (ppm)
f1 (ppm)

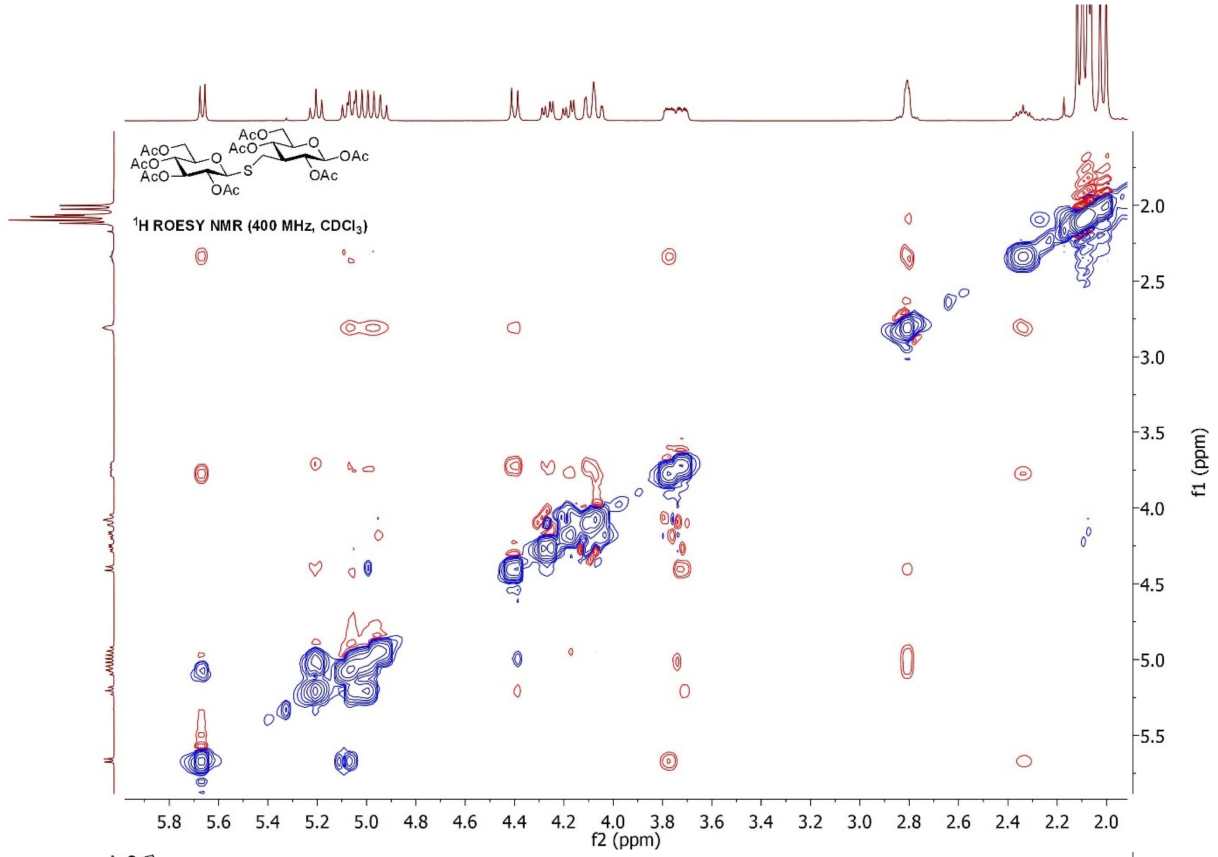
1H ROESY NMR (400 MHz, CDCl3)
f2 (ppm)
f1 (ppm)

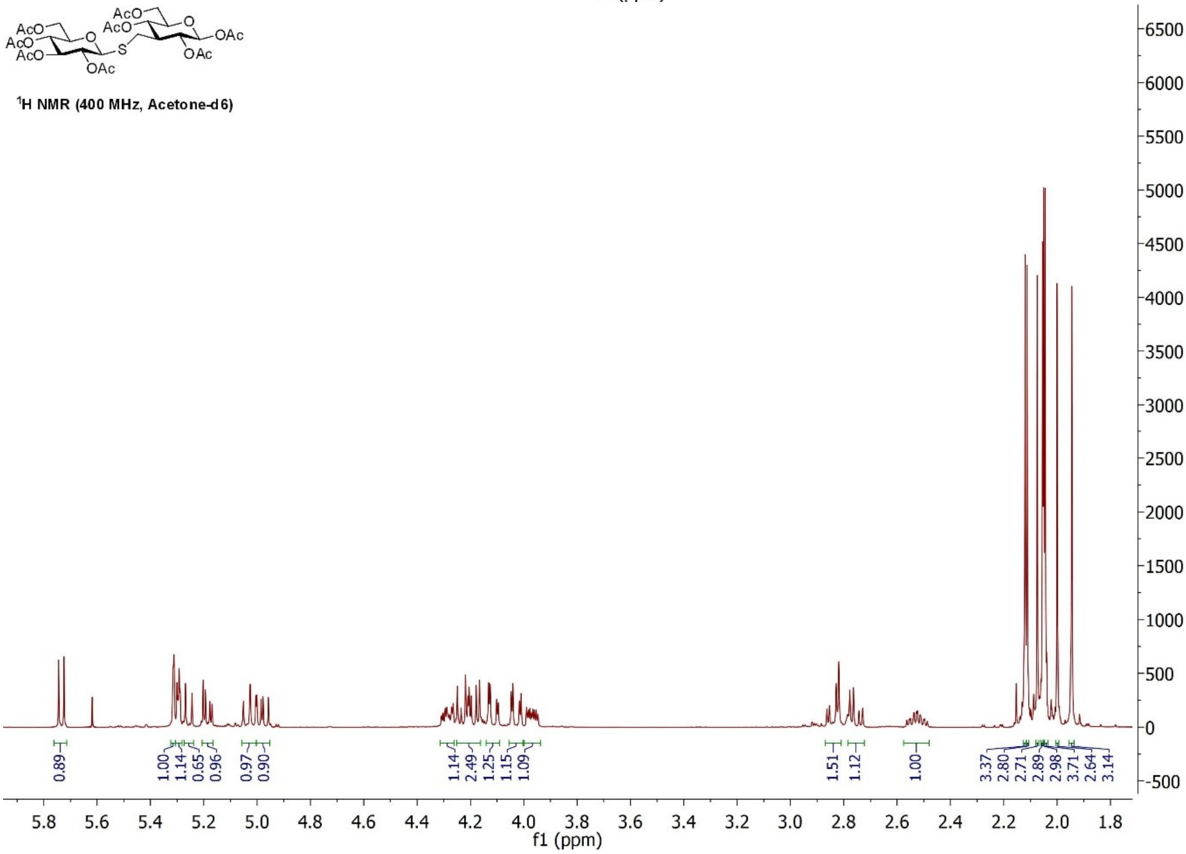
1H NMR (400 MHz, Acetone-d6)
0.89
1.00
1.14
0.65
0.96
0.97
0.90
1.14
2.49
1.25
1.15
1.09
1.51
1.12
1.00
3.37
2.80
2.71
2.89
2.98
3.71
2.64
3.14
f1 (ppm)

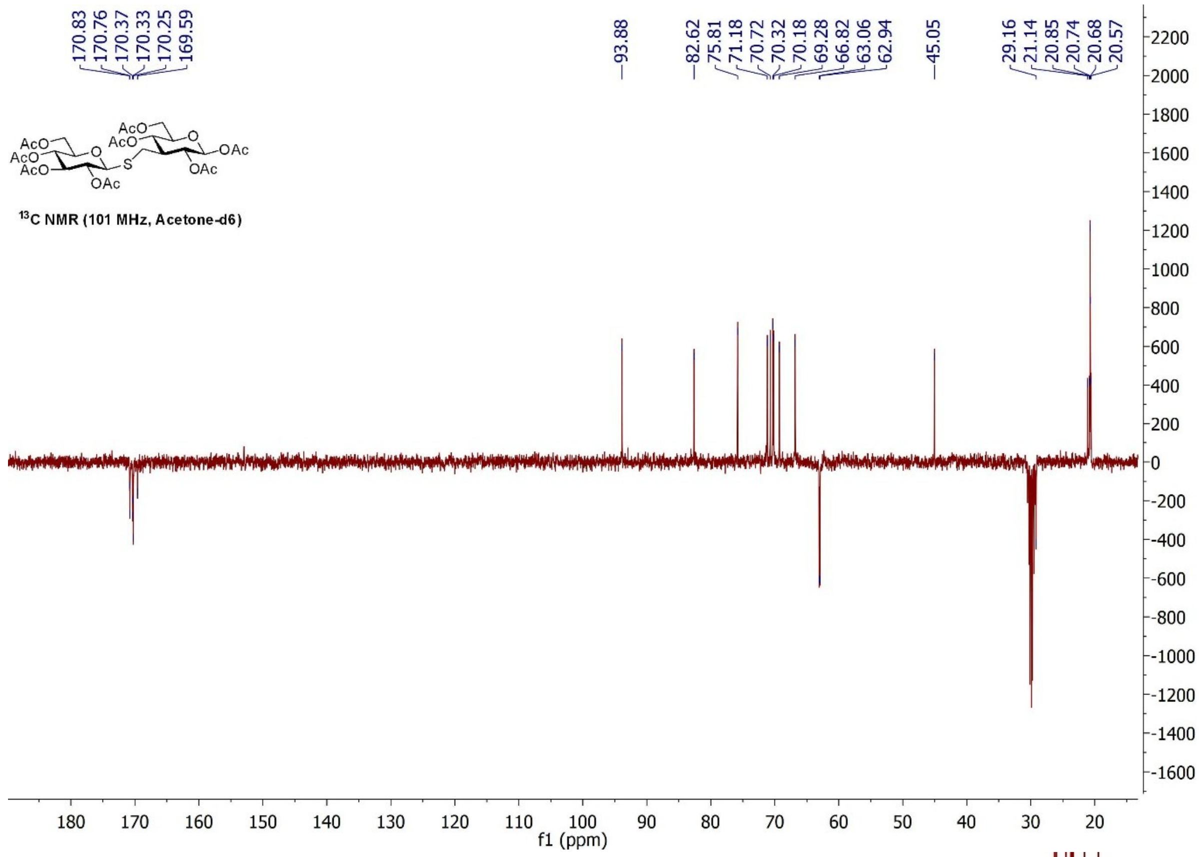
170.83
170.76
170.37
170.33
170.25
169.59
93.88
82.62
75.81
71.18
70.72
70.32
70.18
69.28
66.82
63.06
62.94
45.05
29.16
21.14
20.85
20.74
20.68
20.57
AcO
AcO
AcO
AcO
AcO
O
O
S
OAc
OAc
OAc
13C NMR (101 MHz, Acetone-d6)
f1 (ppm)

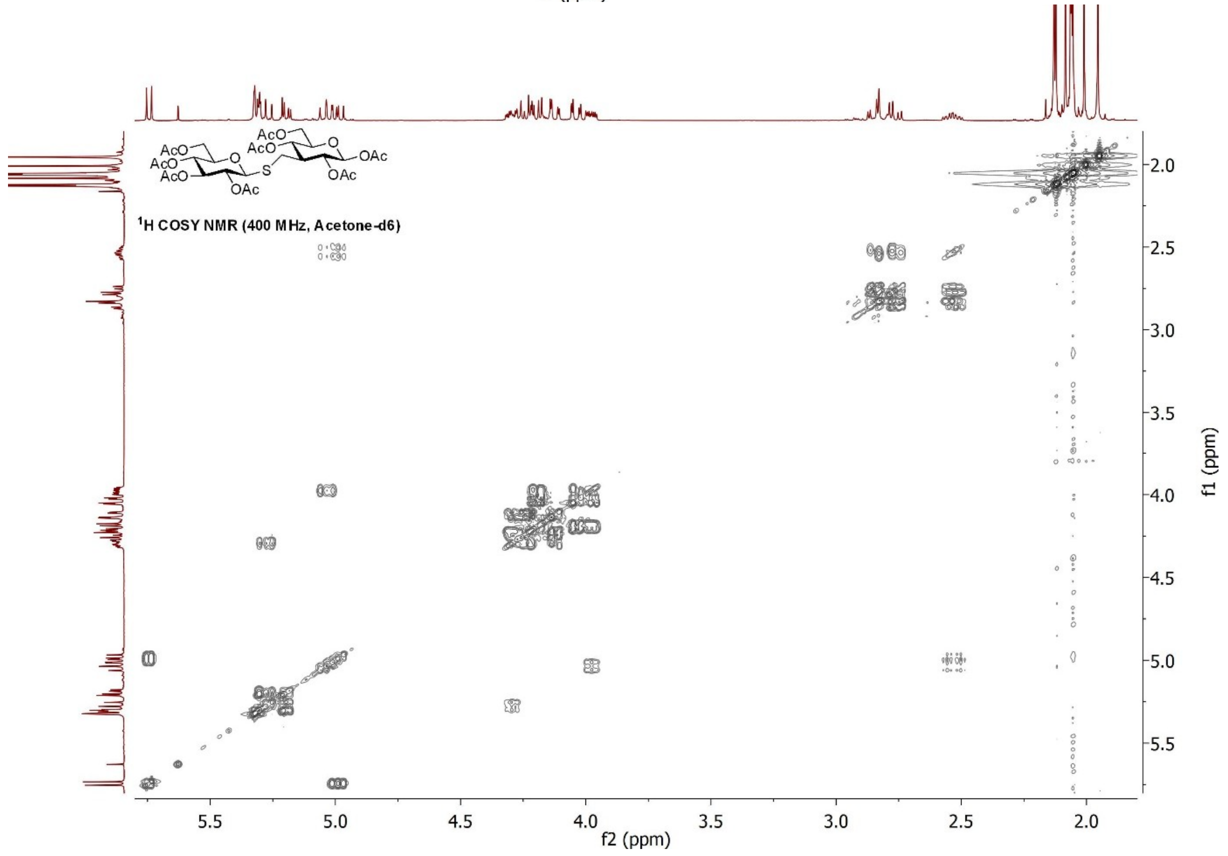
AcO
AcO
AcO
AcO
AcO
O
O
S
OAc
OAc
OAc
1H COSY NMR (400 MHz, Acetone-d6)
f2 (ppm)
f1 (ppm)

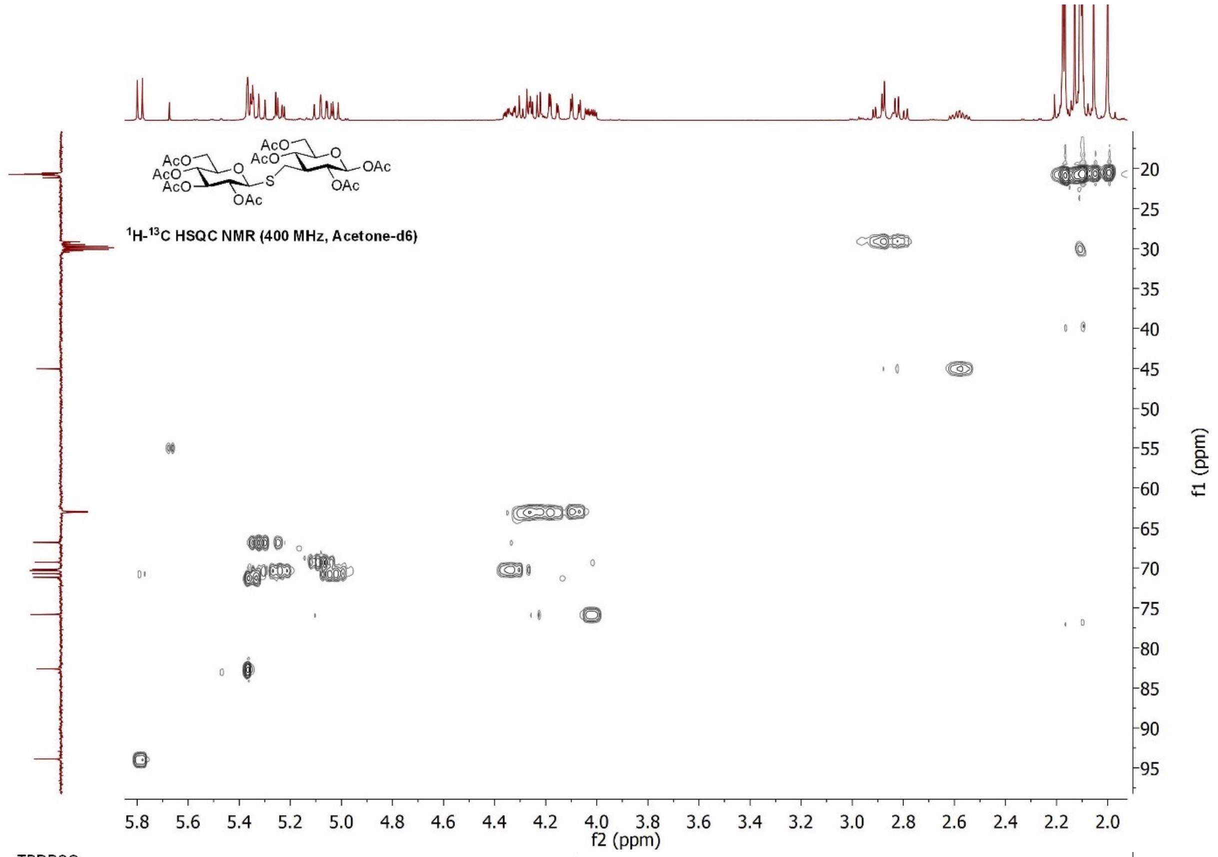
AcO AcO AcO OAc S AcO AcO OAc OAc
^{1}H-^{13}C HSQC NMR (400 MHz, Acetone-d6)
f2 (ppm)
f1 (ppm)

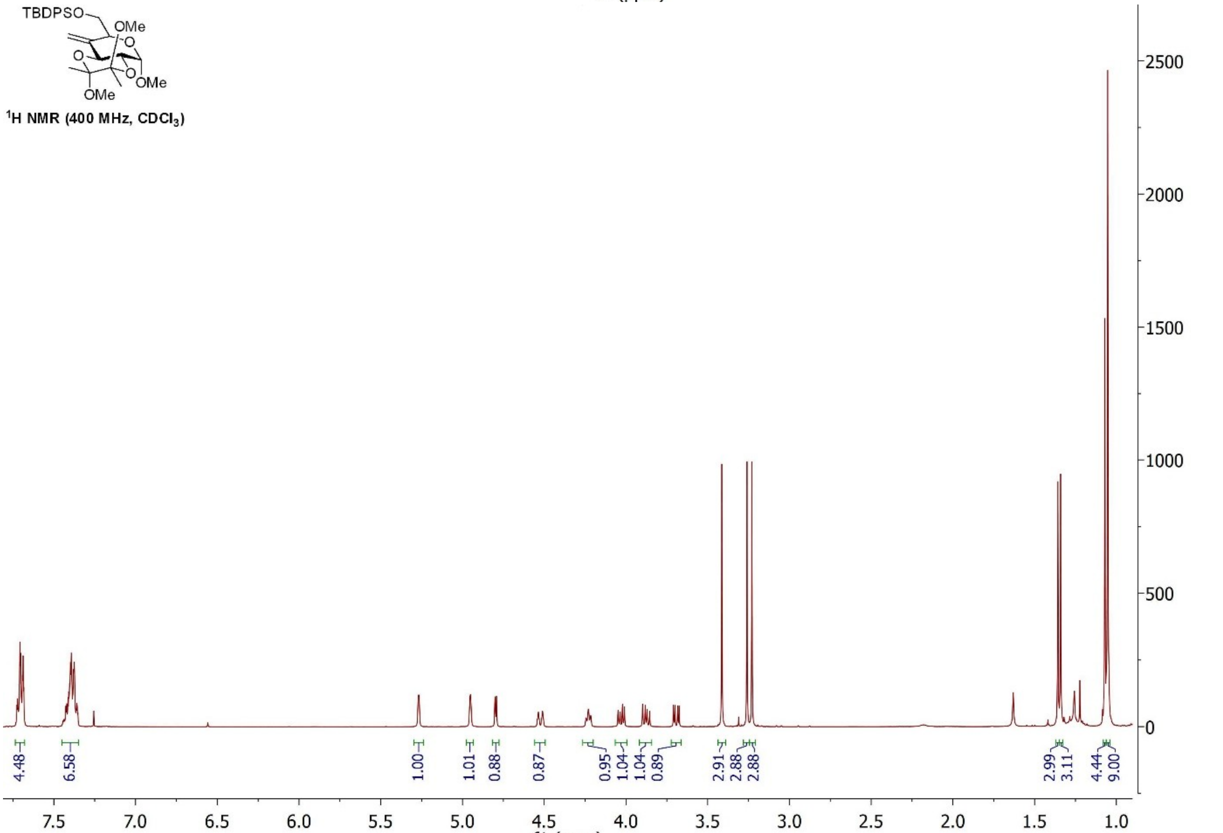
TBDPSO OMe OMe OMe
^{1}H NMR (400 MHz, $CDCl_3$)
4.48 6.58 1.00 1.01 0.88 0.87 0.95 1.04 1.04 0.89 2.91 2.88 2.88 2.99 3.11 4.44 9.00
f1 (ppm)

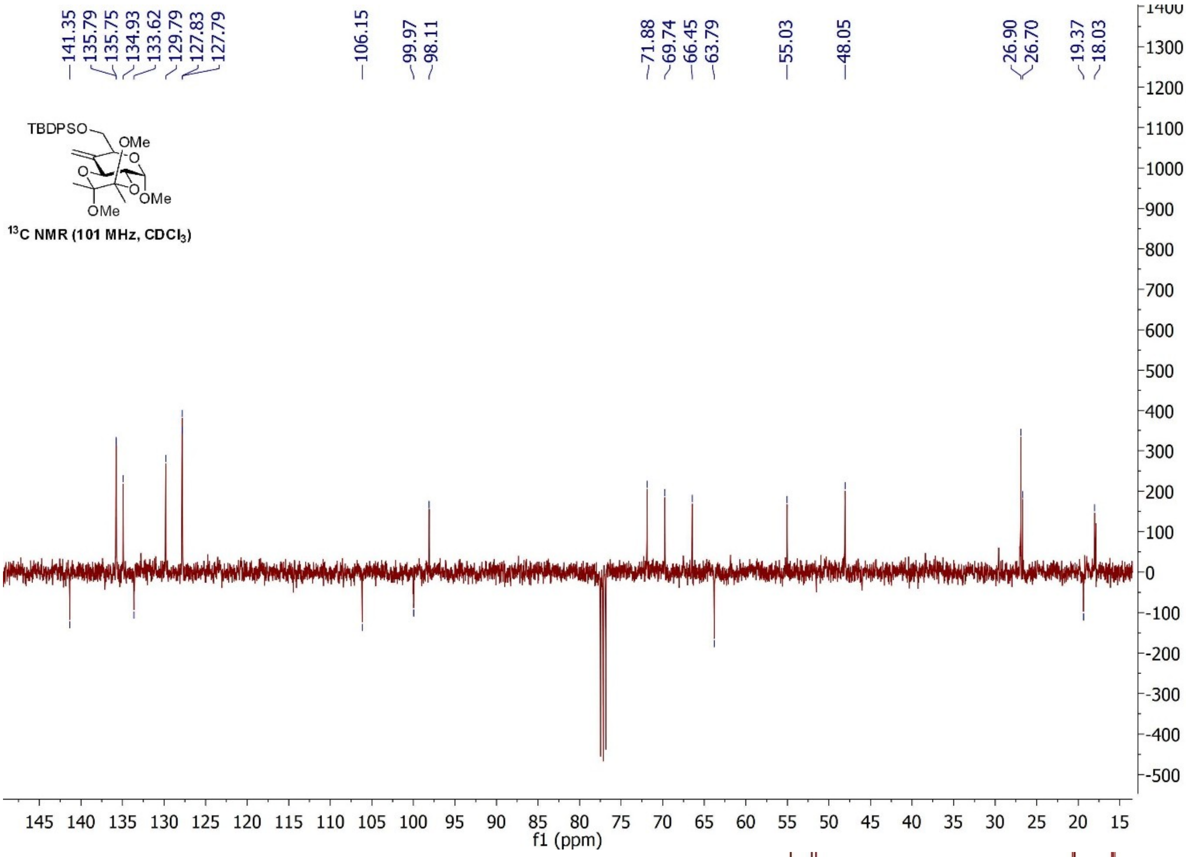
141.35
135.79
135.75
134.93
133.62
129.79
127.83
127.79
106.15
99.97
98.11
71.88
69.74
66.45
63.79
55.03
48.05
26.90
26.70
19.37
18.03
TBDPSO
OMe
O
O
O
OMe
OMe
^{13}C NMR (101 MHz, CDCl$_3$)
f1 (ppm)

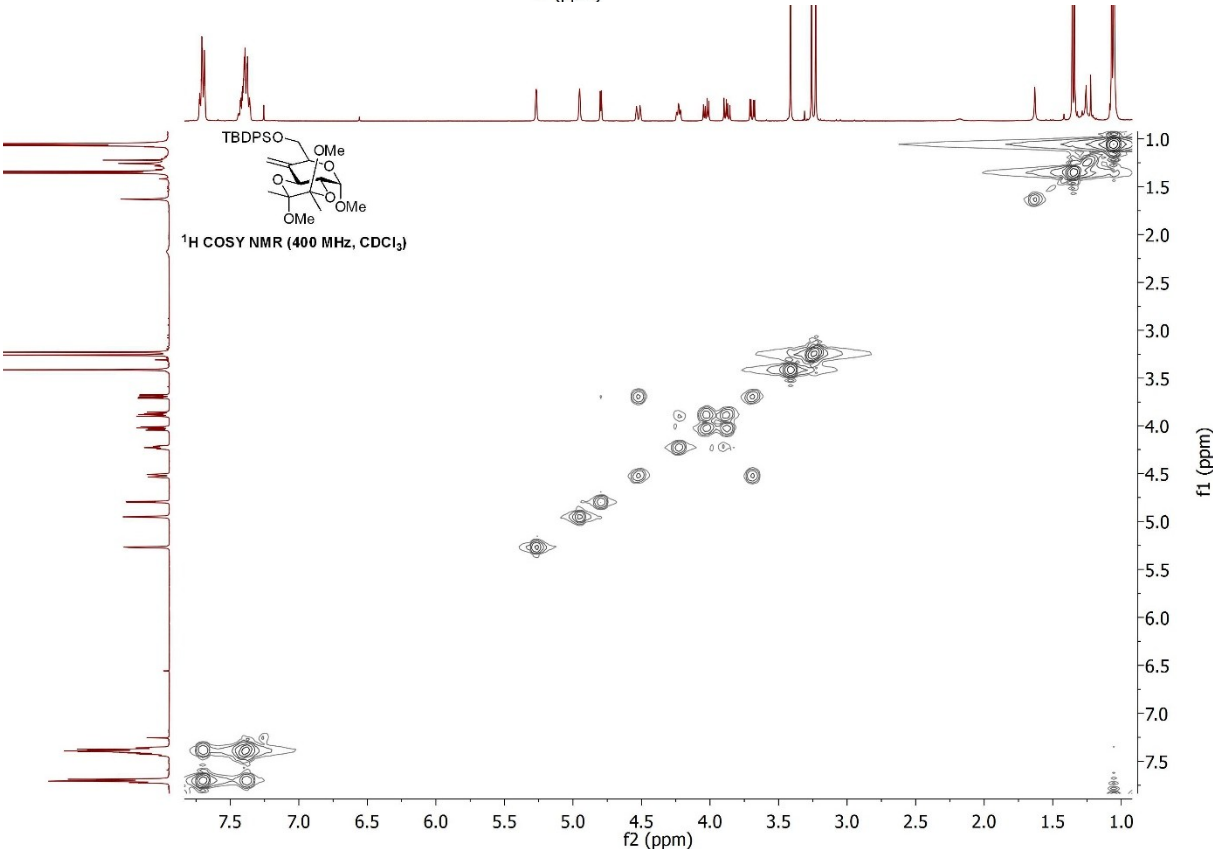
TBDPSO
OMe
O
O
O
OMe
OMe
^{1}H COSY NMR (400 MHz, CDCl$_3$)
f2 (ppm)
f1 (ppm)

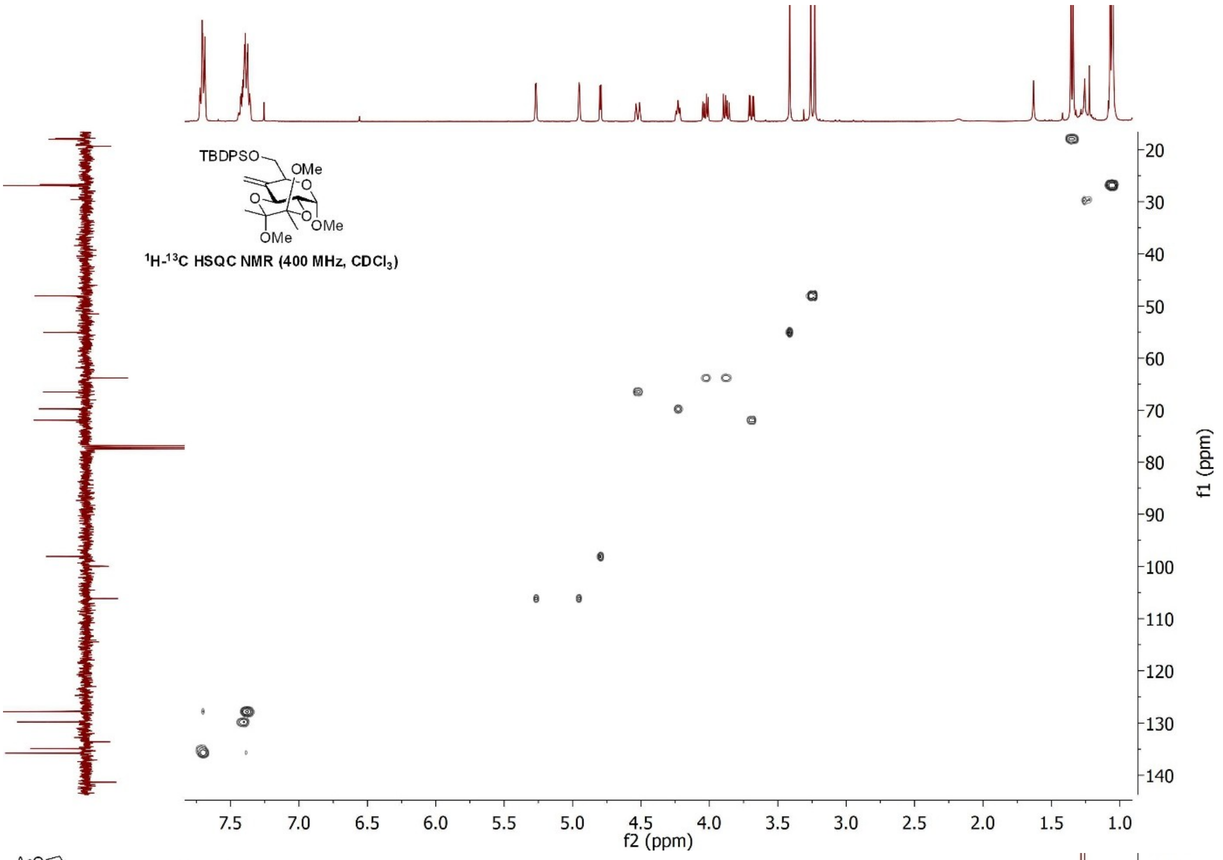
TBDPSO
OMe
OMe
OMe
1H-13C HSQC NMR (400 MHz, CDCl3)
f2 (ppm)
f1 (ppm)

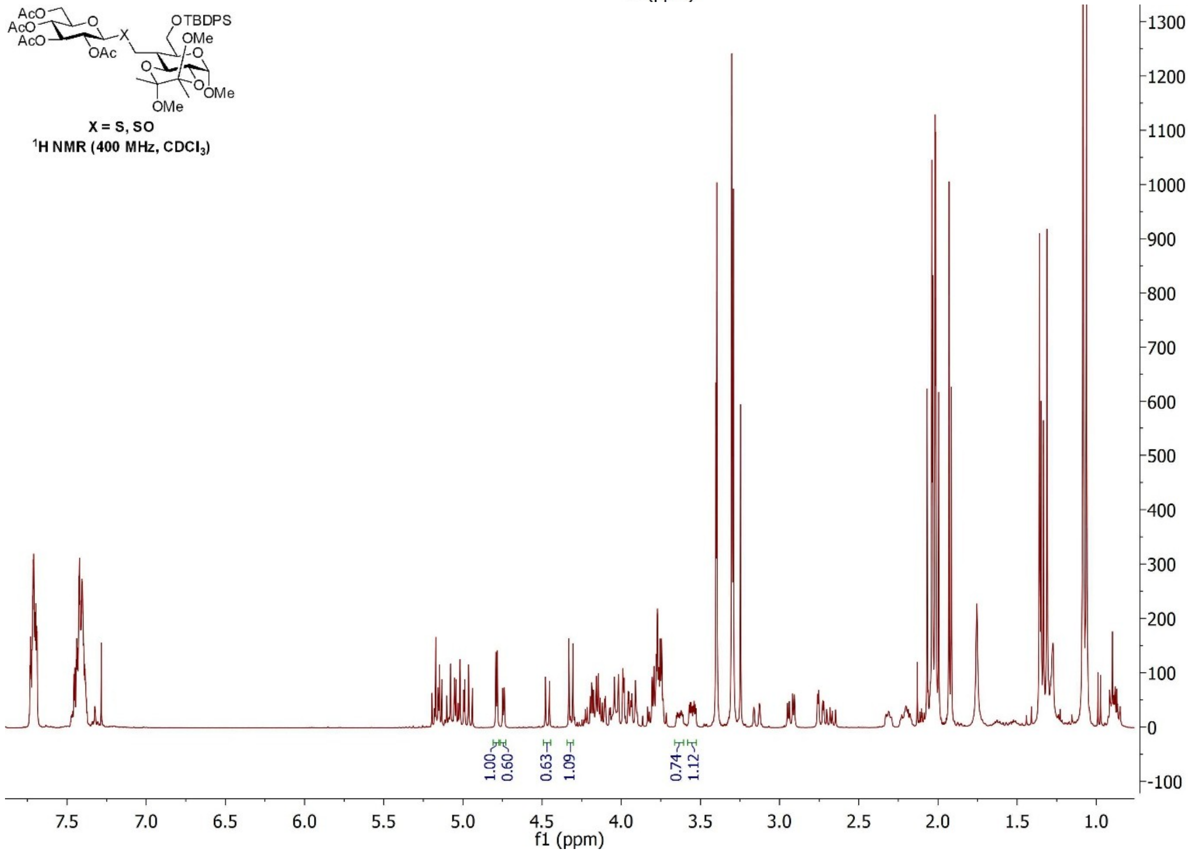
AcO
AcO
AcO
OAc
X
OTBDPS
OMe
OMe
OMe
X = S, SO
1H NMR (400 MHz, CDCl3)
f1 (ppm)

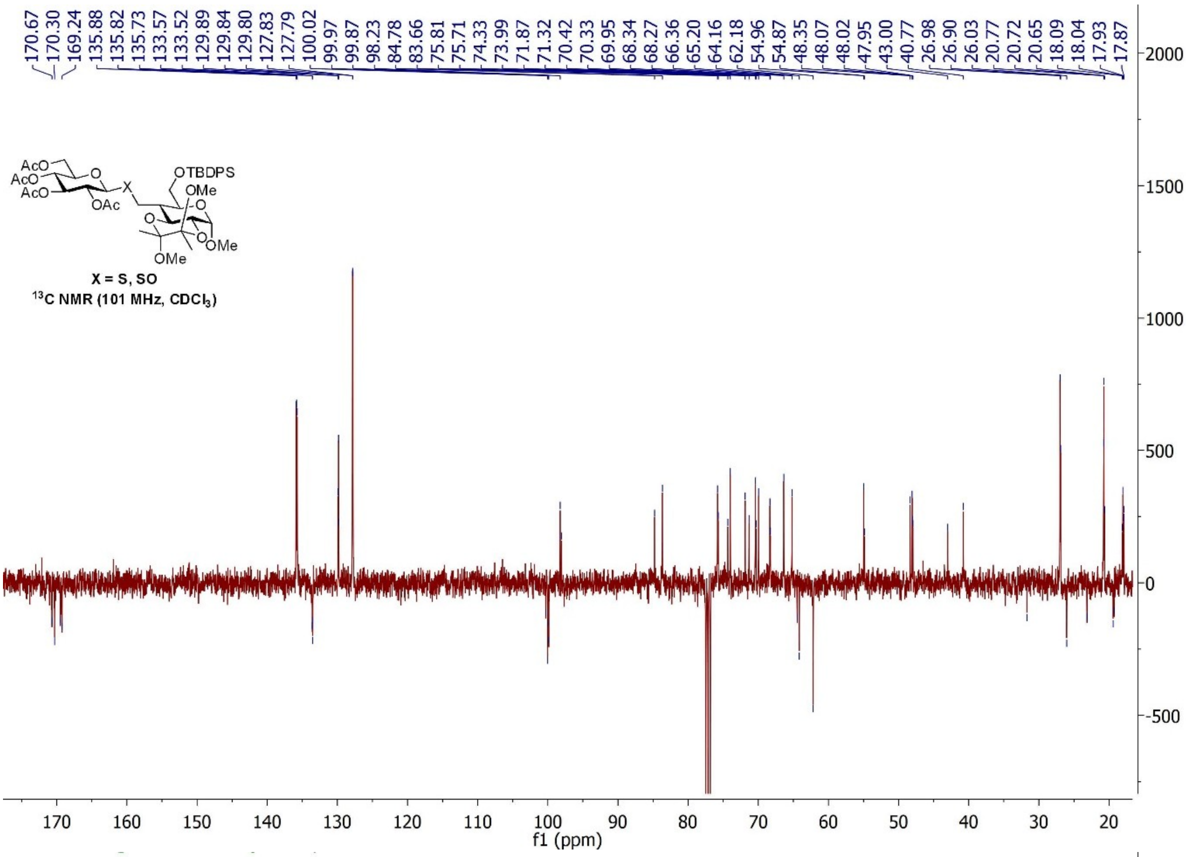
170.67
170.30
169.24
135.88
135.82
135.73
133.57
133.52
129.89
129.84
129.80
127.83
127.79
100.02
99.97
99.87
98.23
84.78
83.66
75.81
75.71
74.33
73.99
71.87
71.32
70.42
70.33
69.95
68.34
68.27
66.36
65.20
64.16
62.18
54.96
54.87
48.35
48.07
48.02
47.95
43.00
40.77
26.98
26.90
26.03
20.77
20.72
20.65
18.09
18.04
17.93
17.87
AcO
AcO
AcO
OAc
X
OTBDPS
OMe
O
O
O
OMe
OMe
X = S, SO
13C NMR (101 MHz, CDCl3)
f1 (ppm)

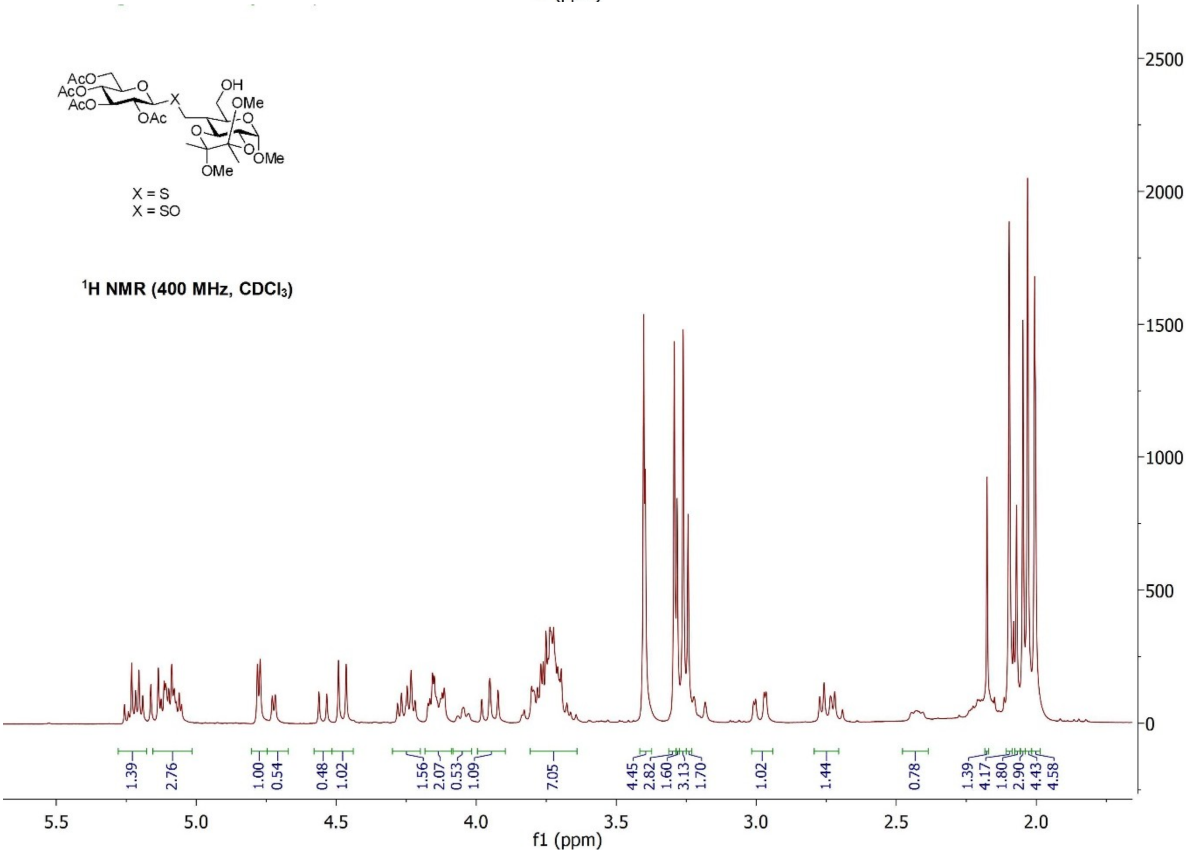
AcO
AcO
AcO
OAc
X
OH
OMe
O
O
O
OMe
OMe
X = S
X = SO
1H NMR (400 MHz, CDCl3)
1.39
2.76
1.00
0.54
0.48
1.02
1.56
2.07
0.53
1.09
7.05
4.45
2.82
1.60
3.13
1.70
1.02
1.44
0.78
1.39
4.17
1.80
2.90
4.43
4.58
f1 (ppm)

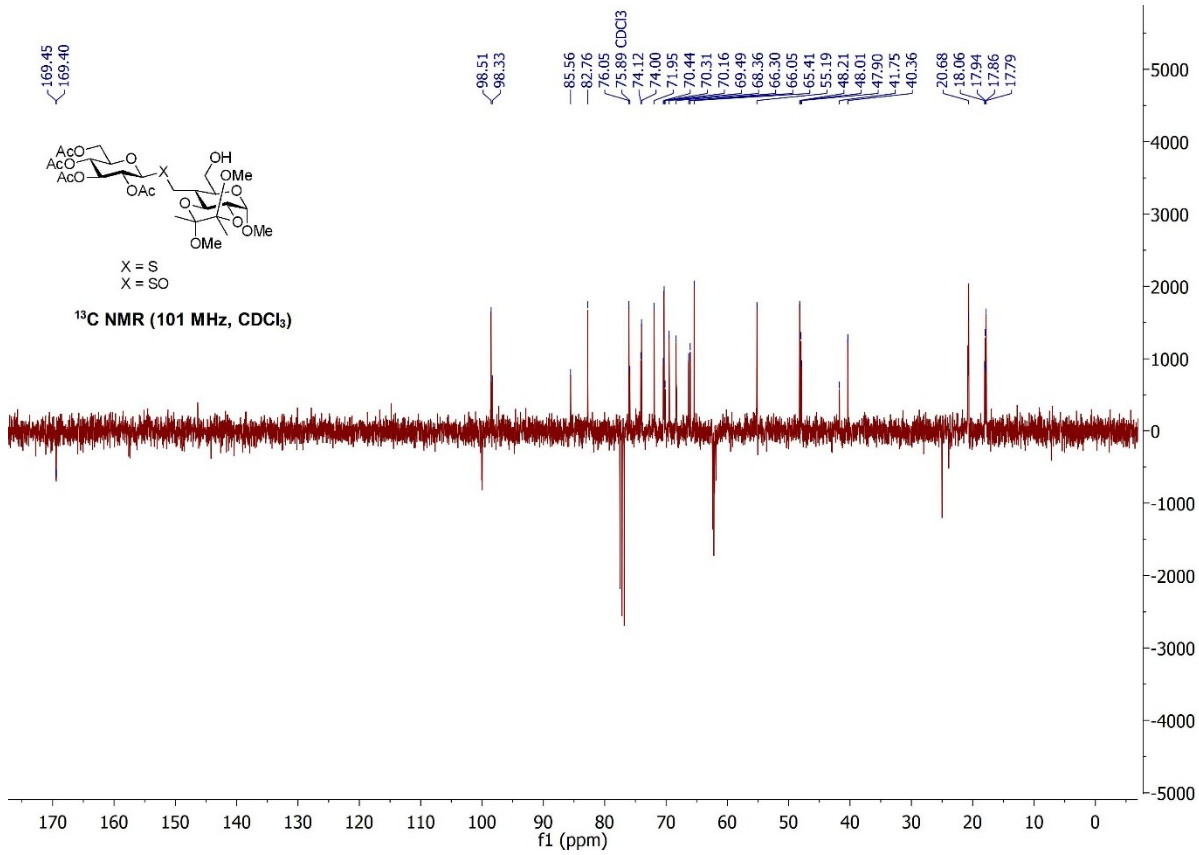
169.45
169.40
98.51
98.33
85.56
82.76
76.05
75.89 CDCl3
74.12
74.00
71.95
70.44
70.31
70.16
69.49
68.36
66.30
66.05
65.41
55.19
48.21
48.01
47.90
41.75
40.36
20.68
18.06
17.94
17.86
17.79
X = S
X = SO
13C NMR (101 MHz, CDCl3)
f1 (ppm)

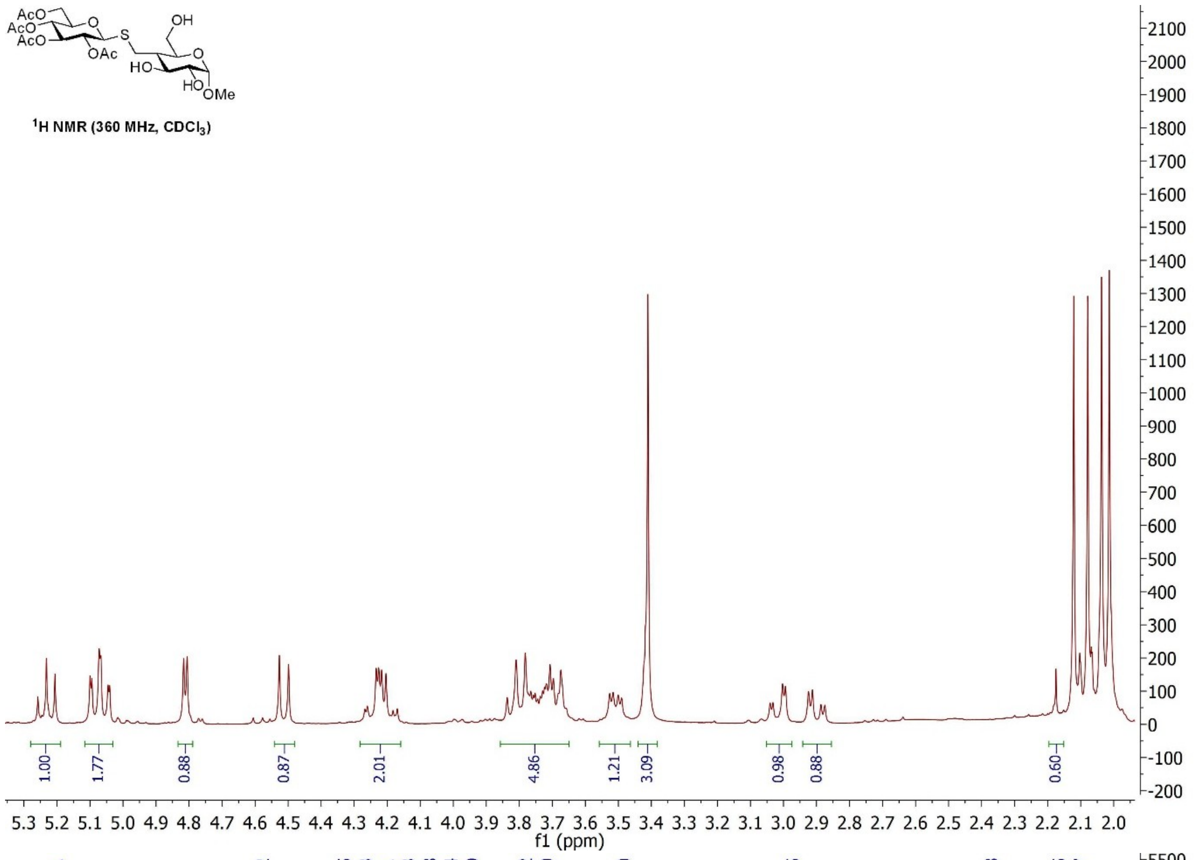

AcO
AcO
AcO
OAc
S
OH
HO
HO
OMe
1H NMR (360 MHz, CDCl3)
1.00
1.77
0.88
0.87
2.01
4.86
1.21
3.09
0.98
0.88
0.60
f1 (ppm)

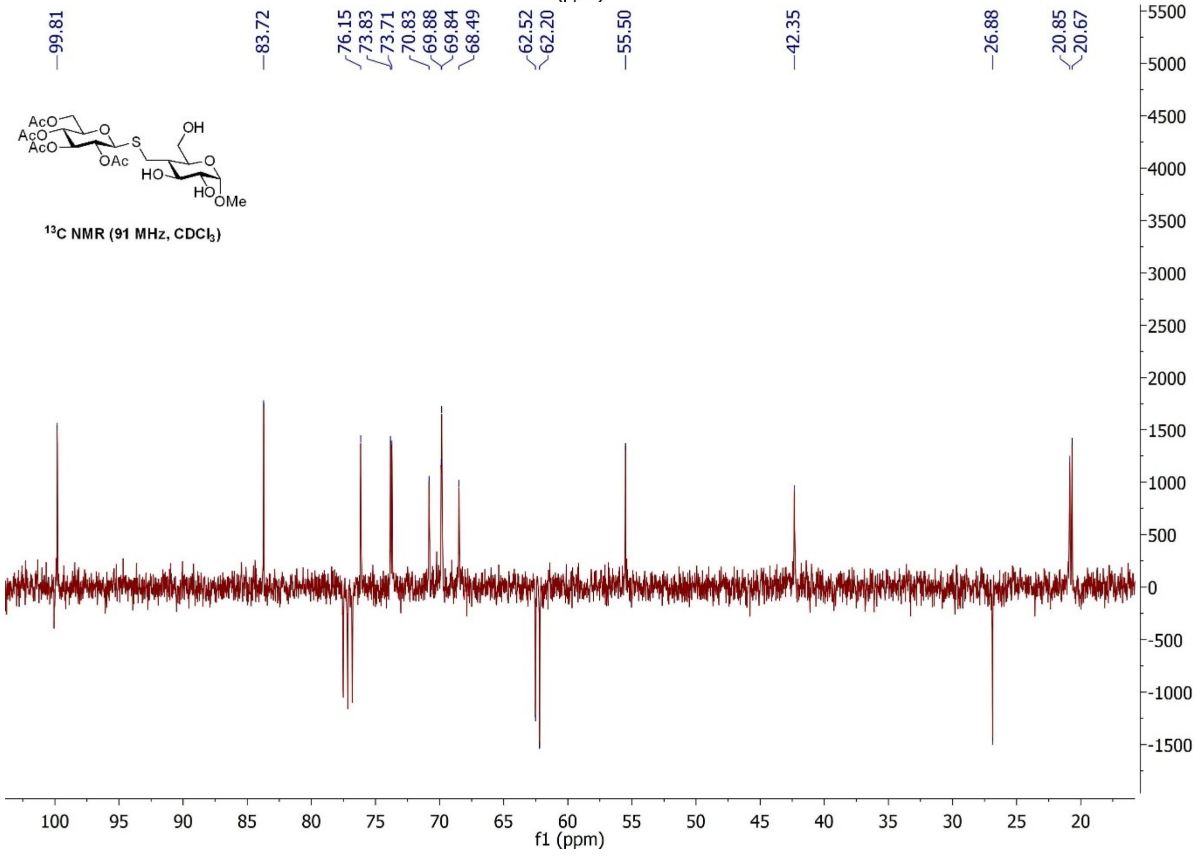

99.81
83.72
76.15
73.83
73.71
70.83
69.88
69.84
68.49
62.52
62.20
55.50
42.35
26.88
20.85
20.67
AcO
AcO
AcO
OAc
S
OH
HO
HO
OMe
13C NMR (91 MHz, CDCl3)
f1 (ppm)

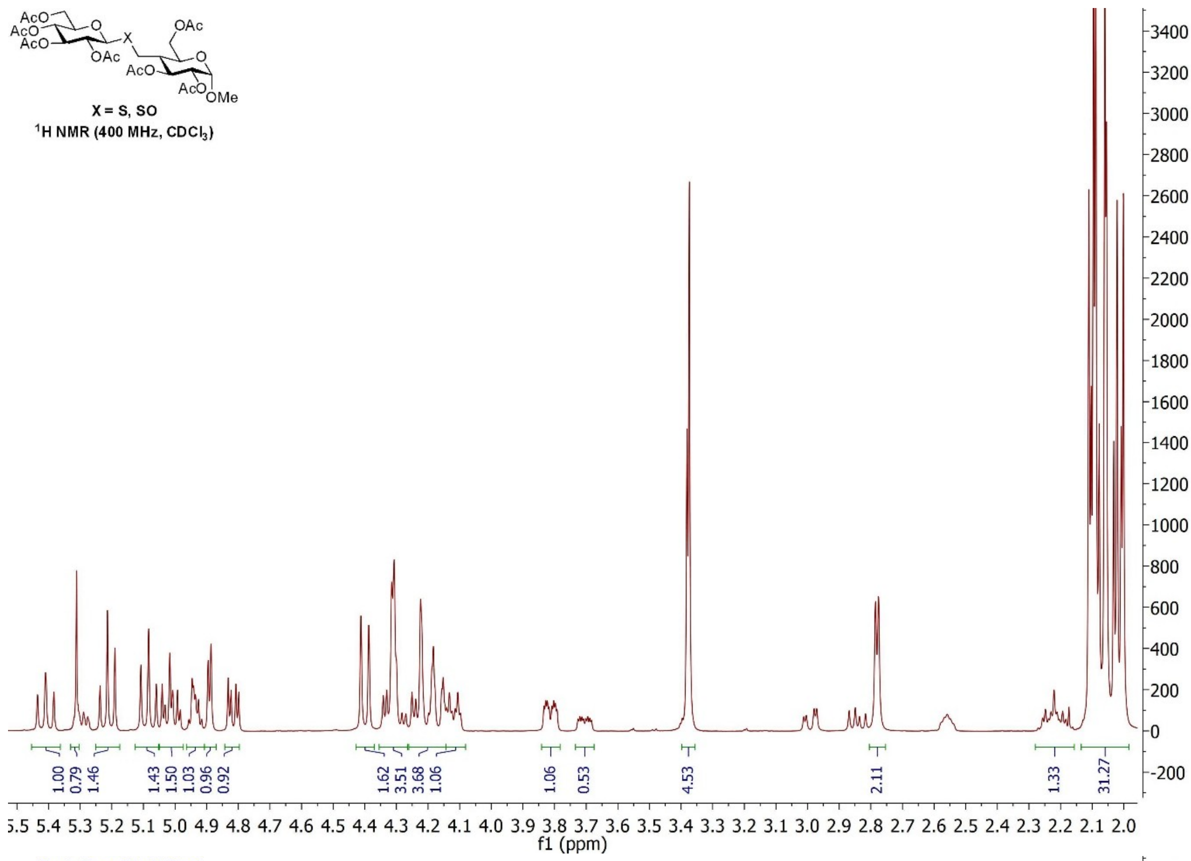
X = S, SO
¹H NMR (400 MHz, CDCl₃)
f1 (ppm)

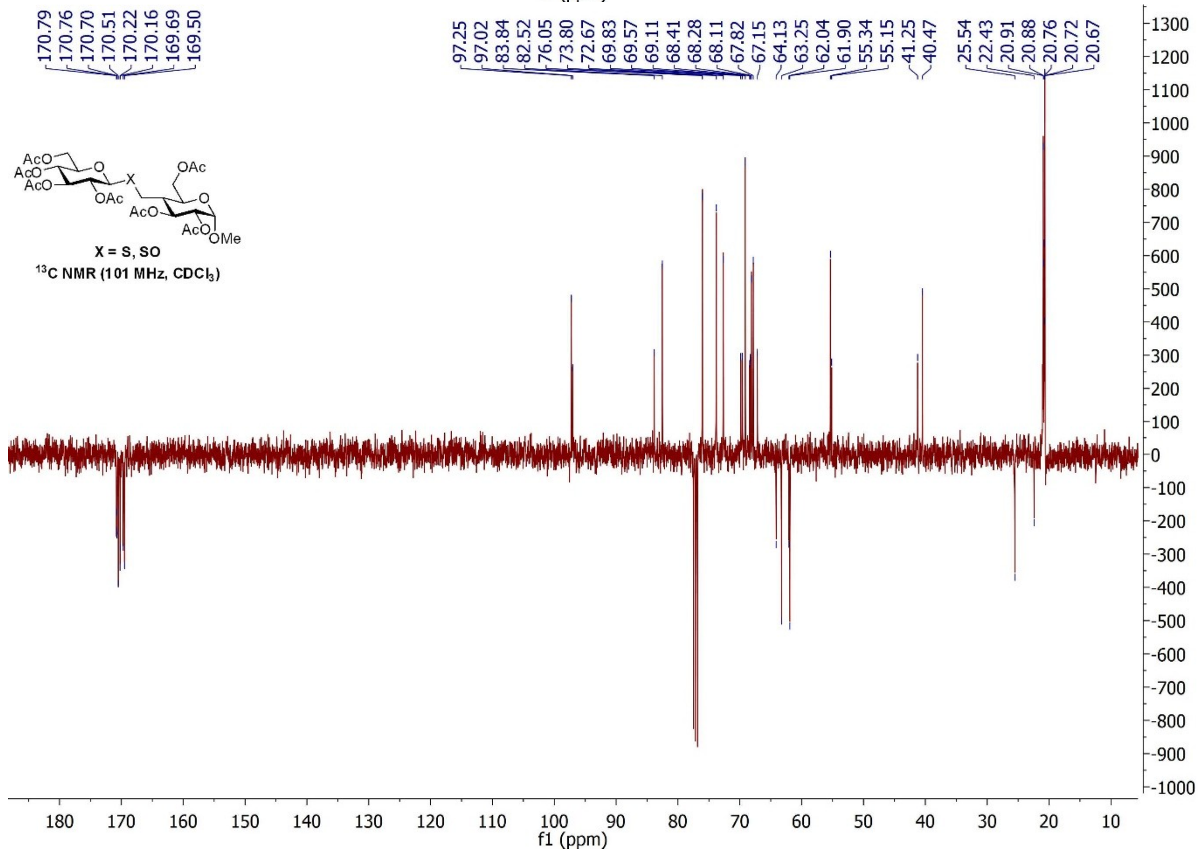
X = S, SO
¹³C NMR (101 MHz, CDCl₃)
f1 (ppm)

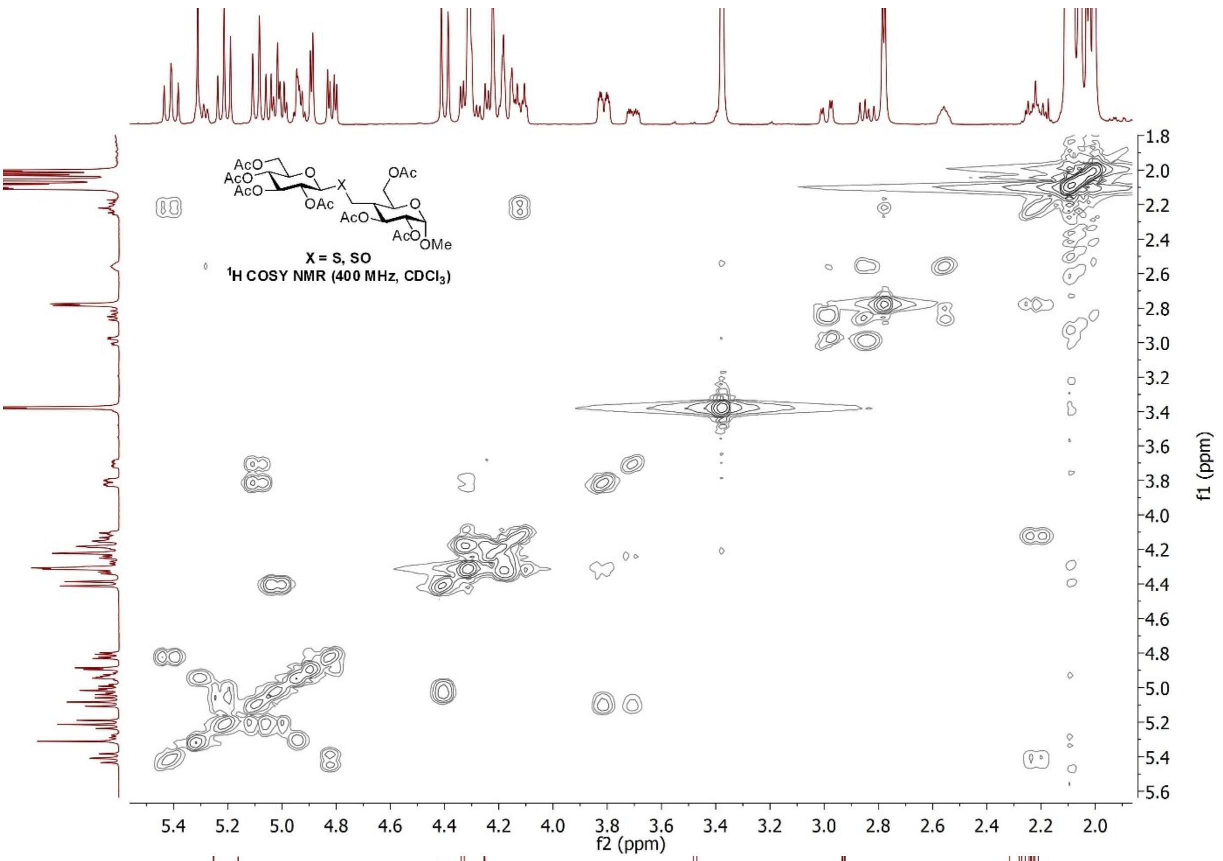
AcO
AcO
AcO
X
OAc
OAc
AcO
AcO
OMe
X = S, SO
1H COSY NMR (400 MHz, CDCl3)
f2 (ppm)
f1 (ppm)

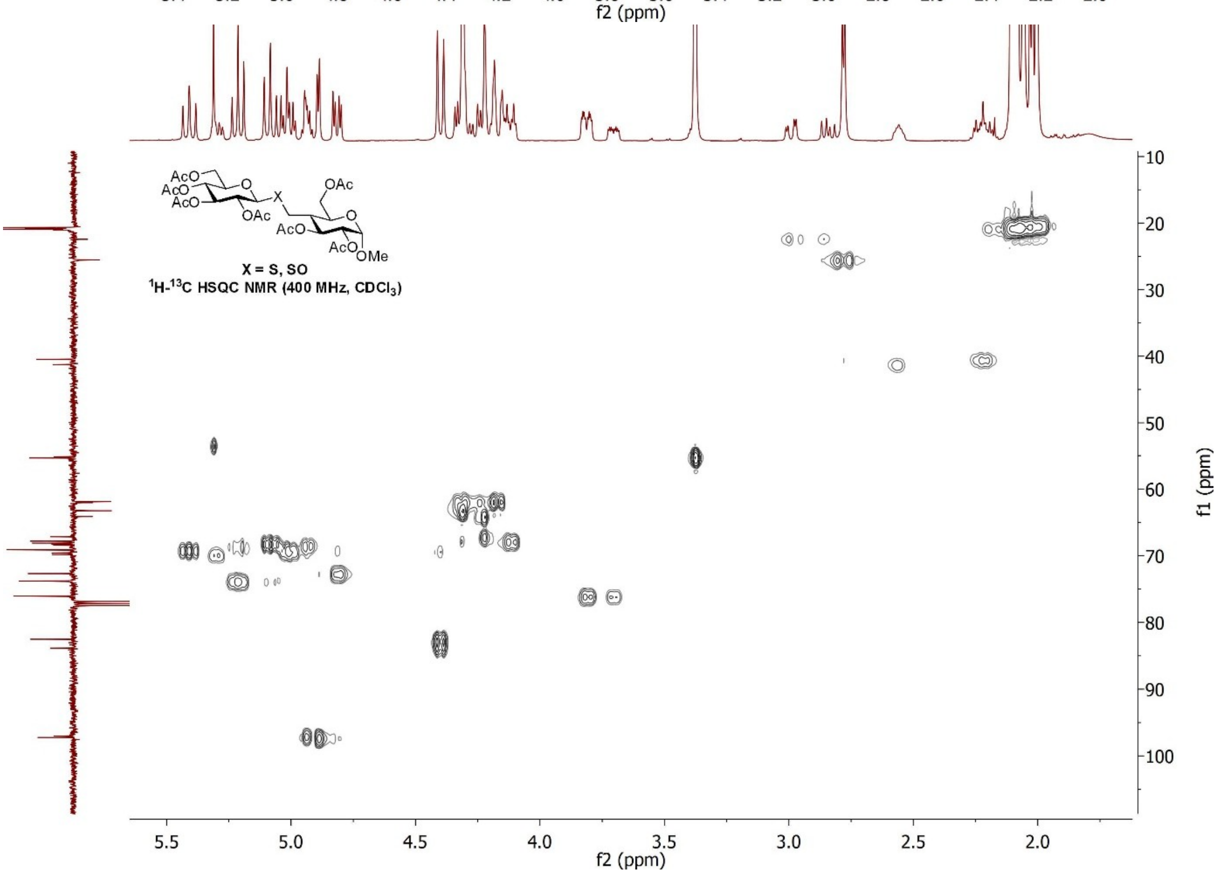
AcO
AcO
AcO
X
OAc
OAc
AcO
AcO
OMe
X = S, SO
1H-13C HSQC NMR (400 MHz, CDCl3)
f2 (ppm)
f1 (ppm)

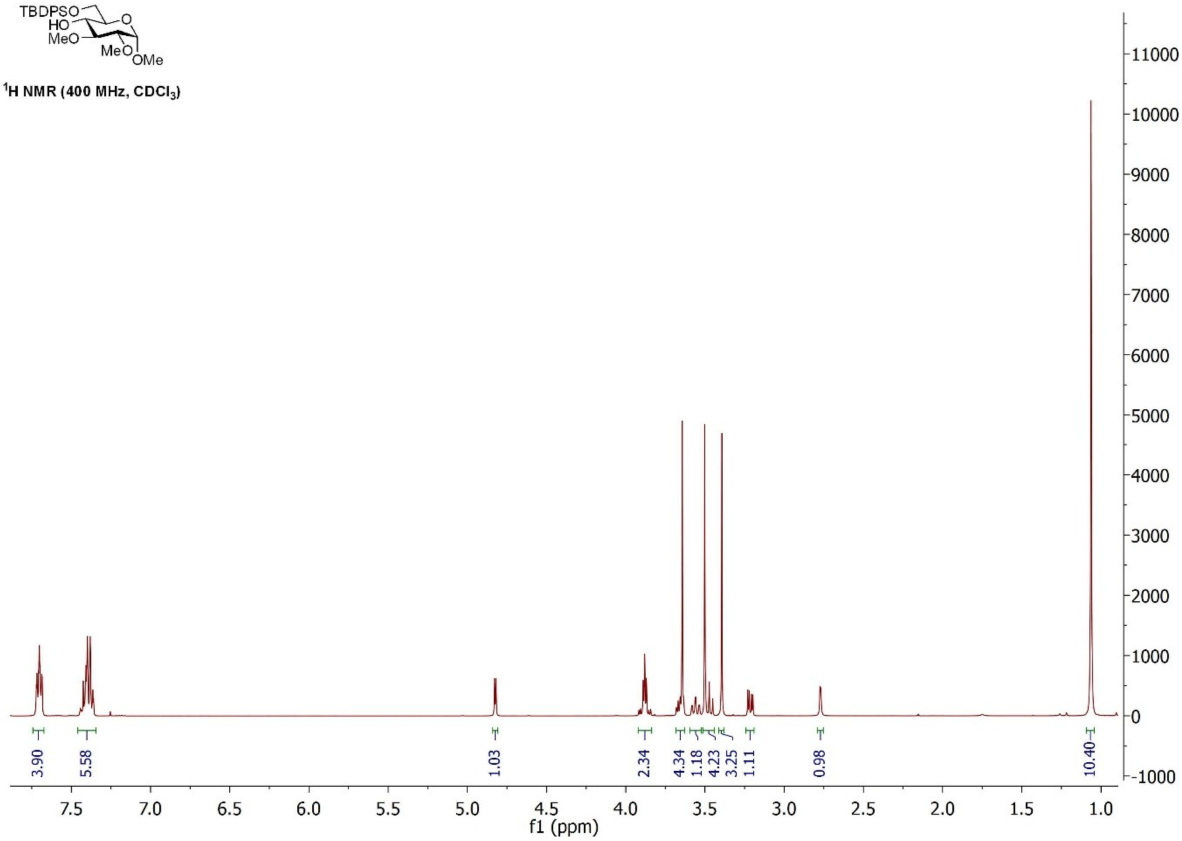
TBDPSO
HO
MeO
MeO
OMe
O
^{1}H NMR (400 MHz, CDCl$_3$)
3.90
5.58
1.03
2.34
4.34
1.18
4.23
3.25
1.11
0.98
10.40
f1 (ppm)

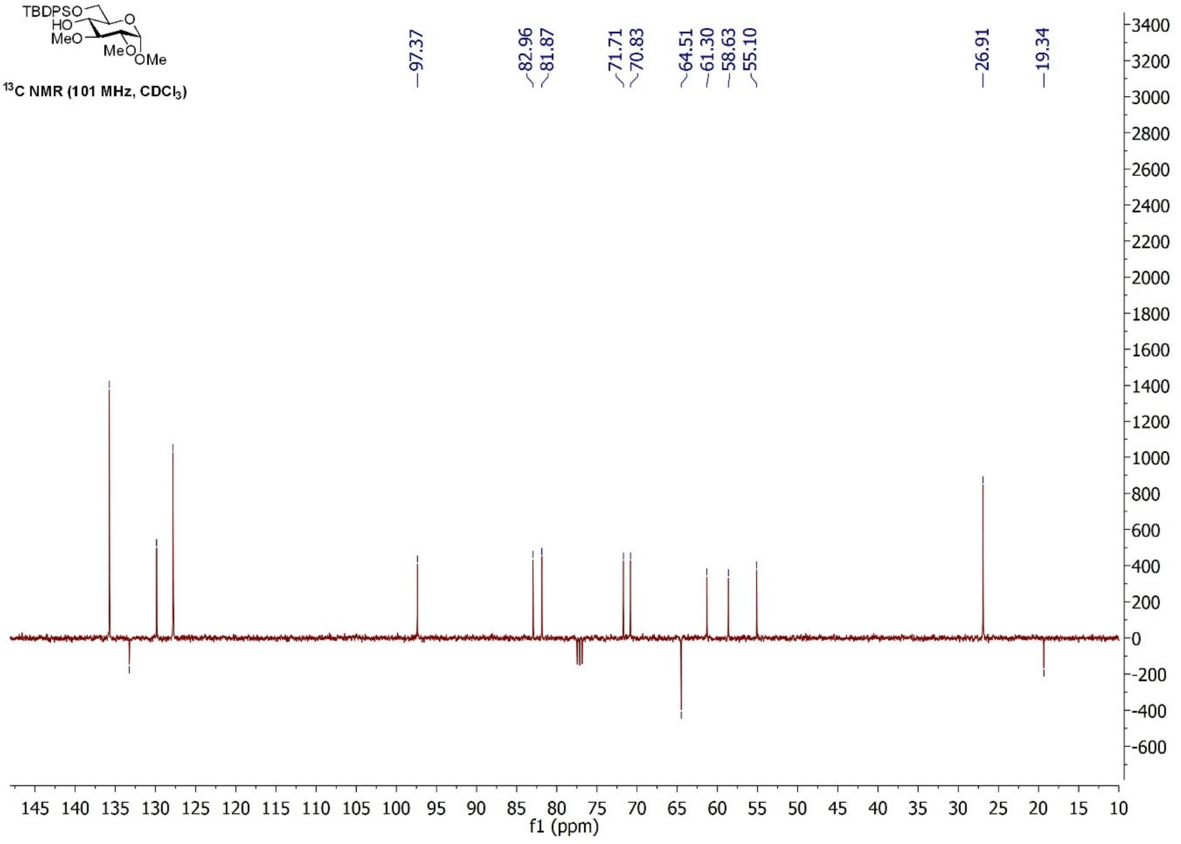
TBDPSO
HO
MeO
MeO
OMe
O
^{13}C NMR (101 MHz, CDCl$_3$)
97.37
82.96
81.87
71.71
70.83
64.51
61.30
58.63
55.10
26.91
19.34
f1 (ppm)

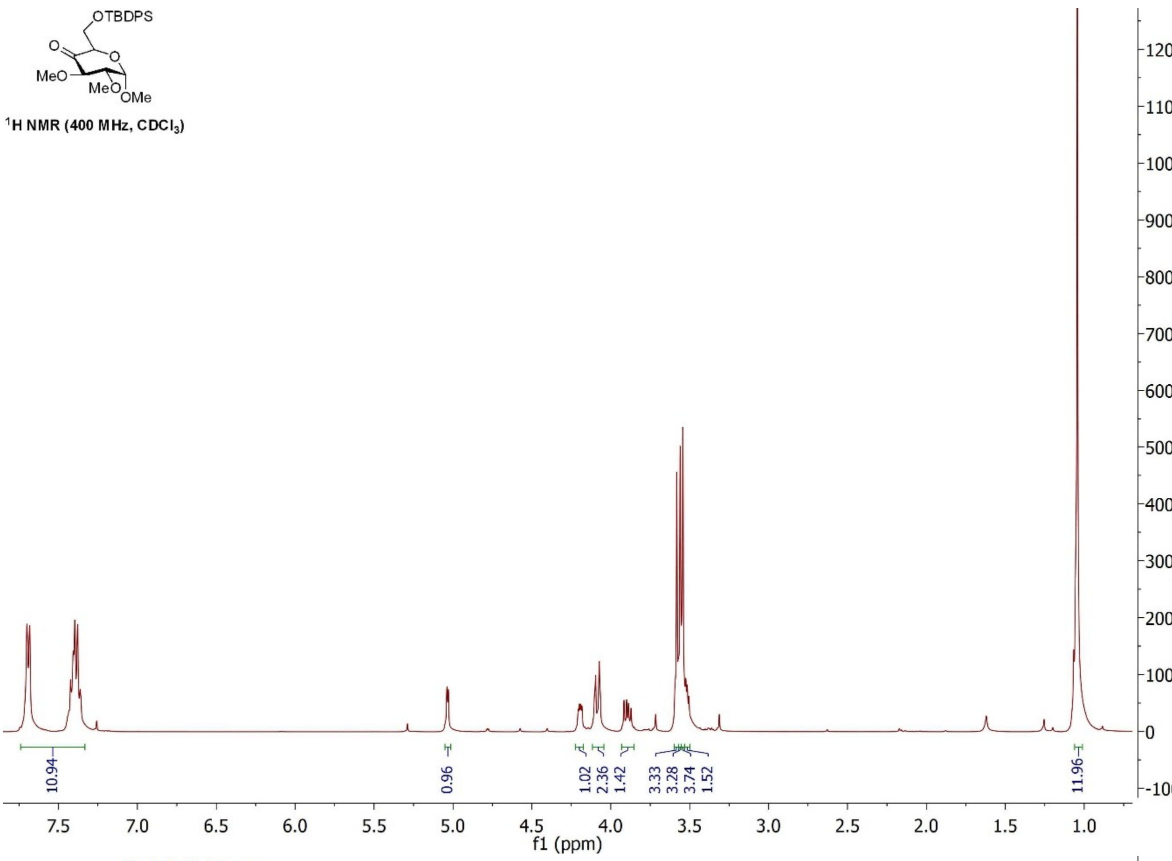

OTBDPS
O
MeO
MeO
OMe
O
1H NMR (400 MHz, CDCl3)
10.94
0.96
1.02
2.36
1.42
3.33
3.28
3.74
1.52
11.96
f1 (ppm)

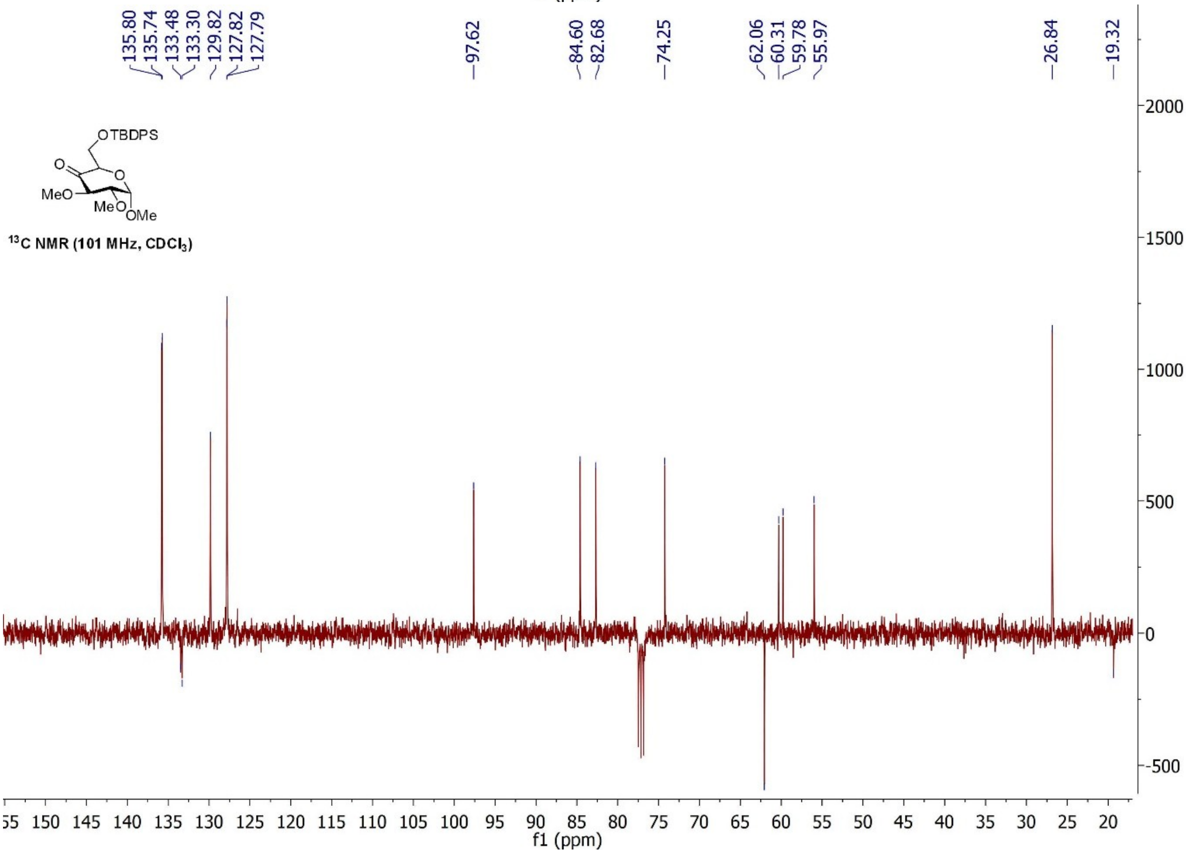

135.80
135.74
133.48
133.30
129.82
127.82
127.79
97.62
84.60
82.68
74.25
62.06
60.31
59.78
55.97
26.84
19.32
OTBDPS
O
MeO
MeO
OMe
O
13C NMR (101 MHz, CDCl3)
f1 (ppm)

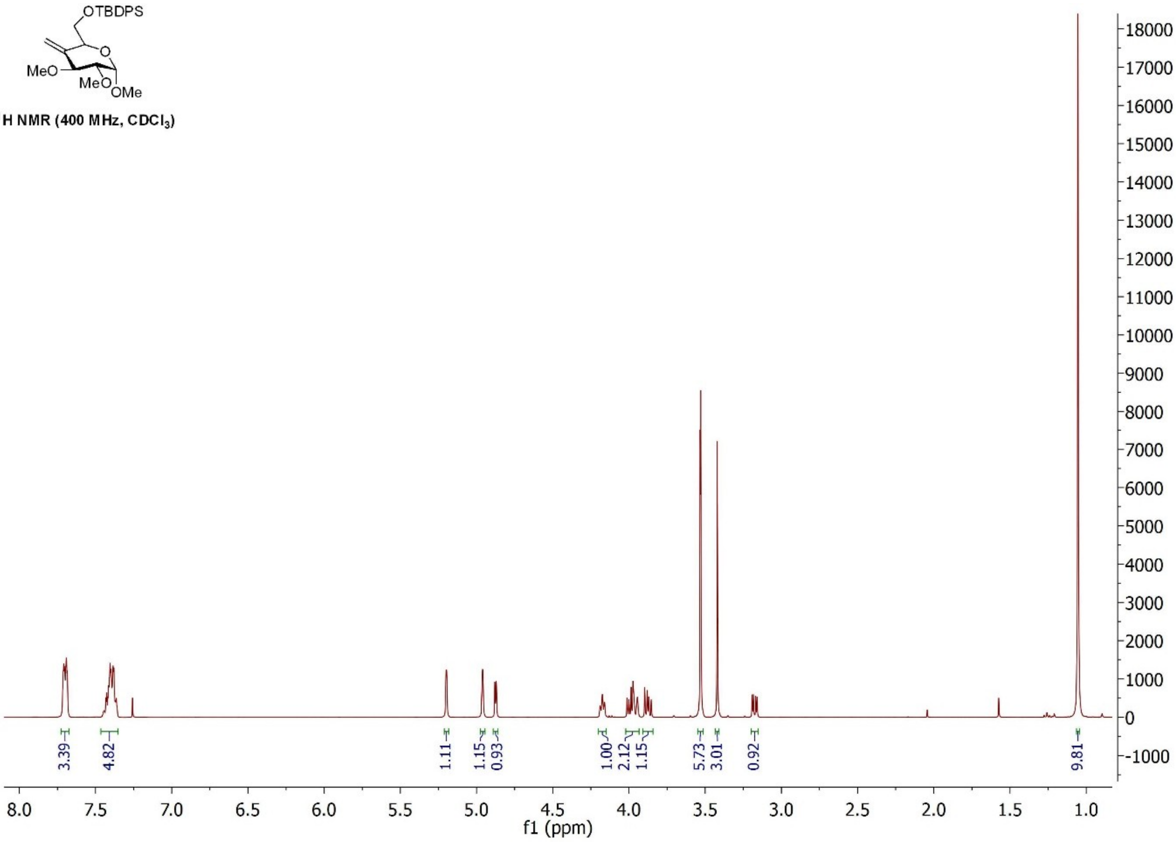

^{1}H NMR (400 MHz, CDCl$_3$)

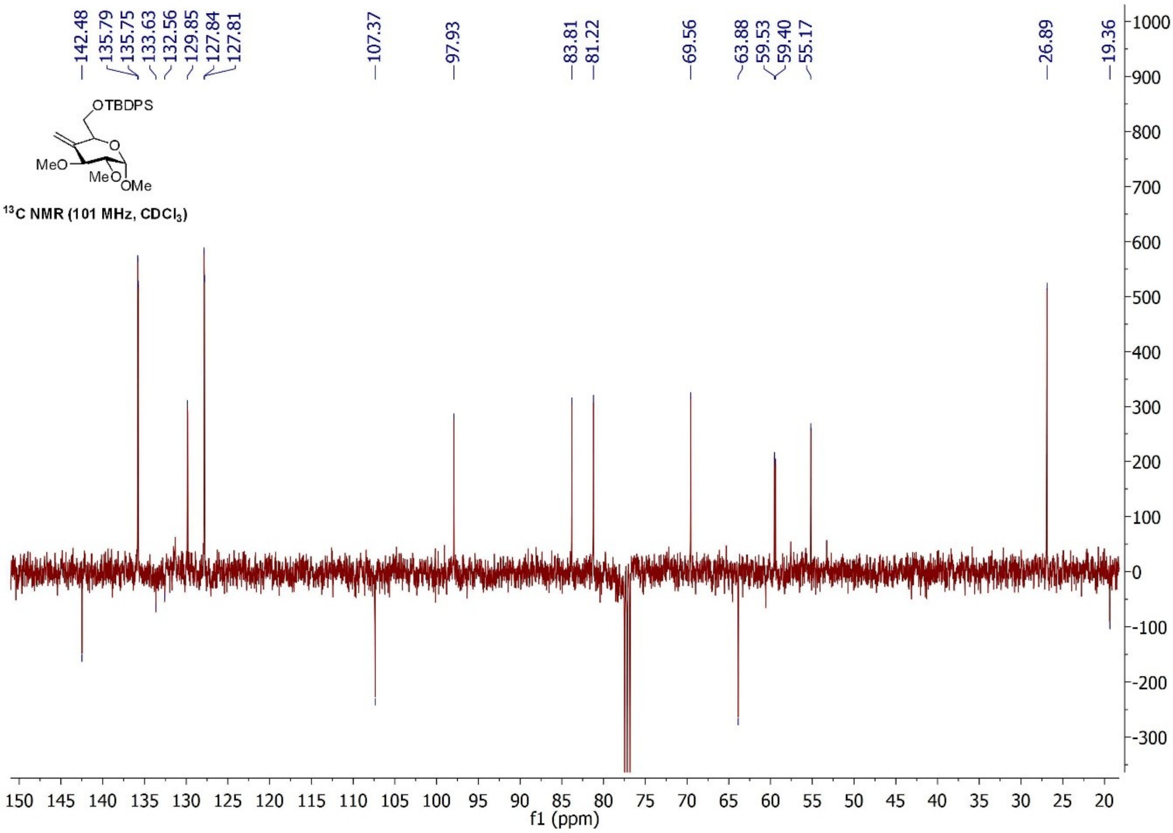

^{13}C NMR (101 MHz, CDCl$_3$)

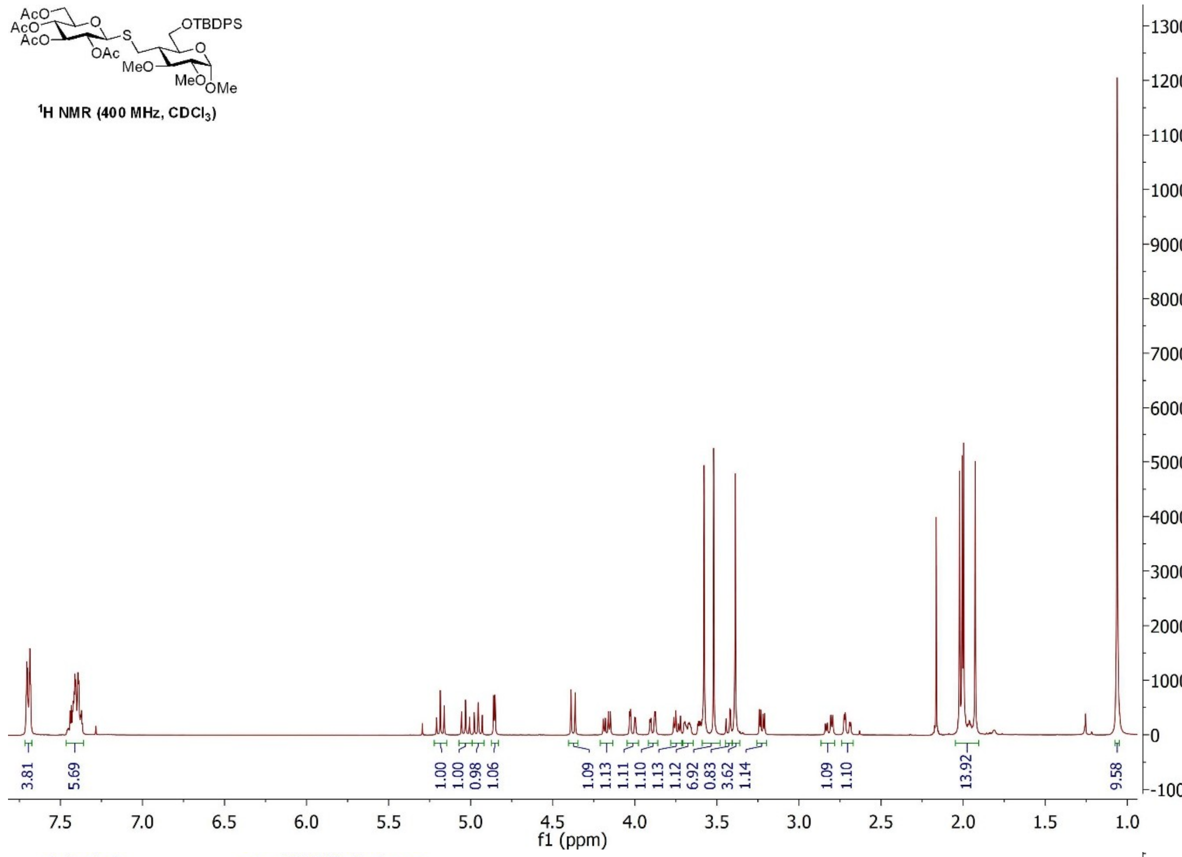
AcO
AcO
AcO
OAc
S
OTBDPS
MeO
MeO
OMe
^{1}H NMR (400 MHz, CDCl$_3$)
3.81
5.69
1.00
1.00
0.98
1.06
1.09
1.13
1.11
1.10
1.13
1.12
6.92
0.83
3.62
1.14
1.09
1.10
13.92
9.58
f1 (ppm)

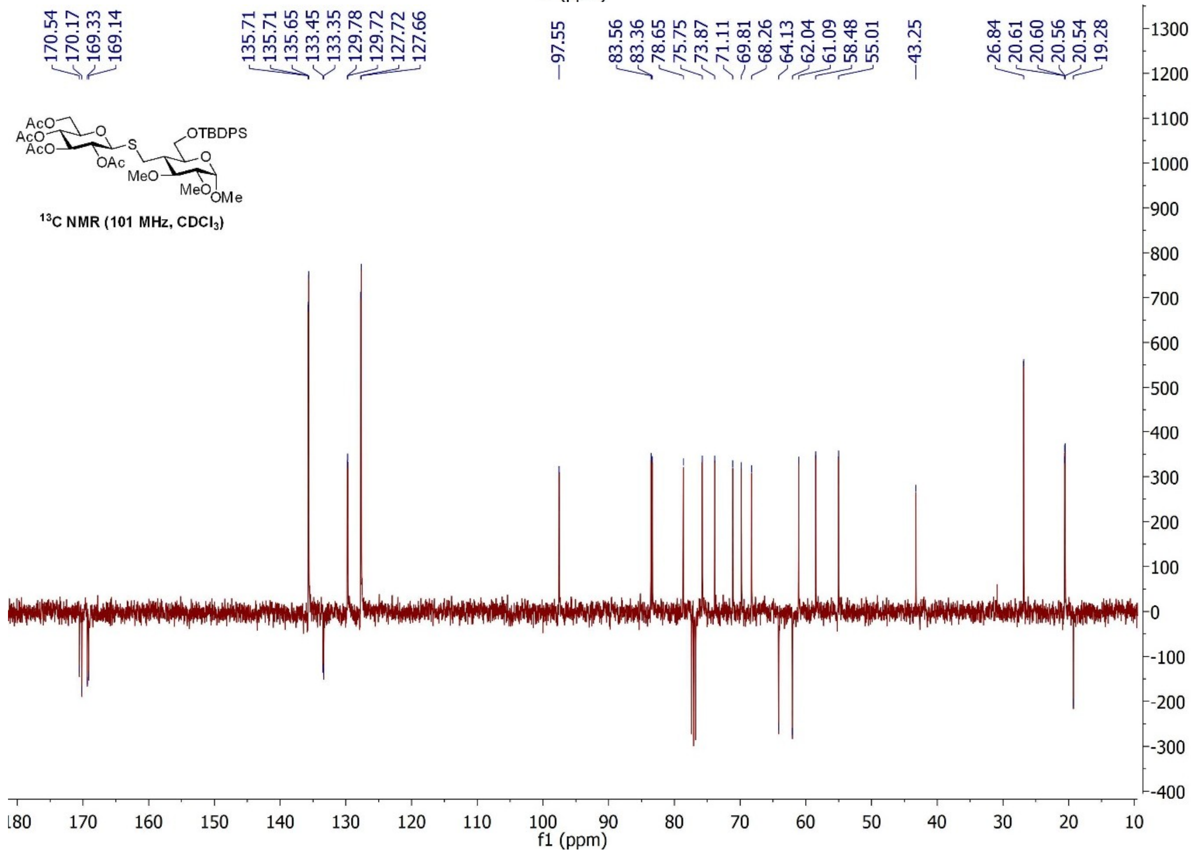
170.54
170.17
169.33
169.14
135.71
135.71
135.65
133.45
133.35
129.78
129.72
127.72
127.66
97.55
83.56
83.36
78.65
75.75
73.87
71.11
69.81
68.26
64.13
62.04
61.09
58.48
55.01
43.25
26.84
20.61
20.60
20.56
20.54
19.28
AcO
AcO
AcO
OAc
S
OTBDPS
MeO
MeO
OMe
^{13}C NMR (101 MHz, CDCl$_3$)
f1 (ppm)

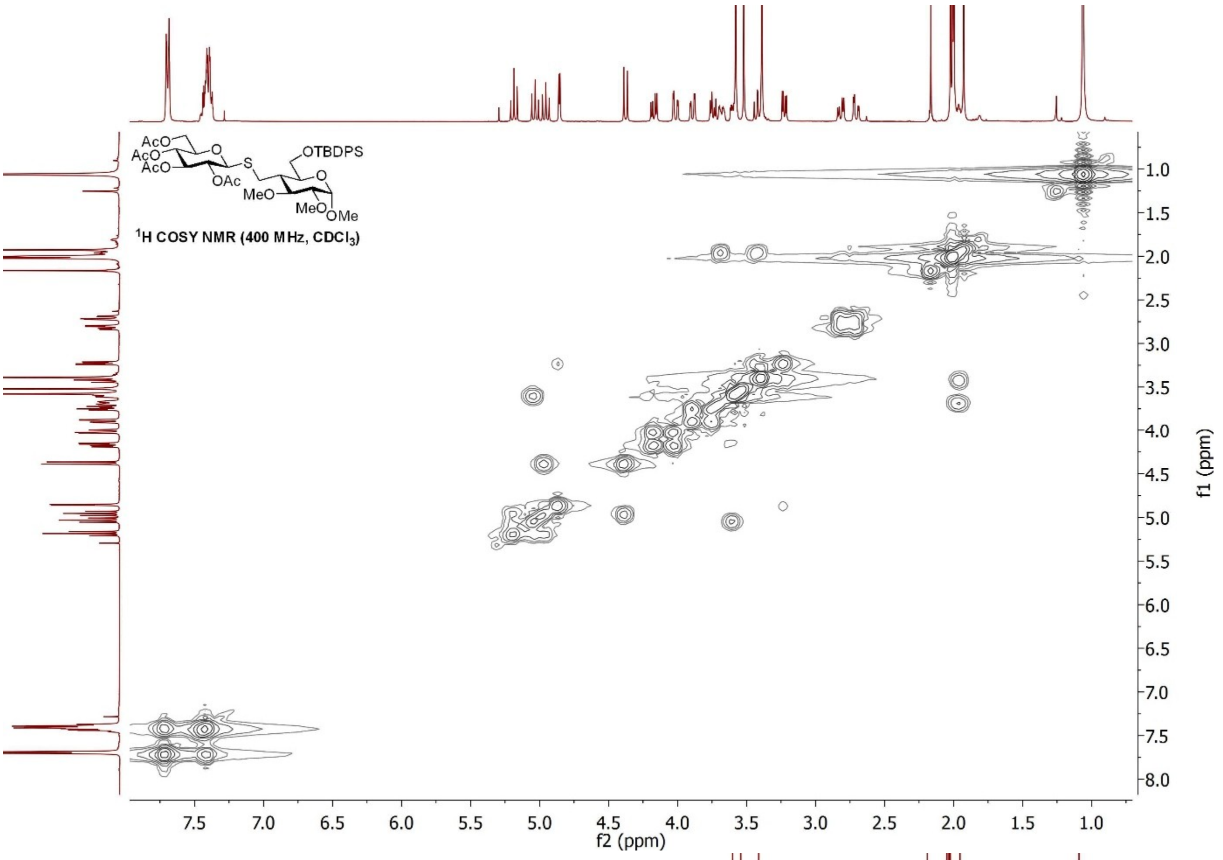
AcO
AcO
AcO
OAc
S
OTBDPS
MeO
MeO
OMe
^{1}H COSY NMR (400 MHz, CDCl$_3$)
f1 (ppm)
f2 (ppm)

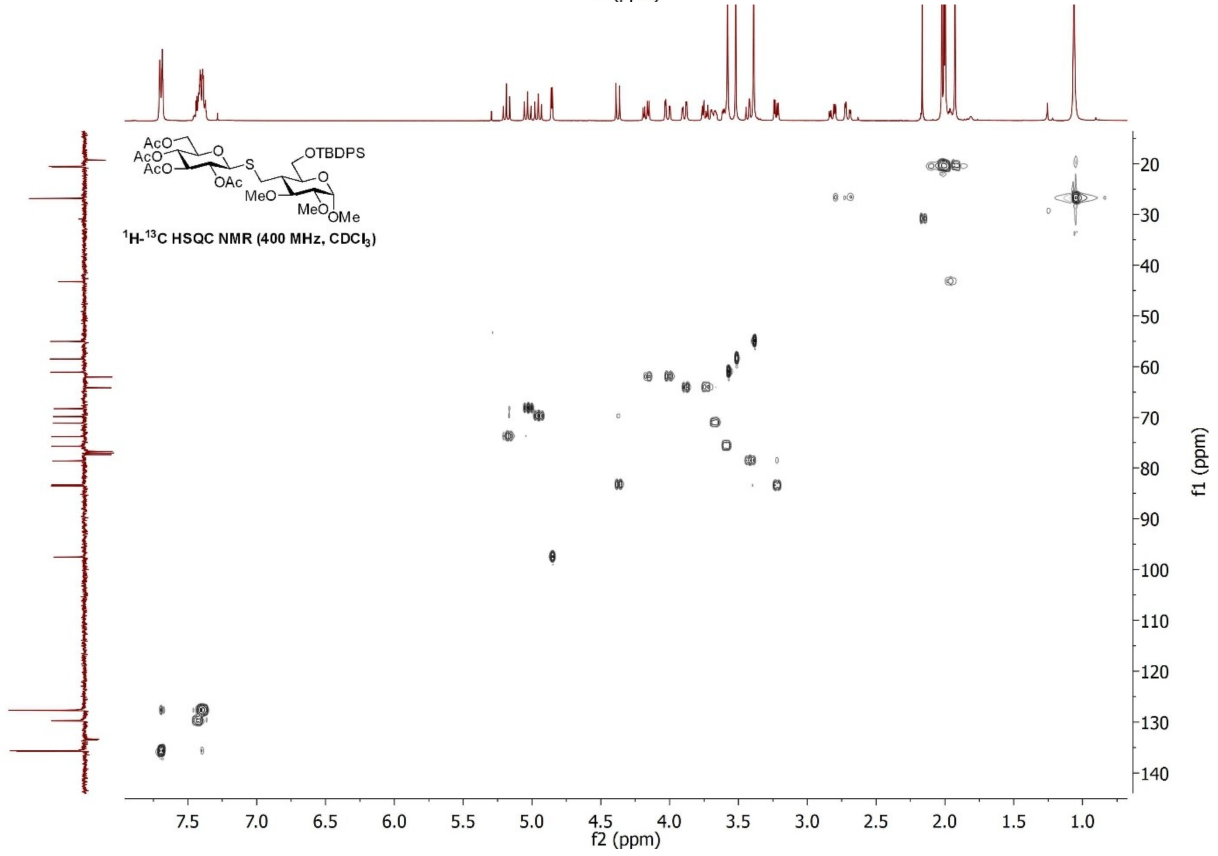
AcO
AcO
AcO
OAc
S
OTBDPS
MeO
MeO
OMe
^{1}H-^{13}C HSQC NMR (400 MHz, CDCl$_3$)
f1 (ppm)
f2 (ppm)

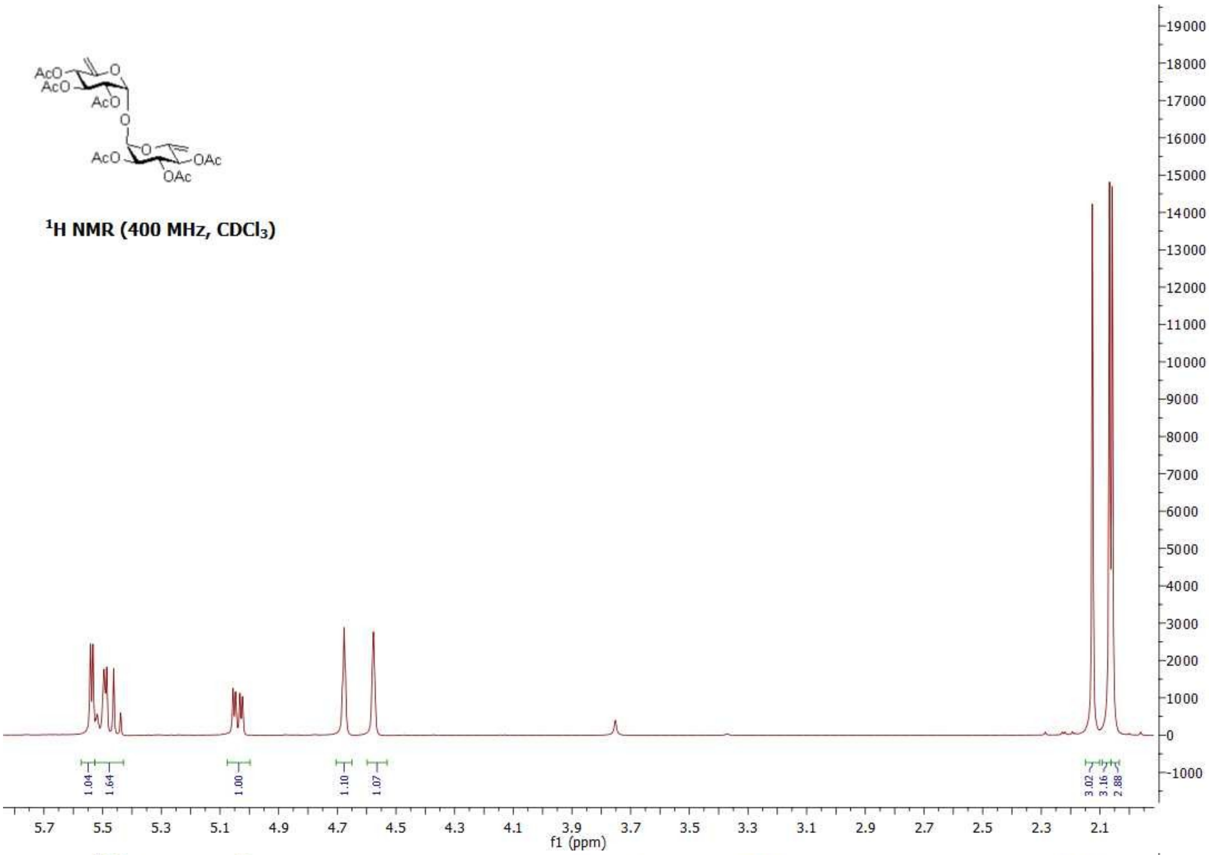
AcO
AcO
AcO
O
AcO
OAc
OAc
¹H NMR (400 MHz, CDCl₃)
1.04
1.64
1.00
1.10
1.07
3.02
3.16
2.88
f1 (ppm)

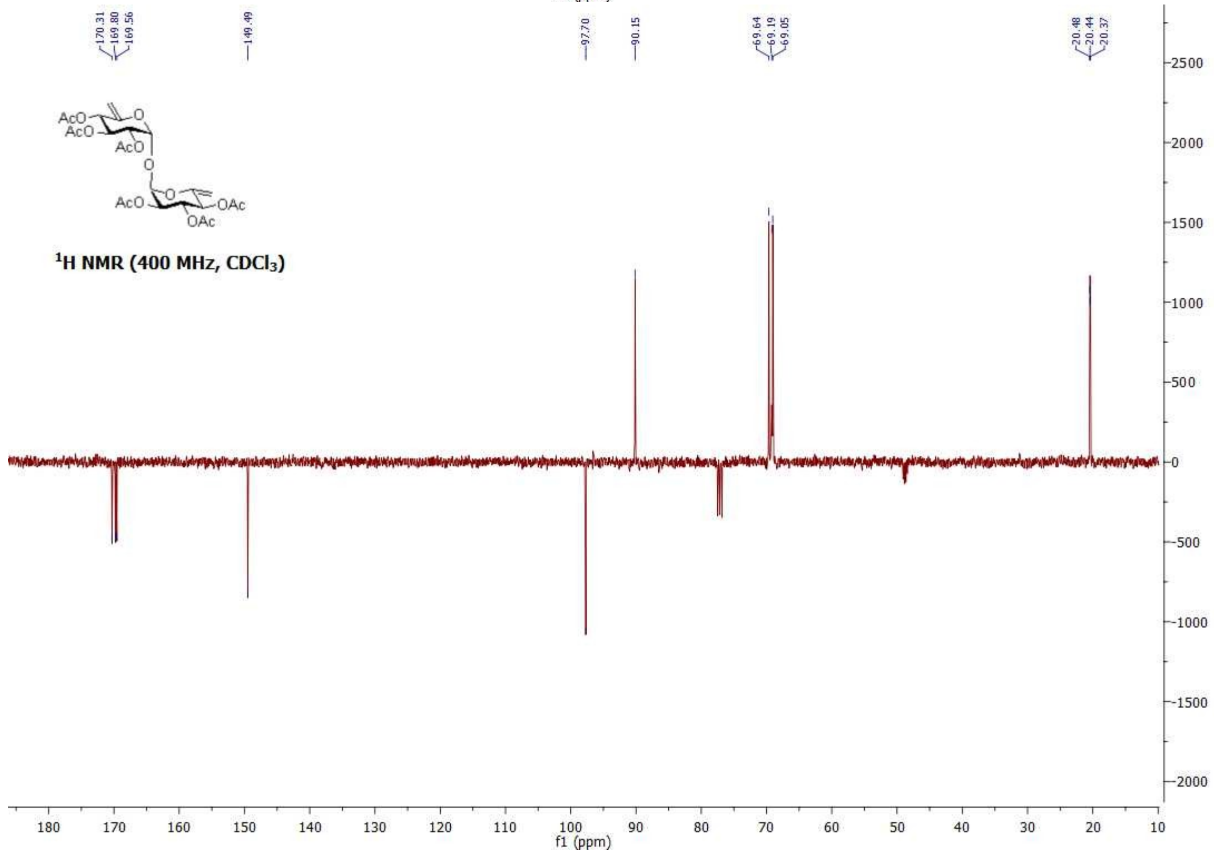
170.31
169.80
169.56
149.49
97.70
90.15
69.64
69.19
69.05
20.48
20.44
20.37
AcO
AcO
AcO
O
AcO
OAc
OAc
¹H NMR (400 MHz, CDCl₃)
f1 (ppm)

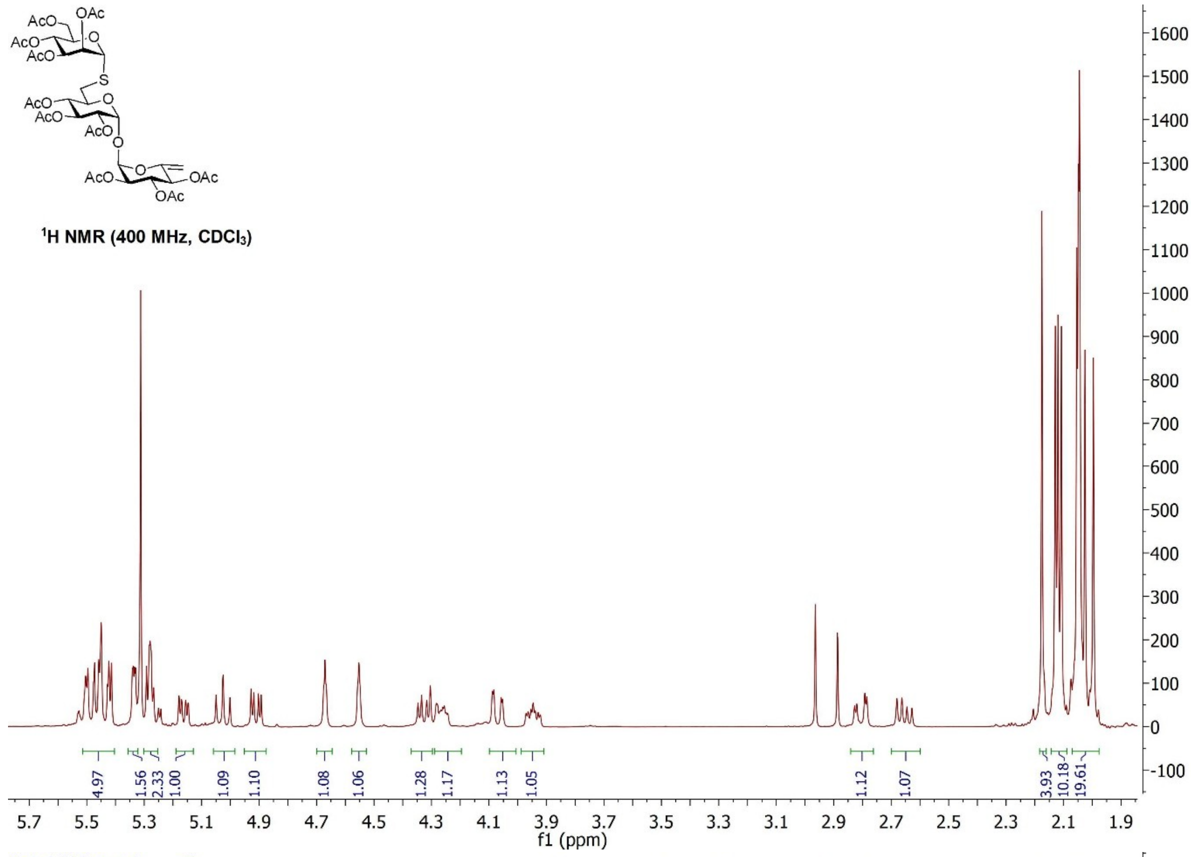

^{1}H NMR (400 MHz, $CDCl_3$)

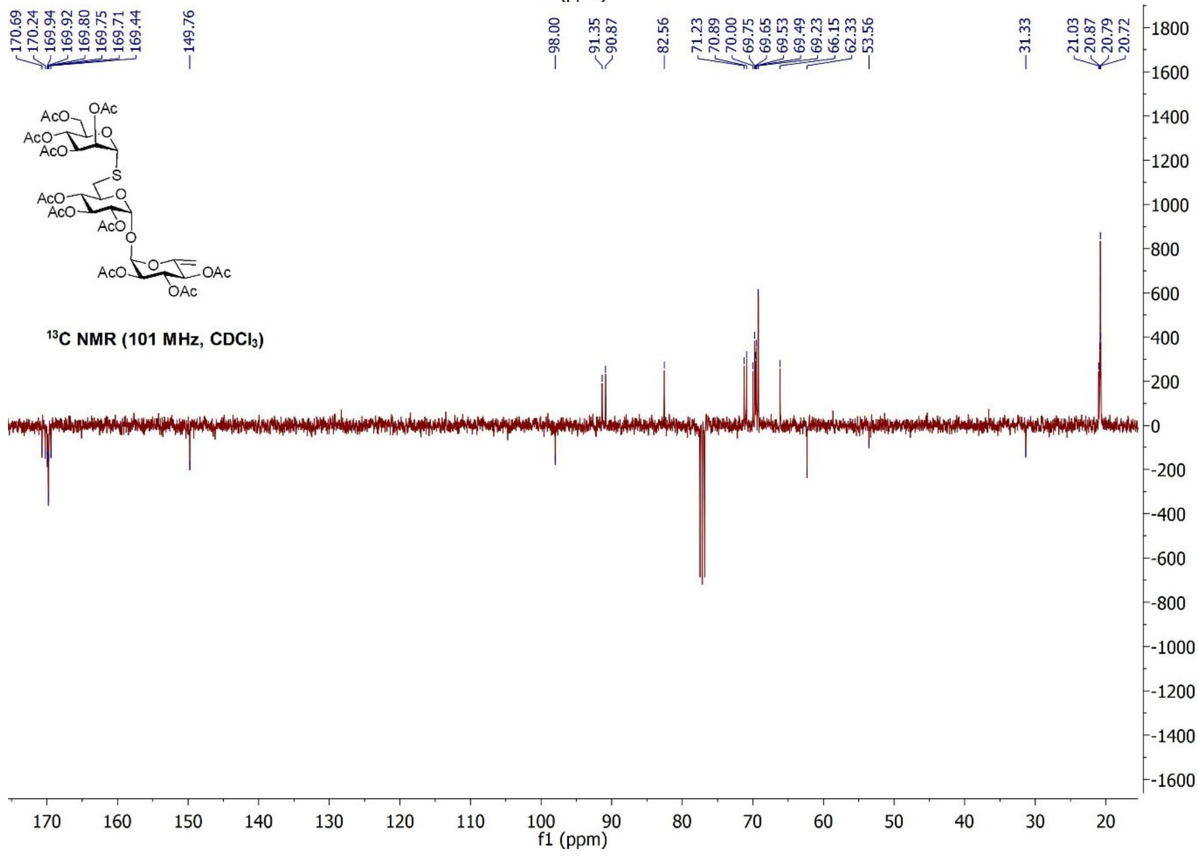

^{13}C NMR (101 MHz, $CDCl_3$)

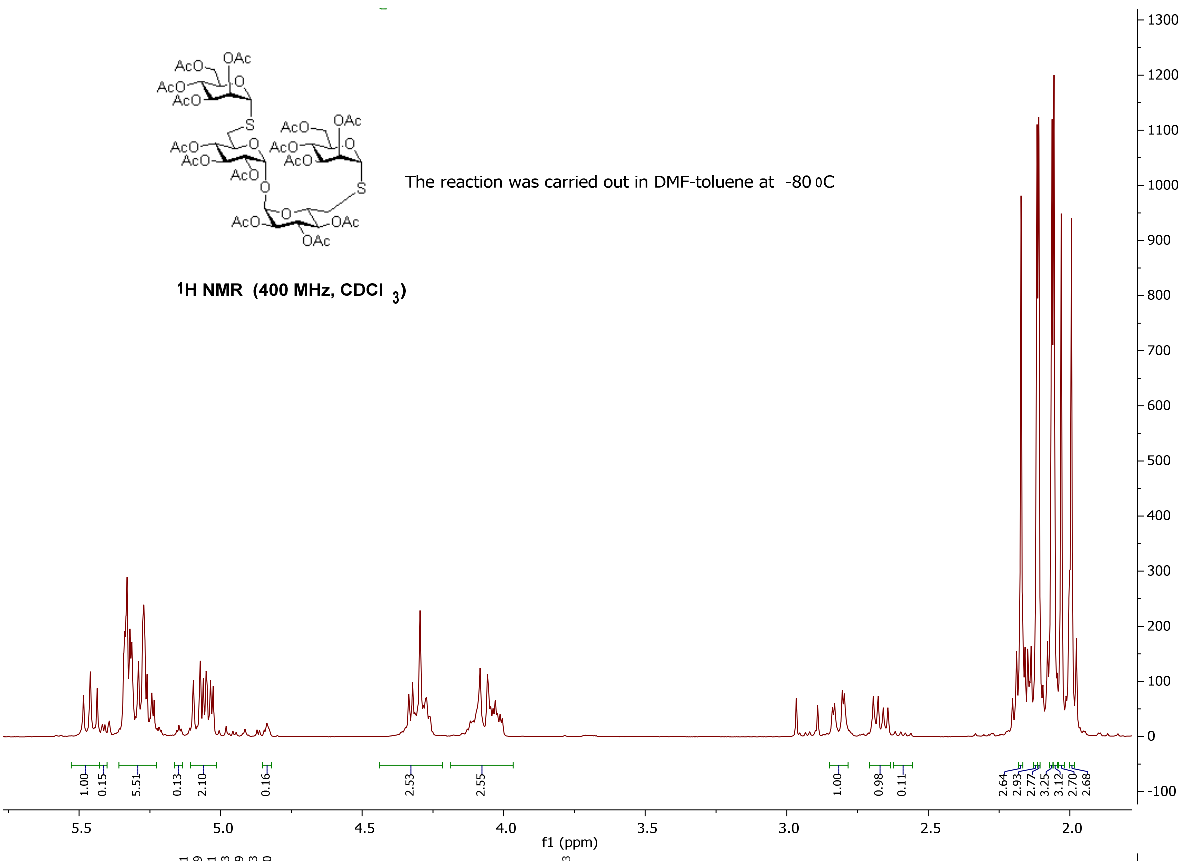
The reaction was carried out in DMF-toluene at -80 0C
1H NMR (400 MHz, CDCl 3)
1.00
0.15
5.51
0.13
2.10
0.16
2.53
2.55
1.00
0.98
0.11
2.64
2.93
2.77
3.25
3.12
2.70
2.68
f1 (ppm)

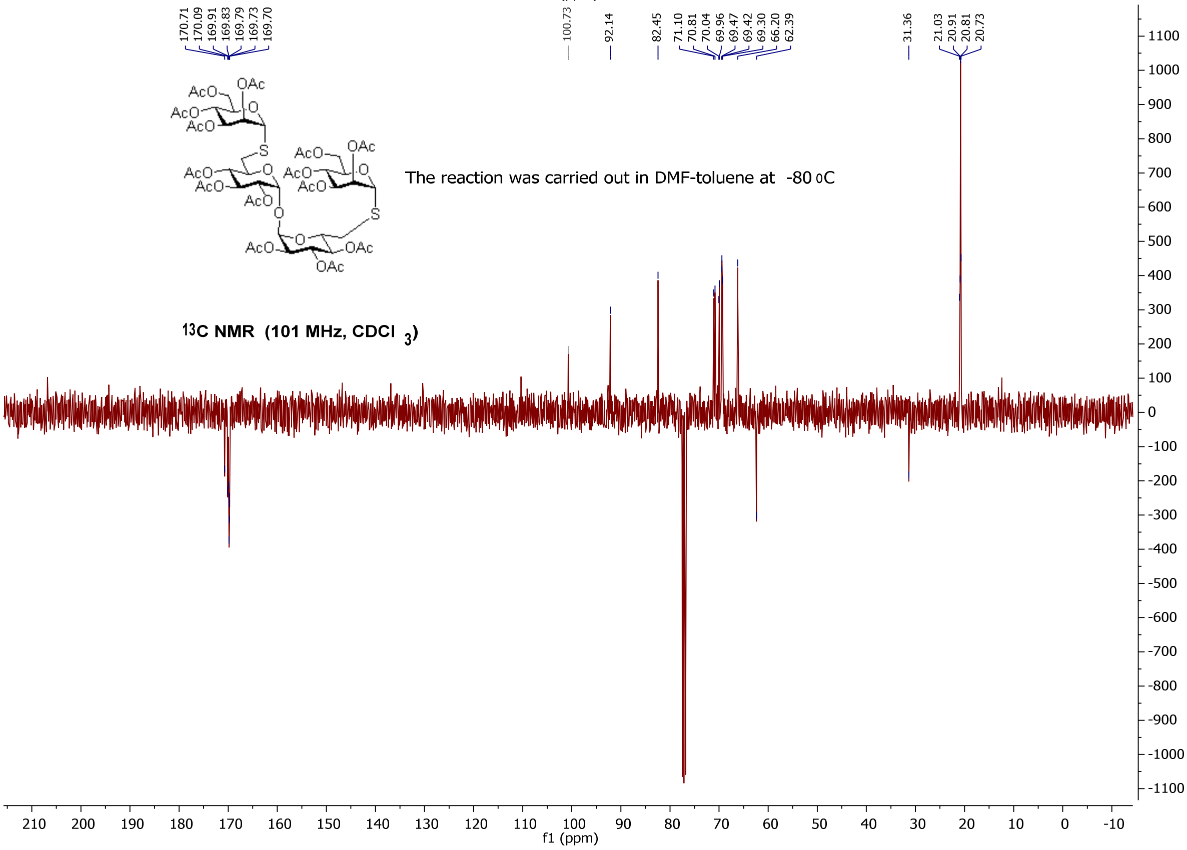
170.71
170.09
169.91
169.83
169.79
169.73
169.70
100.73
92.14
82.45
71.10
70.81
70.04
69.96
69.47
69.42
69.30
66.20
62.39
31.36
21.03
20.91
20.81
20.73
The reaction was carried out in DMF-toluene at -80 0C
13C NMR (101 MHz, CDCl 3)
f1 (ppm)

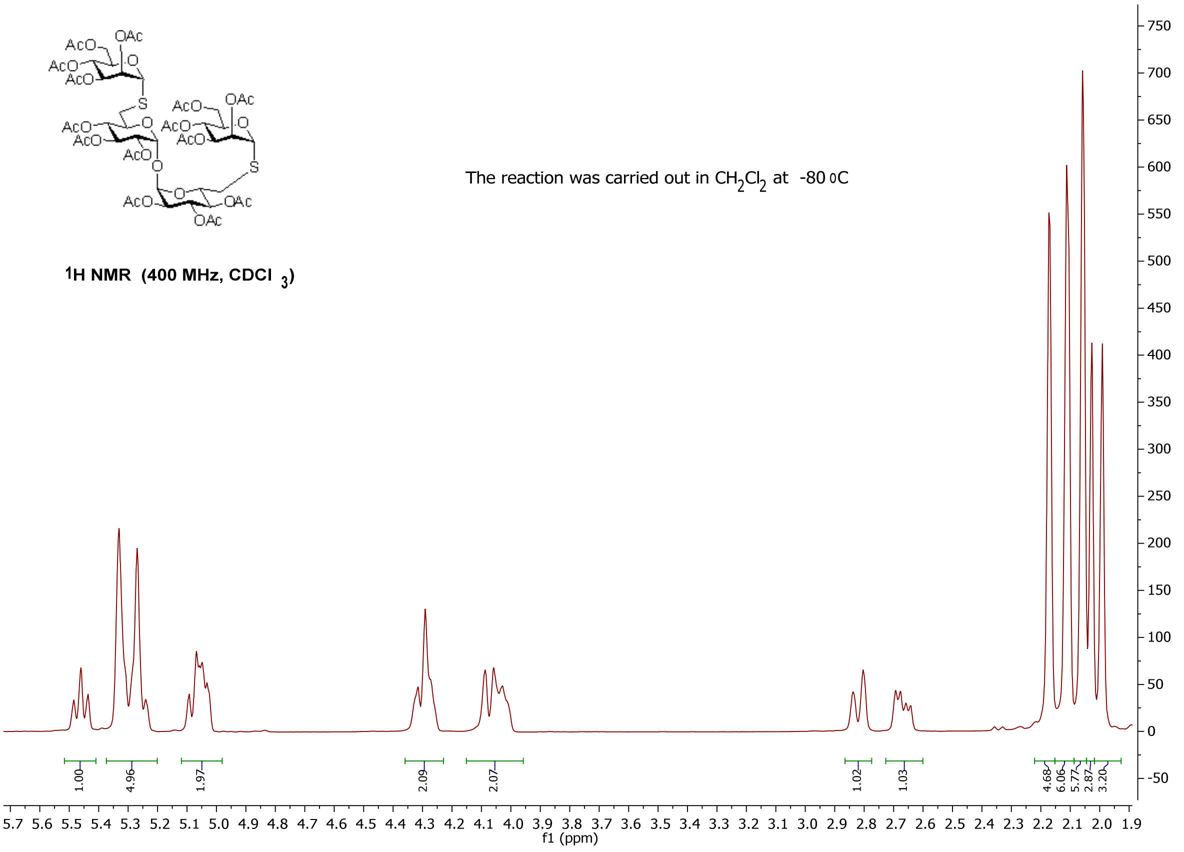

AcO
OAc
O
S
The reaction was carried out in CH_2Cl_2 at -80 0C
1H NMR (400 MHz, CDCl 3)
1.00
4.96
1.97
2.09
2.07
1.02
1.03
4.68
6.06
5.77
2.87
3.20
f1 (ppm)

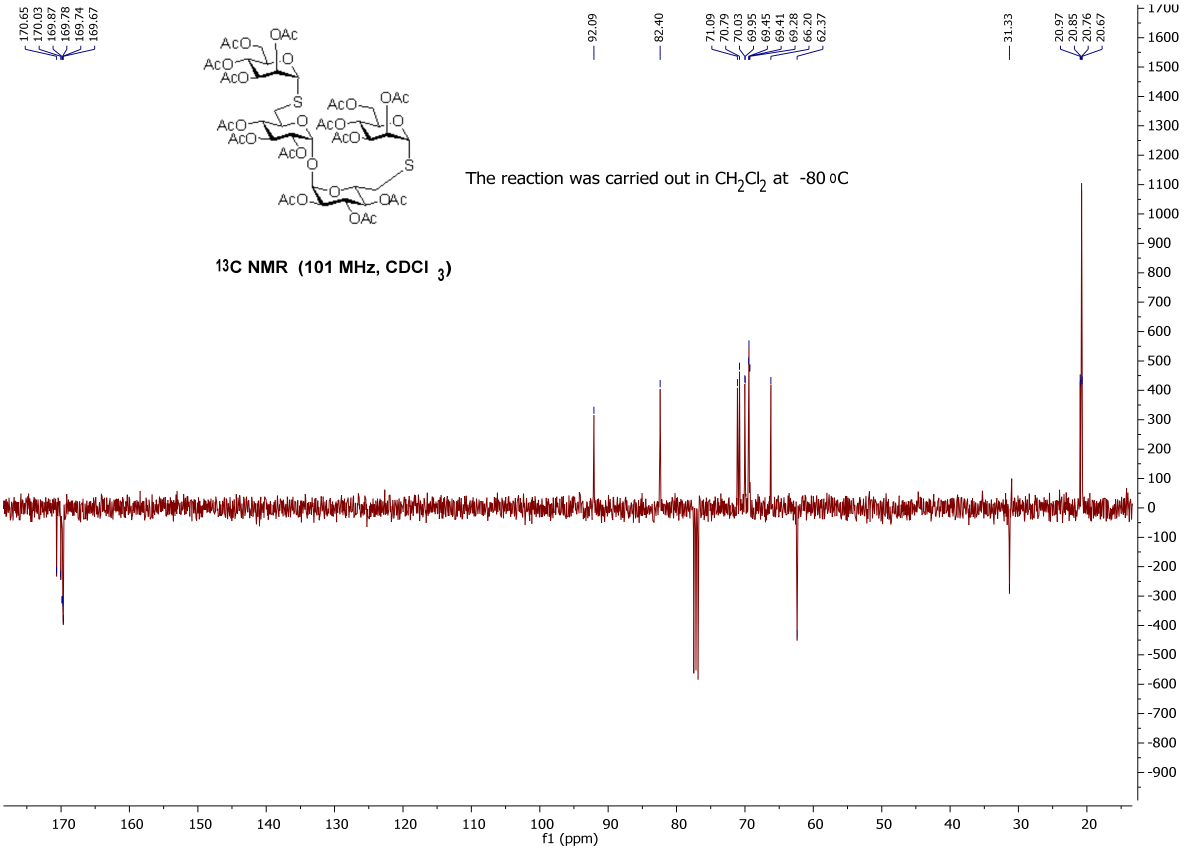

170.65
170.03
169.87
169.78
169.74
169.67
92.09
82.40
71.09
70.79
70.03
69.95
69.45
69.41
69.28
66.20
62.37
31.33
20.97
20.85
20.76
20.67
The reaction was carried out in CH_2Cl_2 at -80 0C
13C NMR (101 MHz, CDCl 3)
f1 (ppm)

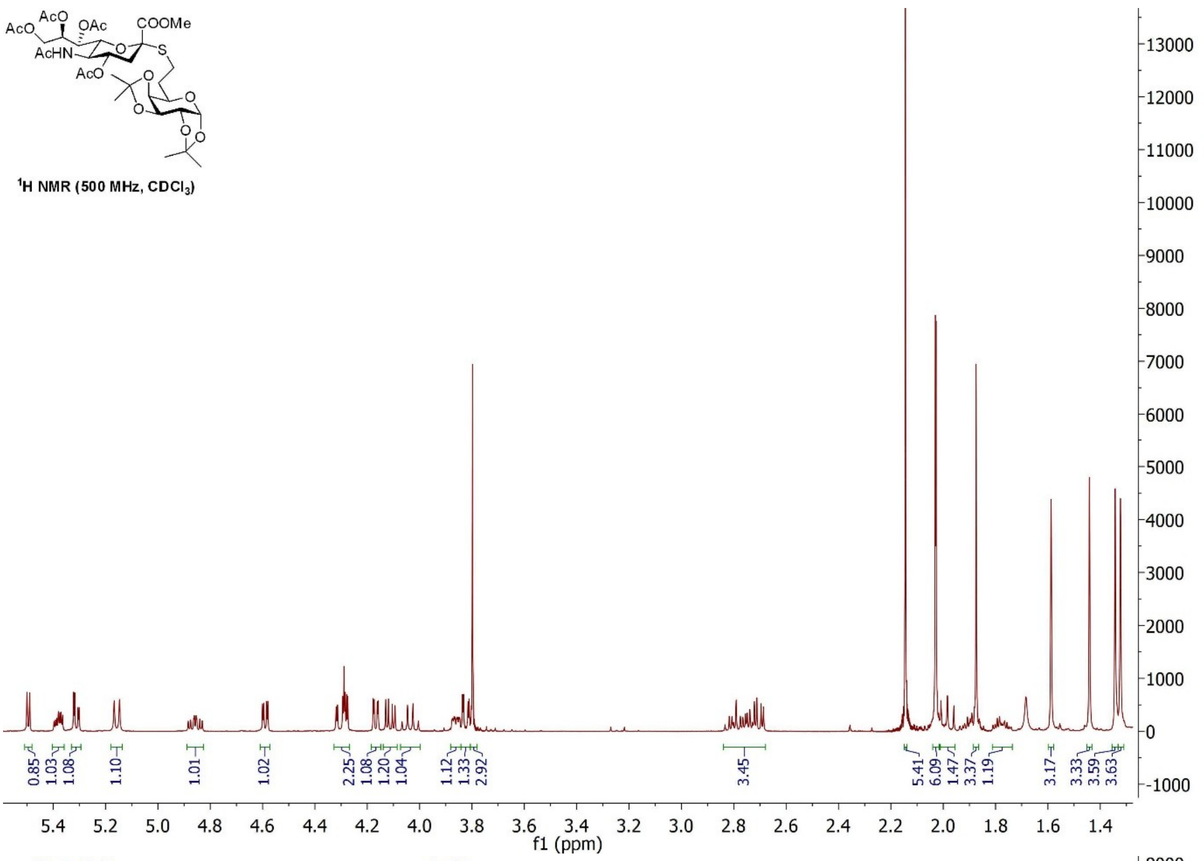
AcO AcO OAc COOMe AcHN O S AcO O O O O O
¹H NMR (500 MHz, CDCl₃)
0.85 1.03 1.08 1.10 1.01 1.02 2.25 1.08 1.20 1.04 1.12 1.33 2.92 3.45 5.41 6.09 1.47 3.37 1.19 3.17 3.33 3.59 3.63
f1 (ppm)

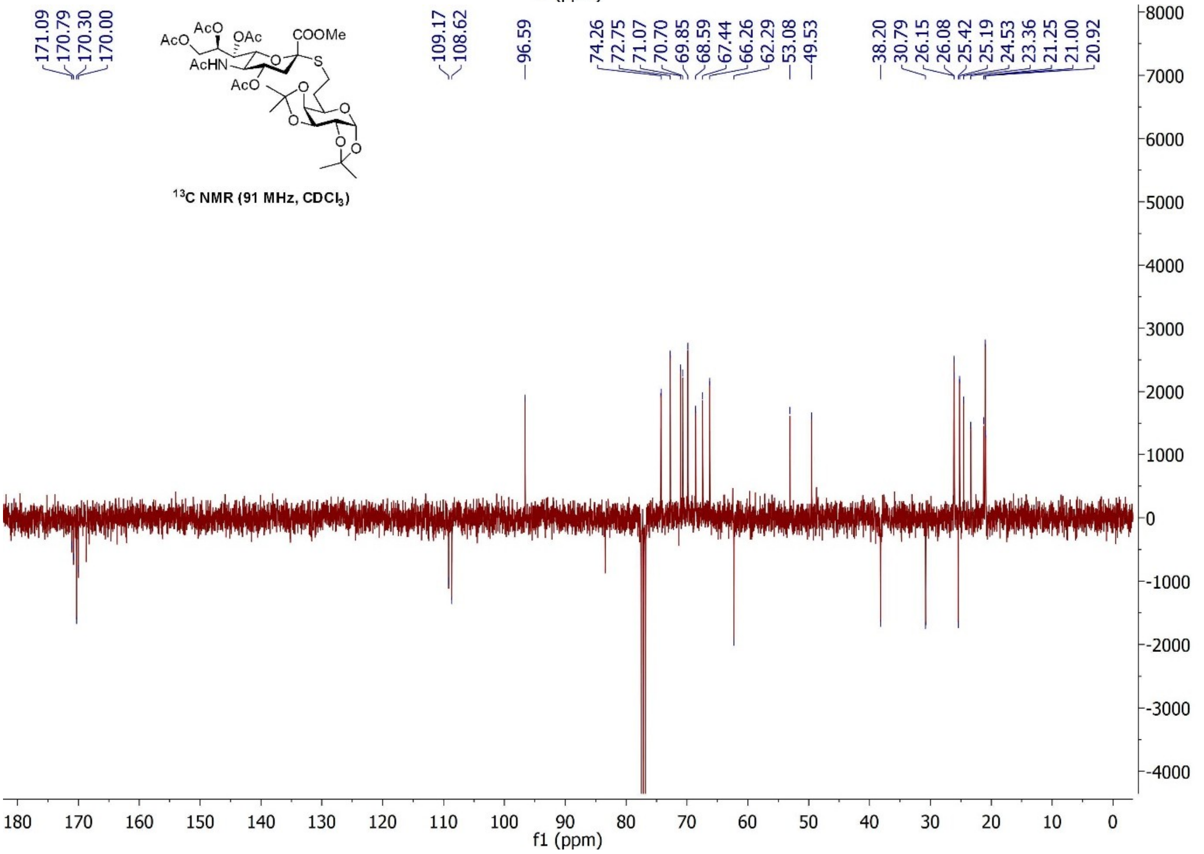
171.09 170.79 170.30 170.00
109.17 108.62
96.59
74.26 72.75 71.07 70.70 69.85 68.59 67.44 66.26 62.29 53.08 49.53
38.20 30.79 26.15 26.08 25.42 25.19 24.53 23.36 21.25 21.00 20.92
¹³C NMR (91 MHz, CDCl₃)
f1 (ppm)

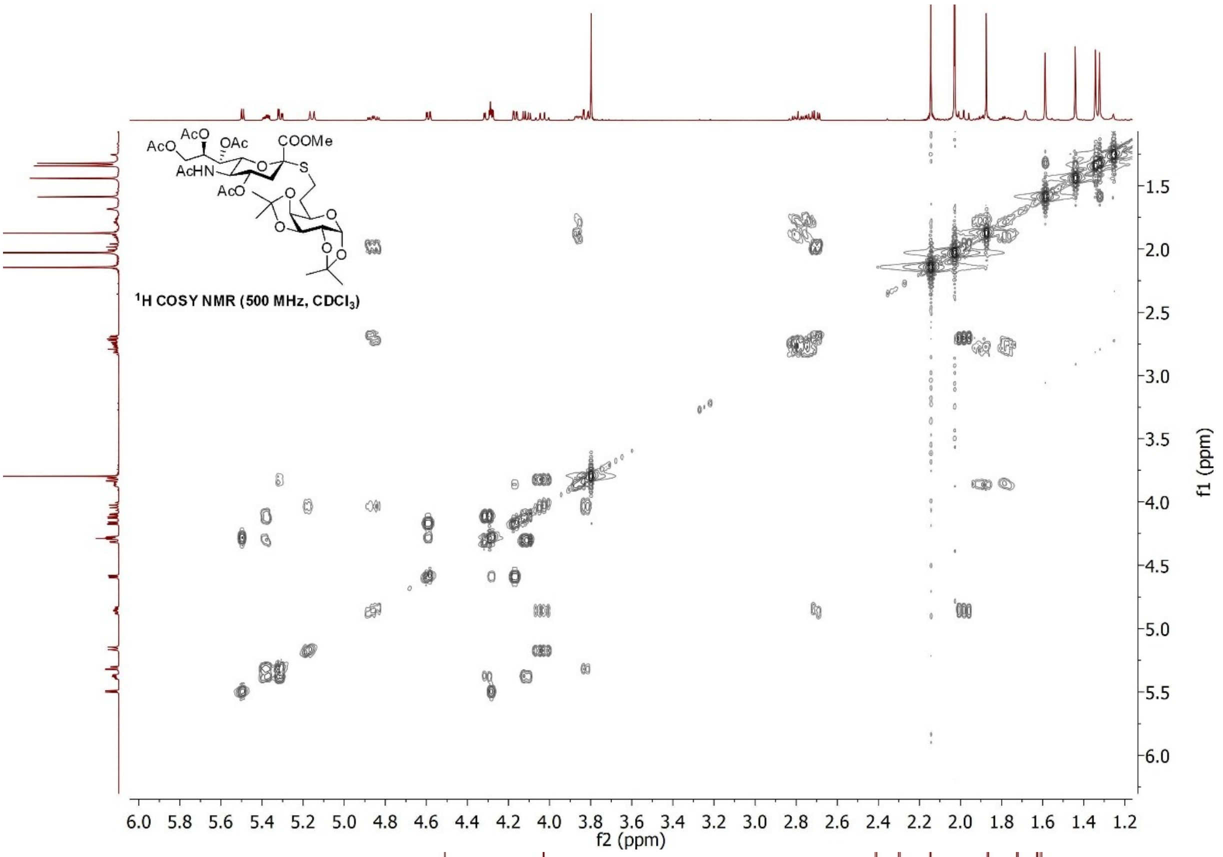
AcO
AcO
OAc
COOMe
S
AcHN
AcO
O
O
O
O
O
O
^{1}H COSY NMR (500 MHz, $CDCl_3$)
f2 (ppm)
f1 (ppm)

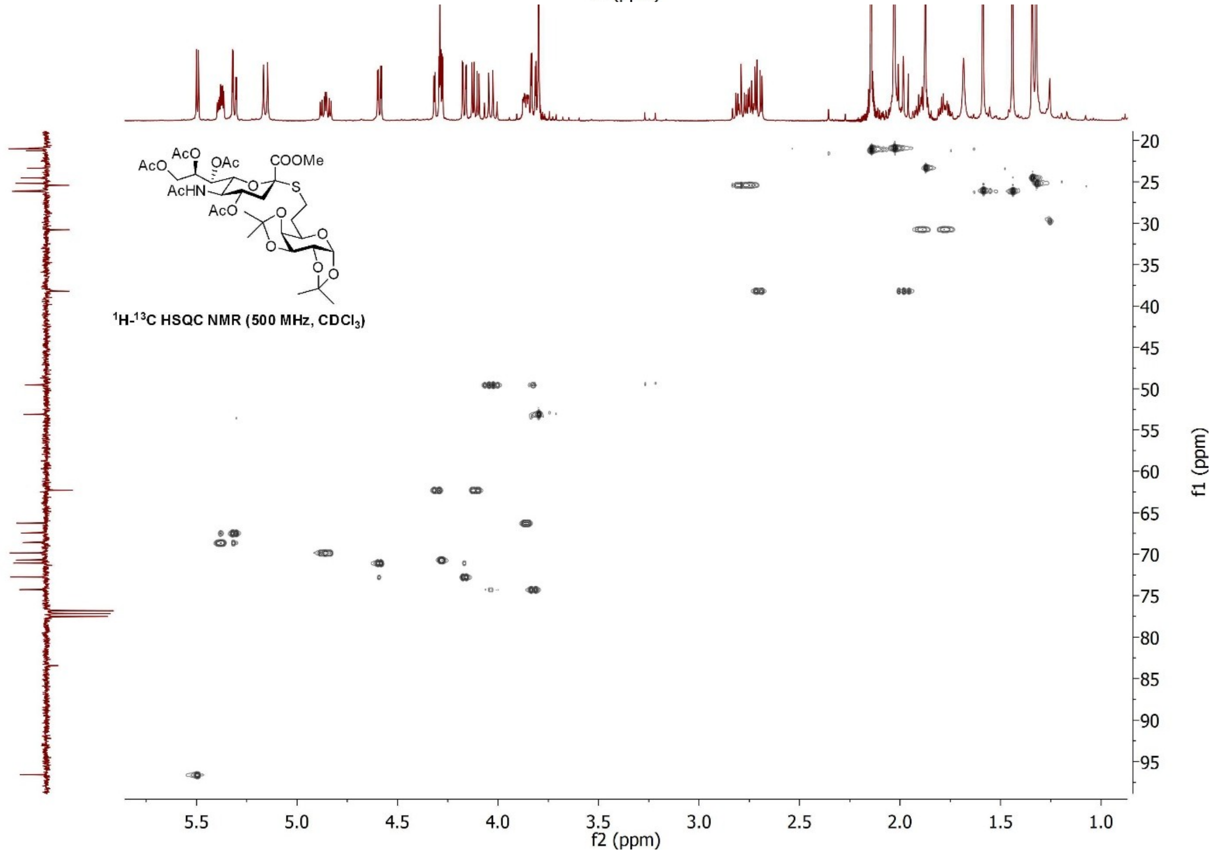
AcO
AcO
OAc
COOMe
S
AcHN
AcO
^{1}H-^{13}C HSQC NMR (500 MHz, $CDCl_3$)
f2 (ppm)
f1 (ppm)
